# Thiete Dioxides as Templates Towards Twisted Scaffolds and Macrocyclic Structures

**DOI:** 10.1002/chem.201905751

**Published:** 2020-04-28

**Authors:** Andreas N. Baumann, Felix Reiners, Alexander F. Siegle, Peter Mayer, Oliver Trapp, Dorian Didier

**Affiliations:** ^1^ Department of Chemistry Ludwig-Maximilians University Butenandtstraße 5–13 81377 Munich Germany

**Keywords:** axial chirality, C−H functionalization, four-membered rings, macrocycles, thiete dioxides

## Abstract

Thiete dioxide units have been employed as a template for further functionalization through C−H activation strategies. Using simple thiete dioxide building blocks, a new library of axially chiral molecules has been synthesized that owe their stability to electrostatic interactions in the solid state. Similar starting materials were further engaged in the formation of cyclic trimeric structures, opening the pathway to unprecedented macrocyclic ring systems.

## Graphical abstract

**C−H activation**: Thiete dioxide units have been employed as a template for further functionalization through C−H activation strategies. Using simple thiete dioxide building blocks, a new library of axially chiral molecules has been synthesized. Similar starting materials were further engaged in the formation of novel cyclic trimeric structures (see scheme).

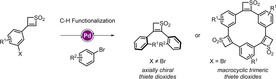

## Introduction

Thiete dioxides possess unique electronic and structural properties.^1^ Although natural products containing thiete cores were not to be found in the literature, they constitute an interesting entry point to their saturated analogs, thietanes, which have found applications in drug discovery or demonstrated their interesting properties in life‐science as pesticide or sweetener.^2^

In the course of our program dedicated to the development of efficient routes towards unsaturated four‐membered rings, we recently put together simple strategies for the synthesis and functionalization of cyclobutenes,^3^ azetines^4^ and 2 H‐thiete dioxides,^5^ as well as their involvement in accessing sophisticated fused ring systems.^6^ Concerning 2 H‐thiete dioxides, two sequences were developed. While the first one relies on an *α‐*lithiation/transmetalation/Negishi cross‐coupling sequence, the second is based on C−H activation strategy and allows for a broad and tolerant functionalization of the unsaturated S‐containing four‐membered ring scaffold.

**Scheme 1:**
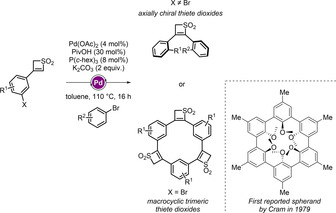
Thiete dioxide units as a common template for accessing axially chiral disubstituted structures and macrocyclic analogues to the known spherand, first reported by Cram.

Given that such structures constitute a unique entry in the repertoire of strained heterocycles, we set out to take the next step in their architectural diversification. We describe herein the study of palladium‐catalyzed C−H functionalization of 3‐substituted thiete dioxides towards the formation of chiral disubstituted thiete dioxides and thiete dioxide‐based macrocycles (Scheme 1). Axial chirality is of great importance in many areas of chemistry and is not limited to the synthesis of novel catalysts^7^ and atropisomers^8^ but plays an important role in drug design.^9^ Beside that, thiete dioxide‐based macrocycles can be interesting analogs to the known compound class, namely spherands, which were first reported by Cram in 1979.^10^ They can be classified as macrocyclic ligands with limited conformational flexibility. With a preorganization and an electron‐pair‐lined cavity, they are strong complexation receptors for ions, such as lithium, sodium or potassium.^11^ Indeed, the binding constant for lithium ions is one of the strongest reported to date.^12^, ^13^ With this in mind, the preparation of thiete dioxide based macrocyclic structures was envisioned. Therefore, 3‐substituted thiete dioxide units bearing a bromide at the *meta* position of the aryl substituent were utilized (Scheme 1).

## Results and Discussion

**Scheme 2:**
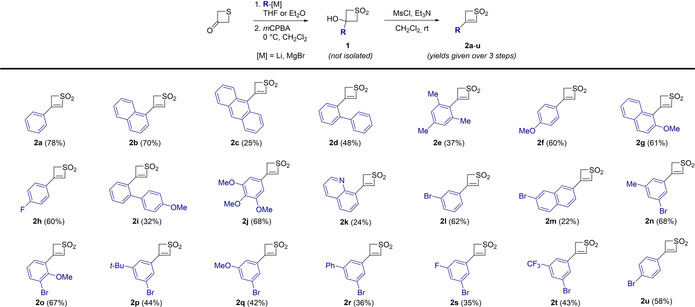
Synthetic scope of monofunctionalized thiete dioxides.

The project was initiated by synthesizing a range of 3‐substituted thiete dioxide building blocks. Starting from commercially available 3‐thietanone, the addition of an organomagnesium or an organolithium furnished the corresponding tertiary alcohols after hydrolysis (Scheme 2). The crude product was subsequently oxidized with *m*CPBA to give thietane **1**. Desired building blocks **2 a**–**t** were obtained upon addition of mesyl chloride and trimethylamine, triggering a β‐elimination to generate the double bond. For simple aromatic systems (phenyl, naphthyl, anthryl), we have witnessed a general decrease in efficiency with increasing sterical hindrance (from 78 % for **2 a** to 25 % for **2 c**). However, the procedure proved to be quite versatile, allowing for the introduction of a variety of substituents, including electron deficient groups (*p*‐fluorophenyl, *m*‐trifluomethylphenyl or 8‐quinolinyl) and electron rich substituents (*p*‐, *m*‐ or *o*‐methoxyphenyl). A broad range of functionalized compounds (**2 a**–**u**) was isolated in moderate to good yields over three synthetic steps (22 to 78 %).

**Scheme 3:**
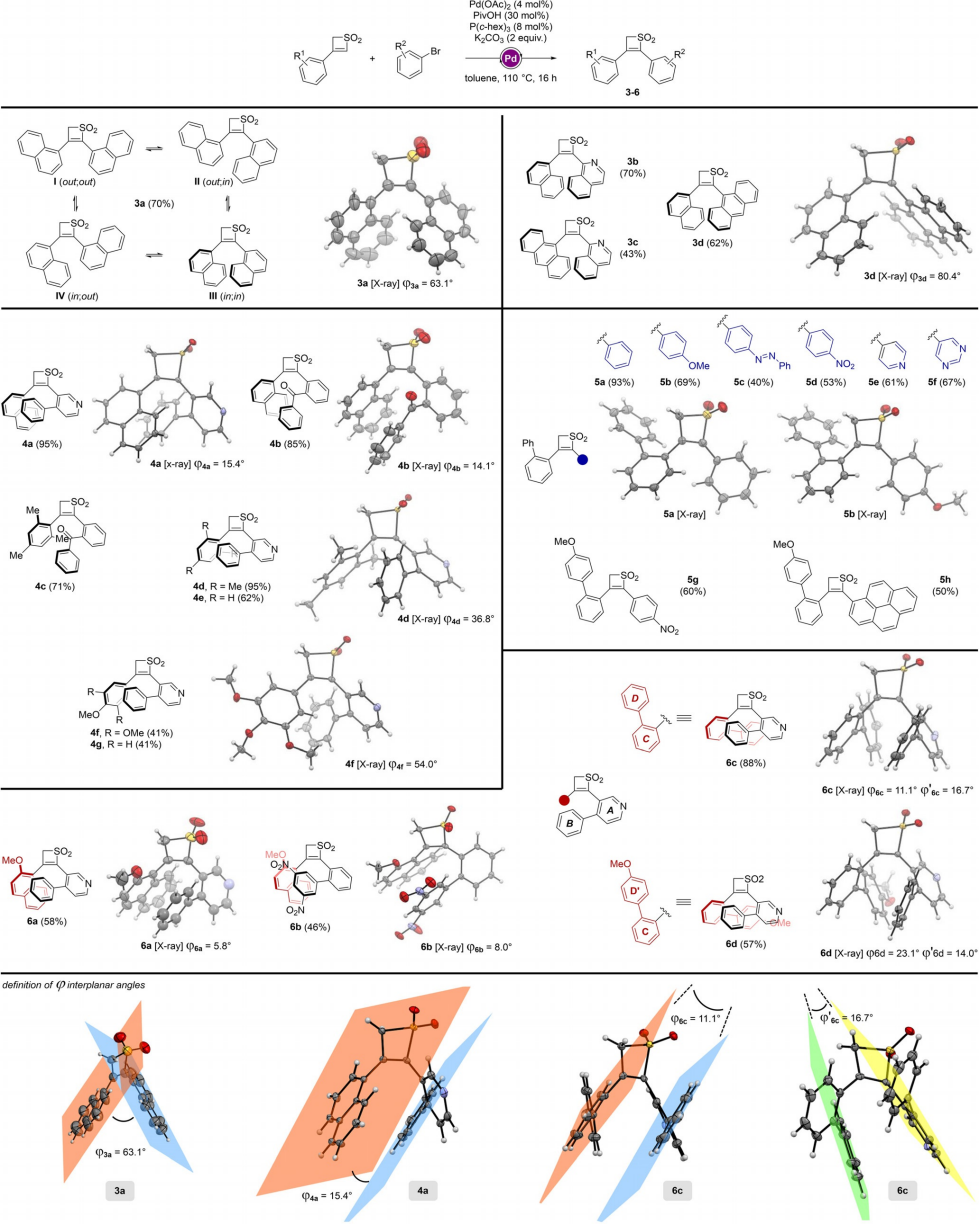
Diversification of the axially chiral thiete dioxides library.

In the course of our study on the functionalization of thiete dioxides through C−H activation, we synthesized a range of bis‐arylated structures such as **3 a** (70 %), employing catalytic amounts of Pd(OAc)_2_ in the presence of pivalic acid, tricyclohexyl phosphine and potassium carbonate (Scheme 3). In our previous report, we showed that such catalytic system preferentially follows a BIES (base‐assisted intramolecular electrophilic substitution) mechanism. With two naphthyl groups at positions 3 and 4, four different conformers of **3 a** (*out*;*out*, *out*;*in*, *in*;*out* and *in*;*in*) can be anticipated. Although steric factors could have ruled out conformers **II**, **III** and **IV**, a crystal structure of compound **3 a** (Scheme 3) showed that conformer **III** (*in*;*in*) is exclusively observed in the solid state.^14^

In **3 a**(**III**), two σ‐bonds are twisted, providing the molecule with a helical shape. In addition to steric factors, it has been demonstrated that electronic effects can play a determinant role in the geometrical arrangement of electron‐poor strained ring systems, as in the present case.^15^ The observation of such thiete dioxide‐based double axial chirality in the solid state might provide the opportunity towards the development of novel chiral scaffolds. However, despite having evidence for chirality in the crystal structure of **3 a**(**III**), we questioned the configurational stability of the structures in solution, as no evidence for enantiomer separation could be demonstrated under various chromatographic conditions, probably due to a low rotation barrier. We became interested in studying the influence of substituents and electronic effects on both aryl parts of the structure.

We first investigated the influence of different aromatic groups on the axial chirality. Compounds **3 b** and **3 c** were synthesized from **2 b** and **2 c**, respectively and 1‐bromoisoquinoline in 43–70 % yield. 9‐Bromoanthracene was also used as a cross‐coupling partner, giving **3 d** in 62 %, that also showed axial chirality in the solid state.

However, the rigidity of the two aryl groups results in interplanar angles (*φ*) of 63.1° and 80.4° in the structures of **3 a** and **3 d**, respectively, which does not allow them for adapting to one another^16^—separation of enantiomers in solution remained unsuccessful. As Wittig already observed when synthesizing phenanthrene derivatives,^17^ electrostatic interactions play a determinant role in the stabilization of axial chirality. We envisioned to modulate the nature of the aryl substituents to increase their affinity for one another. We therefore designed new structures to evaluate the impact of electron‐donor and electron‐acceptor moieties, as well as their bulkiness, on the stabilization of the stereointegrity.

In order to increase potential interactions, some flexibility was added to the cross‐coupling partner in such way that the molecule could gain stability by decreasing the interplanar angle between the aromatic moieties at positions 2 and 3. Moreover, given the initial polarization of the thiete dioxide moiety,6c electron deficient coupling partners were chosen to be introduced at position 2. With 3‐naphthylthiete dioxide **2 b** as starting material, molecules **4 a** and **4 b** were synthesized from 4‐(2‐bromophenyl)pyridine and (2‐bromophenyl)(phenyl)methanone, respectively (Scheme 3).

As evidenced by X‐ray measurements, these chains seem to fold on top of the naphthyl group. The implementation of flexibility through a keto group or a σ‐bond between the phenyl and the aryl moiety at position 2 allowed for reaching interplanar angles of 15.4° (**4 a**) and 14.1° (**4 b**) between the naphthyl at position 3 and the phenyl group. The bulkiness of the aryl at position 3 was further increased by implementation of a mesityl group (from **2 e**). Similarly, folding of the side phenylpyridyl chain (electron poor) onto the mesityl moiety was observed in the solid state (**4 d**), showing however a wider interplanar angle of 36.8°. Same observations were made when mesityl was replaced by electron‐richer aromatics such as 3,4,5‐trimethoxyphenyl or 4‐methoxyphenyl in structures **4 f** and **4 g**, synthesized from thiete dioxides **2 j** and **2 f**, respectively. It is worth noting that methoxy‐substituted aryls at position 3 are in the same plane as the thiete core (0° dihedral angle), inducing a wider interplanar angle (*φ*_**4**_ **f**=54°). Next, 3‐([1,1′‐biphenyl]‐2‐yl)‐thiete dioxide **2 d** was used as starting material in order to introduce flexibility at position 3.

C−H functionalization was performed with neutral, electron‐rich and electron‐poor aryl and heteroaryl coupling partners, giving products **5 a**–**f** in moderate to good yields, up to 93 %. Surprisingly, the “*out*” conformation of the biphenyl was favored in all cases, as attested by the crystal structures of **5 a** and **5 h**. The absence of folding was also witnessed when electron‐enriched biaryl (*p*‐MeO) was introduced at position 3 (**5 g**, 60 %), in the presence of an electron‐poor phenyl (*p*‐NO_2_). The presence of a larger pyrenyl moiety at position 4 did not positively influence intramolecular interactions (**5 h**). However, the introduction of an electron‐donating group on the naphthyl moiety (2‐methoxynaphthalen‐1‐yl) tremendously decreased the interplanar angle when having flexible electron‐deficient biaryl moieties at position 2, probably due to stronger non‐covalent interactions. Compounds **6 a** and **6 b** were synthesized employing 4‐(2‐bromophenyl)pyridine and 2‐bromo‐3′,5′‐dinitro‐1,1′‐biphenyl, respectively, and displayed torsion angles of 5.8° and 8.0°. Interestingly, splitting in ^13^C NMR signals of **6 a** was observed,^13^ pointing out the higher structural constraint of the molecule. Although examples **5 a**–**h** did not show any folding in the solid state, the presence of an additional flexible chain at position 2 allowed for both biaryl to fold onto one another. Starting from 3‐substituted thiete dioxides **2 d** and **2 i**, tetraarylated scaffolds **6 c** and **6 d** were generated in 57 to 88 % yield using 4‐(2‐bromophenyl)pyridine. X‐ray measurements revealed that the flexibility given to both chains at positions 3 and 2 was profitable to the system, allowing for a better adaptation of the different groups with their respective counterpart. For instance, the electron‐rich phenyl moiety ***C*** in compound **6 c** was found to be placed in opposition to the phenyl group ***B*** with a torsion angle of 11.1°, and the electron‐poor pyridyl group ***A*** opposes the phenyl group ***D*** with a torsion angle of 16.7°. Similar observations were made for compound **6 d**, although a wider angle of 23.1° was measured between aromatic rings ***B*** and ***C***. Unfortunately, despite having optimized the electrostatic interactions, none of the abovementioned examples showed any stable chirality in solution. Tuning the electronic properties of the substituents as well as their bulkiness did not allow for two enantiomers to be observed, even at low temperature, pointing out the low rotation barrier of these systems.

The variations of both substituents and coupling partners allowed for the synthesis of a wide range of sophisticated structures with specific geometries. To push the diversification further, we envisioned to employ thiete dioxide units that could act as the coupling partner, as a template in the C−H functionalization step. For this purpose, mono‐arylated thiete dioxides possessing a bromide at the *meta* position of the aryl moiety (**2 l**, **n**, **p**–**u**) were engaged in the presence of the above‐mentioned catalytic system.

**Scheme 4:**
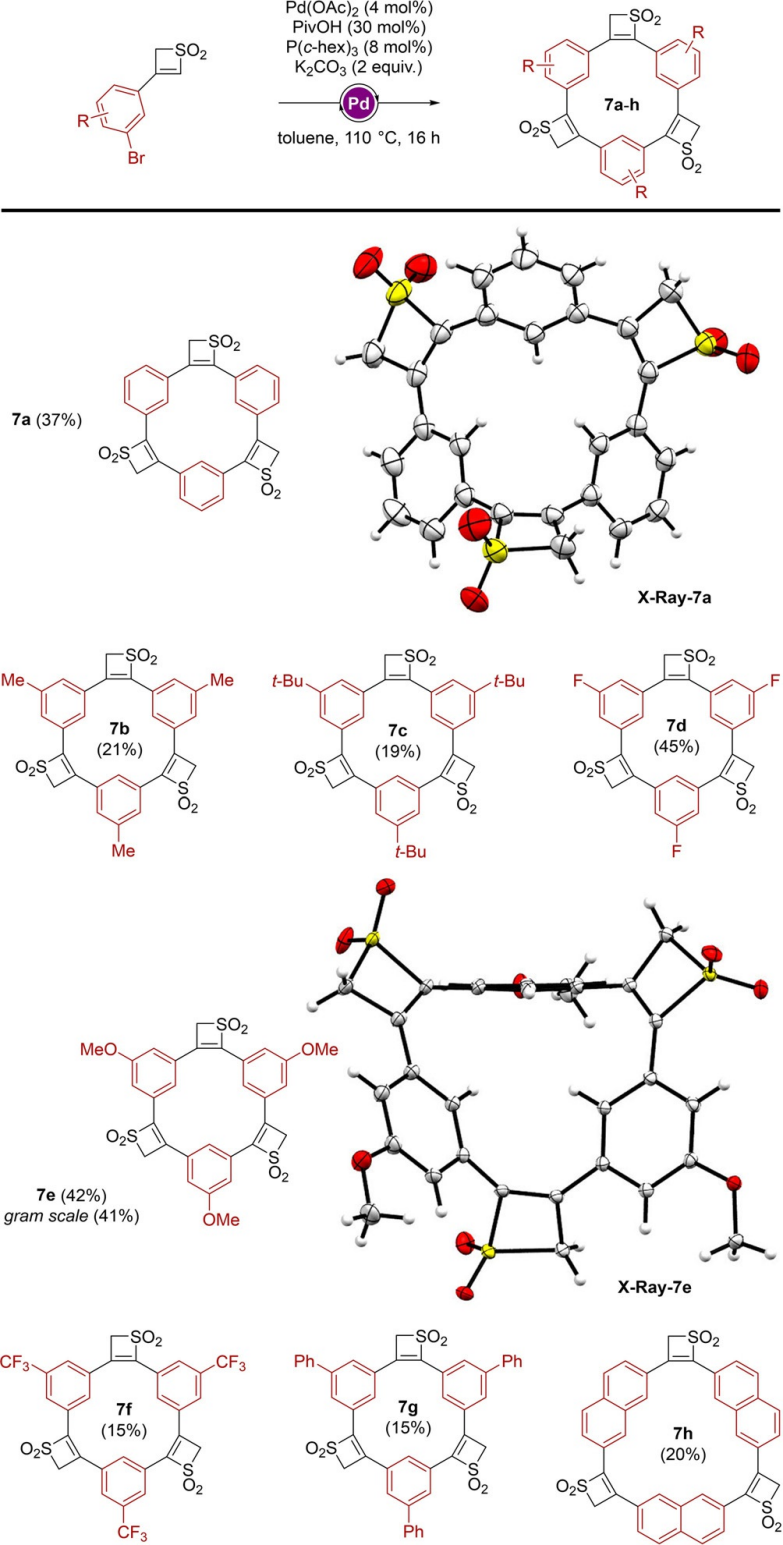
Thiete dioxide units as a base for macrocyclic structure formation.

Although different products of successive couplings can be expected (linear oligomers or macrocycles), trimeric macrocycles were observed as the major components of the reaction (Scheme 4). Given the specific geometry of the starting material, it was postulated that the entropic factor plays a determinant role in the formation of the trimers, disfavoring larger sizes of cyclic structures (although they were observed as side products in mass spectroscopy in certain cases). Even though full conversion of starting materials **2** was observed after 16 h, **7 a**–**h** were obtained in low to moderate yields (15 to 45 %), which we attributed to the very low solubility of the products in common solvents. Best yields (42 to 45 %) were obtained for *m*‐methoxy and *m*‐fluoro‐phenyl derivatives (**7 e** and **7 d**) and the reaction proved to tolerate bulkier substituents like *tert‐butyl* and phenyl groups at the *meta* position with reduced isolated yields (**7 c** and **7 f,g**, 15 to 19 %). Considering procedures for the synthesis of related macrocyclic spherands, the yields between 20 and 45 % are quite good. Moreover, a gram scale procedure of macrocycle **7 e** was successful conducted in similar yield of 41 %. Interestingly, we were able to synthesize larger trimeric cyclic “naphthyl‐thiete dioxide‐based macrocycle” **7 h**, for which the solubility problems in classical solvents did not allow for its isolation in more than 20 % yield. Crystal structures of **7 a** and **7 e** confirm the cyclic structures, consisting of three thiete dioxide units. Moreover, due to non‐aromaticity of the cyclic system and sterical repulsion a non‐planar ring system can be observed in the X‐ray structures.

**Scheme 5:**
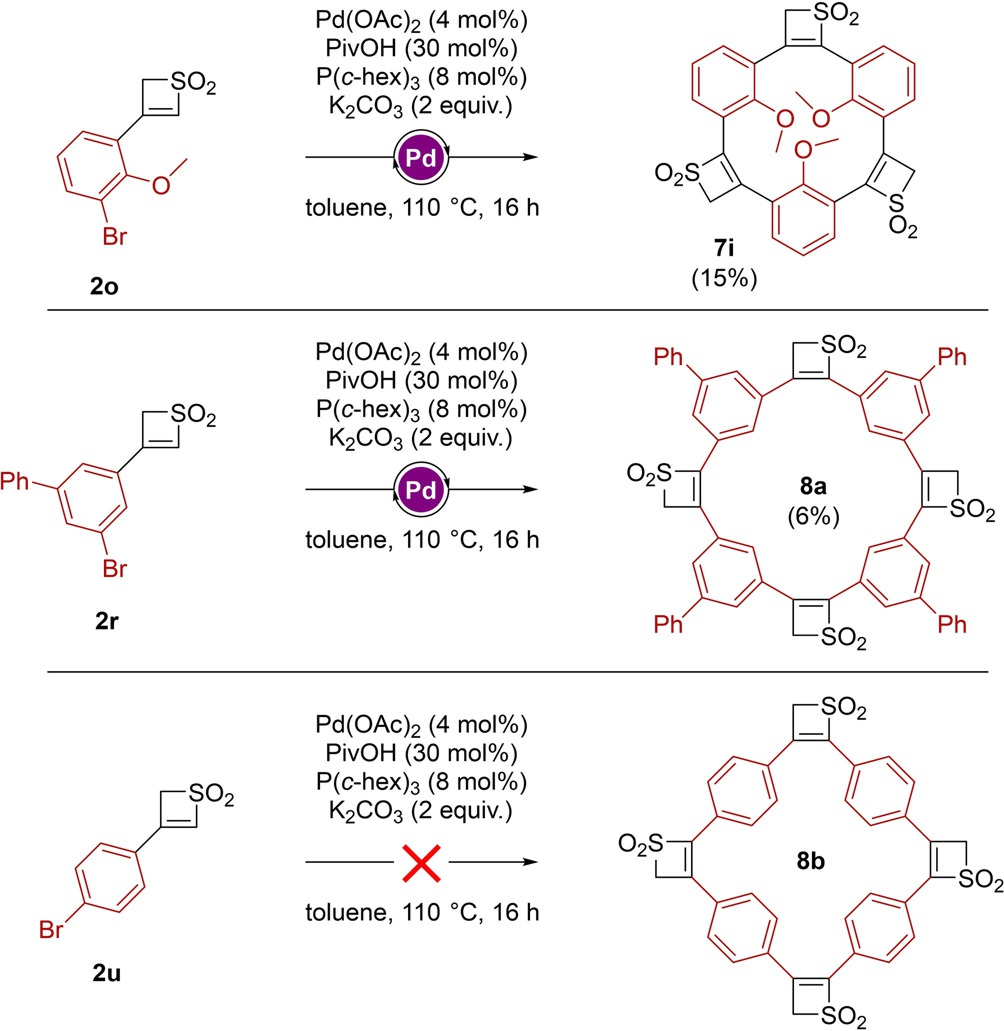
Attempts to synthesize alternative cyclic structures.

As a next step, we attempted to form a macrocycle showing more structural similarity to the spherand reported by Cram, which bears methoxy‐groups inside the cavity (Scheme 1).^8^ For this purpose, **2 o** was chosen as the building unit. The reaction resulted in the trimeric structure **7 i**, giving a modest yield of 15 % (Scheme 5). Although the compound was identified in mass spectrometry, ^1^H and ^13^C NMR remained inconclusive and no crystal structure could be obtained. After further investigation of the reaction, we observed that in certain cases a tetrameric structure was formed. When employing **2 r** as the thiete dioxide‐unit, we were able to not only isolate structure **7 g**, but also isolate the tetrameric structure **8 a** in a very modest yield of 6 %. Despite the bad solubility of the compound, we were able to record a ^1^H spectrum, showing widely broadened signals compared to the spectrum of the corresponding trimer **7 g**. This phenomenon can be attributed to the constricted flexibility of such system, and conducting NMR measurements at higher temperature allowed us to acquire sharper signals. Furthermore, we employed the *p*‐bromo‐phenyl thiete dioxide **2 u** in the attempt to form the tetramer **8 b**. Unfortunately, no reaction was observed.

## Conclusions

In this work, we at first expanded our scope of 3‐substituted thiete dioxide‐based building blocks, forming new compounds containing bulkier aromatic substituents, as well as new functional groups. We were then able to functionalize these, following a simple C−H‐activation strategy. Thereby, we created a new library of 2,3‐disubstituted thiete dioxides, showing axial chirality in the solid state. We then showed that the thiete dioxide unit can also act as the coupling partner itself, allowing for the synthesis of novel macrocyclic compounds. In conclusion, our results show that the thiete dioxide moiety can be used as a very versatile platform for molecular design, which makes it an attractive structure for further investigation.

## Experimental Section

### General procedure A: For the synthesis of thiete dioxides 2 a–u:

A flask was charged with thietan‐3‐one (10.0 mmol, 1.0 equiv) and THF (0.5 m) was added. The reaction mixture was cooled to −78 °C and a solution of organolithium reagent (1.30 equiv) was added dropwise. Alternatively, the reaction mixture was cooled to −30 °C and a solution of organomagnesium reagent (1.30 equiv) was added dropwise. After stirring for 60 min the mixture was brought to ambient temperature and quenched with a solution of saturated aqueous NH_4_Cl. The aqueous phase was extracted with dichloromethane (3×50 mL) and washed with a solution of saturated aqueous NaCl (1×50 mL). The combined organic phases were dried over magnesium sulfate and concentrated in vacuo. The residue, containing the thietanol, was dissolved in dichloromethane (50 mL), cooled to 0 °C and *m*CPBA (20.0 mmol, 2.0 equiv, 77 %) was added portion wise. After TLC showed full conversion of the thietanol (approx. 10 min) water was added. The aqueous phase was extracted with dichloromethane (3×50 mL) and washed with a solution of saturated aqueous NaCl (1×50 mL). The combined organic layers were dried over magnesium sulfate, filtered and concentrated in vacuo. The residue, containing the thietanol dioxide **1**, was dissolved in dichloromethane (50 mL) and triethylamine (30 mmol, 3.0 equiv) was added. Mesyl chloride (30 mmol, 3.0 equiv) was subsequently added dropwise and the mixture was stirred until TLC indicated full conversion of the starting thietanol dioxide **1** (approx. 30 min) water was added. The aqueous phase was extracted with dichloromethane (3×50 mL) and washed with a solution of saturated aqueous NaCl (1×50 mL). The combined organic phases were dried over magnesium sulfate, filtered and concentrated in vacuo. The crude thiete dioxides **2 a**–**u** were purified by flash column chromatography with appropriate solvent mixtures.

### Representative example—synthesis 2 g

Using (2‐methoxynaphthalen‐1‐yl)magnesium bromide according to general procedure A, provided **2 g** (1.59 g, 6.1 mmol, 61 %) as a white solid. *R*_f_=0.2 (hexane/EtOAc 7:3, UV, KMnO_4_, PAA). ^1^H NMR (400 MHz, CDCl_3_): *δ*=8.07–8.02 (m, 1 H), 7.96 (d, *J=*9.2 Hz, 1 H), 7.86–7.81 (m, 1 H), 7.59–7.52 (m, 1 H), 7.46–7.39 (m, 1 H), 7.29 (d, *J=*9.1 Hz, 1 H), 7.00 (s, 1 H), 5.08 (s, 2 H), 4.00 ppm (s, 3 H). ^13^C NMR (101 MHz, CDCl_3_): *δ*=156.2, 145.1, 143.9, 133.4, 131.5, 129.0, 129.0, 128.5, 124.5, 123.3, 112.4, 112.2, 74.5, 56.4 ppm. HRMS (EI‐Orbitrap): *m*/*z*: [*M*]+ Calcd for C_14_H_12_O_3_^32^S^+^: 260.0507; found: 260.0501. IR (Diamond‐ATR, neat): $\tilde{\nu }$ =1286 (vs.), 1277 (s), 1255 (m), 1193 (vs.), 1167 (s), 1156 (m), 1134 (s), 1116 (s), 1094 (s), 1065 (s), 1056 (s), 1027 (m), 820 (s), 790 (s), 750 (s), 668 cm^−1^ (s). Melting point: 135(±2) °C.

### General procedure B: For the synthesis of 2,3‐disubstituted thiete dioxides 3–6

A pressure tube was charged with 2*H*‐thiete 1,1‐ dioxide derivatives **2 a**–**k** (0.2 mmol, 1 equiv) and 2 mL toluene was added. Subsequently were added K_2_CO_3_ (55 mg, 0.4 mmol, 2.0 equiv), Pd(OAc)_2_ (1.8 mg, 8 μmol, 4 mol %), tricyclohexylphosphane (PCy_3_) (4.5 mg, 16 μmol, 8 mol %), the corresponding halogenide (0.3 mmol, 1.5 equiv) and a few drops of pivalic acid (≈7 μL, 30 mol %). The mixture was stirred at 110 °C in the sealed pressure tube until TLC showed consumption of the starting 2*H*‐thiete 1,1‐ dioxides (approx. 16 hours). After cooling to ambient temperature, the tube was opened and a 1:1 mixture of H_2_O:Et_2_O (4 mL) was added. The aqueous phase was extracted with Et_2_O (3×20 mL). The combined organic phases were dried over magnesium sulfate, filtrated, concentrated in vacuo and purified by flash‐column chromatography on silica gel with the appropriate solvent mixture to obtain pure **3**–**6**.

### Representative example—synthesis 4 b

Using (2‐bromophenyl)(phenyl)methanone according to general procedure B provided **4 b** (70 mg, 0.17 mmol, 85 %) as a white solid. *R*_f_=0.2 (hexane/EtOAc 8:2, UV, KMnO_4_, PAA). ^1^H NMR (400 MHz, CDCl3): *δ*=7.92 (dd, *J=*7.7, 1.1 Hz, 1 H), 7.67 (dd, *J=*8.3, 1.2 Hz, 1 H), 7.63–7.53 (m, 3 H), 7.46–7.34 (m, 3 H), 7.29 (ddd, *J=*8.3, 6.8, 1.4 Hz, 1 H), 7.25–7.17 (m, 3 H), 7.10 (t, *J=*7.8 Hz, 2 H), 7.04 (dd, *J=*8.3, 1.5 Hz, 2 H), 4.87 ppm (s, 2 H). ^13^C NMR (101 MHz, CDCl3): *δ*=194.4, 151.0, 141.3, 139.0, 135.0, 133.6, 132.9, 131.3, 131.2, 129.9, 129.8, 129.6, 129.3, 129.3, 128.9, 127.9, 127.5, 127.2, 127.1, 126.5, 126.5, 125.0, 124.8, 72.5 ppm. LRMS (DEP/EI‐Orbitrap): *m*/*z* (%): 346.1 (30), 331.1 (15), 239.1 (60). HRMS (EI‐Orbitrap): *m*/*z*: [*M*−SO_2_]+ Calcd for C_26_H_18_O^+^: 346.1358; found: 346.1359. IR (Diamond‐ATR, neat): $\tilde{\nu }$ =1656 (s), 1299 (s), 1286 (m), 1266 (m), 1256 (m), 1184 (m), 1135 (s), 1114 (m), 927 (m), 799 (m), 777 (s), 761 (m), 704 cm^−1^ (vs.). Melting point: 181(±2) °C.

### General procedure C: For the synthesis of macrocyclic structures 7 a–i

A pressure tube was charged with 2*H*‐thiete 1,1‐ dioxides derivative **2 l**–**u** (0.2 mmol, 1 equiv) and 2 mL toluene was added. Subsequently were added K_2_CO_3_ (55 mg, 0.4 mmol, 2.0 equiv), Pd(OAc)_2_ (1.8 mg, 8 μmol, 4 mol %), tricyclohexylphosphane (PCy_3_) (4.5 mg, 16 μmol, 8 mol %) and a few drops of pivalic acid (≈7 μL, 30 mol %). The mixture was stirred at 110 °C in the sealed pressure tube until TLC showed consumption of the starting 2 H‐thiete 1,1‐ dioxides (approx. 16 hours). After cooling to ambient temperature, the tube was opened and a 1:1 mixture of H_2_O:CH_2_Cl_2_ (4 mL) was added. The aqueous phase was extracted with CH_2_Cl_2_ (3×20 mL). The combined organic phases were dried over magnesium sulfate, filtrated, concentrated in vacuo and purified by flash‐column chromatography on silica gel with the appropriate solvent mixture to obtain pure **7 a**–**i**.

### Representative example—synthesis 7 d

Using 3‐(3‐bromo‐5‐methoxyphenyl)‐2*H*‐thiete 1,1‐dioxide (**2 q**) according to general procedure C, provided **7 d** (2.14 g, 3.43 mmol, 42 %) as yellowish solid. *R*_f_=0.2 (hexane/EtOAc 5:5, UV, KMnO_4_). ^1^H NMR (400 MHz, CDCl_3_): *δ*=7.27 (s, 3 H), 7.22–7.20 (m, 3 H), 6.89–6.87 (m, 3 H), 4.83 (s, 6 H), 3.89 ppm (s, 9 H). ^13^C NMR (101 MHz, CDCl_3_): *δ*=160.8, 150.6, 139.7, 132.1, 128.8, 119.0, 116.7, 114.7, 72.1, 56.0 ppm. HRMS (ESI‐Quadrupole): *m*/*z*: [*M*+‐H] Calcd for C_30_H_23_O_9_^32^S_3_^+^: 623.0504; found: 623.0507. IR (Diamond‐ATR, neat): $\tilde{\nu }$ =1585 (m), 1362 (m), 1291 (s), 1261 (s), 1226 (s), 1182 (s), 1156 (m), 1129 (vs.), 1064 (s), 1050 (s), 1017 (m), 1000 (s), 985 (s), 861 (m), 822 (m), 806 (m), 800 (m), 736 cm^−1^ (m). Melting point: 265(±2) °C decomposition.

## Conflict of interest

The authors declare no conflict of interest.

## Supporting information

As a service to our authors and readers, this journal provides supporting information supplied by the authors. Such materials are peer reviewed and may be re‐organized for online delivery, but are not copy‐edited or typeset. Technical support issues arising from supporting information (other than missing files) should be addressed to the authors.

Supplementary
