# Supplementary material for: Thiete Dioxides as Templates Towards Twisted Scaffolds and Macrocyclic Structures
Source: Chemistry. 2020 Apr 28;26(27):6029–35. doi: 10.1002/chem.201905751 (PMC7318563; doi:10.1002/chem.201905751)
Supplement: Supplementary file 1 — Supplementary [file CHEM-26-6029-s001.pdf]

Chemistry–A European Journal

Supporting Information

Thiete Dioxides as Templates Towards Twisted Scaffolds and Macrocyclic Structures

Andreas N. Baumann+, Felix Reiners+, Alexander F. Siegle, Peter Mayer, Oliver Trapp, and Dorian Didier*[a]

1. General Considerations 2

2. Experimental Procedures 4

2.1 General Procedures 4

2.2 Experimental Data 6

3. NMR Spectra 29

4. Screening of Helicoidal Compounds 72

4.1 Column Screening 72

4.2 Low Temperature Measurements 76

5. NMR Measurements on 6a/6d 79

6. Single Crystal X-Ray Diffraction 80

1. General considerations

Commercially available starting materials were used without further purification unless otherwise stated. All reactions were carried out under N_2 atmosphere in flame-dried glassware. Syringes, which were used to transfer anhydrous solvents or reagents, were purged with nitrogen prior to use. THF was refluxed and distilled from sodium benzophenone ketyl under nitrogen. Et_2O was predried over $CaCl_2$ and passed through activated Al_2O_3 (the solvent purification system SPS-400-2 from Innovative Technologies Inc.). Chromatography purifications were performed using silica gel (SiO_2, 0.040-0.063 mm, 230-400 mesh ASTM) from Merck. The spots were visualized under UV (254 nm) or by staining the TLC with $KMnO_4$ solution (K_2CO_3, 10 g – $KMnO_4$, 1.5 g – H_2O, 150 mL – NaOH 10% in H_2O, 1.25 mL), PAA: *p*-anisaldehyde solution (conc. H_2SO_4, 10 mL – EtOH, 200 mL – AcOH, 3 mL – *p*-anisaldehyde, 4 mL). ^{13}C and ^{1}H NMR spectra were recorded on VARIAN Mercury 200, BRUKER ARX 300, VARIAN VXR 400 S and BRUKER AMX 600 instruments. Chemical shifts are reported as δ values in ppm relative to residual solvent peak (^{1}H-NMR) or solvent peak (^{13}C-NMR) in deuterated chloroform ($CDCl_3$: δ 7.26 ppm for ^{1}H-NMR and δ 77.16 ppm for ^{13}C-NMR) or deuterated benzene (C_6D_6: δ 7.16 ppm for ^{1}H-NMR and δ 128.06 ppm for ^{13}C-NMR). Abbreviations for signal coupling are as follows: s (singlet), d (doublet), t (triplet), q (quartet), quint (quintet), m (multiplet) and br (broad). Reaction endpoints were determined by GC monitoring of the reactions. Gas chromatography was performed with machines of Agilent Technologies 7890, using a column of type HP 5 (Agilent 5% phenylmethylpolysiloxane; length: 15 m; diameter: 0.25 mm; film thickness: 0.25 μm) or Hewlett-Packard 6890 or 5890 series II, using a column of type HP 5 (HewlettPackard, 5% phenylmethylpolysiloxane; length: 15 m; diameter: 0,25 mm; film thickness: 0.25 μm). High resolution mass spectra (HRMS) and low resolution mass spectra (LRMS) were recorded on Finnigan MAT 95Q or Finnigan MAT 90 instrument or JEOL JMS-700. Infrared spectra were recorded on a Perkin 281 IR spectrometer and samples were measured neat (ATR, Smiths Detection DuraSample IR II Diamond ATR). The absorption bands were reported in wave numbers (cm-1) and abbreviations for intensity are as follows: vs (very strong; maximum intensity), s (strong; above 75% of max. intensity), m (medium; from 50% to 75% of max. intensity), w (weak; below 50% of max. intensity) and br (broad). Melting points were determined on a Büchi B-540 apparatus and uncorrected. *n*-BuLi, *s*-BuLi was purchased as solutions in cyclohexane/hexanes mixtures from Rockwood Lithium GmbH. The concentration of organometallic reagent from commercially purchased and synthesized reagents was determined either by titration of isopropyl alcohol using the indicator indicator 1,10-phenanthrolinein in THF or by using iodide dissolved in THF. Single crystals were grown in small quench vials with a volume of 5.0 ml from slow evaporation of dichloromethane/hexanes mixtures at room temperature. Suitable single crystals were then introduced into perfluorinated oil and mounted on top of a thin glass wire. Data collection was performed at 100 K with a Bruker D8 Venture TXS equipped with a Spellman generator (50 kV, 40 mA) and a Kappa CCD detector operating with Mo-Kα radiation (l = 0.71071 Å). The stereodynamics of thiete dioxides was investigated on an Agilent 1200 Infinity HPLC with an Agilent 6120 quadrupole mass spectrometer equipped with an APCI source. Separations were performed on Chiralpak IG-3 (150 mm, i.d. 4.6 mm, particle size 3 μm), Chiralpak IA, Chiralpak IB, Chiralpak IC, Chiralpak IE and Chiralpak IF (250 mm, i.d. 4.6 mm, particle size 5 μm respectively)

columns, which were purchased from Chiral Technologies and a (R,R)-Whelk-O1 column (250 mm, i.d. 4.6 mm, particle size 5 µm), which was purchased from Regis Technologies. HPLC grade solvents were obtained from Sigma-Aldrich.

For the low temperature measurements, the chiral columns were placed in a cooling bath (dry ice/acetone) and connected to the HPLC system by an 80 cm stainless steel capillary that was used as a mobile phase pre-cooler. Due to back pressure limitations the 2-propanol in the mobile phase was replaced by DCM and the detection wavelength was adjusted from 220 nm to 250 nm. In all cases, separation of enantiomers could not be observed at temperatures down to -65°C. A selection of representative chromatograms of the attempted separations is shown in chapter 4 of the Supporting Information

2. Experimental Procedures

2.1 General Procedures

2.1.1 General procedure A: Synthesis of *2H*-thiete 1,1- dioxides

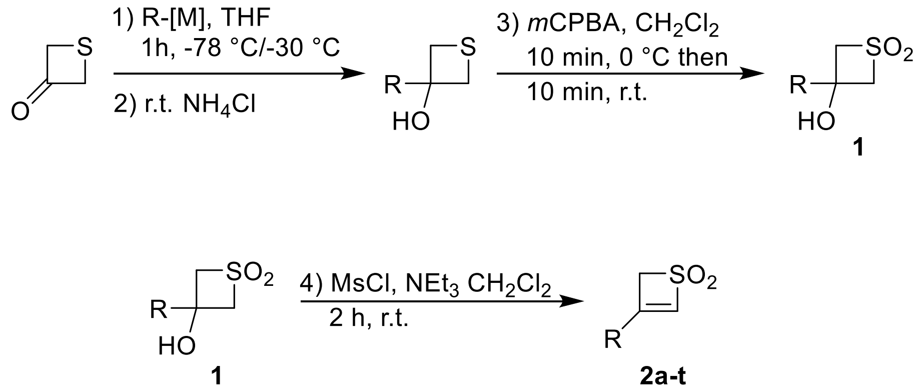

A flask was charged with thietan-3-one (10.0 mmol, 1.0 equiv.) and THF (20 mL) was added. The reaction mixture was cooled to -78 °C and a solution of organolithium reagent (1.30 equiv.) was added dropwise. Alternatively, the reaction mixture was cooled to -30 °C and a solution of organomagnesium reagent (1.30 equiv.) was added dropwise. After stirring for 60 min the mixture was brought to ambient temperature and quenched with a solution of saturated aqueous NH_4Cl. The aqueous phase was extracted with dichloromethane (3 × 50 mL) and washed with a solution of saturated aqueous NaCl (1 × 50 mL). The combined organic phases were dried over magnesium sulfate and concentrated in vacuo. The residue, containing the thietanol, was dissolved in dichloromethane (50 mL), cooled to 0 °C and *m*-CPBA (20.0 mmol, 2.0 equiv., 77%) was added portion wise. After TLC showed full conversion of the thietanol (approx. 10 min) water was added. The aqueous phase was extracted with dichloromethane (3 × 50 mL) and washed with a solution of saturated aqueous NaCl (1 × 50 mL). The combined organic phases were dried over magnesium sulfate, filtered and concentrated in vacuo. The residue, containing the thietanol dioxide **1**, was dissolved in dichloromethane (50 mL) and triethylamine (30 mmol, 3.0 equiv.) was added. Mesylchlorid (30 mmol, 3.0 equiv.) was subsequently added dropwise and the mixture was stirred until TLC indicated full conversion of the starting thietanol dioxide **1** (approx. 30 min) water was added. The aqueous phase was extracted with dichloromethane (3 × 50 mL) and washed with a solution of saturated aqueous NaCl (1 × 50 mL). The combined organic phases were dried over magnesium sulfate, filtered and concentrated in vacuo. The crude thiete dioxides **2a-t** were purified by flash column chromatography with appropriate solvent mixtures.

2.1.2 General procedure B: C-H Functionalization of *2H*-thiete 1,1- dioxides

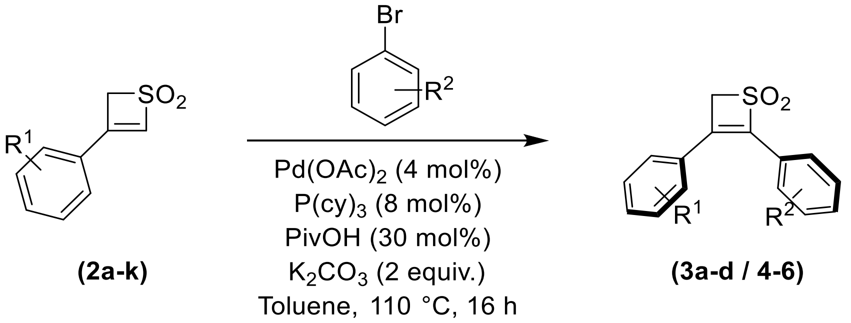

(2a-k) **(3a-d / 4-6)**

A pressure tube was charged with 2*H*-thiete 1,1- dioxides derivative **2a-k** (0.2 mmol, 1 equiv.) and 2 mL toluene was added. Subsequently were added K_2CO_3 (55 mg, 0.4 mmol, 2.0 equiv.), $Pd(OAc)_2$ (1.8 mg, 8 µmol, 4 mol%), tricyclohexylphosphane (PCy_3) (4.5 mg, 16 µmol, 8 mol%), the corresponding halogenide (0.3 mmol, 1.5 equiv.) and a few drops of pivalic acid (~7 µL, 30 mol%). The mixture was stirred at 110 °C in the sealed pressure tube until TLC showed consumption of the starting 2*H*-thiete 1,1- dioxides (approx. 16 hours). After cooling to ambient temperature, the tube was opened and a 1:1 mixture of H_2O:Et_2O (4 mL) was added. The aqueous phase was extracted with Et_2O (3 x 20 mL). The combined organic phases were dried over magnesium sulfate, filtrated, concentrated in vacuo and purified by flash-column chromatography on silica gel with the appropriate solvent mixture to obtain pure **3a-d**.

2.1.2 General procedure C: C-H Macrocyclization of *2H*-thiete 1,1- dioxides

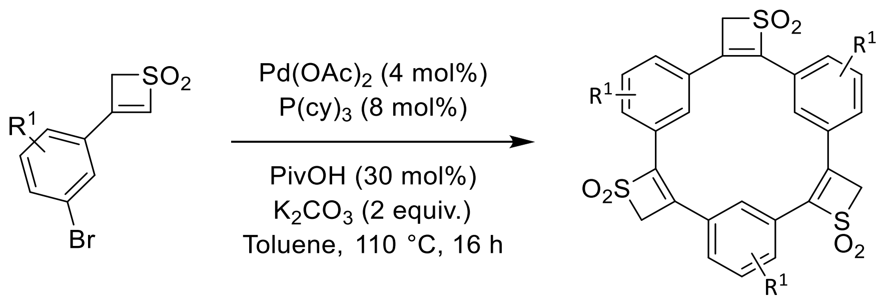

(2l-t) **(7a-h)**

A pressure tube was charged with 2*H*-thiete 1,1- dioxides derivative **2l-t** (0.2 mmol, 1 equiv.) and 2 mL toluene was added. Subsequently were added K_2CO_3 (55 mg, 0.4 mmol, 2.0 equiv.), $Pd(OAc)_2$ (1.8 mg, 8 µmol, 4 mol%), tricyclohexylphosphane (PCy_3) (4.5 mg, 16 µmol, 8 mol%) and a few drops of pivalic acid (~7 µL, 30 mol%). The mixture was stirred at 110 °C in the sealed pressure tube until TLC showed consumption of the starting 2*H*-thiete 1,1- dioxides (approx. 16 hours). After cooling to ambient temperature, the tube was opened and a 1:1 mixture of H_2O:CH_2Cl_2 (4 mL) was added. The aqueous phase was extracted with CH_2Cl_2 (3 x 20 mL). The combined organic phases were dried over magnesium sulfate, filtrated, concentrated in vacuo and purified by flash-column chromatography on silica gel with the appropriate solvent mixture to obtain pure **7a-h**.

2.2 Experimental Data

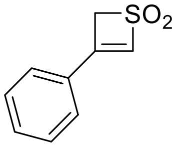

3-Phenyl-*2H*-thiete 1,1-dioxide (2a)

Using phenylmagnesium chloride according to general procedure **A**, provided **2a** (1.4 g, 7.8 mmol, 78%) as a colorless solid. **^{1}H NMR** (400 MHz, $CDCl_3$) δ 7.60-7.38 (m, 6H), 6.95 (s, 1H), 4.80 ppm (s, 2H). **^{13}C NMR** (101 MHz, $CDCl_3$) δ 147.4, 136.8, 132.4, 129.3, 128.9, 127.6, 70.0 ppm. Analytical data was in agreement with the literature[1].

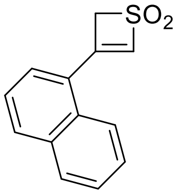

3-(Naphthalen-1-yl)-*2H*-thiete 1,1-dioxide (2b)

Using naphthalen-1-ylmagnesium bromide according to general procedure **A**, provided **2b** (1.10 g, 7.0 mmol, 70%) as a colorless solid. **^{1}H NMR** (400 MHz, $CDCl_3$) δ 8.17 (d, *J* = 8.4 Hz, 1H), 8.01 (d, *J* = 7.8 Hz, 1H), 7.96 (d, *J* = 8.0 Hz, 1H), 7.67 (t, *J* = 7.6 Hz, 1H), 7.61 (t, *J* = 7.4 Hz, 1H), 7.53 (t, *J* = 7.5 Hz, 1H), 7.49 (d, *J* = 6.5 Hz, 1H), 7.16 (s, 1H), 5.04 ppm (s, 2H). **^{13}C NMR** (101 MHz, $CDCl_3$) δ 146.2, 139.9, 134.0, 133.2, 130.8, 129.6, 128.6, 128.4, 127.0, 126.5, 125.1, 124.1, 72.8 ppm. Analytical data was in agreement with the literature[2].

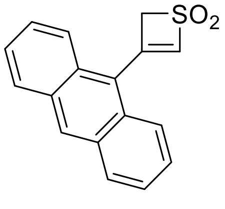

3-(Anthracen-9-yl)-2*H*-thiete 1,1-dioxide (2c)

Using anthracen-9-yllithium according to general procedure **A**, provided **2c** (0.70 g, 2.5 mmol, 25%) as yellow solid. ***R_f*** = 0.4 (hexane/EtOAc 8:2, UV, $KMnO_4$, PAA). **^{1}H NMR** (400 MHz, $CDCl_3$) δ 8.55 (s, 1H), 8.07 (d, *J* = 8.3 Hz, 2H), 8.01 (d, *J* = 8.5 Hz, 2H), 7.58 (dddd, *J* = 21.3, 7.9, 6.6, 1.3 Hz, 4H), 7.09 (s, 1H), 5.04 ppm (s, 2H).**^{13}C NMR** (101 MHz, $CDCl_3$) δ 149.0, 147.6, 131.0, 129.5, 129.2, 128.2, 127.6, 125.9, 124.3, 123.8, 75.4 ppm. **LRMS** (DEP/EI-Orbitrap): *m/z* (%): 280.0 (10), 215.1 (100), 202.1 (20). **HRMS** (EI-Orbitrap): m/z: [M]+ Calcd for $C_{17}H_{12}O_2{}^{32}S^+$: 280.0558; found: 280.0547. **IR** (Diamond-ATR, neat) $\tilde{\nu}_{max}$: 1474 (w), 1301 (vs), 1261 (w), 1232 (m), 1204 (s), 1184 (s), 1174 (m), 1165 (s), 1148 (s),

---

[1] J. A. Burkhard, Dissertation: https://doi.org/10.3929/ethz-a-006834147.
[2] M. Eisold; A. Mueller-Deku; F. Reiners; D. Didier, *Org. Lett.* **2018**, *20*, 4654.

1132 (m), 1120 (s), 1092 (m), 1077 (m), 1044 (w), 1014 (w), 958 (w), 944 (w), 912 (m), 890 (w), 848 (m), 812 (w), 783 (s), 762 (m), 750 (m), 732 cm^{-1} (vs). **Melting point**: 200 (±2) °C decomposition.

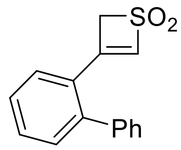

3-([1,1'-Biphenyl]-2-yl)-*2H*-thiete 1,1-dioxide (2d)

Using [1,1'-biphenyl]-2-ylmagnesium bromide according to general procedure **A**, provided **2d** (1.23 g, 4.8 mmol, 48%) as a yellowish solid. $\boldsymbol{R_f}$ = 0.5 (hexane/EtOAc 7:3, UV, $KMnO_4$, PAA). **^{1}H NMR** (400 MHz, CDCl3) δ 7.53 (td, *J* = 7.5, 1.5 Hz, 1H), 7.48-7.43 (m, 4H), 7.39 (dd, *J* = 7.8, 1.4 Hz, 1H), 7.34 (dd, *J* = 7.5, 1.4 Hz, 1H), 7.29-7.24 (m, 2H), 5.87 (s, 1H), 4.46 ppm (s, 2H). **^{13}C NMR** (101 MHz, $CDCl_3$) δ 146.9, 143.5, 140.2, 139.7, 131.7, 131.2, 129.3, 129.1, 128.7, 128.6, 128.1, 127.8, 71.5 ppm. Analytical data was in agreement with the literature[3].

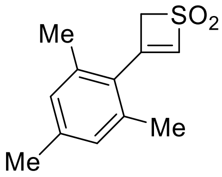

3-Mesityl-2*H*-thiete 1,1-dioxide (2e)

Using mesitylmagnesium bromide according to general procedure **A**, provided 2e (0.82 g, 3.7 mmol, 37%) as a colorless solid. $\boldsymbol{R_f}$ = 0.5 (hexane/EtOAc 8:2, UV, $KMnO_4$, PAA). **^{1}H NMR** (400 MHz, $CDCl_3$) δ 6.93 (s, 2H), 6.68 (s, 1H), 4.75 (s, 2H), 2.35-2.23 ppm (m, 9H). **^{13}C NMR** (101 MHz, $CDCl_3$) δ 150.0, 144.7, 139.9, 135.4, 131.7, 129.1, 73.6, 21.2, 20.5 ppm. **HRMS** (EI-Orbitrap): m/z: [M]+ Calcd for $C_{12}H_{14}O_2{}^{32}S^+$: 222.0715; found: 222.0710. **IR** (Diamond-ATR, neat) $\tilde{\nu}_{max}$: 1607 (w), 1448 (w), 1288 (s), 1276 (m), 1201 (vs), 1171 (m), 1150 (m), 1115 (vs), 1033 (m), 868 (m), 805 (m), 795 cm^{-1} (s). **Melting point**: 156 (±2) °C.

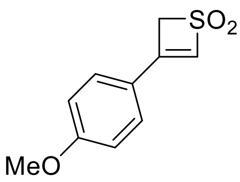

3-(4-Methoxyphenyl)-*2H*-thiete 1,1-dioxide (2f)

Using (4-methoxyphenyl)magnesium bromide according to general procedure **A**, provided **2f** (1.26 g, 6.0 mmol, 60%) as a colorless solid. **^{1}H NMR** (400 MHz, $CDCl_3$) δ 7.56-7.33 (m, 2H), 7.05-6.89 (m, 2H),

[3] A. N. Baumann; F. Reiners; T. Juli; D. Didier, *Org. Lett.* **2018**, *20*, 6736.

6.79 (s, 1H), 4.76 (s, 2H), 3.87 ppm (s, 3H). **^{13}C NMR** (101 MHz, $CDCl_3$) δ 162.9, 146.8, 134.0, 129.6, 121.6, 114.7, 70.0, 55.7 ppm. Analytical data was in agreement with the literature.[2]

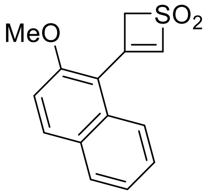

3-(2-Methoxynaphthalen-1-yl)-2*H*-thiete 1,1-dioxide (2g)

Using (2-methoxynaphthalen-1-yl)magnesium bromide according to general procedure **A**, provided **2g** (1.59 g, 6.1 mmol, 61%) as a colorless solid. $\boldsymbol{R}_f$ = 0.2 (hexane/EtOAc 7:3, UV, $KMnO_4$, PAA). **^{1}H NMR** (400 MHz, $CDCl_3$) δ 8.07-8.02 (m, 1H), 7.96 (d, *J* = 9.2 Hz, 1H), 7.86-7.81 (m, 1H), 7.59-7.52 (m, 1H), 7.46 – 7.39 (m, 1H), 7.29 (d, *J* = 9.1 Hz, 1H), 7.00 (s, 1H), 5.08 (s, 2H), 4.00 ppm (s, 3H). **^{13}C NMR** (101 MHz, $CDCl_3$) δ 156.2, 145.1, 143.9, 133.4, 131.5, 129.0, 129.0, 128.5, 124.5, 123.3, 112.4, 112.2, 74.5, 56.4 ppm. **HRMS** (EI-Orbitrap): m/z: [M]+ Calcd for $C_{14}H_{12}O_3{}^{32}S^+$: 260.0507; found: 260.0501. **IR** (Diamond-ATR, neat) $\tilde{\nu}_{max}$: 1286 (vs), 1277 (s), 1255 (m), 1193 (vs), 1167 (s), 1156 (m), 1134 (s), 1116 (s), 1094 (s), 1065 (s), 1056 (s), 1027 (m), 820 (s), 790 (s), 750 (s), 668 cm^{-1} (s). **Melting point**: 135 (±2) °C.

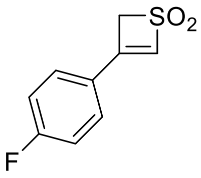

3-(4-Fluorophenyl)-*2H*-thiete 1,1-dioxide (2h)

Using (4-fluorophenyl)magnesium bromide according to general procedure **A**, provided **2h** (1.19 g, 6.0 mmol, 60%) as a colorless solid. **^{1}H NMR** (400 MHz, $CDCl_3$) δ 7.57-7.39 (m, 2H), 7.21-7.10 (m, 2H), 6.90 (s, 1H), 4.78 ppm (s, 2H). **^{13}C NMR** (101 MHz, $CDCl_3$) δ 165.0 (d, *J* = 255.1 Hz), 146.2, 136.5 (d, *J* = 2.3 Hz), 130.0 (d, *J* = 9.1 Hz), 125.4 (d, *J* = 3.4 Hz), 116.7 (d, *J* = 22.3 Hz), 70.1 ppm. Analytical data was in agreement with the literature.[2]

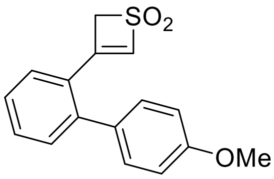

3-(4'-Methoxy-[1,1'-biphenyl]-2-yl)-*2H*-thiete 1,1-dioxide (2i)

Using (4'-methoxy-[1,1'-biphenyl]-2-yl)lithium according to general procedure **A**, provided **2i** (1.37 g, 4.8 mmol, 48%) as a yellowish solid. $\boldsymbol{R}_f$ = 0.25 (hexane/EtOAc 8:2, UV, $KMnO_4$, PAA). **^{1}H NMR** (400 MHz, $CDCl_3$) δ 7.52 (td, *J* = 7.4, 1.6 Hz, 1H), 7.46-7.37 (m, 2H), 7.34 (dd, *J* = 7.6, 1.3 Hz, 1H), 7.22-7.17 (m, 2H), 7.01-6.95 (m, 2H), 5.98 (s, 1H), 4.47 (s, 2H), 3.87 ppm (s, 3H). **^{13}C NMR** (101 MHz,

$CDCl_3$) δ 159.9, 147.2, 143.2, 139.6, 132.4, 131.7, 131.5, 129.9, 129.3, 128.0, 127.9, 114.4, 71.5, 55.5 ppm. Analytical data was in agreement with the literature.[3]

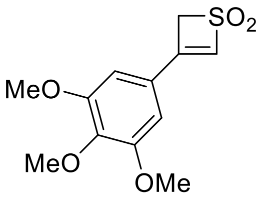

3-(3,4,5-Trimethoxyphenyl)-*2H*-thiete 1,1-dioxide (2j)

Using (3,4,5-trimethoxyphenyl)magnesium bromide according to general procedure **A**, provided **2j** (1.84 g, 6.8 mmol, 68%) as a colorless solid. **^{1}H NMR** (400 MHz, $CDCl_3$) δ 6.87 (s, 1H), 6.63 (s, 2H), 4.77 (s, 2H), 3.91 (s, 3H), 3.89 ppm (s, 6H). **^{13}C NMR** (101 MHz, $CDCl_3$) δ 153.7, 147.3, 141.9, 135.9, 124.3, 105.0, 70.0, 61.2, 56.5 ppm. Analytical data was in agreement with the literature[2].

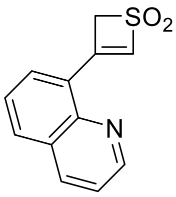

3-(Quinolin-8-yl)-*2H*-thiete 1,1-dioxide (2k)

Using quinolin-8-yllithium according to a modified general procedure **A** (see literature: A. N. Baumann; F. Reiners; T. Juli; D. Didier, *Org. Lett.* **2018**, *20*, 6736), provided **2k** (0.55 g, 2.4 mmol, 24%) as an orange solid. ***R*$_f$** = 0.15 (hexane/EtOAc 7:3, UV, $KMnO_4$, PAA). **^{1}H NMR** (400 MHz, $CDCl_3$) δ 8.99 (dd, *J* = 4.2, 1.8 Hz, 1H), 8.23 (dd, *J* = 8.3, 1.8 Hz, 1H), 7.99 (dd, *J* = 8.2, 1.4 Hz, 1H), 7.89 (s, 1H), 7.71 (dd, *J* = 7.3, 1.4 Hz, 1H), 7.60 (t, *J* = 7.7 Hz, 1H), 7.53 (dd, *J* = 8.3, 4.2 Hz, 1H), 5.06 ppm (s, 2H). **^{13}C NMR** (101 MHz, $CDCl_3$) δ 151.2, 146.9, 144.2, 143.2, 136.7, 132.8, 131.1, 128.7, 127.5, 126.0, 122.2, 72.0 ppm. Analytical data was in agreement with the literature[3].

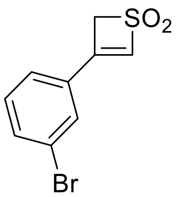

3-(3-Bromophenyl)-*2H*-thiete 1,1-dioxide (2l)

Using (3-bromophenyl)magnesium iodide according to general procedure **A**, provided **2l** (0.78 g, 3.0 mmol, 30%) as a colorless solid. ***R*$_f$** = 0.4 (hexane/EtOAc 7:3, UV, $KMnO_4$, PAA). **^{1}H NMR** (400 MHz, $CDCl_3$) δ 7.66 (dt, *J* = 7.4, 1.8 Hz, 1H), 7.60-7.58 (m, 1H), 7.42-7.34 (m, 2H), 6.99 (s, 1H), 4.79 ppm (s, 2H). **^{13}C NMR** (101 MHz, $CDCl_3$) δ 146.0, 138.4, 135.2, 130.9, 130.9, 130.4, 126.1, 123.5, 70.1 ppm. Analytical data was in agreement with the literature[3].

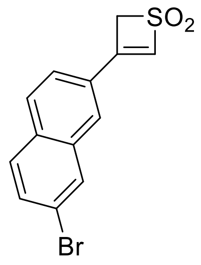

3-(7-Bromonaphthalen-2-yl)-2*H*-thiete 1,1-dioxide (2m)

Using (7-bromonaphthalen-2-yl)lithium according to general procedure **A**, provided **2m** (0.68 g, 2.2 mmol, 22%) as a colorless solid. $\boldsymbol{R_f}$ = 0.3 (CH_2Cl_2, UV, $KMnO_4$, PAA). **^{1}H NMR** (400 MHz, $CDCl_3$) δ 8.07 (s, 1H), 7.90 (d, *J* = 8.7 Hz, 1H), 7.80-7.74 (m, 2H), 7.68 (dd, *J* = 8.7, 1.9 Hz, 1H), 7.59 (dd, *J* = 8.6, 1.8 Hz, 1H), 7.08 (s, 1H), 4.91 ppm (s, 2H). **^{13}C NMR** (101 MHz, $CDCl_3$) δ 146.8, 137.9, 133.9, 133.1, 132.1, 130.9, 129.7, 129.4, 127.5, 127.4, 123.7, 121.7, 70.2 ppm. **HRMS** (EI-Orbitrap): m/z: [M]+ Calcd for $C_{13}H_9O_2BrS^+$: 307.9507; found: 307.9500. **IR** (Diamond-ATR, neat) $\tilde{\nu}_{max}$: 3008 (w), 1592 (w), 1578 (m), 1496 (w), 1312 (m), 1288 (vs), 1230 (w), 1202 (s), 1170 (s), 1152 (m), 1140 (s), 1121 (s), 1111 (s), 1076 (m), 1062 (s), 1002 (m), 973 (m), 962 (w), 948 (m), 927 (m), 914 (m), 902 (s), 870 (w), 849 (s), 810 (s), 786 (s), 776 (vs), 758 cm^{-1} (s). **Melting point**: 248 (±2) °C.

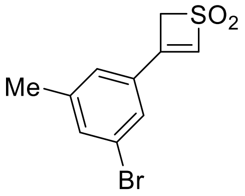

3-(3-Bromo-5-methylphenyl)-2H-thiete 1,1-dioxide (2n)

Using (3-bromo-5-methylphenyl)lithium according to general procedure **A**, provided **2n** (6.7 mmol, 1.9 g, 67%) as a colorless solid. $\boldsymbol{R_f}$ = 0.2 (hexane/EtOAc 9:1, UV, $KMnO_4$). **^{1}H NMR** (400 MHz, $CDCl_3$): *δ* 7.48 (s, 1H), 7.39 (s, 1H), 7.18 (s, 1H), 6.96 (s, 1H), 4.77 (s, 2H), 2.39 ppm (s, 3H). **^{13}C NMR** (101 MHz, $CDCl_3$): *δ* 146.1, 141.4, 138.0, 135.9, 130.6, 127.6, 126.8, 123.2, 70.1, 21.2 ppm. **LRMS** (DEP/EI-Orbitrap): *m/z* (%): 273.9 (17), 256.9 (1), 244.9 (1), 224.9 (5), 208.0 (14), 128.1 (100). **HRMS** (EI-Orbitrap): m/z: [M]+ Calcd for $C_{10}H_9O_2BrS^+$: 271.9507; found: 271.9501. **IR** (Diamond-ATR, neat) $\tilde{\nu}_{max}$:: 3094 (vw), 1558 (w), 1432 (w), 1424 (w), 1399 (w), 1348 (m), 1289 (vs), 1211 (s), 1174 (vs), 1125 (vs), 1007 (m), 973 (m), 915 (s), 854 (s), 770 (vs), 744 (m), 670 cm^{-1} (m). **Melting Point**: 97 (±2) °C.

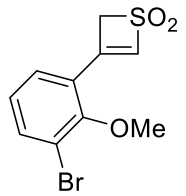

3-(3-Bromo-2-methoxyphenyl)-2*H*-thiete 1,1-dioxide (2o)

Using (3-bromo-2-methoxyphenyl)lithium according to general procedure **A**, provided **2o** (6.7 mmol, 1.9 g, 67%) as colorless solid. R_f = 0.2 (hexane/EtOAc 8:2, UV, $KMnO_4$). **^{1}H NMR** (400 MHz, $CDCl_3$): δ 7.72 (dd, *J* = 8.0, 1.6 Hz, 1H), 7.22 (dd, *J* = 7.7, 1.6 Hz, 1H), 7.14 (s, 1H), 7.10 (t, *J* = 7.9 Hz, 1H), 4.84 (s, 2H), 3.90 ppm (s, 3H). **^{13}C NMR** (101 MHz, $CDCl_3$): δ 157.3, 143.0, 141.5, 137.9, 129.1, 125.9, 124.75, 118.8, 71.5, 60.8 ppm. **LRMS** (DEP/EI-Orbitrap): *m/z* (%): 290.0 (20), 240.0 (10), 225.0 (20), 209.0 (100). **HRMS** (EI-Orbitrap): m/z: [M]+ Calcd for $C_{10}H_9O_3BrS^+$: 287.9456; found: 287.9450. **IR** (Diamond-ATR, neat) $\tilde{\nu}_{max}$: 3075 (w), 2970 (w), 2943 (w), 1594 (m), 1572 (w), 1559 (w), 1470 (s), 1449 (m), 1421 (s), 1349 (m), 1288 (vs), 1260 (m), 1243 (m), 1224 (s), 1192 (s), 1177 (s), 1156 (m), 1131 (vs), 1107 (s), 1082 (m), 1062 (m), 1007 (m), 1000 (m), 982 (s), 921 (s), 819 (m), 800 (s), 781 (vs), 756 (m), 741 (m), 724 (s), 692 (m), 685 cm^{-1} (m). **Melting Point**: 135 (±2) °C.

.

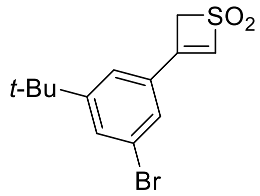

3-(3-Bromo-5-(*tert*-butyl)phenyl)-2*H*-thiete 1,1-dioxide (2p)

Using (3-bromo-5-(tert-butyl)phenyl)lithium according to general procedure **A**, provided **2p** (4.44 mmol, 1.4 g, 44%) as colorless solid. R_f = 0.2 (hexane/EtOAc 9:1, UV, $KMnO_4$). **^{1}H NMR** (400 MHz, $CDCl_3$): δ 7.66 (t, *J* = 1.7 Hz, 1H), 7.40 (t, *J* = 1.7 Hz, 1H), 7.35 (t, *J* = 1.6 Hz, 1H), 6.98 (s, 1H), 4.79 (s, 2H), 1.33 ppm (s, 9H). **^{13}C NMR** (101 MHz, $CDCl_3$): δ 154.8, 146.5, 137.9, 132.7, 130.6, 127.6, 123.4, 123.1, 70.1, 35.2, 31.2 ppm. **LRMS** (DEP/EI-Orbitrap): *m/z* (%): 316.0 (50), 301.0 (75), 265.0 (10), 251.0 (80), 235.0 (100). **HRMS** (EI-Orbitrap): m/z: [M]+ Calcd for $C_{13}H_{15}O_2BrS^+$: 313.9976; found: 313.9972. **IR** (Diamond-ATR, neat) $\tilde{\nu}_{max}$: 2962 (w), 1591 (w), 1553 (m), 1430 (w), 1404 (w), 1370 (w), 1362 (w), 1292 (vs), 1269 (m), 1242 (w), 1205 (s), 1170 (w), 1125 (s), 1094 (w), 995 (w), 929 (m), 887 (w), 860 (m), 800 (m), 782 (s), 758 (m), 738 (m), 713 (w), 690 cm^{-1} (m). **Melting Point**: 181 (±2) °C.

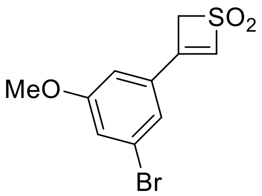

3-(3-Bromo-5-methoxyphenyl)-2*H*-thiete 1,1-dioxide (2q)

Using (3-bromo-5-methoxyphenyl)lithium according to general procedure **A**, provided **2q** (1.21 g, 4.2 mmol, 42%) as a colorless solid. $\boldsymbol{R_f}$ = 0.3 (hexane/EtOAc 8:2, UV, $KMnO_4$, PAA). **^{1}H NMR** (400 MHz, $CDCl_3$) δ 7.18-7.13 (m, 2H), 6.96 (s, 0H), 6.90-6.80 (m, 1H), 4.75 (s, 2H), 3.83 ppm (s, 3H). **^{13}C NMR** (101 MHz, $CDCl_3$) δ 160.7, 146.0, 138.5, 131.4, 123.8, 122.7, 120.5, 112.3, 70.1, 56.0 ppm. **HRMS** (EI-Orbitrap): m/z: [M]+ Calcd for $C_{10}H_9O_3BrS$: 287.9456; found: 287.9450. **IR** (Diamond-ATR, neat) $\tilde{\nu}_{max}$: 1693 (w), 1588 (m), 1576 (m), 1559 (s), 1453 (m), 1418 (m), 1316 (m), 1289 (vs), 1264 (m), 1236 (m), 1192 (vs), 1168 (s), 1136 (s), 1123 (s), 1088 (w), 1057 (m), 1002 (m), 990 (m), 930 (m), 898 (w), 869 (m), 854 (s), 844 (s), 790 (s), 780 (s), 774 (s), 742 cm^{-1} (s). **Melting point**: 156 (±2) °C.

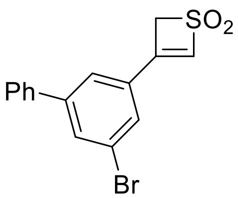

3-(5-Bromo-[1,1'-biphenyl]-3-yl)-2*H*-thiete 1,1-dioxide (2r)

Using (5-bromo-[1,1'-biphenyl]-3-yl)lithium according to general procedure **A**, provided **2r** (5.37 mmol, 1.8 g, 36%) as a colorless solid. $\boldsymbol{R_f}$ = 0.2 (hexane/EtOAc 9:1, UV, $KMnO_4$). **^{1}H NMR** (400 MHz, $CDCl_3$): *δ* 7.86 (s, 1H), 7.63-7.36 (m, 7H), 7.04 (s, 1H), 4.83 ppm (s, 2H). **^{13}C NMR** (101 MHz, $CDCl_3$): *δ* 146.0, 144.6, 138.6, 138.4, 133.8, 131.2, 129.3, 128.9, 128.9, 127.3, 124.9, 123.8, 70.2 ppm. **LRMS** (DEP/EI-Orbitrap): *m/z* (%): 333.9 (33), 288.0 (10), 272.0 (10), 258 (10), 189.1 (100). **HRMS** (EI-Orbitrap): m/z: [M]+ Calcd for $C_{15}H_{11}O_2BrS^+$: 333.9663; found: 333.9658. **IR** (Diamond-ATR, neat) $\tilde{\nu}_{max}$: 1572 (m), 1565 (m), 1501 (w), 1441 (w), 1410 (w), 1396 (w), 1307 (w), 1290 (vs), 1247 (w), 1203 (vs), 1141 (vs), 1122 (vs), 1075 (m), 1035 (w), 994 (w), 924 (w), 887 (w), 864 (m), 809 (m), 787 (s), 760 (vs), 740 (s), 694 (s), 678 cm^{-1} (m). **Melting Point**: 156 (±2) °C.

.

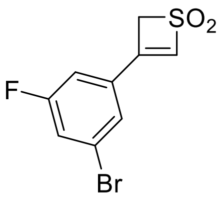

3-(3-Bromo-5-fluorophenyl)-2*H*-thiete 1,1-dioxide (2s)

Using (3-bromo-5-fluorophenyl)lithium according to general procedure **A**, provided **2s** (0.58 g, 2.1 mmol, 21%) as a colorless solid. $\boldsymbol{R_f}$ = 0.4 (hexane/EtOAc 8:2, UV, $KMnO_4$, PAA). **^{1}H NMR** (400 MHz, $CDCl_3$) δ 7.45-7.35 (m, 2H), 7.12 (dd, *J* = 8.4, 1.9 Hz, 1H), 7.03 (s, 1H), 4.78 ppm (s, 2H). **^{13}C NMR** (101 MHz,

$CDCl_3$) δ 162.8 (d, *J* = 254.2 Hz), 144.9 (d, *J* = 2.9 Hz), 139.7, 132.2 (d, *J* = 8.5 Hz), 126.6 (d, *J* = 3.4 Hz), 123.9 (d, *J* = 9.7 Hz), 122.8 (d, *J* = 24.2 Hz), 113.4 (d, *J* = 22.5 Hz), 70.1 ppm. **HRMS** (EI-Orbitrap): m/z: [M]+ Calcd for $C_9H_6O_2BrFS^+$: 275.9256; found: 275.9252. **IR** (Diamond-ATR, neat) $\tilde{\nu}_{max}$: 1587 (m), 1570 (m), 1428 (m), 1306 (s), 1217 (m), 1183 (vs), 1142 (m), 1127 (s), 876 (m), 794 cm^{-1} (m). **Melting point**: 204 (±2) °C.

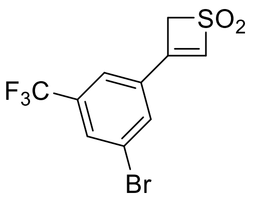

3-(3-Bromo-5-(trifluoromethyl)phenyl)-2*H*-thiete 1,1-dioxide (2t)

Using (3-bromo-5-(trifluoromethyl)phenyl)lithium according to general procedure **A**, provided **2t** (4.28 mmol, 1.4 g, 43%) as a colorless solid. ***R*f** = 0.1 (hexane/EtOAc 9:1, UV, $KMnO_4$). **^{1}H NMR** (400 MHz, $CDCl_3$): δ 7.91 (s, 1H), 7.78 (s, 1H), 7.61 (s, 1H), 7.11 (s, 1H), 4.84 ppm (s, 2H). **^{13}C NMR** (101 MHz, $CDCl_3$): δ 144.6, 140.4, 133.6, 131.8 (q, *J* = 3.8 Hz), 131.7, 124.1, 123.91, 122.9 (d, *J* = 3.8 Hz), 121.2, 70.2 ppm. **LRMS** (DEP/EI-Orbitrap): *m/z* (%): 327.9 (11), 308.9 (4), 263.9 (11), 249.9 (27), 183.0 (100). **HRMS** (EI-Orbitrap): m/z: [M]+ Calcd for $C_{10}H_6O_2BrF_3S^+$: 325.9225; found: 325.9217. **IR** (Diamond-ATR, neat) $\tilde{\nu}_{max}$: 3080 (w), 2361 (w), 1570 (w), 1454 (w), 1429 (w), 1406 cm^{-1} (w). **Melting point**: 176 (±2) °C.

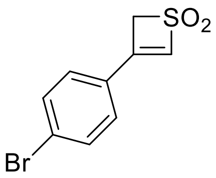

3-(4-Bromophenyl)-2*H*-thiete 1,1-dioxide (2u)

Using (4-bromophenyl)magnesium bromide according to general procedure **A**, provided **2u** (5.80 mmol, 1.5 g, 58%) as white solid. ***R*f** = 0.2 (hexane/EtOAc 9:1, UV, $KMnO_4$). **^{1}H NMR** (400 MHz, $CDCl_3$): *δ* 7.65-7.60 (m, 2H), 7.36-7.30 (m, 2H), 6.98 (s, 1H), 4.80 ppm (s, 2H). **^{13}C NMR** (101 MHz, $CDCl_3$): *δ* 146.3, 137.6, 132.7, 129.0, 127.9, 127.2, 70.1 ppm. **HRMS** (EI-Orbitrap): m/z: [M]+ Calcd for $C_9H_7O_2BrS^+$: 257.9350; found: 257.9355. **Melting Point**: 188 (±2) °C.

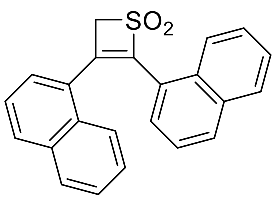

3,4-Di(naphthalen-1-yl)-2*H*-thiete 1,1-dioxide (3a)

Using 1-bromonaphthalene according to general procedure **B**, provided **3a** (0.50 g, 1.4 mmol, 70%) as a brownish solid. ***R*f** = 0.4 (hexane/EtOAc 8:2, UV, $KMnO_4$, PAA). **^{1}H NMR** (400 MHz, $CDCl_3$) δ 8.12 (dd, *J* = 8.5, 1.1 Hz, 1H), 7.91-7.74 (m, 5H), 7.59 (dd, *J* = 7.2, 1.2 Hz, 1H), 7.52 (dd, *J* = 7.2, 1.3 Hz, 1H), 7.47-7.30 (m, 5H), 7.21 (ddd, *J* = 8.4, 6.9, 1.3 Hz, 1H), 5.16 ppm (s, 2H). **^{13}C NMR** (101 MHz, $CDCl_3$) δ 153.7, 141.2, 133.7, 133.6, 131.5, 131.1, 130.8, 129.7, 128.9, 128.6, 128.5, 128.3, 127.4, 127.3, 127.2, 126.7, 126.6, 125.5, 125.3, 125.3, 125.2, 124.7, 72.3 ppm. **LRMS** (DEP/EI-Orbitrap): *m/z* (%): 356.2 (10), 339.1 (2), 308.2 (60), 291.1 (100), 276.1 (95). **HRMS** (EI-Orbitrap): m/z: [M]+ Calcd for $C_{23}H_{16}O_2S^+$: 356.0871; found: 356.0863. **IR** (Diamond-ATR, neat) $\tilde{\nu}_{max}$: 2364 (m), 2358 (m), 2344 (w), 1312 (w), 1296 (m), 1287 (s), 1276 (m), 1260 (m), 1251 (m), 1235 (m), 1218 (m), 1204 (m), 1183 (s), 1138 (s), 1114 (m), 1087 (m), 1057 (w), 981 (m), 803 cm^{-1} (m). **Melting point**: 170 (±2) °C.

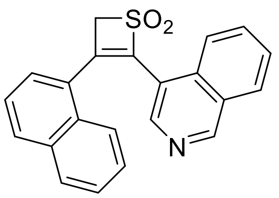

4-(Isoquinolin-4-yl)-3-(naphthalen-1-yl)-2*H*-thiete 1,1-dioxide (3b)

Using 4-bromoisoquinoline according to general procedure **B**, provided **3b** (50 mg, 0.14 mmol, 70%) as a colorless solid. ***R*f** = 0.2 (hexane/EtOAc 7:3, UV, $KMnO_4$, PAA). **^{1}H NMR** (400 MHz, $CDCl_3$) δ 9.17 (s, 1H), 8.43 (s, 1H), 8.24 (d, *J* = 8.4 Hz, 1H), 7.96 (d, *J* = 8.1 Hz, 1H), 7.91 (d, *J* = 8.2 Hz, 1H), 7.82 (d, *J* = 8.2 Hz, 1H), 7.70 (t, *J* = 8.3 Hz, 2H), 7.62 (t, *J* = 7.5 Hz, 1H), 7.54 (d, *J* = 7.1 Hz, 1H), 7.49-7.44 (m, 1H), 7.40 (t, *J* = 7.3 Hz, 1H), 7.21 (t, *J* = 7.7 Hz, 1H), 5.18 ppm (s, 2H). **^{13}C NMR** (101 MHz, $CDCl_3$) δ 154.7, 151.3, 144.4, 143.0, 133.8, 133.3, 132.1, 131.7, 129.5, 129.1, 128.4, 128.3, 128.3, 127.6, 127.4, 127.2, 126.8, 125.3, 124.7, 124.5, 119.9, 72.6 ppm. **LRMS** (DEP/EI-Orbitrap): *m/z* (%): 357.1 (40), 309.1 (90), 292.1 (100), 266.1 (30). **HRMS** (EI-Orbitrap): m/z: [M]+ Calcd for $C_{22}H_{15}NO_2S^+$: 357.0823; found: 357.0816. **Melting point**: 165 (±2) °C.

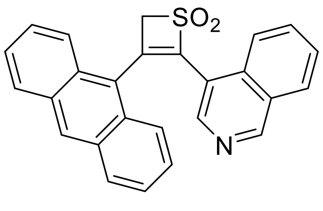

3-(Anthracen-9-yl)-4-(isoquinolin-4-yl)-2*H*-thiete 1,1-dioxide (3c)

Using 4-bromoisoquinoline according to general procedure **B**, provided **3c** (35 mg, 0.09 mmol, 43%) as a colorless solid. ***R*f** = 0.2 (hexane/EtOAc 7:3, UV, $KMnO_4$, PAA). **^{1}H NMR** (400 MHz, $CDCl_3$) δ 8.57 – 8.47 (m, 2H), 8.14 (d, *J* = 8.5 Hz, 2H), 8.03 (dd, *J* = 8.6, 1.4 Hz, 2H), 7.93 (d, *J* = 8.6 Hz, 1H), 7.68 (d, *J* = 8.3 Hz, 1H), 7.60 – 7.41 (m, 6H), 7.07 (ddd, *J* = 8.4, 6.9, 1.3 Hz, 1H), 5.19 ppm (s, 2H). **^{13}C NMR** (101 MHz, $CDCl_3$) δ 157.4, 147.1, 143.4, 143.1, 136.4, 131.2, 130.5, 129.6, 129.2, 128.4, 128.1, 127.7, 127.3, 126.7, 125.9, 125.1, 124.7, 124.7, 122.4, 74.0 ppm. **HRMS** (EI-Orbitrap): m/z: [M]+ Calcd for $C_{26}H_{17}NO_2S^+$: 407.0980; found: 407.0973. **Melting point**: 180 (±2) °C.

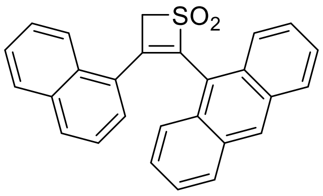

4-(Anthracen-9-yl)-3-(naphthalen-1-yl)-2*H*-thiete 1,1-dioxide (3d)

Using 9-bromoanthracene according to general procedure **B**, provided **3d** (50 mg, 0.12 mmol, 62%) as a brownish solid. $\boldsymbol{R_f}$ = 0.5 (hexane/EtOAc 7:3, UV, $KMnO_4$, PAA). **^{1}H NMR** (400 MHz, $CDCl_3$) δ 8.49 (s, 1H), 8.39 (d, *J* = 8.8 Hz, 2H), 7.98 (d, *J* = 8.4 Hz, 3H), 7.74 (t, *J* = 8.3 Hz, 2H), 7.52 (ddd, *J* = 8.6, 6.6, 1.4 Hz, 2H), 7.48 – 7.43 (m, 3H), 7.41 – 7.35 (m, 1H), 7.29 – 7.23 (m, 2H), 5.36 ppm(s, 2H). **^{13}C NMR** (101 MHz, $CDCl_3$) δ 153.5, 145.8, 133.7, 131.4, 131.2, 131.0, 130.4, 130.1, 129.0, 128.9, 127.9, 127.6, 127.2, 127.1, 126.6, 125.8, 125.6, 125.2, 124.4, 120.8, 72.3 ppm. **LRMS** (DEP/EI-Orbitrap): *m/z* (%): 406.2 (15), 341.2 (100), 326.1 (20). **HRMS** (EI-Orbitrap): m/z: [M]+ Calcd for $C_{27}H_{18}O_2S^+$: 406.1028; found: 406.1023. **IR** (Diamond-ATR, neat) $\tilde{\nu}_{max}$: 1300 (s), 1205 (m), 1180 (m), 1160 (m), 1134 (vs), 1114 (m), 898 (m), 804 (m), 781 (s), 733 cm^{-1} (vs). **Melting point**: 185 (±2) °C.

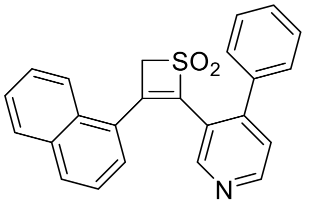

3-(Naphthalen-1-yl)-4-(4-phenylpyridin-3-yl)-2*H*-thiete 1,1-dioxide (4a)

Using 4-(2-bromophenyl)pyridine according to general procedure **B**, provided **4a** (73 mg, 0.19 mmol, 95%) as a colorless solid. $\boldsymbol{R_f}$ = 0.2 (hexane/EtOAc 7:3, UV, $KMnO_4$, PAA). **^{1}H NMR** (400 MHz, $CDCl_3$) δ 9.14 (s, 1H), 8.61 (d, *J* = 5.1 Hz, 1H), 7.75 (t, *J* = 8.2 Hz, 2H), 7.42 (ddd, *J* = 8.1, 6.8, 1.2 Hz, 1H), 7.32 (d, *J* = 8.0 Hz, 1H), 7.20 (ddd, *J* = 8.3, 6.8, 1.3 Hz, 1H), 7.17-7.13 (m, 1H), 7.00 (d, *J* = 5.1 Hz, 1H), 6.95-6.86 (m, 3H), 6.80 (dd, *J* = 7.2, 1.2 Hz, 1H), 6.67-6.63 (m, 2H), 4.92 ppm (s, 2H). **^{13}C NMR** (101 MHz, $CDCl_3$) δ 151.2, 150.1, 149.7, 149.0, 142.6, 136.9, 133.4, 131.3, 129.2, 128.7, 128.2, 128.1, 127.8, 127.7, 126.9, 126.5, 126.4, 124.7, 124.6, 124.6, 122.8, 72.3 ppm. **LRMS** (DEP/EI-Orbitrap): *m/z* (%): 383.1 (10), 354.9 (1), 335.1 (25), 318.1 (30), 304.1 (50). **HRMS** (EI-Orbitrap): m/z: [M]+ Calcd for $C_{24}H_{17}NO_2S^+$: 383.0980; found: 383.0974. **IR** (Diamond-ATR, neat) $\tilde{\nu}_{max}$: 1708 (vs), 1435 (w), 1418 (m), 1360 (s), 1320 (m), 1221 (s), 1132 (w), 1093 (w), 777 cm^{-1} (m). **Melting point**: 215 (±2) °C.

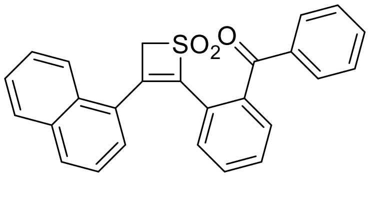

(2-(3-(Naphthalen-1-yl)-1,1-dioxido-2*H*-thiet-4-yl)phenyl)(phenyl)methanone (4b)

Using (2-bromophenyl)(phenyl)methanone according to general procedure **B**, provided **4b** (70 mg, 0.17 mmol, 85%) as a white solid. ***R*f** = 0.2 (hexane/EtOAc 8:2, UV, $KMnO_4$, PAA). **^{1}H NMR** (400 MHz, $CDCl_3$) δ 7.92 (dd, *J* = 7.7, 1.1 Hz, 1H), 7.67 (dd, *J* = 8.3, 1.2 Hz, 1H), 7.63-7.53 (m, 3H), 7.46-7.34 (m, 3H), 7.29 (ddd, *J* = 8.3, 6.8, 1.4 Hz, 1H), 7.25-7.17 (m, 3H), 7.10 (t, *J* = 7.8 Hz, 2H), 7.04 (dd, *J* = 8.3, 1.5 Hz, 2H), 4.87 (s, 2H). **^{13}C NMR** (101 MHz, $CDCl_3$) δ 194.4, 151.0, 141.3, 139.0, 135.0, 133.6, 132.9, 131.3, 131.2, 129.9, 129.8, 129.6, 129.3, 129.3, 128.9, 127.9, 127.5, 127.2, 127.1, 126.5, 126.5, 125.0, 124.8, 72.5 ppm. **LRMS** (DEP/EI-Orbitrap): *m/z* (%): 346.1 (30), 331.1 (15), 239.1 (60). **HRMS** (EI-Orbitrap): m/z: [M-SO_2]+ Calcd for $C_{26}H_{18}O^+$: 346.1358; found: 346.1359. **IR** (Diamond-ATR, neat) $\tilde{\nu}_{max}$: 1656 (s), 1299 (s), 1286 (m), 1266 (m), 1256 (m), 1184 (m), 1135 (s), 1114 (m), 927 (m), 799 (m), 777 (s), 761 (m), 704 cm^{-1} (vs). **Melting point**: 181 (±2) °C.

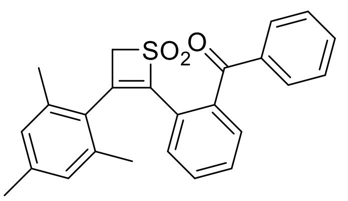

(2-(3-Mesityl-1,1-dioxido-2*H*-thiet-4-yl)phenyl)(phenyl)methanone (4c)

Using (2-bromophenyl)(phenyl)methanone according to general procedure **B**, provided **4c** (57 mg, 0.14 mmol, 71%) as a colorless solid. ***R*f** = 0.4 (hexane/EtOAc 8:2, UV, $KMnO_4$, PAA). **^{1}H NMR** (400 MHz, $CDCl_3$) δ 7.96 (d, *J* = 7.2 Hz, 1H), 7.63 (td, *J* = 7.6, 1.3 Hz, 1H), 7.54-7.40 (m, 4H), 7.33-7.24 (m, 3H), 6.43 (s, 2H), 4.74 (s, 2H), 2.00 ppm (s, 9H). **^{13}C NMR** (101 MHz, $CDCl_3$) δ 193.7, 153.3, 142.8, 139.6, 138.7, 135.8, 134.9, 132.7, 130.9, 130.1, 129.4, 129.2, 129.0, 128.8, 127.9, 127.0, 125.6, 72.2, 21.0, 20.1 ppm. **LRMS** (DEP/EI-Orbitrap): *m/z* (%): 338.1 (70), 323.1 (40), 305.1 (20). **HRMS** (EI-Orbitrap): m/z: [M-SO_2]+ Calcd for $C_{24}H_{19}O_2^+$: 338.1671; found: 338.1680. **IR** (Diamond-ATR, neat) $\tilde{\nu}_{max}$: 1654 (m), 1447 (w), 1440 (w), 1314 (w), 1303 (vs), 1287 (m), 1269 (m), 1203 (m), 1184 (s), 1166 (w), 1156 (w), 1128 (s), 926 (m), 846 (m), 830 (w), 804 (w), 760 (s), 701 cm^{-1} (vs). **Melting point**: 162 (±2) °C.

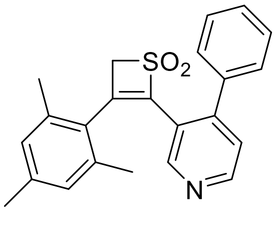

3-Mesityl-4-(4-phenylpyridin-3-yl)-2*H*-thiete 1,1-dioxide (4d)

Using 4-(2-bromophenyl)pyridine according to general procedure **B**, provided **4d** (71 mg, 0.19 mmol, 95%) as a colorless solid. ***R*f** = 0.25 (hexane/EtOAc 7:3, UV, $KMnO_4$, PAA). **^{1}H NMR** (400 MHz, $CDCl_3$) δ 8.91 (s, 1H), 8.60 (d, *J* = 5.1 Hz, 1H), 7.36-7.22 (m, 3H), 7.13 (d, *J* = 5.1 Hz, 1H), 7.10-7.07 (m, 2H), 6.61 (s, 2H), 4.66 (s, 2H), 2.19 (s, 3H), 1.75 ppm (s, 6H). **^{13}C NMR** (101 MHz, $CDCl_3$) δ 152.3, 150.7, 149.9, 149.7, 145.1, 139.6, 137.6, 135.6, 128.8, 128.7, 128.6, 128.1, 125.8, 125.1, 123.2, 72.3, 21.1, 20.3 ppm. **LRMS** (DEP/EI-Orbitrap): *m/z* (%): 375.1 (10), 327.1 (5), 311.1 (10), 296.1 (100). **HRMS** (EI-

Orbitrap): m/z: [M]+ Calcd for $C_{23}H_{21}NO_2S^+$: 375.1293; found: 375.1294. **IR** (Diamond-ATR, neat) $\tilde{\nu}_{max}$: 1580 (m), 1478 (w), 1396 (m), 1303 (vs), 1289 (m), 1262 (m), 1244 (m), 1193 (s), 1177 (s), 1156 (m), 1128 (s), 1065 (w), 1036 (w), 982 (w), 848 (s), 825 (w), 780 (m), 764 (m), 749 (m), 734 (m), 706 cm^{-1} (s). **Melting point**: 196 (±2) °C.

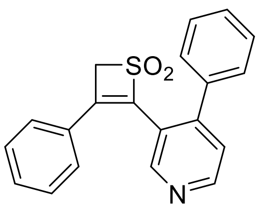

3-Phenyl-4-(4-phenylpyridin-3-yl)-2*H*-thiete 1,1-dioxide (4e)

Using 4-(2-bromophenyl)pyridine according to general procedure **B**, provided **4e** (41 mg, 0.12 mmol, 62%) as a colorless solid. ***R*f** = 0.2 (hexane/EtOAc 7:3, UV, $KMnO_4$, PAA). **^{1}H NMR** (400 MHz, $CDCl_3$) δ 8.90 (s, 1H), 8.77 (d, *J* = 5.1 Hz, 1H), 7.50-7.30 (m, 4H), 7.26 (s, 5H), 7.01 (d, *J* = 7.6 Hz, 2H), 4.74 ppm (s, 2H). **^{13}C NMR** (101 MHz, $CDCl_3$) δ 151.5, 150.5, 150.1, 146.4, 141.9, 137.1, 131.8, 129.2, 129.0, 128.8, 128.6, 128.5, 128.2, 124.7, 122.3, 69.5 ppm. **LRMS** (DEP/EI-Orbitrap): *m/z* (%): 333.1 (2), 268.1 (100), 191.1 (30). **HRMS** (EI-Orbitrap): m/z: [M-HSO_2]+ Calcd for $C_{20}H_{14}N^+$: 268.1126; found: 268.1129. **IR** (Diamond-ATR, neat) $\tilde{\nu}_{max}$: 2970 (w), 2909 (w), 1582 (w), 1576 (w), 1476 (w), 1447 (w), 1405 (w), 1334 (w), 1293 (s), 1278 (m), 1262 (w), 1203 (m), 1189 (w), 1180 (w), 1132 (vs), 1104 (w), 1076 (m), 1064 (w), 1041 (w), 1022 (w), 988 (w), 958 (w), 910 (w), 851 (m), 836 (w), 780 (m), 761 cm^{-1} (s). **Melting point**: 216 (±2) °C.

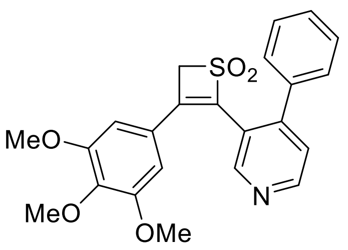

4-(4-Phenylpyridin-3-yl)-3-(3,4,5-trimethoxyphenyl)-2*H*-thiete 1,1-dioxide (4f)

Using 4-(2-bromophenyl)pyridine according to general procedure **B**, provided **4f** (35 mg, 0.08 mmol, 41%) as a colorless solid. ***R*f** = 0.05 (hexane/EtOAc 7:3, UV, $KMnO_4$, PAA). **^{1}H NMR** (400 MHz, $CDCl_3$) δ 8.89 (s, 1H), 8.75 (d, *J* = 5.1 Hz, 1H), 7.45-7.37 (m, 3H), 7.34-7.27 (m, 3H), 6.17 (s, 2H), 4.71 (s, 2H), 3.82 (s, 3H), 3.62 ppm (s, 6H). **^{13}C NMR** (101 MHz, $CDCl_3$) δ 153.2, 151.5, 150.7, 150.1, 145.8, 142.0, 141.3, 137.0, 129.1, 128.7, 128.5, 124.5, 124.5, 122.3, 105.6, 69.5, 61.1, 56.2 ppm. **LRMS** (DEP/EI-Orbitrap): *m/z* (%): 423.1 (30), 375.1 (10), 359.1 (20), 328.1 (40). **HRMS** (EI-Orbitrap): m/z: [M]+ Calcd for $C_{23}H_{21}NO_5S^+$: 423.1140; found: 423.1144. **IR** (Diamond-ATR, neat) $\tilde{\nu}_{max}$: 1579 (m), 1508 (m), 1499 (w), 1452 (m), 1414 (m), 1400 (w), 1358 (m), 1296 (s), 1241 (m), 1194 (m), 1126 (vs), 1104 (m), 1079 (m), 1004 (m), 963 (w), 947 (w), 922 (m), 914 (m), 858 (m), 829 (m), 798 (w), 782 (m), 760 (m), 743 cm^{-1} (s). **Melting point**: 208 (±2) °C.

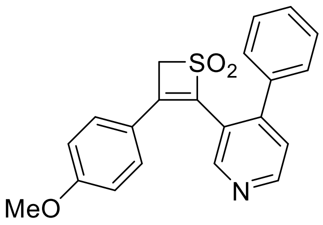

3-(4-Methoxyphenyl)-4-(4-phenylpyridin-3-yl)-2*H*-thiete 1,1-dioxide (4g)

Using 4-(2-bromophenyl)pyridine according to general procedure **B**, provided **4g** (30 mg, 0.08 mmol, 41%) as a colorless solid. R_f = 0.1 (hexane/EtOAc 7:3, UV, $KMnO_4$, PAA). **^{1}H NMR** (400 MHz, $CDCl_3$) δ 8.88 (s, 1H), 8.76 (d, *J* = 5.2 Hz, 1H), 7.50-7.38 (m, 3H), 7.32-7.23 (m, 3H), 6.98 (d, *J* = 8.9 Hz, 2H), 6.71 (d, *J* = 8.8 Hz, 2H), 4.69 (s, 2H), 3.78 ppm (s, 3H). **^{13}C NMR** (101 MHz, $CDCl_3$) δ 162.4, 151.3, 150.7, 150.2, 143.5, 141.3, 137.2, 130.2, 129.1, 128.6, 128.6, 124.7, 122.6, 121.8, 114.3, 69.3, 55.6 ppm. **LRMS** (DEP/EI-Orbitrap): *m/z* (%): 363.1 (5),315.1 (5), 298.1 (10), 268.1 (20). **HRMS** (EI-Orbitrap): m/z: [M]+ Calcd for $C_{21}H_{17}NO_3S^+$: 363.0929; found: 363.0919. **IR** (Diamond-ATR, neat) $\tilde{\nu}_{max}$: 2967 (w), 2909 (w), 1601 (s), 1581 (m), 1571 (m), 1516 (s), 1476 (w), 1463 (w), 1427 (m), 1397 (w), 1336 (m), 1313 (m), 1289 (s), 1267 (s), 1200 (m), 1178 (s), 1127 (vs), 1118 (vs), 1080 (m), 1073 (m), 1045 (m), 1018 (s), 988 (w), 982 (w), 958 (m), 926 (w), 853 (m), 838 (s), 814 (m), 790 (m), 778 (m), 762 (m), 746 cm^{-1} (s). **Melting point**: 223 (±2) °C.

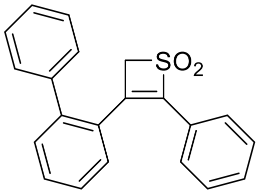

3-([1,1'-Biphenyl]-2-yl)-4-phenyl-2*H*-thiete 1,1-dioxide (5a)

Using bromobenzene according to general procedure **B**, provided **5a** (62 mg, 0.19 mmol, 93%) as a colorless solid. R_f = 0.5 (hexane/EtOAc 8:2, UV, $KMnO_4$, PAA). **^{1}H NMR** (400 MHz, $CDCl_3$) δ 7.57-7.48 (m, 5H), 7.44-7.33 (m, 9H), 4.06 ppm (s, 2H). **^{13}C NMR** (101 MHz, $CDCl_3$) δ 152.6, 141.8, 139.8, 139.2, 130.8, 130.7, 130.4, 129.7, 129.2, 129.0, 129.0, 128.7, 128.5, 127.9, 127.2, 126.9, 71.7 ppm. **LRMS** (DEP/EI-Orbitrap): *m/z* (%): 267.1 (100), 252.1 (30), 239.0 (10). **HRMS** (EI-Orbitrap): m/z: [M]+ Calcd for $C_{21}H_{16}O_2S^+$: 332.0871; found: 332.0859. **IR** (Diamond-ATR, neat) $\tilde{\nu}_{max}$: 3059 (w), 3027 (w), 1964 (w), 1953 (w), 1711 (m), 1595 (w), 1493 (w), 1473 (m), 1448 (m), 1435 (w), 1362 (w), 1327 (m), 1304 (vs), 1221 (w), 1194 (s), 1176 (vs), 1130 (vs), 1076 (w), 1031 (w), 1025 (w), 1009 (w), 963 (w), 917 (w), 850 (w), 761 cm^{-1} (vs). **Melting point**: 146 (±2) °C.

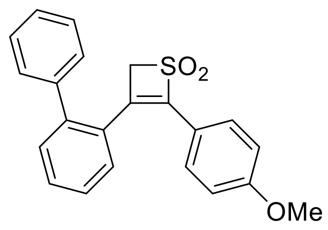

3-([1,1'-Biphenyl]-2-yl)-4-(4-methoxyphenyl)-2*H*-thiete 1,1-dioxide (5b)

Using 1-bromo-4-methoxybenzene according to general procedure **B**, provided **5b** (50 mg, 0.14 mmol, 69%) as a colorless solid. $\boldsymbol{R_f}$ = 0.2 (hexane/EtOAc 9:1, UV, $KMnO_4$, PAA). **^{1}H NMR** (400 MHz, $CDCl_3$) δ 7.61-7.31 (m, 11H), 6.87 (d, *J* = 8.9 Hz, 2H), 4.02 (s, 2H), 3.83 ppm (s, 3H). **^{13}C NMR** (101 MHz, $CDCl_3$) δ 161.2, 152.4, 141.8, 139.8, 136.2, 130.7, 130.4, 130.0, 129.0, 129.0, 128.8, 128.7, 128.5, 127.9, 119.4, 114.7, 71.5, 55.5 ppm. **LRMS** (DEP/EI-Orbitrap): *m/z* (%): 362.2 (5), 298.2 (40), 267.1 (40), 239.1 (20). **HRMS** (EI-Orbitrap): m/z: [M]+ Calcd for $C_{22}H_{18}O_3S^+$: 362.0977; found: 362.0971. **IR** (Diamond-ATR, neat) $\tilde{\nu}_{max}$: 1603 (m), 1511 (m), 1474 (w), 1454 (w), 1420 (w), 1298 (s), 1256 (s), 1185 (vs), 1176 (s), 1127 (s), 1075 (w), 1064 (w), 1048 (w), 1025 (s), 1007 (m), 998 (w), 985 (w), 961 (w), 925 (w), 856 (w), 831 (s), 815 (w), 786 (w), 765 (vs), 743 (s), 726 (m), 716 (m), 704 cm^{-1} (vs). **Melting point**: 152 (±2) °C.

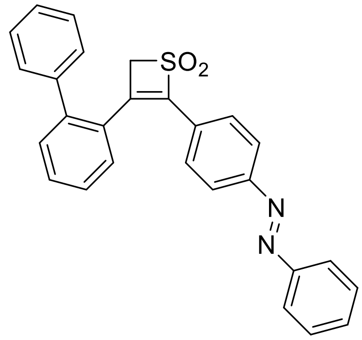

(*E*)-3-([1,1'-Biphenyl]-2-yl)-4-(4-(phenyldiazenyl)phenyl)-2*H*-thiete 1,1-dioxide (5c)

Using (*E*)-1-(4-iodophenyl)-2-phenyldiazene according to general procedure **B**, provided **5c** (35 mg, 0.08 mmol, 40%) as a red solid. $\boldsymbol{R_f}$ = 0.5 (hexane/EtOAc 8:2, UV, $KMnO_4$, PAA). **^{1}H NMR** (400 MHz, $CDCl_3$) δ 7.98-7.87 (m, 4H), 7.67 (d, *J* = 8.5 Hz, 2H), 7.62-7.49 (m, 6H), 7.48-7.33 (m, 6H), 4.12 ppm (s, 2H). **^{13}C NMR** (101 MHz, $CDCl_3$) δ 153.2, 152.6, 152.0, 142.0, 140.5, 139.7, 131.7, 130.9, 130.9, 129.5, 129.3, 129.1, 129.0, 129.0, 128.8, 128.6, 128.1, 128.0, 123.7, 123.2, 71.9 ppm. **LRMS** (DEP/EI-Orbitrap): *m/z* (%): 436.1 (20), 388.1 (5), 371.1 (10), 252.1 (50). **HRMS** (EI-Orbitrap): m/z: [M]+ Calcd for $C_{27}H_{20}N_2O_2S^+$: 436.1245; found: 436.1240. **IR** (Diamond-ATR, neat) $\tilde{\nu}_{max}$: 1481 (w), 1432 (w), 1408 (w), 1324 (w), 1301 (vs), 1278 (w), 1262 (w), 1221 (w), 1191 (m), 1172 (s), 1164 (m), 1132 (s), 1123 (m), 1106 (w), 1071 (w), 1041 (w), 1027 (w), 1020 (w), 1012 (w), 1006 (w), 1000 (w), 961 (w), 916 (w), 859 (m), 835 (m), 765 (vs), 750 (m), 740 cm^{-1} (s). **Melting point**: 160 (±2) °C.

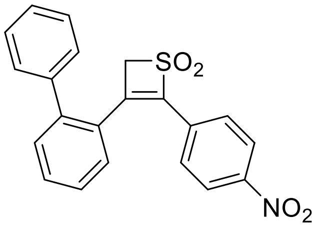

3-([1,1'-Biphenyl]-2-yl)-4-(4-nitrophenyl)-2*H*-thiete 1,1-dioxide (5d)

Using 1-bromo-4-nitrobenzene according to general procedure **B**, provided **5d** (40 mg, 0.11 mmol, 53%) as a colorless solid. ***R*$_f$** = 0.5 (hexane/EtOAc 8:2, UV, $KMnO_4$, PAA). **^{1}H NMR** (400 MHz, $CDCl_3$) δ 8.19 (d, *J* = 8.8 Hz, 2H), 7.64-7.57 (m, 3H), 7.54-7.44 (m, 3H), 7.43-7.35 (m, 3H), 7.32-7.27 (m, 2H), 4.23 ppm (s, 2H). **^{13}C NMR** (101 MHz, $CDCl_3$) δ 150.2, 148.3, 143.5, 142.1, 139.4, 132.8, 131.5, 131.2, 129.1, 128.8, 128.8, 128.7, 128.2, 128.1, 124.4, 72.4 ppm. **LRMS** (DEP/EI-Orbitrap): *m/z* (%): 358.2 (10), 313.2 (50), 296.1 (5), 283.1 (10), 266.1 (100). **HRMS** (EI-Orbitrap): m/z: [M]+ Calcd for $C_{21}H_{15}NO_4S^+$: 377.0722; found: 377.0713. **IR** (Diamond-ATR, neat) $\tilde{\nu}_{max}$: 3095 (w), 1569 (m), 1559 (m), 1432 (w), 1292 (vs), 1212 (s), 1186 (s), 1142 (m), 1127 (vs), 854 (m), 792 cm^{-1} (s). **Melting point**: 210 (±2) °C.

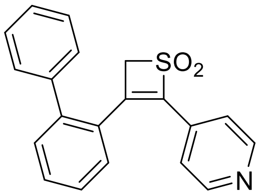

3-([1,1'-Biphenyl]-2-yl)-4-(pyridin-4-yl)-2*H*-thiete 1,1-dioxide (5e)

Using 4-bromopyridine according to general procedure **B**, provided **5e** (40 mg, 0.11 mmol, 61%) as a yellow oil. ***R*$_f$** = 0.1 (hexane/EtOAc 7:3, UV, $KMnO_4$, PAA). **^{1}H NMR** (400 MHz, $CDCl_3$) δ 8.61 (d, *J* = 5.9 Hz, 2H), 7.58 (td, *J* = 7.5, 1.5 Hz, 1H), 7.53-7.48 (m, 2H), 7.46-7.36 (m, 4H), 7.35-7.27 (m, 4H), 4.18 ppm (s, 2H). **^{13}C NMR** (101 MHz, $CDCl_3$) δ 150.6, 150.0, 144.4, 142.0, 139.4, 134.1, 131.4, 131.1, 129.1, 128.8, 128.8, 128.7, 128.1, 120.9, 72.4 ppm. **LRMS** (DEP/EI-Orbitrap): *m/z* (%): 268.1 (100), 239.1 (10), 226.1 (5). **HRMS** (EI-Orbitrap): m/z: [M]+ Calcd for $C_{20}H_{15}NO_2S^+$: 333.0823; found: 333.0827.

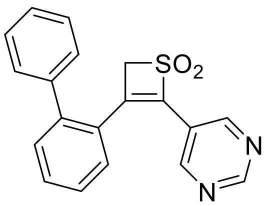

3-([1,1'-Biphenyl]-2-yl)-4-(pyrimidin-5-yl)-2*H*-thiete 1,1-dioxide (5f)

Using 5-bromopyrimidine according to general procedure **B**, provided **5f** (45 mg, 0.13 mmol, 67%) as a yellow oil. ***R*$_f$** = 0.4 (hexane/EtOAc 6:4, UV, $KMnO_4$, PAA). **^{1}H NMR** (400 MHz, $CDCl_3$) δ 9.13 (s, 1H), 8.62 (s, 2H), 7.62-7.51 (m, 1H), 7.52-7.41 (m, 3H), 7.37-7.30 (m, 3H), 7.25-7.20 (m, 2H), 4.46 ppm (s, 2H). **^{13}C NMR** (101 MHz, $CDCl_3$) δ 158.9, 154.6, 146.2, 143.5, 141.9, 139.2, 131.6, 131.3, 129.0, 128.8, 128.7, 128.5, 128.3, 128.2, 122.4, 72.7 ppm. **LRMS** (DEP/EI-Orbitrap): *m/z* (%): 334.2 (2), 269.2 (100), 215.1 (10). **HRMS** (EI-Orbitrap): m/z: [M]+ Calcd for $C_{19}H_{14}N_2O_2S^+$: 334.0776; found: 334.0774.

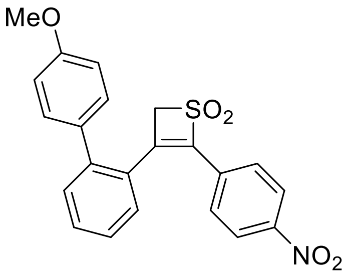

3-(4'-Methoxy-[1,1'-biphenyl]-2-yl)-4-(4-nitrophenyl)-2*H*-thiete 1,1-dioxide (5g)

Using 1-bromo-4-nitrobenzene according to general procedure **B**, provided **5g** (49 mg, 0.12 mmol, 60%) as a yellow oil. $\boldsymbol{R_f}$ = 0.5 (hexane/EtOAc 7:3, UV, $KMnO_4$, PAA). **^{1}H NMR** (400 MHz, $CDCl_3$) δ 8.19 (d, *J* = 8.8 Hz, 2H), 7.63 (d, *J* = 8.8 Hz, 2H), 7.56 (td, *J* = 7.5, 1.5 Hz, 1H), 7.51-7.45 (m, 2H), 7.40 (td, *J* = 7.5, 1.4 Hz, 1H), 7.22 (d, *J* = 8.7 Hz, 2H), 6.92 (d, *J* = 8.6 Hz, 2H), 4.27 (s, 2H), 3.82 ppm (s, 3H). **^{13}C NMR** (101 MHz, $CDCl_3$) δ 160.0, 150.0, 148.3, 143.7, 141.8, 132.9, 131.6, 131.5, 131.1, 130.0, 128.8, 128.6, 128.0, 127.7, 124.4, 114.5, 72.3, 55.5 ppm. **LRMS** (DEP/EI-Orbitrap): *m/z* (%): 355.1 (5), 343.1 (50), 326.9 (5), 313.1 (10). **HRMS** (EI-Orbitrap): m/z: [M]+ Calcd for $C_{22}H_{17}NO_5S^+$: 407.0827; found: 407.0821.

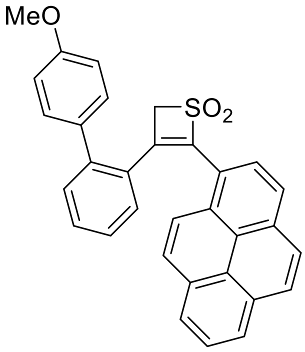

3-(4'-Methoxy-[1,1'-biphenyl]-2-yl)-4-(pyren-1-yl)-2*H*-thiete 1,1-dioxide (5h)

Using 1-bromopyrene according to general procedure **B**, provided **5h** (49 mg, 0.10 mmol, 50%) as a yellow oil. $\boldsymbol{R_f}$ = 0.5 (hexane/EtOAc 8:2, UV, $KMnO_4$, PAA). **^{1}H NMR** (400 MHz, $CDCl_3$) δ 8.23-8.15 (m, 3H), 8.11 (d, *J* = 8.9 Hz, 1H), 8.06-7.99 (m, 4H), 7.96 (d, *J* = 7.9 Hz, 1H), 7.37 (td, *J* = 7.5, 1.3 Hz, 1H), 7.32 (dd, *J* = 7.9, 1.2 Hz, 1H), 7.25 (d, *J* = 6.8 Hz, 1H), 7.15 (td, *J* = 7.6, 1.4 Hz, 1H), 6.99 (d, *J* = 8.6 Hz, 2H), 6.60 (d, *J* = 8.6 Hz, 2H), 4.56 (s, 2H), 3.54 ppm (s, 3H). **^{13}C NMR** (101 MHz, $CDCl_3$) δ 159.2, 153.0, 142.3, 141.9, 132.6, 132.2, 131.2, 131.1, 130.8, 130.7, 129.8, 129.5, 129.4, 129.3, 128.9, 128.7, 127.3, 127.2, 127.0, 126.4, 126.0, 126.0, 124.8, 124.7, 124.3, 122.1, 113.9, 71.0, 55.0 ppm. **LRMS** (DEP/EI-Orbitrap): *m/z* (%): 486.1 (5), 480.1 (60). **HRMS** (EI-Orbitrap): m/z: [M]+ Calcd for $C_{32}H_{22}O_3S^+$: 486.1290; found: 486.1291.

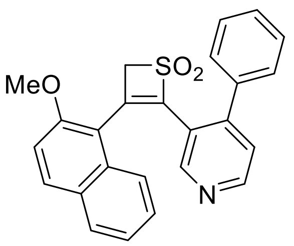

3-(2-Methoxynaphthalen-1-yl)-4-(4-phenylpyridin-3-yl)-2*H*-thiete 1,1-dioxide (6a)

Using 4-(2-bromophenyl)pyridine according to general procedure **B**, provided **6a** (52 mg, 0.13 mmol, 63%) as yellowish solid. R_f = 0.5 (hexane/EtOAc 6:4, UV, $KMnO_4$, PAA). **^{1}H NMR** (400 MHz, $CDCl_3$) δ 9.16 (s, 1H), 8.54 (d, *J* = 5.1 Hz, 1H), 7.71 (d, *J* = 9.1 Hz, 1H), 7.67 (d, *J* = 7.3 Hz, 1H), 7.23 (d, *J* = 7.6 Hz, 2H), 7.13 (ddd, *J* = 8.3, 6.7, 1.4 Hz, 1H), 7.09-7.03 (m, 1H), 6.97 (t, *J* = 7.6 Hz, 2H), 6.91 (d, *J* = 5.1 Hz, 1H), 6.85 (d, *J* = 9.1 Hz, 1H), 6.66-6.62 (m, 2H), 5.00 (s, 2H), 3.70 ppm (s, 3H). **^{13}C NMR** (101 MHz, $CDCl_3$) δ 155.1, 151.0, 150.7, 149.7, 149.3, 140.8, 137.0, 132.7, 130.2, 128.4, 128.3, 127.6, 127.5, 127.5, 127.5, 124.8, 124.0, 123.5, 123.5, 111.8, 111.6, 72.9, 55.5 ppm. **LRMS** (DEP/EI-Orbitrap): *m/z* (%): 413.1 (10), 349.1 (10), 318.1 (30). **HRMS** (EI-Orbitrap): m/z: [M]+ Calcd for $C_{25}H_{19}NO_3S^+$: 413.1086; found: 413.1087. **IR** (Diamond-ATR, neat) $\tilde{\nu}_{max}$: 1592 (m), 1580 (m), 1510 (m), 1467 (m), 1294 (s), 1267 (s), 1249 (m), 1223 (w), 1176 (m), 1165 (vs), 1136 (s), 1106 (m), 1079 (s), 1026 (m), 998 (w), 969 (m), 909 (w), 869 (m), 856 (m), 812 (s), 778 (s), 762 (s), 748 cm^{-1} (s). **Melting point**: 190 (±2) °C.

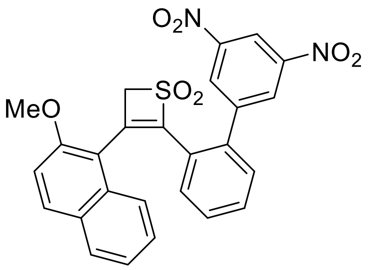

4-(3',5'-Dinitro-[1,1'-biphenyl]-2-yl)-3-(2-methoxynaphthalen-1-yl)-2*H*-thiete 1,1-dioxide (6b)

Using 2-bromo-3',5'-dinitro-1,1'-biphenyl according to general procedure **B**, provided **6b** (46 mg, 0.09 mmol, 46%) as yellowish solid. R_f = 0.7 (hexane/EtOAc 6:4, UV, $KMnO_4$, PAA). **^{1}H NMR** (400 MHz, $CDCl_3$) δ 8.53 (t, *J* = 2.1 Hz, 1H), 8.14 (dd, *J* = 7.8, 1.2 Hz, 1H), 7.65 (dtt, *J* = 7.7, 5.6, 3.1 Hz, 5H), 7.48 (td, *J* = 7.6, 1.3 Hz, 1H), 7.33-7.27 (m, 1H), 7.19-7.08 (m, 2H), 7.03 (dd, *J* = 7.7, 1.2 Hz, 1H), 6.86 (d, *J* = 9.1 Hz, 1H), 4.98 (s, 2H), 3.68 ppm (s, 3H). **^{13}C NMR** (101 MHz, $CDCl_3$) δ 155.1, 152.6, 147.9, 143.1, 138.2, 137.2, 133.0, 131.0, 130.3, 130.1, 130.1, 129.0, 128.9, 128.4, 128.2, 127.9, 127.1, 124.8, 123.3, 116.4, 112.1, 111.5, 72.8, 55.8 ppm. **LRMS** (DEP/EI-Orbitrap): *m/z* (%): 502.1 (100), 421.1 (95), 391.1 (30). **HRMS** (EI-Orbitrap): m/z: [M]+ Calcd for $C_{26}H_{18}N_2O_7S^+$: 502.0835; found: 502.0838. **IR** (Diamond-ATR, neat) $\tilde{\nu}_{max}$: 1548 (s), 1533 (m), 1510 (m), 1469 (w), 1360 (m), 1343 (s), 1309 (s), 1266 (s), 1246 (m), 1184 (m), 1174 (s), 1158 (m), 1138 (s), 1120 (m), 1104 (m), 1072 (s), 1048 (w), 1028 (m), 996 (w), 965 (w), 920 (w), 912 (m), 895 (w), 884 (w), 858 (w), 815 (s), 783 (m), 775 cm^{-1} (s). **Melting point**: 270 (±2) °C.

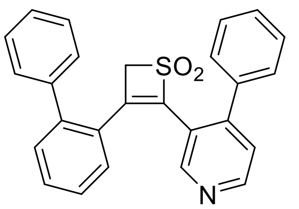

3-([1,1'-Biphenyl]-2-yl)-4-(4-phenylpyridin-3-yl)-2*H*-thiete 1,1-dioxide (6c)

Using 4-(2-bromophenyl)pyridine according to general procedure **B**, provided **6c** (72 mg, 0.18 mmol, 88%) as white solid. R_f = 0.2 (hexane/EtOAc 7:3, UV, $KMnO_4$, PAA). **^{1}H NMR** (400 MHz, $CDCl_3$) δ 8.58 (d, *J* = 5.1 Hz, 1H), 8.47 (s, 1H), 7.36-7.17 (m, 7H), 7.16-7.09 (m, 4H), 7.05 (td, *J* = 7.6, 1.3 Hz, 1H), 6.79-6.69 (m, 3H), 4.22 ppm (s, 2H). **^{13}C NMR** (101 MHz, $CDCl_3$) δ 150.6, 149.7, 149.6, 148.9, 143.7, 141.9, 139.5, 137.4, 130.4, 130.4, 128.6, 128.5, 128.5, 128.4, 128.3, 128.2, 127.4, 124.5, 122.3, 71.3 ppm. **LRMS** (DEP/EI-Orbitrap): *m/z* (%): 409.1 (5), 345.1 (50), 330.1 (20). **HRMS** (EI-Orbitrap): m/z: [M]+ Calcd for $C_{26}H_{19}NO_2S^+$: 409.1136; found: 409.1139. **IR** (Diamond-ATR, neat) $\tilde{\nu}_{max}$: 1709 (m), 1581 (m), 1481 (m), 1444 (w), 1436 (w), 1400 (w), 1362 (m), 1327 (w), 1301 (s), 1221 (m), 1192 (m), 1173 (s), 1130 (s), 1076 (w), 850 (m), 778 (m), 766 (m), 756 cm^{-1} (s). **Melting point**: 210 (±2) °C.

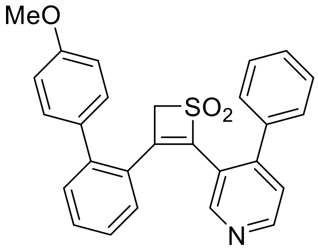

3-(4'-Methoxy-[1,1'-biphenyl]-2-yl)-4-(4-phenylpyridin-3-yl)-2*H*-thiete 1,1-dioxide (6d)

Using 4-(2-bromophenyl)pyridine according to general procedure **B**, provided **6d** (50 mg, 0.11 mmol, 57%) as white solid. R_f = 0.3 (hexane/EtOAc 6:4, UV, $KMnO_4$, PAA). **^{1}H NMR** (400 MHz, $CDCl_3$) δ 8.64-8.54 (m, 2H), 7.33-7.22 (m, 4H), 7.20-7.09 (m, 4H), 7.03 (td, *J* = 7.5, 1.3 Hz, 1H), 6.77 (td, *J* = 7.0, 1.7 Hz, 3H), 6.66 (d, *J* = 8.7 Hz, 2H), 4.19 (s, 2H), 3.84 ppm (s, 3H). **^{13}C NMR** (101 MHz, $CDCl_3$) δ 159.6, 150.7, 149.8, 149.7, 148.7, 143.9, 141.7, 137.5, 131.7, 130.5, 129.8, 128.7, 128.6, 128.5, 128.5, 128.4, 126.9, 124.6, 124.5, 122.4, 114.0, 71.3, 55.5 ppm. **LRMS** (DEP/EI-Orbitrap): *m/z* (%): 439.1 (80), 389.1 (5), 375.1 (85), 360.1 (30). **HRMS** (EI-Orbitrap): m/z: [M]+ Calcd for $C_{27}H_{21}NO_3S^+$: 439.1242; found: 439.1238. **IR** (Diamond-ATR, neat) $\tilde{\nu}_{max}$: 1711 (m), 1611 (m), 1517 (m), 1483 (m), 1442 (m), 1302 (vs), 1246 (s), 1222 (m), 1192 (s), 1176 (s), 1132 (vs), 1035 (m), 834 (m), 761 (m), 755 (m), 700 cm^{-1} (m). **Melting point**: 220 (±2) °C.

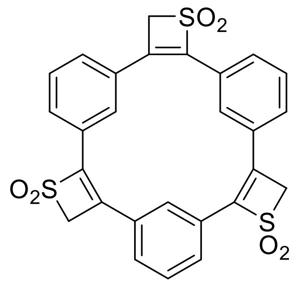

Cyclotris (3-phenylthiete 1,1-dioxide-3', 4-diyl) (7a)

Using 4-(3-bromophenyl)-2*H*-thiete 1,1-dioxide (**2l**) according to general procedure **C**, provided **7a** (40 mg, 0.07 mmol, 37%) as yellowish solid. ***R*f** = 0.2 (hexane/EtOAc 5:5, UV, $KMnO_4$). **^{1}H NMR** (400 MHz, $CDCl_3$) δ 7.82 (d, *J* = 7.9 Hz, 1H), 7.71-7.59 (m, 1H), 7.41 (d, *J* = 7.9 Hz, 1H), 4.86 ppm (s, 1H). **^{13}C NMR** (101 MHz, $CDCl_3$) δ 150.5, 139.7, 131.1, 131.0, 130.9, 129.9, 127.8, 126.6, 72.1 ppm. **HRMS** (ESI-Quadrupole): m/z: [M$^+$-H] Calcd for $C_{27}H_{17}O_6S_3^+$: 533.0187; found: 533.0191. **IR** (Diamond-ATR, neat) $\tilde{\nu}_{max}$: 2925 (m), 2851 (w), 1295 (s), 1261 (m), 1192 (s), 1159 (m), 1127 (vs), 1041 (m), 1020 (m), 890 (m), 856 (m), 798 cm^{-1} (s). **Melting point**: 245 (±2) °C decomposition.

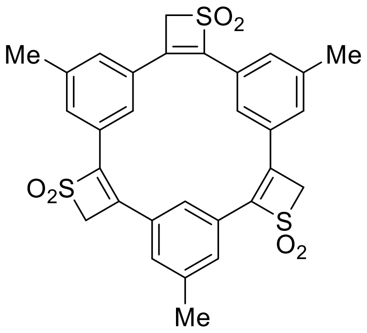

Cyclotris (3-(3'-methylphenyl)thiete 1,1-dioxide-5', 4-diyl) (7b)

Using 3-(3-bromo-5-methylphenyl)-2*H*-thiete 1,1-dioxide (**2n**) according to general procedure **C**, provided **7b** (0.069 mmol, 40 mg, 21%) as white solid. ***R*f** = 0.2 (CH_2Cl_2, UV, $KMnO_4$). **^{1}H NMR** (400 MHz, CD_2Cl_2): *δ* 7.57 (s, 3H), 7.49 (s, 3H), 7.21 (s, 3H), 4.82 (s, 6H), 2.43 ppm (s, 9H). **^{13}C NMR** (101 MHz, CD_2Cl_2): *δ* 150.7, 141.6, 140.4, 131.5, 131.4, 130.9, 128.0, 124.4, 72.6, 21.6 ppm. **LRMS** (DEP/EI-Orbitrap): *m/z* (%): 576.1 (50), 400.1 (100), 384.1 (74), 339.0 (22). **HRMS** (EI-Orbitrap): m/z: [M]+ Calcd for $C_{30}H_{24}O_6S_3^+$: 576.0735; found: 576.0729. **IR** (Diamond-ATR, neat) $\tilde{\nu}_{max}$: 1308 (m), 1293 (s), 1280 (w), 1211 (w), 1188 (s), 1174 (w), 1164 (w), 1131 (vs), 878 (w), 873 (m), 864 (w), 832 (w), 742 (m), 706 (w), 689 (w), 683 cm^{-1} (w). **Melting Point**: >310 °C.

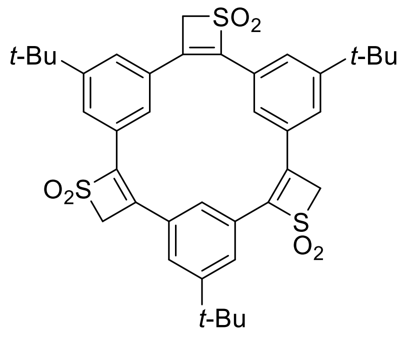

Cyclotris (3-(3’-*tert*-butylphenyl)thiete 1,1-dioxide-5’, 4-diyl) (7c)

Using 3-(3-bromo-5-(*tert*-butyl)phenyl)-2*H*-thiete 1,1-dioxide (**2p**) according to general procedure **C**, provided **7c** (0.064 mmol, 45 mg, 19%) as a white solid. ***R*$_f$** = 0.2 (CH_2Cl_2, UV, $KMnO_4$). **^{1}H NMR** (400 MHz, CD_2Cl_2): *δ* 7.78 (s, 3H), 7.57 (s, 3H), 7.40 (s, 3H), 4.87 (s, 6H), 1.36 ppm (s, 27H). **^{13}C NMR** (101 MHz, CD_2Cl_2)**:** *δ* 154.5, 150.7, 140.4, 131.5, 128.0, 128.0, 127.5, 124.4, 72.6, 35.6, 31.3 ppm. **LRMS** (DEP/EI-Orbitrap): *m/z* (%): 702.2 (40), 638.2 (55), 590.2 (22), 526.3 (62). **HRMS** (EI-Orbitrap): m/z: [M]+ Calcd for $C_{39}H_{42}O_6S_3^+$: 702.2144; found: 702.2133. **IR** (Diamond-ATR, neat) $\tilde{\nu}_{max}$: 1306 (s), 1300 (s), 1254 (m), 1191 (s), 1168 (w), 1132 (vs), 840 (w), 744 (m), 704 (w), 696 cm^{-1} (w). **Melting Point**: 303 (±2) °C decomposition.

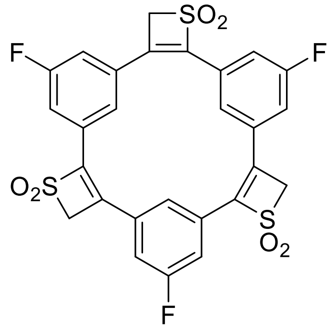

Cyclotris (3-(3’-fluorophenyl)thiete 1,1-dioxide-5’, 4-diyl) (7d)

Using 3-(3-bromo-5-fluorophenyl)-2*H*-thiete 1,1-dioxide (**2s**) according to general procedure **C**, provided **7d** (53 mg, 0.09 mmol, 45%) as yellowish solid. ***R*$_f$** = 0.65 (hexane/EtOAc 5:5, UV, $KMnO_4$). **^{1}H NMR** (400 MHz, $CDCl_3$) δ 7.49 (d, *J* = 8.3 Hz, 3H), 7.36 (s, 3H), 7.22 (d, *J* = 8.5 Hz, 3H), 4.90 ppm (s, 6H). **^{13}C NMR** (101 MHz, $CDCl_3$) δ 163.4 (d, *J* = 253.2 Hz), 138.94, 132.95, 123.2-123.1 (m), 119.03, 118.80, 117.83, 117.59, 71.43 ppm. **HRMS** (ESI-Quadrupole): m/z: [M$^+$-H] Calcd for $C_{27}H_{14}F_3O_6S_3^+$: 586.9905; found: 586.9912. **IR** (Diamond-ATR, neat) $\tilde{\nu}_{max}$: 1589 (m), 1347 (m), 1308 (s), 1201 (m), 1185 (vs), 1132 (vs), 870 cm^{-1} (m). **Melting point**: 250 (±2) °C decomposition.

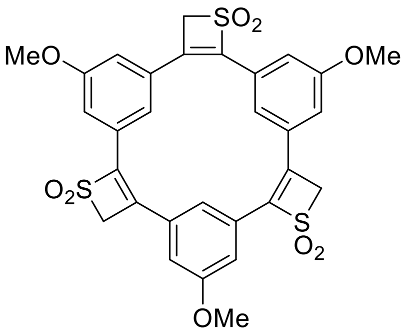

Cyclotris (3-(3'-methoxyphenyl)thiete 1,1-dioxide-5', 4-diyl) (7e)

Using 3-(3-bromo-5-methoxyphenyl)-2*H*-thiete 1,1-dioxide (**2q**) according to general procedure **C**, provided **7e** (2.14 g, 3.43 mmol, 42%) as yellowish solid. R_f = 0.2 (hexane/EtOAc 5:5, UV, $KMnO_4$). **^{1}H NMR** (400 MHz, $CDCl_3$) δ 7.27 (s, 3H), 7.22-7.20 (m, 3H), 6.89-6.87 (m, 3H), 4.83 (s, 6H), 3.89 ppm (s, 9H). **^{13}C NMR** (101 MHz, $CDCl_3$) δ 160.8, 150.6, 139.7, 132.1, 128.8, 119.0, 116.7, 114.7, 72.1, 56.0 ppm. **HRMS** (ESI-Quadrupole): m/z: [M$^+$-H] Calcd for $C_{30}H_{23}O_9S_3^+$: 623.0504; found: 623.0507. **IR** (Diamond-ATR, neat) $\tilde{\nu}_{max}$: 1585 (m), 1362 (m), 1291 (s), 1261 (s), 1226 (s), 1182 (s), 1156 (m), 1129 (vs), 1064 (s), 1050 (s), 1017 (m), 1000 (s), 985 (s), 861 (m), 822 (m), 806 (m), 800 (m), 736 cm^{-1} (m). **Melting point**: 265 (±2) °C decomposition.

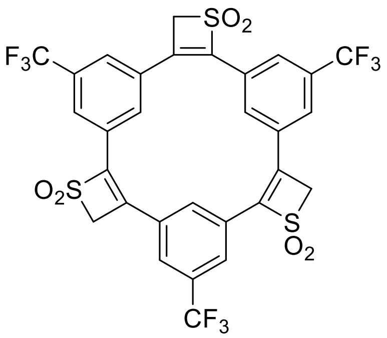

Cyclotris (3-(3'-(trifluoromethyl)phenyl)thiete 1,1-dioxide-5', 4-diyl) (7f)

Using 3-(3-bromo-5-(trifluoromethyl)phenyl)-2*H*-thiete 1,1-dioxide (**2t**) according to general procedure **C**, provided **7f** (0.050 mmol, 37 mg, 15%) as a white solid. R_f = 0.2 (CH_2Cl_2, UV, $KMnO_4$). NMR not recordable due to insolubility in common deuterated solvents. **LRMS** (DEP/EI-Orbitrap): *m/z* (%): 738.1 (2), 674.1 (8), 610.1 (2), 83.9 (100). **HRMS** (EI-Orbitrap): m/z: [M]+ Calcd for $C_{30}H_{15}F_9O_6S_3^+$: 737.9887; found: 737.9883. **IR** (Diamond-ATR, neat) $\tilde{\nu}_{max}$: 1379 (w), 1309 (s), 1280 (s), 1194 (m), 1182 (m), 1131 (vs), 1115 (s), 1056 (m), 1034 (m), 1020 (m), 1009 (m), 931 (w), 912 (m), 886 (m), 813 (m), 742 (w), 704 (s), 687 cm^{-1} (m). **Melting Point**: 250 (±2) °C decomposition.

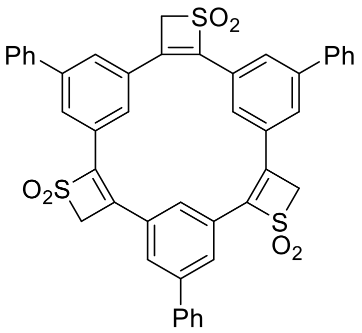

Cyclotris (3-(3’-biphenyl)thiete 1,1-dioxide-5’, 4-diyl) (7g)

Using 3-(5-bromo-[1,1'-biphenyl]-3-yl)-2*H*-thiete 1,1-dioxide (**2r**) according to general procedure **C**, provided **7g** (0.043 mmol, 38 mg, 15%) as a white solid. ***R*f** = 0.2 (CH_2Cl_2, UV, $KMnO_4$). **^{1}H NMR** (400 MHz, CD_2Cl_2): *δ* 7.99 (t, *J* = 1.6 Hz, 3H), 7.75 (t, *J* = 1.6 Hz, 3H), 7.69-7.59 (m, 9H), 7.55-7.38 (m, 9H), 4.95 ppm (s, 6H). **^{13}C NMR** (101 MHz, CD_2Cl_2): *δ* 151.0, 144.4, 140.7, 138.9, 132.2, 129.7, 129.5, 129.3, 129.0, 128.7, 127.8, 125.8, 72.9 ppm. **LRMS** (DEP/EI-Orbitrap): *m/z* (%): 762.1 (16), 714.2 (46), 666.1 (48) 602.2 (100), 586.2 (42), 63.9 (51). **HRMS** (EI-Orbitrap): m/z: [M]+ Calcd for $C_{45}H_{30}O_6S_3^+$: 762.1205; found: 762.1198. **IR** (Diamond-ATR, neat) $\tilde{\nu}_{max}$: 1592 (w), 1362 (w), 1307 (s), 1264 (w), 1188 (s), 1130 (vs), 1078 (w), 906 (w), 894 (w), 882 (w), 871 (w), 799 (w), 766 (s), 733 (m), 698 (s), 680 (m), 657 cm^{-1} (w). **Melting Point**: 255 (±2) °C

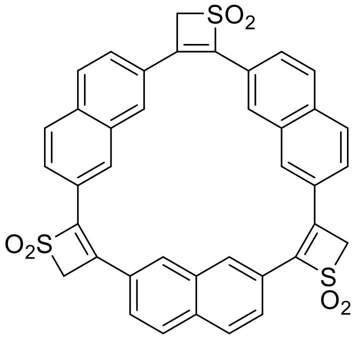

Cyclotris (3-(2’-naphtyl)thiete 1,1-dioxide-7’, 4-diyl) (7h)

Using 3-(7-bromonaphthalen-2-yl)-2*H*-thiete 1,1-dioxide (**2m**) according to general procedure **C**, provided **7h** (27 mg, 0.04 mmol, 20%) as brownish solid. ***R*f** = 0.8 (CH_2Cl_2/MeOH 96:4, UV, $KMnO_4$). **^{1}H NMR** (400 MHz, $CDCl_3$) δ 8.28 (s, 3H), 8.14 (s, 3H), 8.01 (d, *J* = 8.6 Hz, 6H), 7.93 (dd, *J* = 8.5, 1.5 Hz, 3H), 7.46 (dd, *J* = 8.5, 1.6 Hz, 3H), 4.97 ppm (s, 6H). **^{13}C NMR** (101 MHz, $CDCl_3$) δ 150.7, 139.5, 134.9, 132.5, 129.8, 129.6, 128.8, 128.0, 127.8, 126.9, 126.2, 126.0, 71.9 ppm. **HRMS** (ESI-Quadrupole): m/z: [M$^+$-H] Calcd for $C_{39}H_{23}O_6S_3^+$: 683.0657; found: 683.0665. **IR** (Diamond-ATR, neat) $\tilde{\nu}_{max}$: 1302 (vs), 1183 (vs), 1126 (vs), 1120 (vs), 917 (m), 911 (m), 847 (s), 813 (m), 729 cm^{-1} (m). **Melting point**: 290 (±2) °C decomposition.

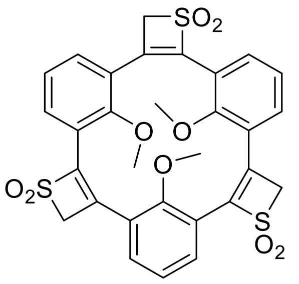

Cyclotris (3-(2’-methoxyphenyl)thiete 1,1-dioxide-3’, 4-diyl) (7i)

Using 3-(3-bromo-2-methoxyphenyl)-2*H*-thiete 1,1-dioxide (**2o**) according to general procedure **C**, provided **7i** (0.050 mmol, 31 mg, 15%) as a yellow solid. ***R*f** = 0.2 (CH_2Cl_2, UV, $KMnO_4$). NMR not recordable due to insolubility in common deuterated solvents. **LRMS** (DEP/EI-Orbitrap): *m/z* (%): 624.1 (15), 545.1 (30), 418.0 (30), 339.0 (100). **HRMS** (EI-Orbitrap): m/z: [M]+ Calcd for $C_{30}H_{24}O_9S_3^+$: 624.0582; found: 624.0582.

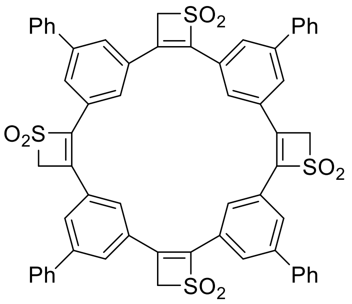

Cyclotetrakis (3-(3’-biphenyl)thiete 1,1-dioxide-5’, 4-diyl) (8a)

Using 3-(5-bromo-[1,1'-biphenyl]-3-yl)-2*H*-thiete 1,1-dioxide (**2r**) according to general procedure **C**, provided **8a** (0.043 mmol, 15 mg, 6%) as a white solid. ***R*f** = 0.1 (CH_2Cl_2, UV, $KMnO_4$). **^{1}H NMR** (400 MHz, DMSO-*d*6, 80 °C): δ 7.93 (s, 4H), 7.87 (t, J = 1.7 Hz, 4H), 7.70 (s, 4H), 7.63 – 7.34 (m, 20H), 5.26 (s, 8H). **LRMS** (DEP/EI-Orbitrap): *m/z* (%): 83.9 (100), 872.4 (30), 920.6 (15), 1016.9 (10). **HRMS** (EI-Orbitrap): m/z: [M]+ Calcd for $C_{60}H_{40}O_8S_4^+$: 1016.1606; found: 1016.1593.

3. NMR-Spectra

3-(Anthracen-9-yl)-2*H*-thiete 1,1-dioxide (2c)

^{1}H NMR (400 MHz, $CDCl_3$) and **^{13}C NMR** (101 MHz, $CDCl_3$)

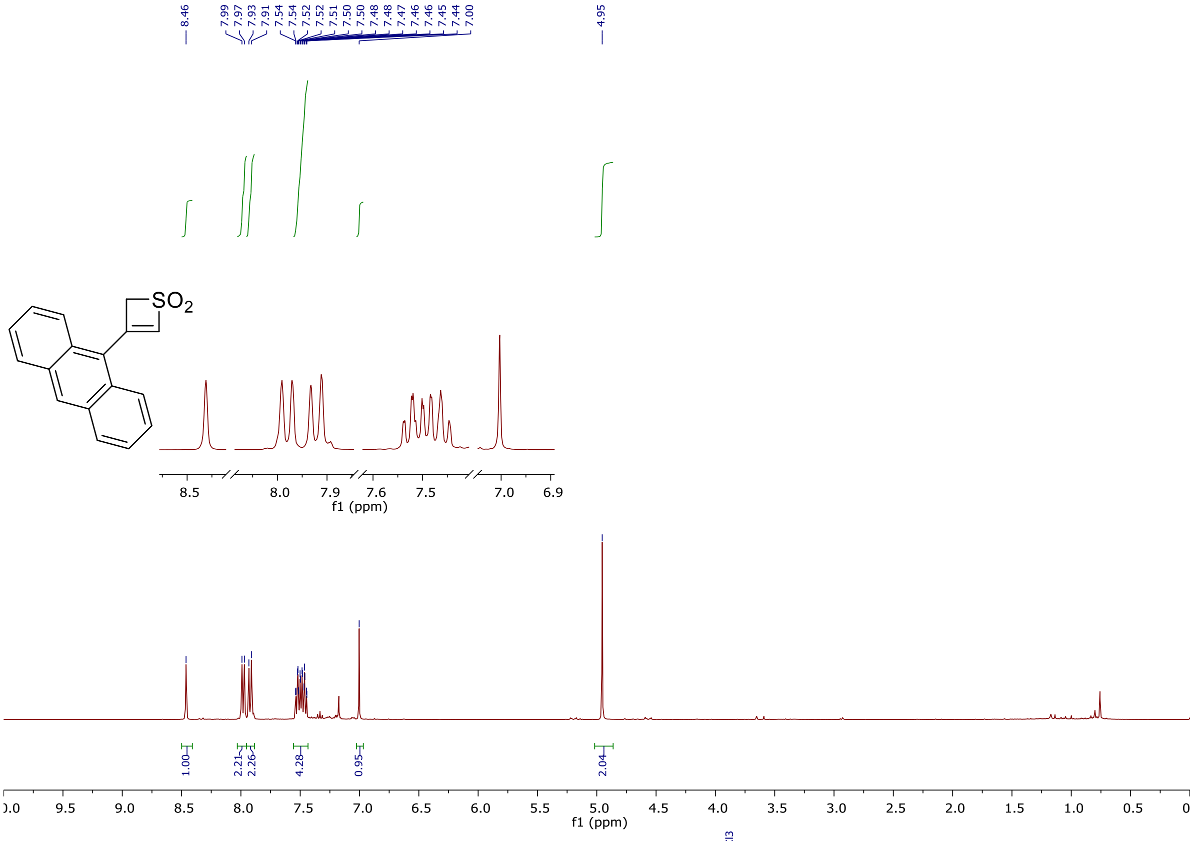

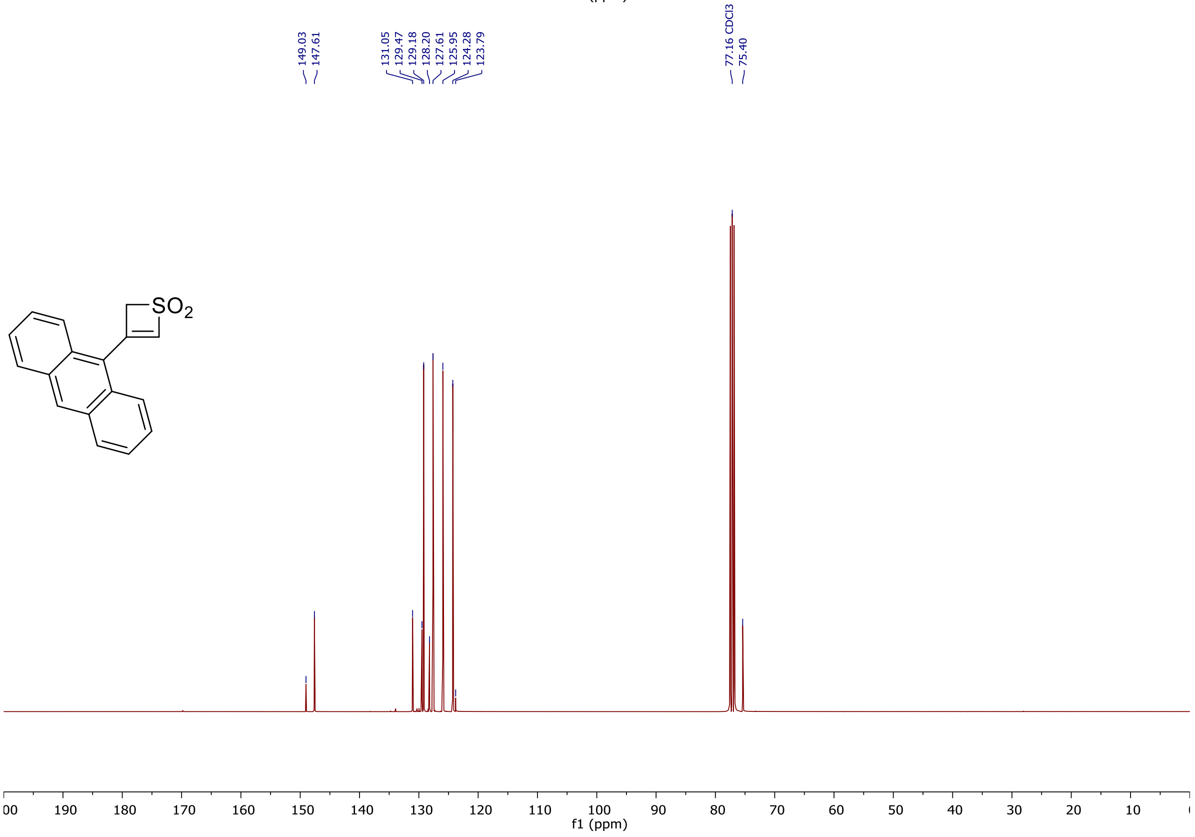

3-Mesityl-2*H*-thiete 1,1-dioxide (2e)

^{1}H NMR (400 MHz, $CDCl_3$) and **^{13}C NMR** (101 MHz, $CDCl_3$)

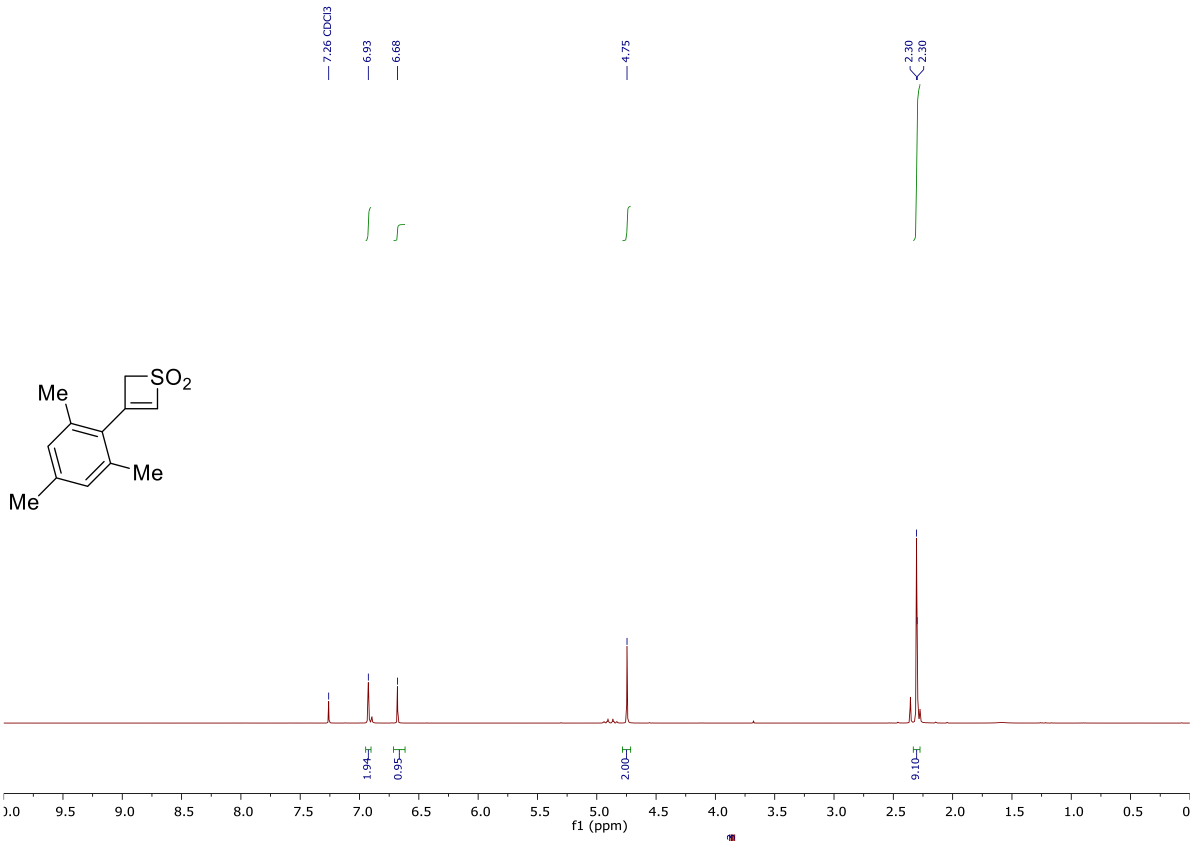

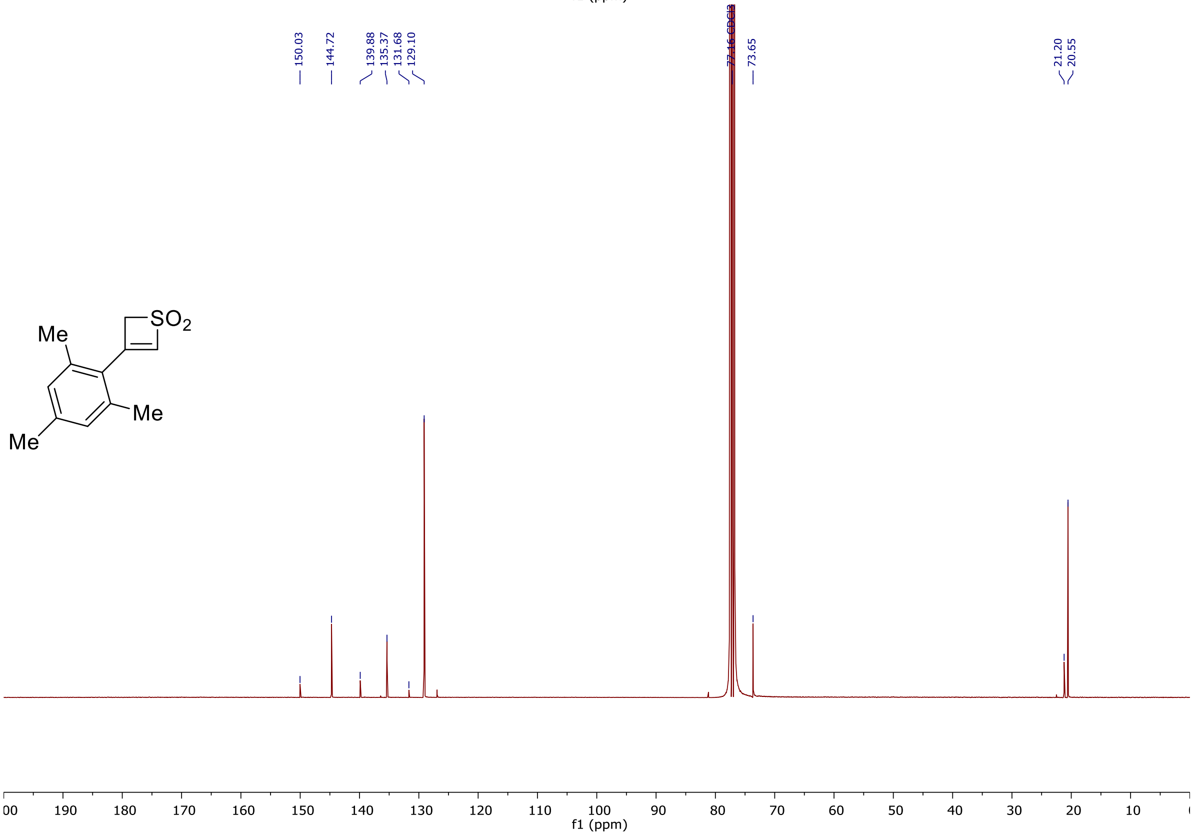

3-(2-Methoxynaphthalen-1-yl)-2*H*-thiete 1,1-dioxide (2g)

^{1}H NMR (400 MHz, $CDCl_3$) and **^{13}C NMR** (101 MHz, $CDCl_3$)

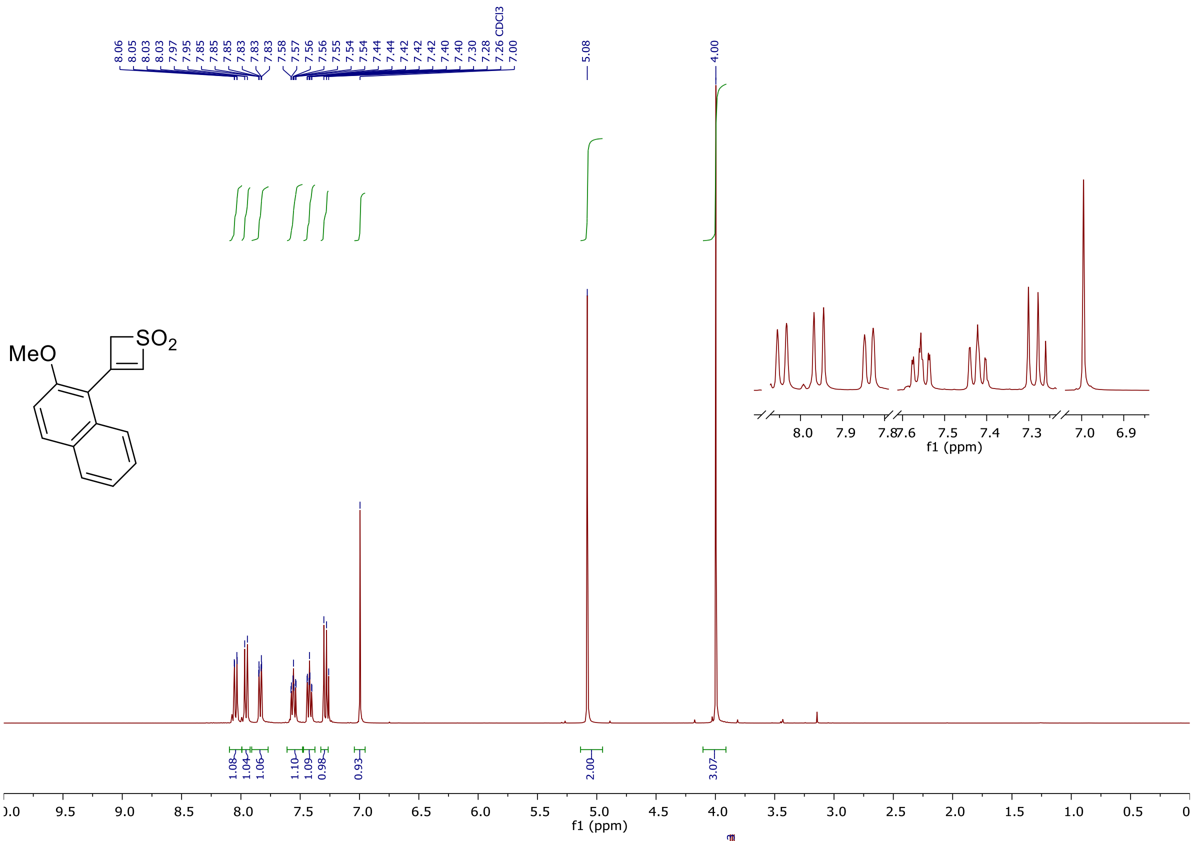

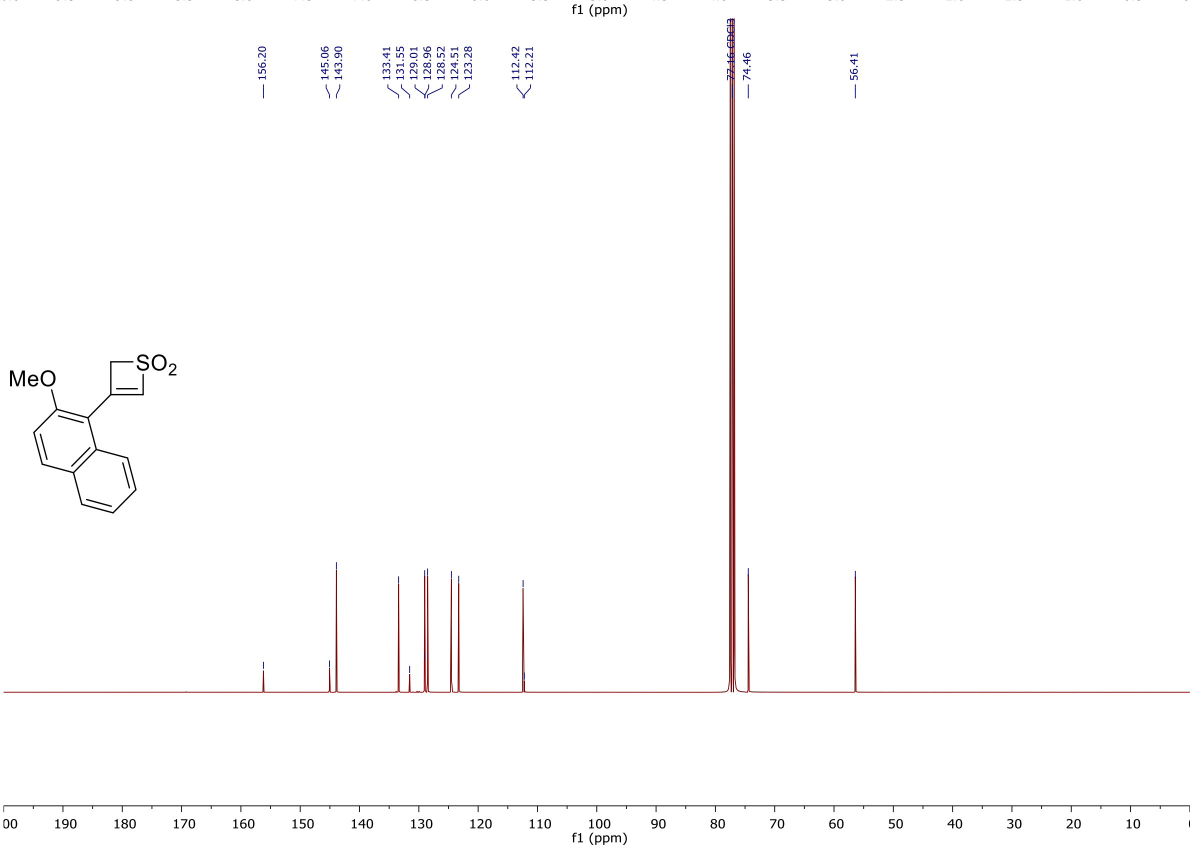

3-(7-Bromonaphthalen-2-yl)-2*H*-thiete 1,1-dioxide (2m)

^{1}H NMR (400 MHz, $CDCl_3$) and **^{13}C NMR** (101 MHz, $CDCl_3$)

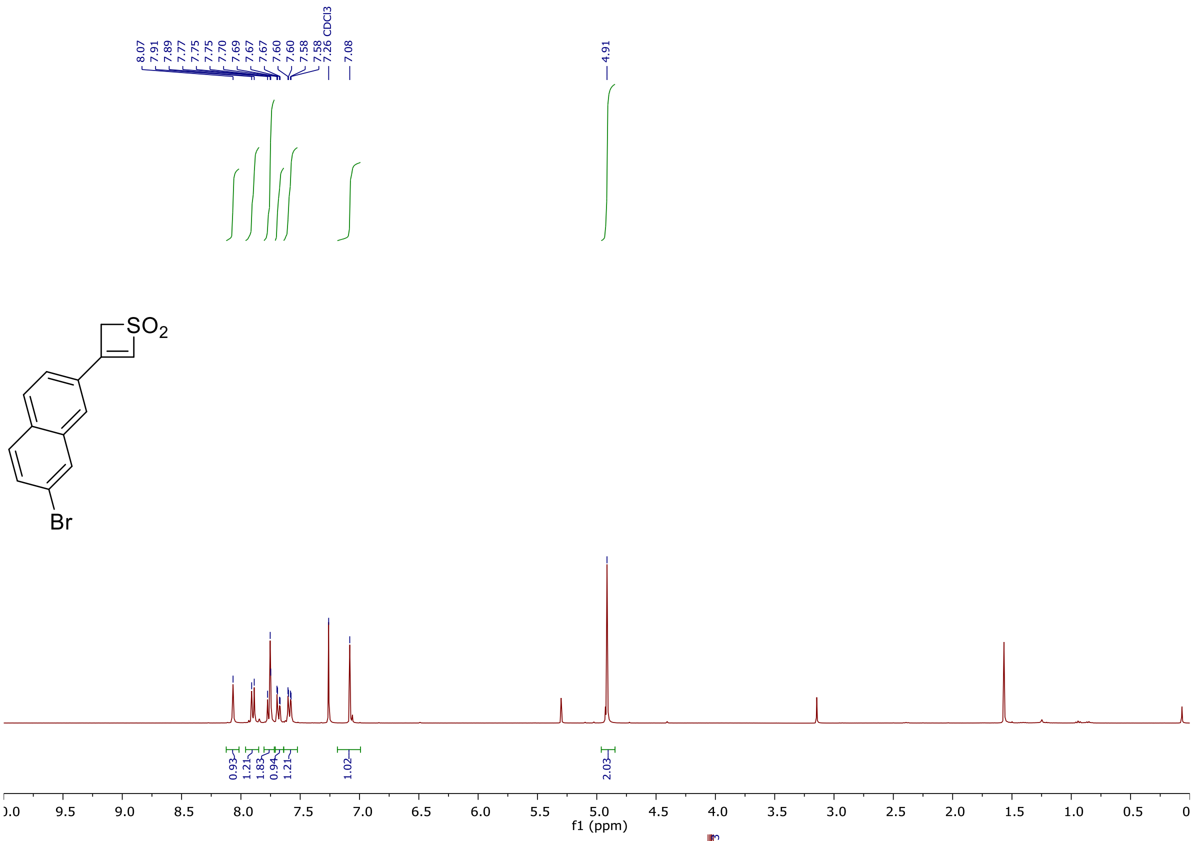

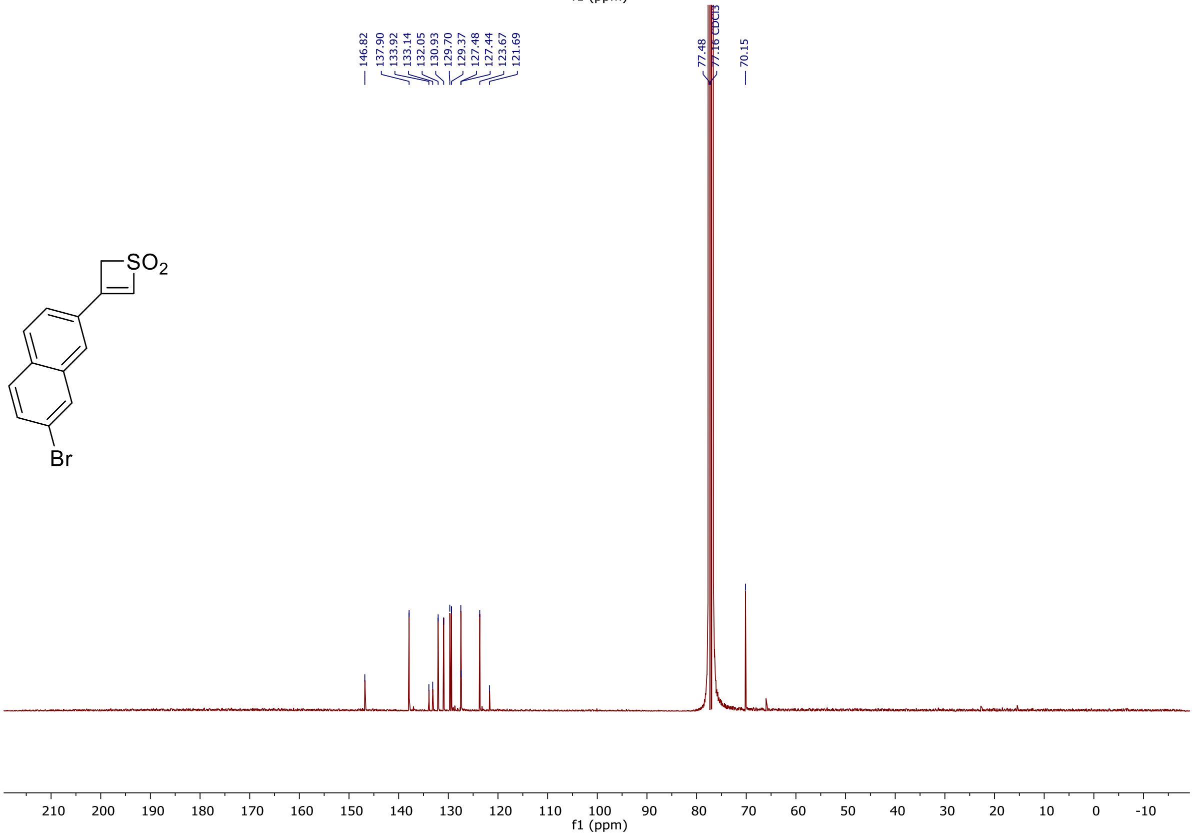

3-(3-Bromo-5-methylphenyl)-2H-thiete 1,1-dioxide (2n)

^{1}H NMR (400 MHz, $CDCl_3$) and **^{13}C NMR** (101 MHz, $CDCl_3$)

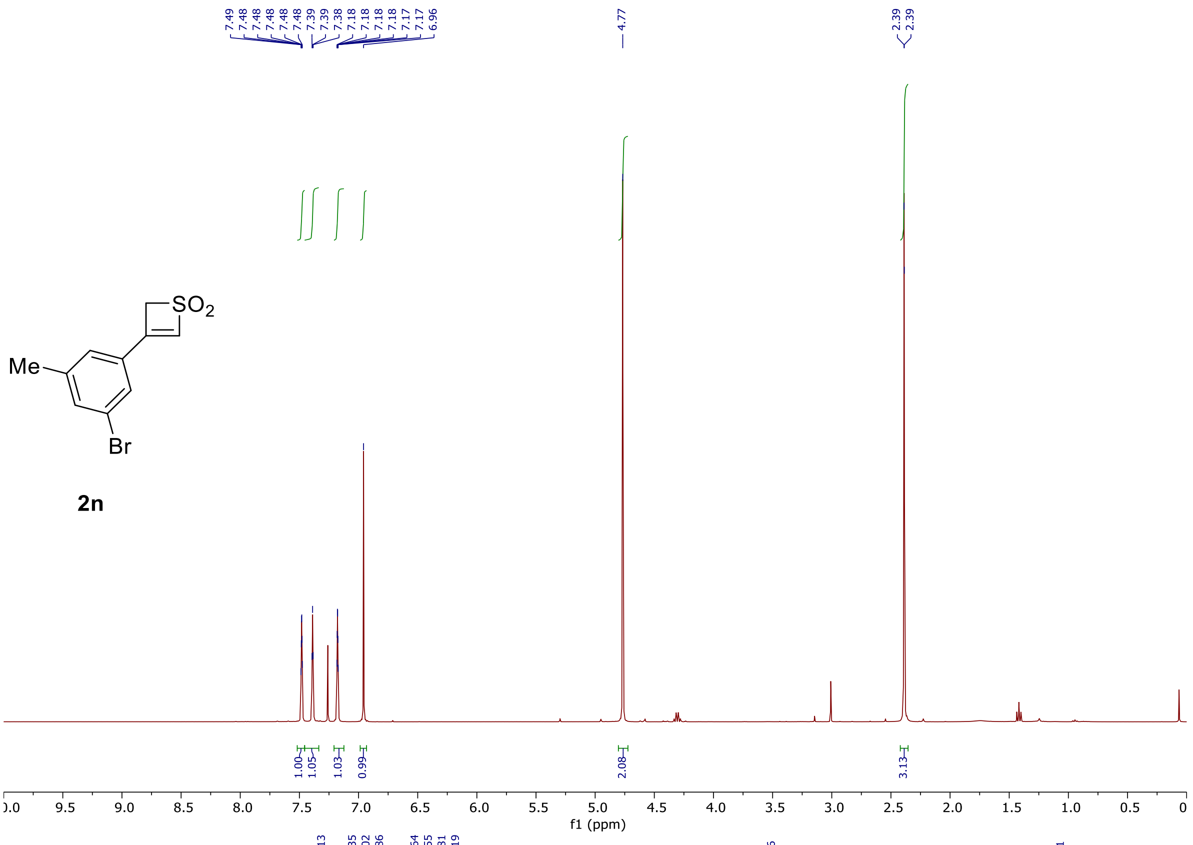

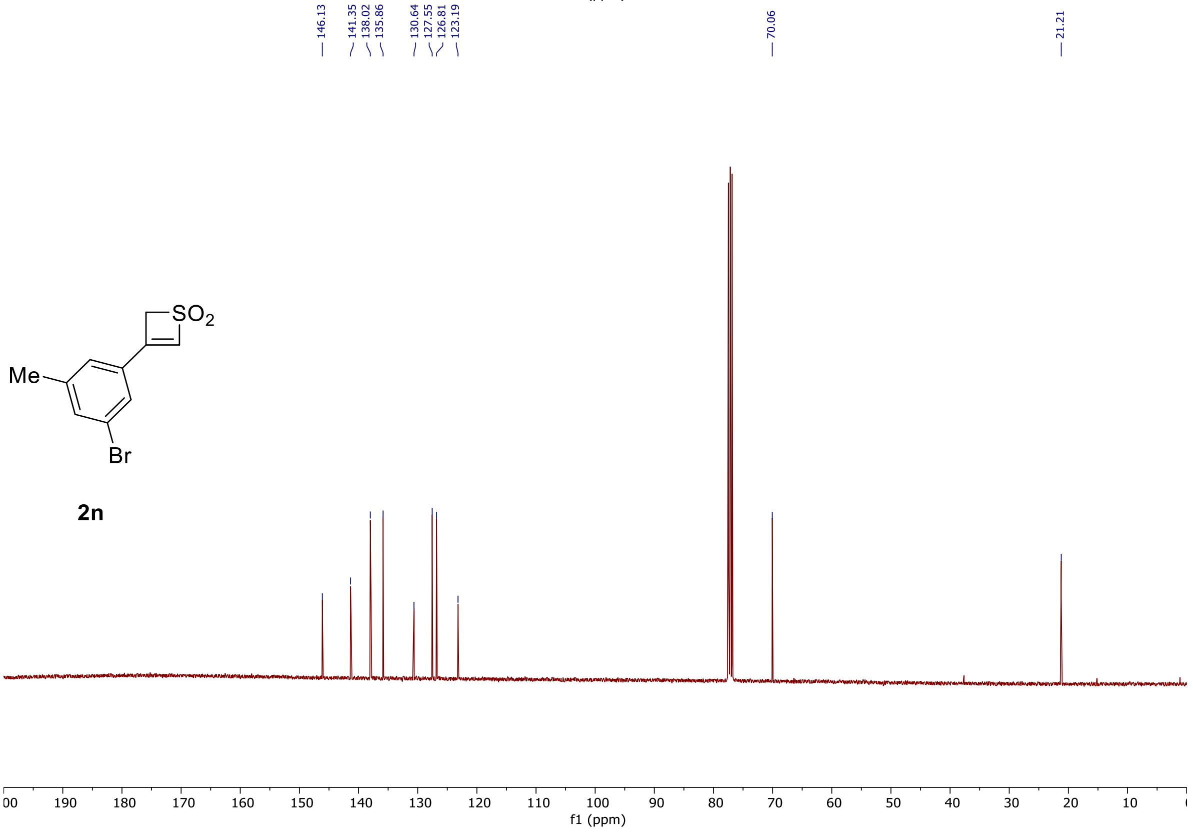

3-(3-Bromo-2-methoxyphenyl)-2*H*-thiete 1,1-dioxide (2o)

^{1}H NMR (400 MHz, $CDCl_3$) and **^{13}C NMR** (101 MHz, $CDCl_3$)

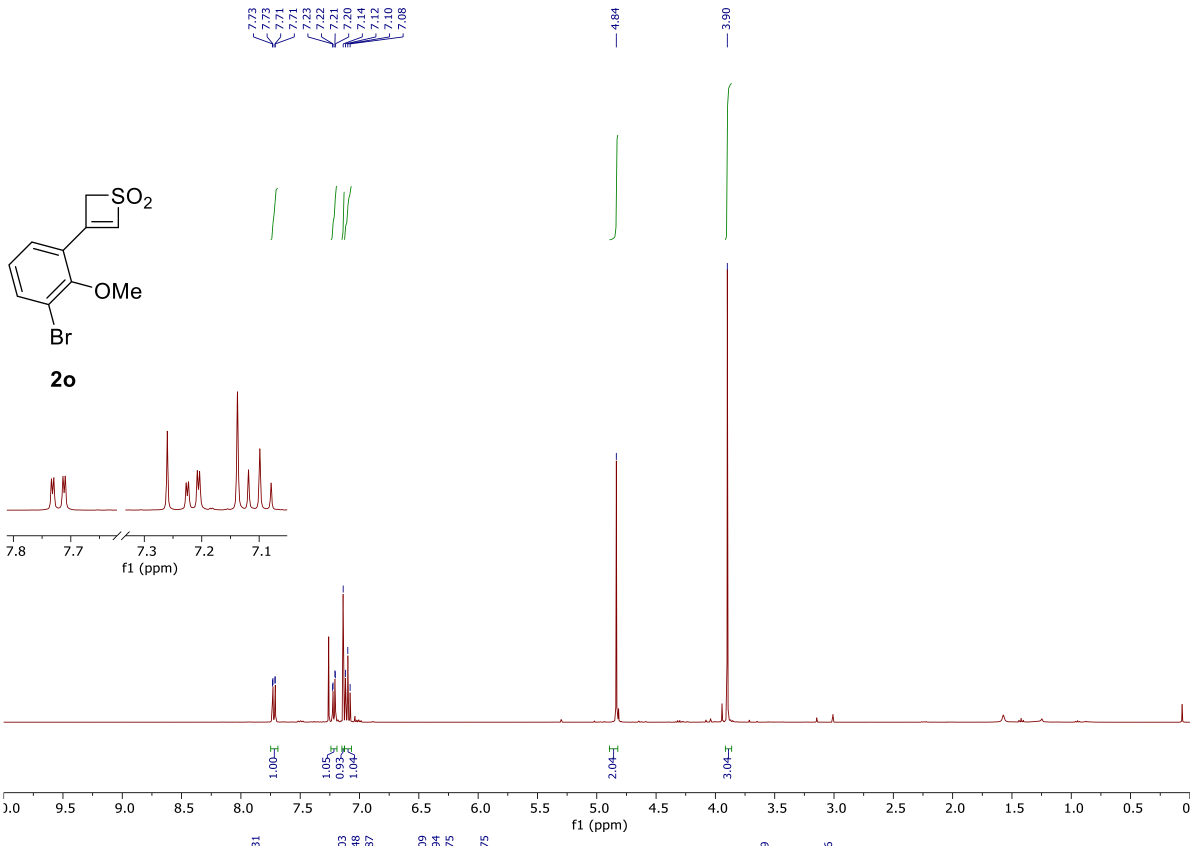

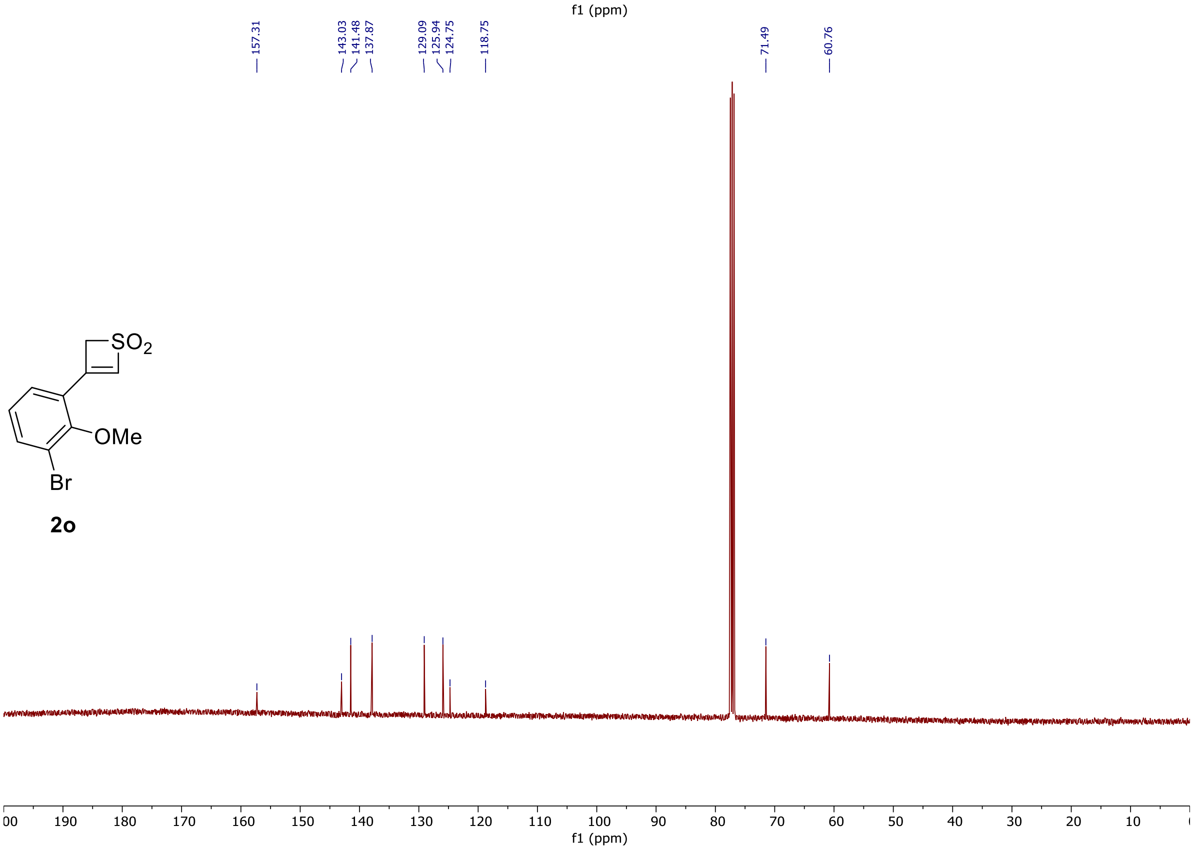

3-(3-Bromo-5-(*tert*-butyl)phenyl)-2*H*-thiete 1,1-dioxide (2p)

^{1}H NMR (400 MHz, $CDCl_3$) and **^{13}C NMR** (101 MHz, $CDCl_3$)

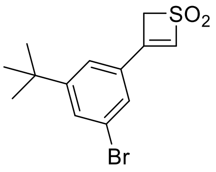

2p

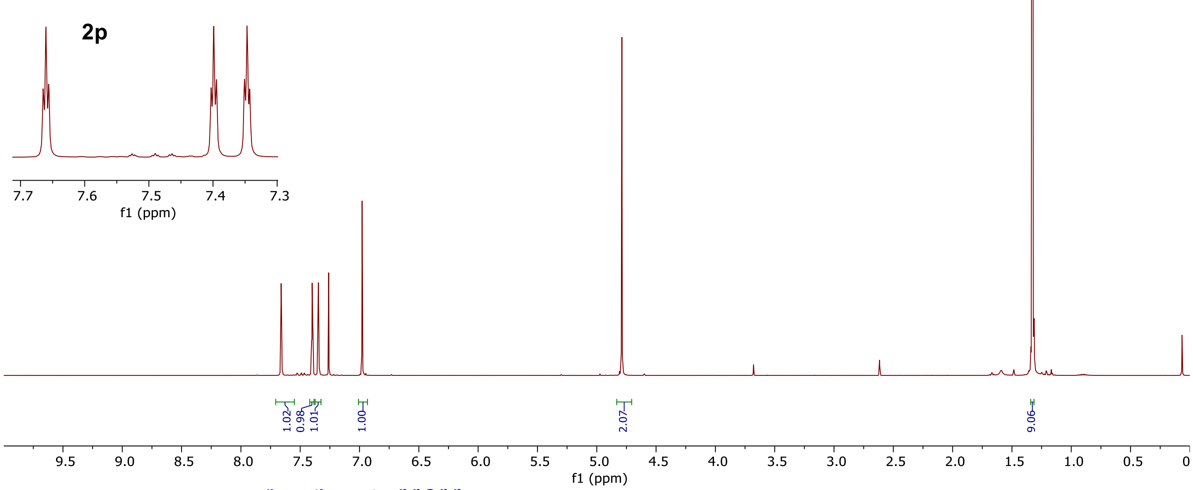

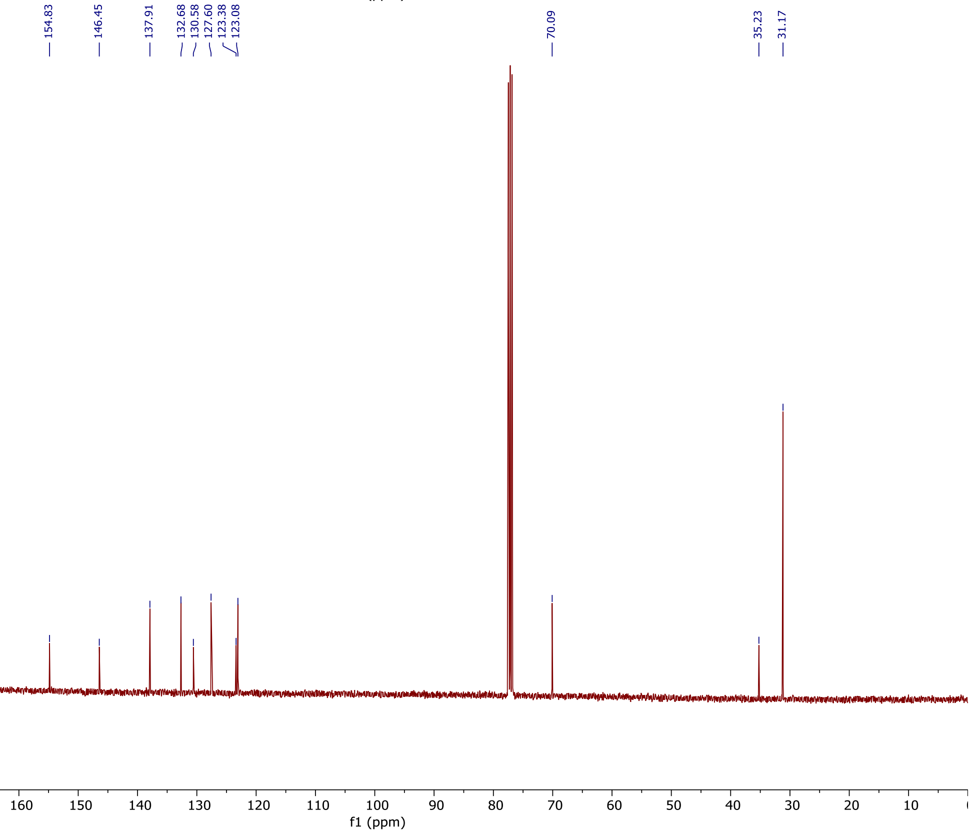

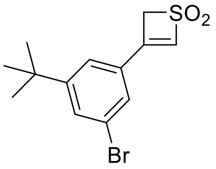

2p

3-(3-Bromo-5-methoxyphenyl)-2*H*-thiete 1,1-dioxide (2q)

^{1}H NMR (400 MHz, $CDCl_3$) and **^{13}C NMR** (101 MHz, $CDCl_3$)

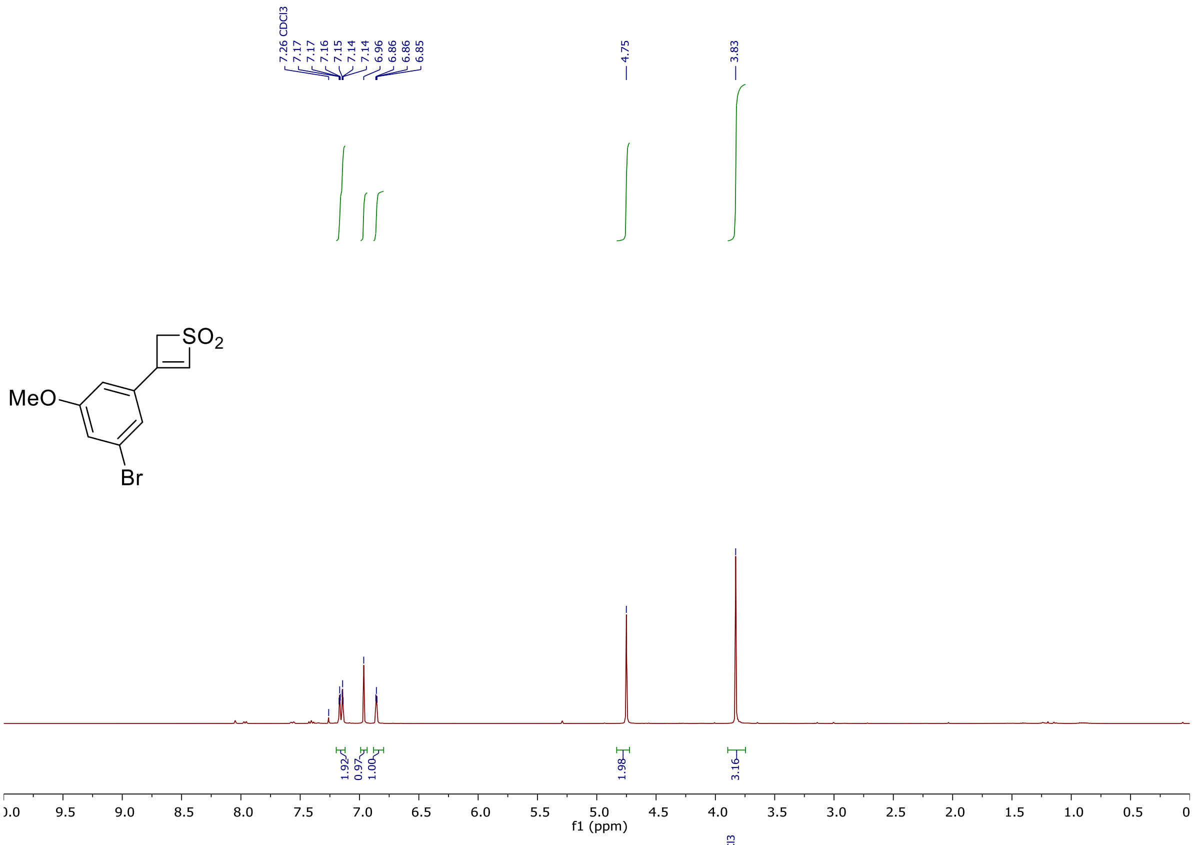

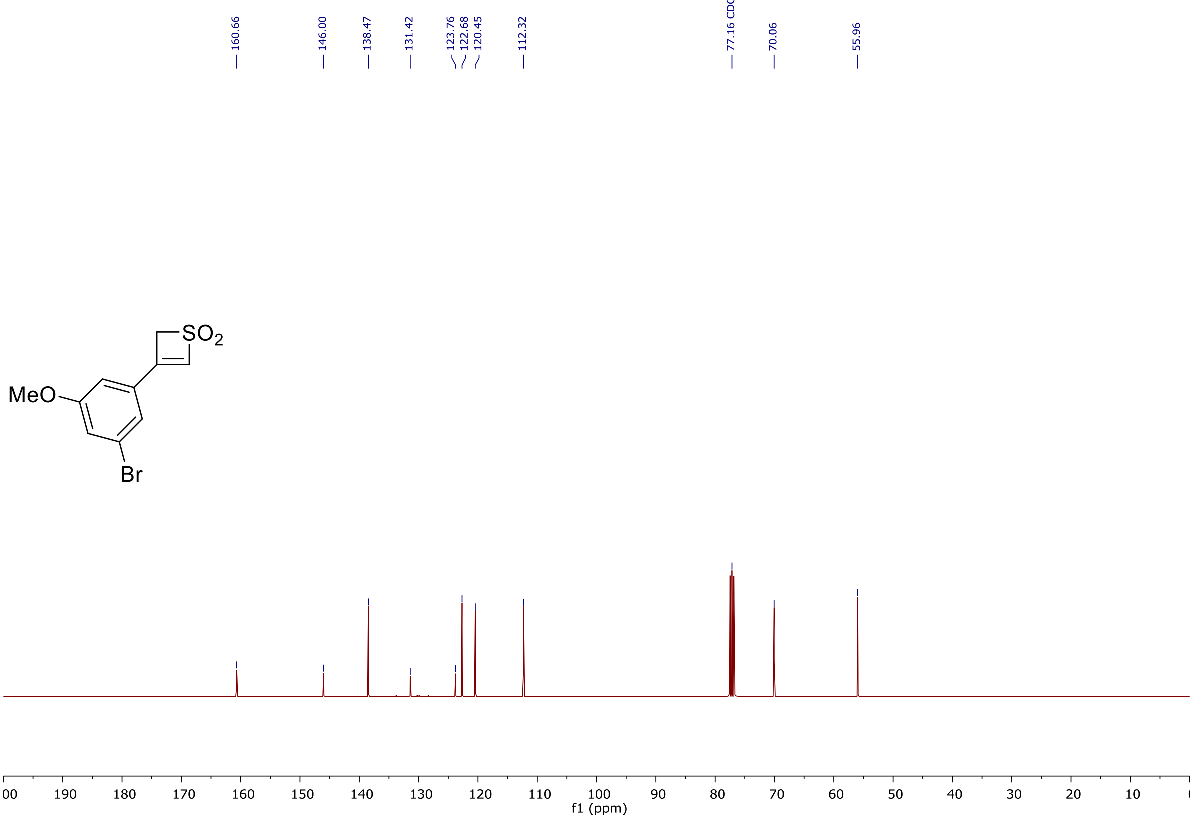

3-(5-Bromo-[1,1'-biphenyl]-3-yl)-2*H*-thiete 1,1-dioxide (2r)

^{1}H NMR (400 MHz, $CDCl_3$) and **^{13}C NMR** (101 MHz, $CDCl_3$)

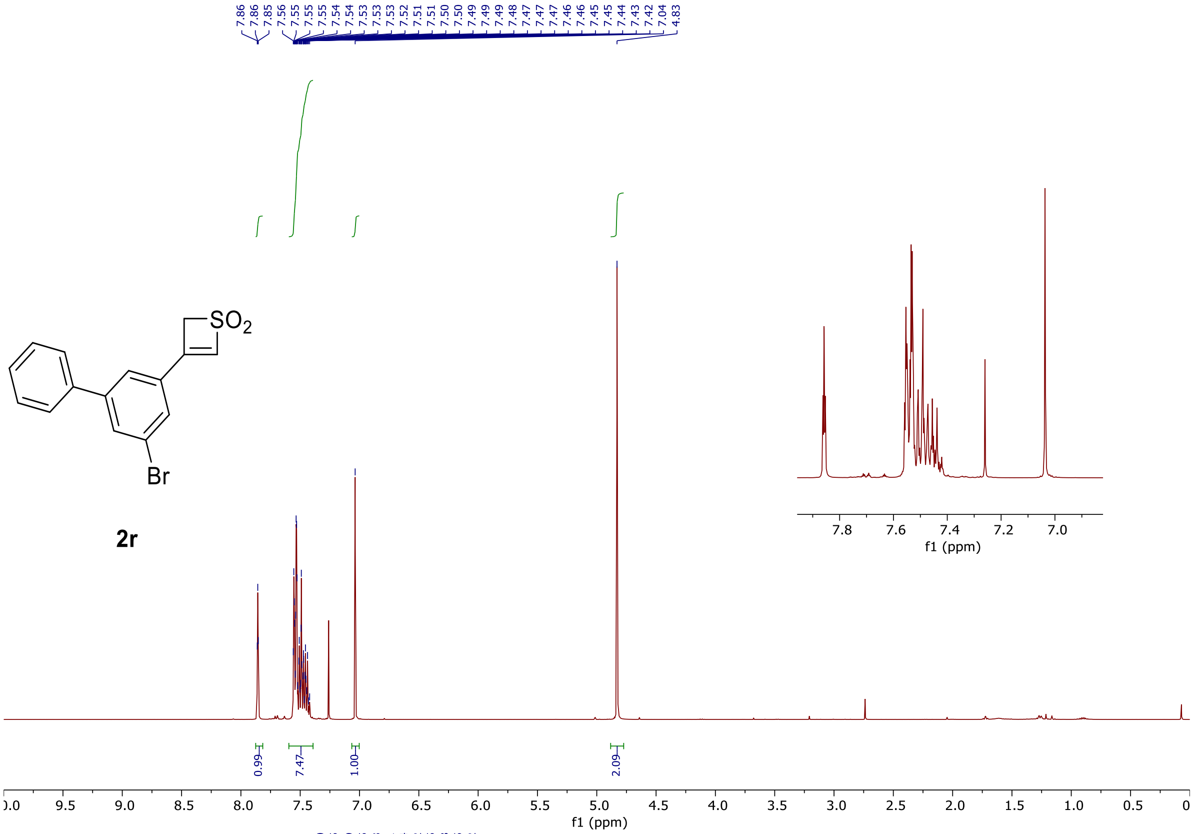

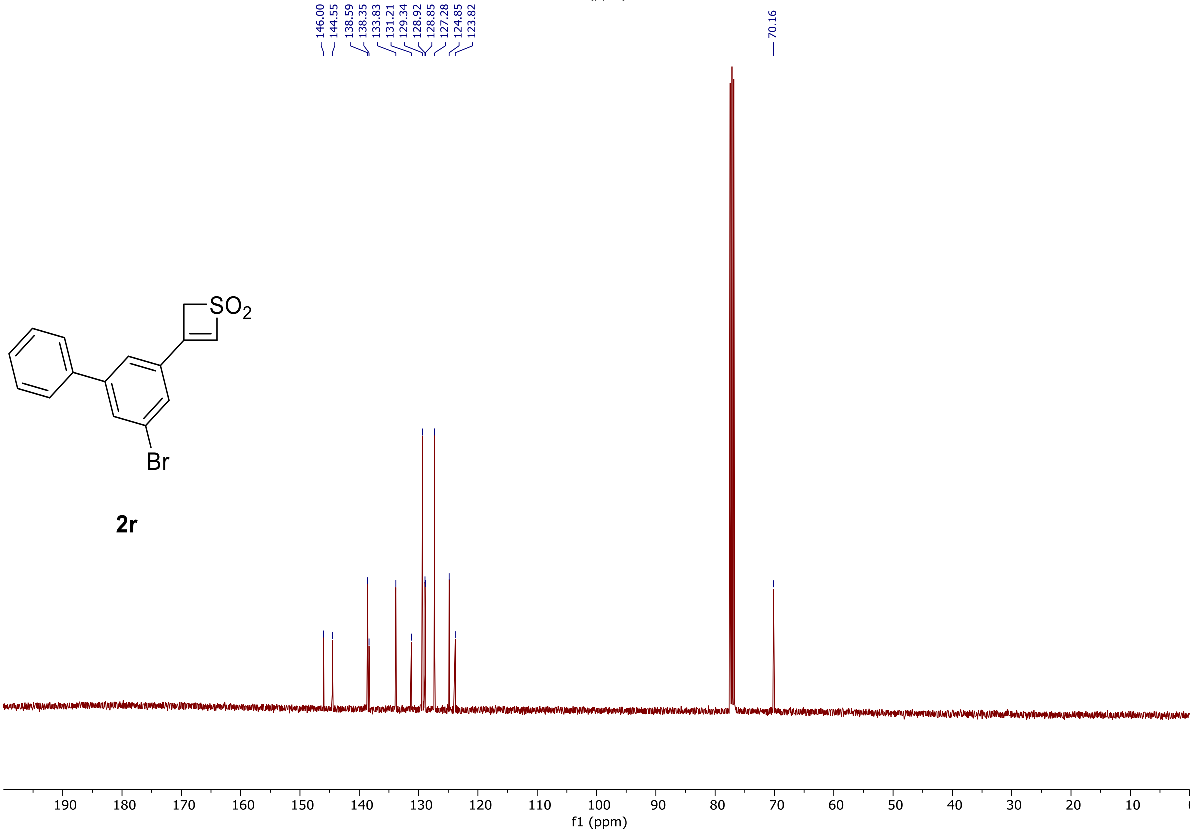

3-(3-Bromo-5-fluorophenyl)-2*H*-thiete 1,1-dioxide (2s)

^{1}H NMR (400 MHz, $CDCl_3$) and **^{13}C NMR** (101 MHz, $CDCl_3$)

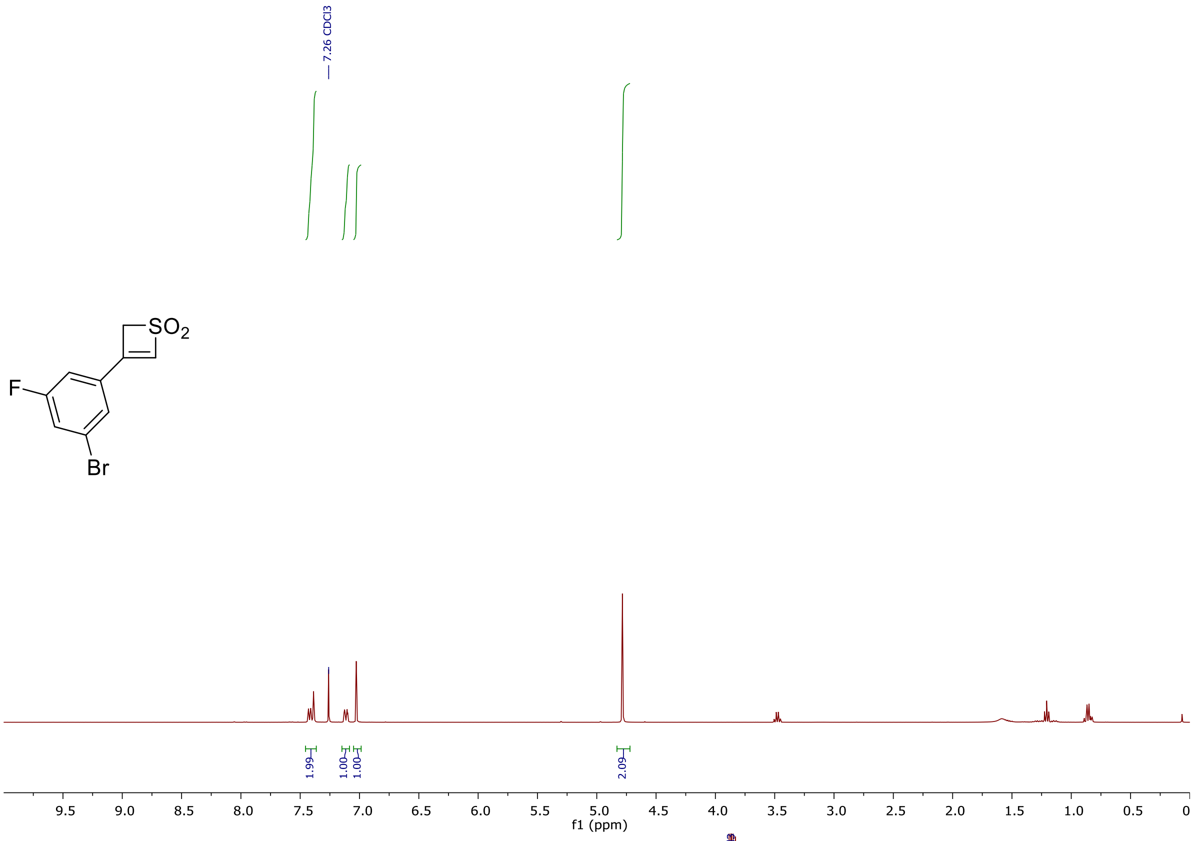

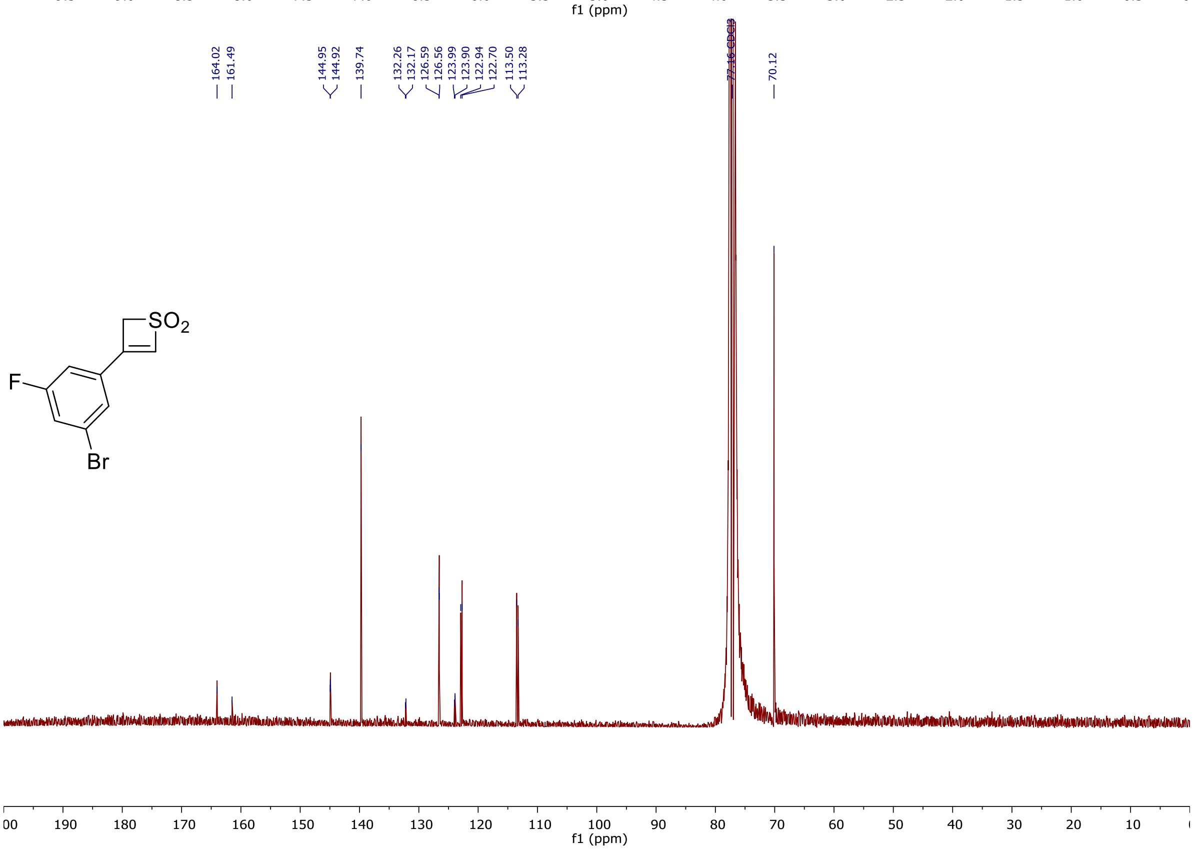

3-(3-Bromo-5-(trifluoromethyl)phenyl)-2*H*-thiete 1,1-dioxide (2t)

^{1}H NMR (400 MHz, $CDCl_3$) and **^{13}C NMR** (101 MHz, $CDCl_3$)

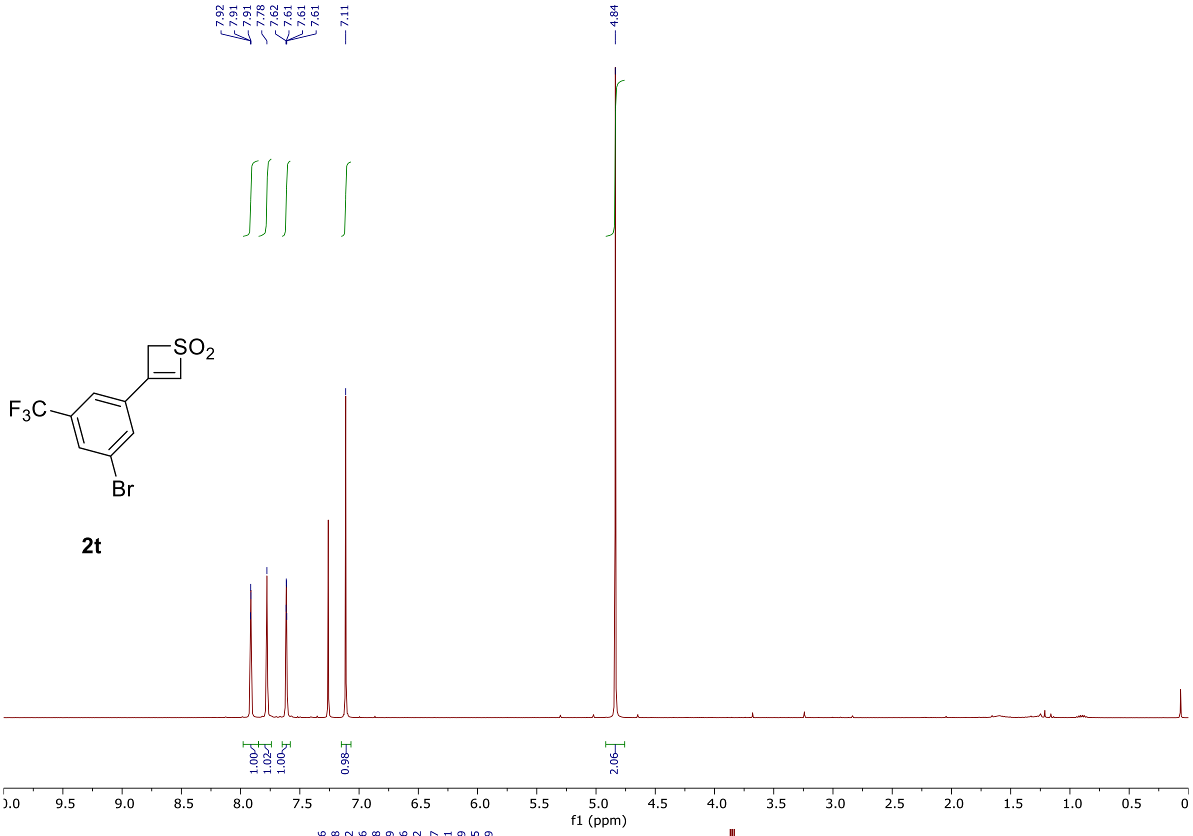

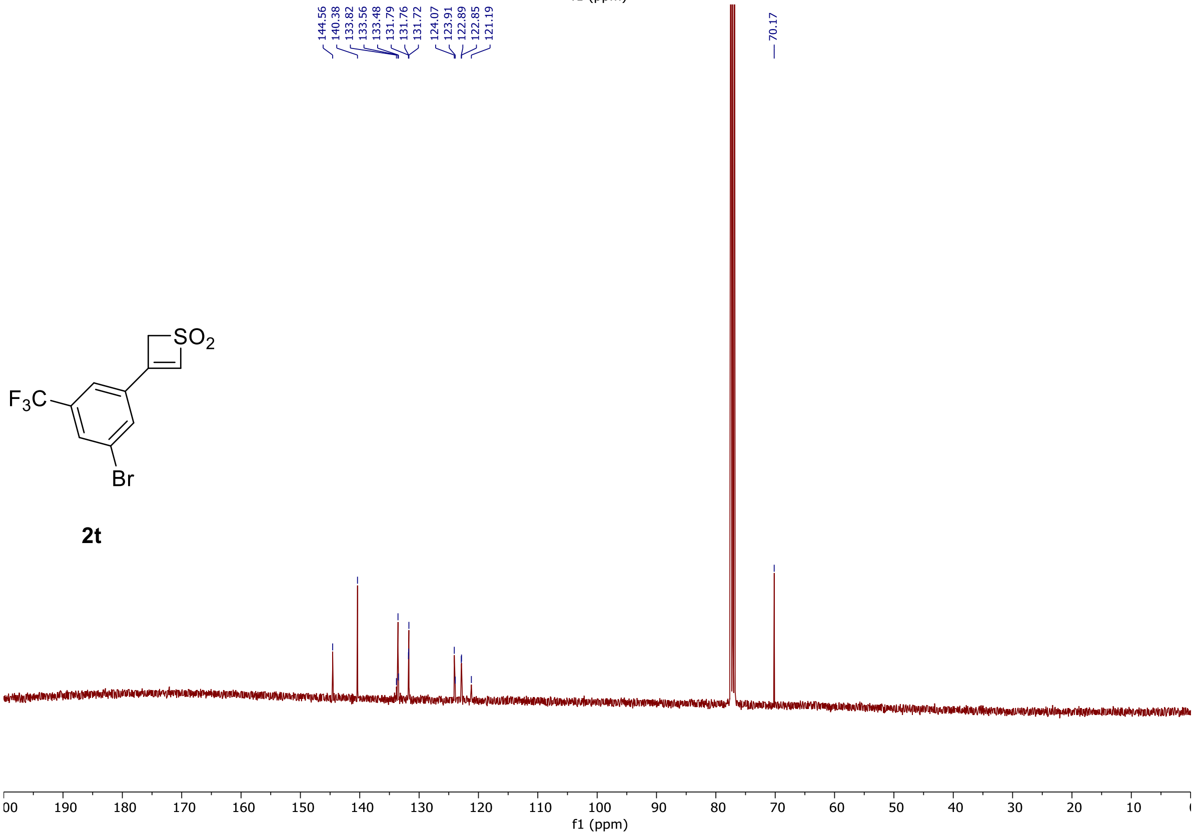

3-(4-Bromophenyl)-2*H*-thiete 1,1-dioxide (2u)

^{1}H NMR (400 MHz, $CDCl_3$) and **^{13}C NMR** (101 MHz, $CDCl_3$)

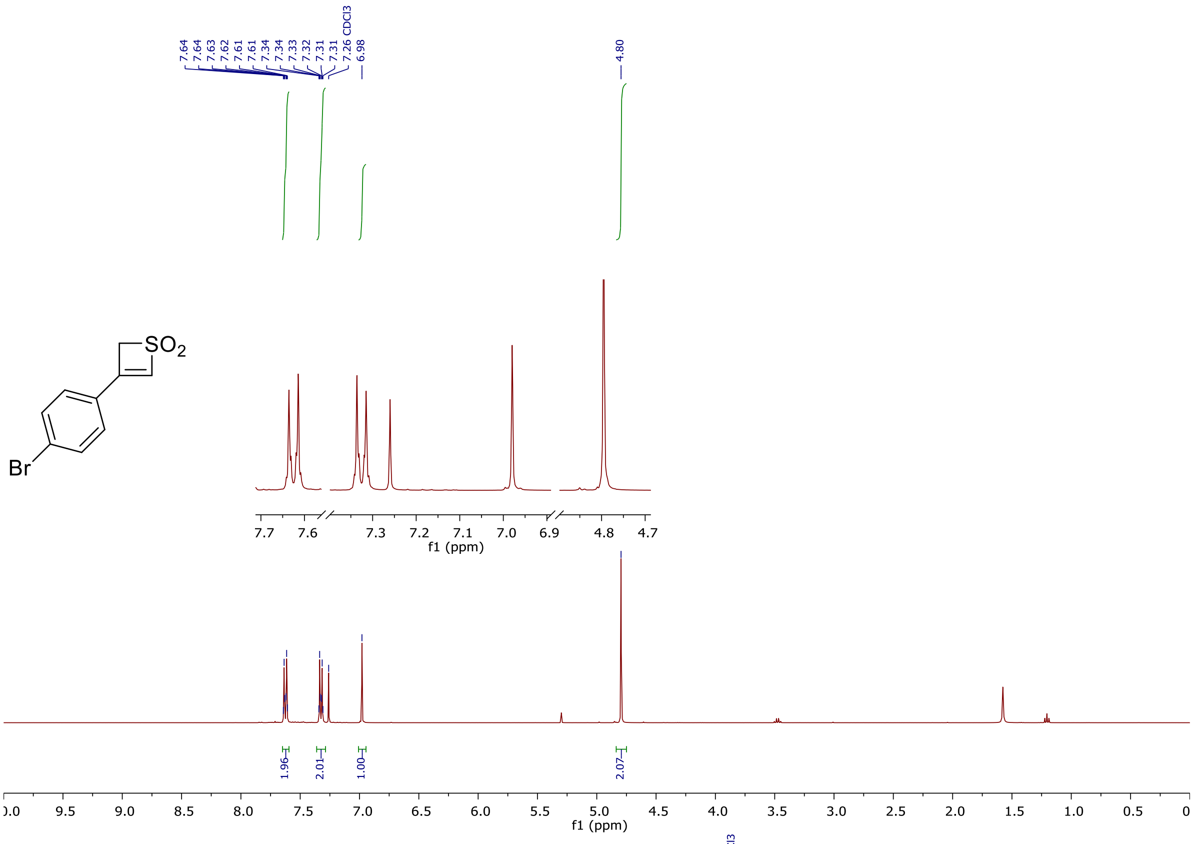

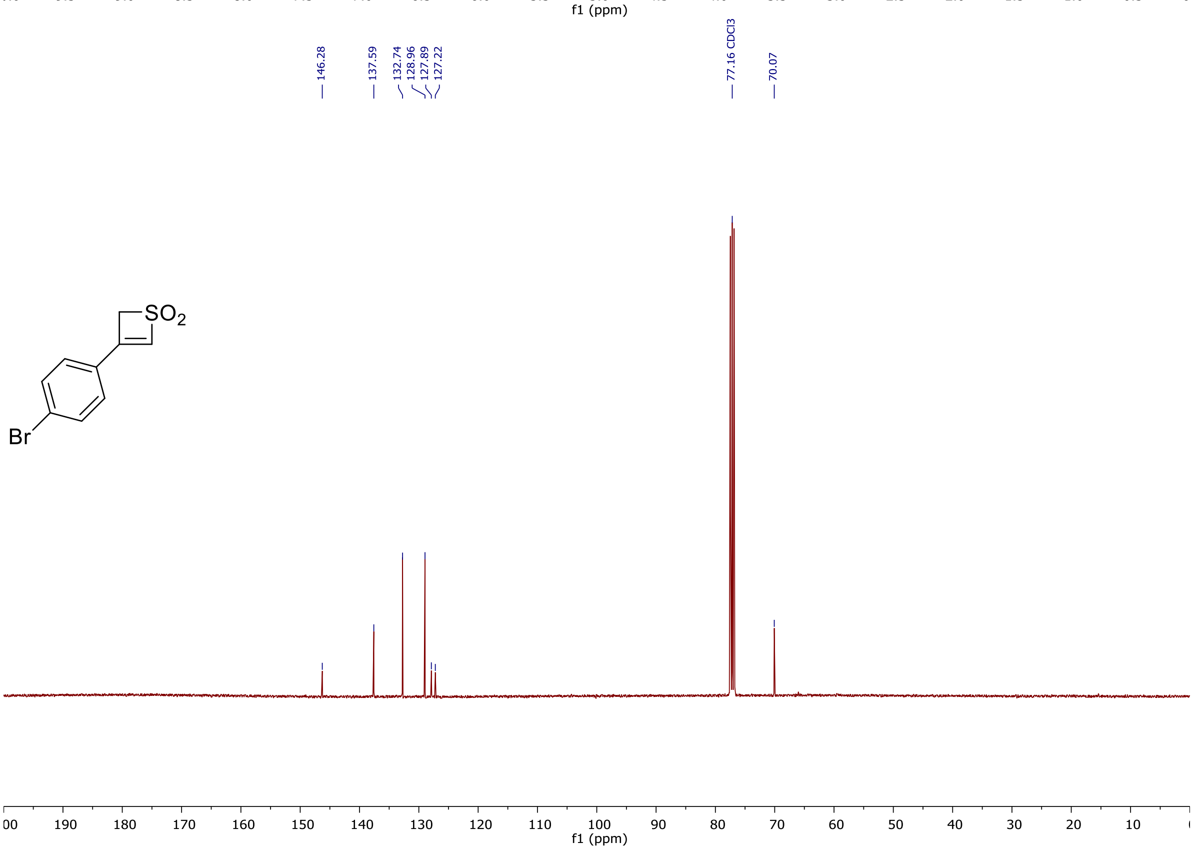

3,4-Di(naphthalen-1-yl)-2*H*-thiete 1,1-dioxide (3a)

^{1}H NMR (400 MHz, $CDCl_3$) and **^{13}C NMR** (101 MHz, $CDCl_3$)

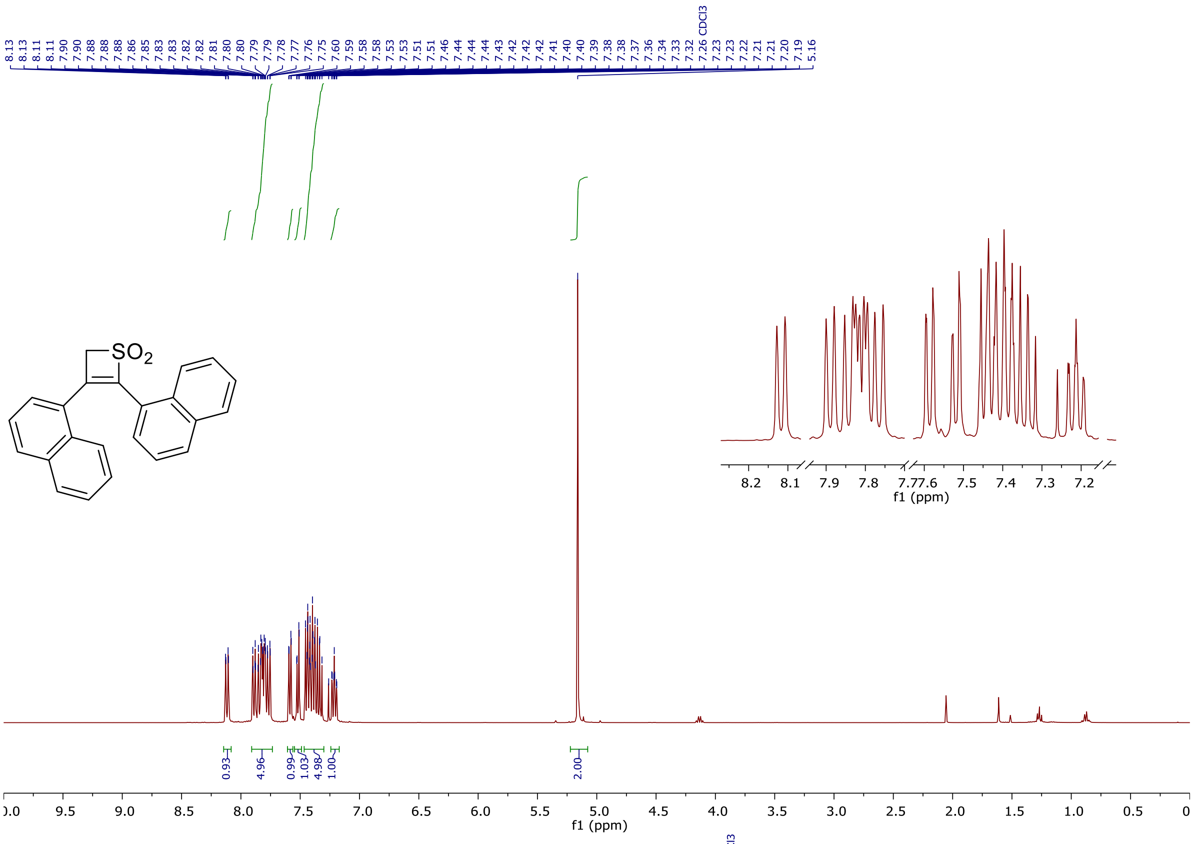

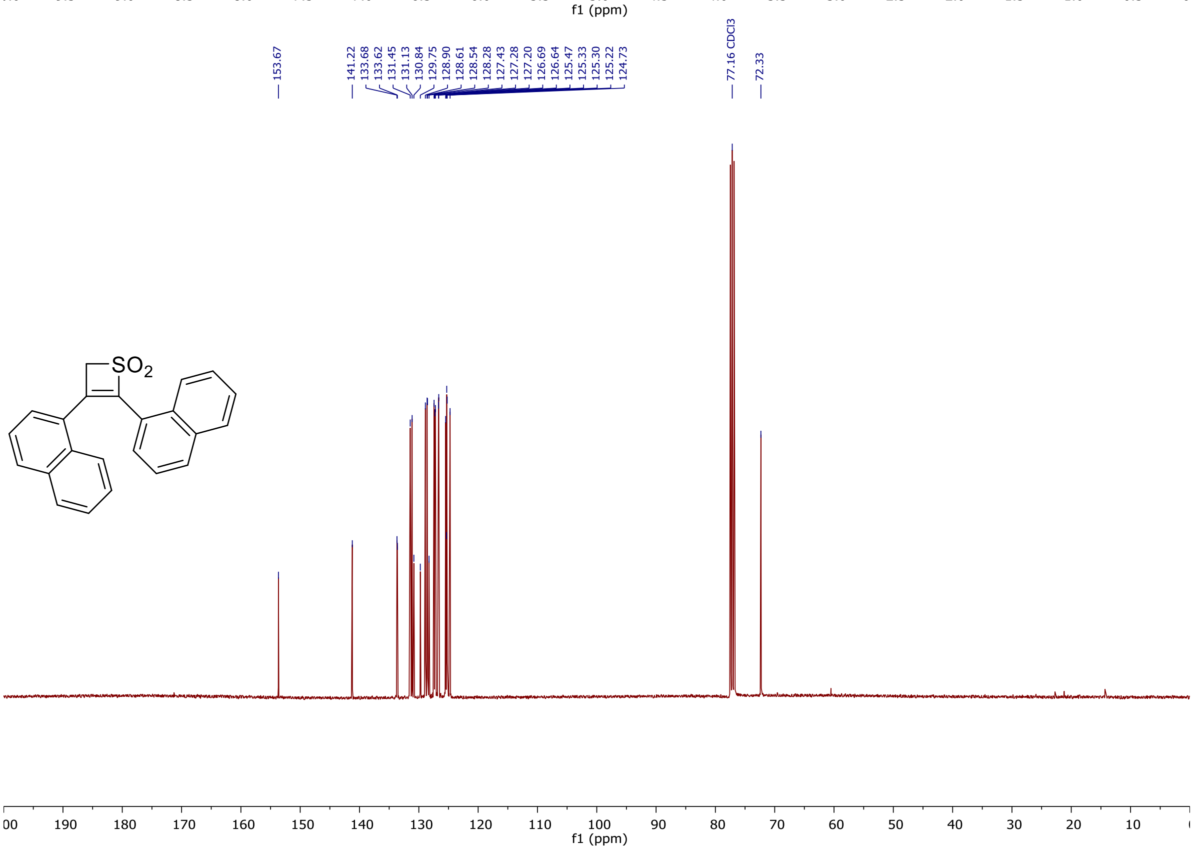

4-(Isoquinolin-4-yl)-3-(naphthalen-1-yl)-2*H*-thiete 1,1-dioxide (3b)

^{1}H NMR (400 MHz, $CDCl_3$) and **^{13}C NMR** (101 MHz, $CDCl_3$)

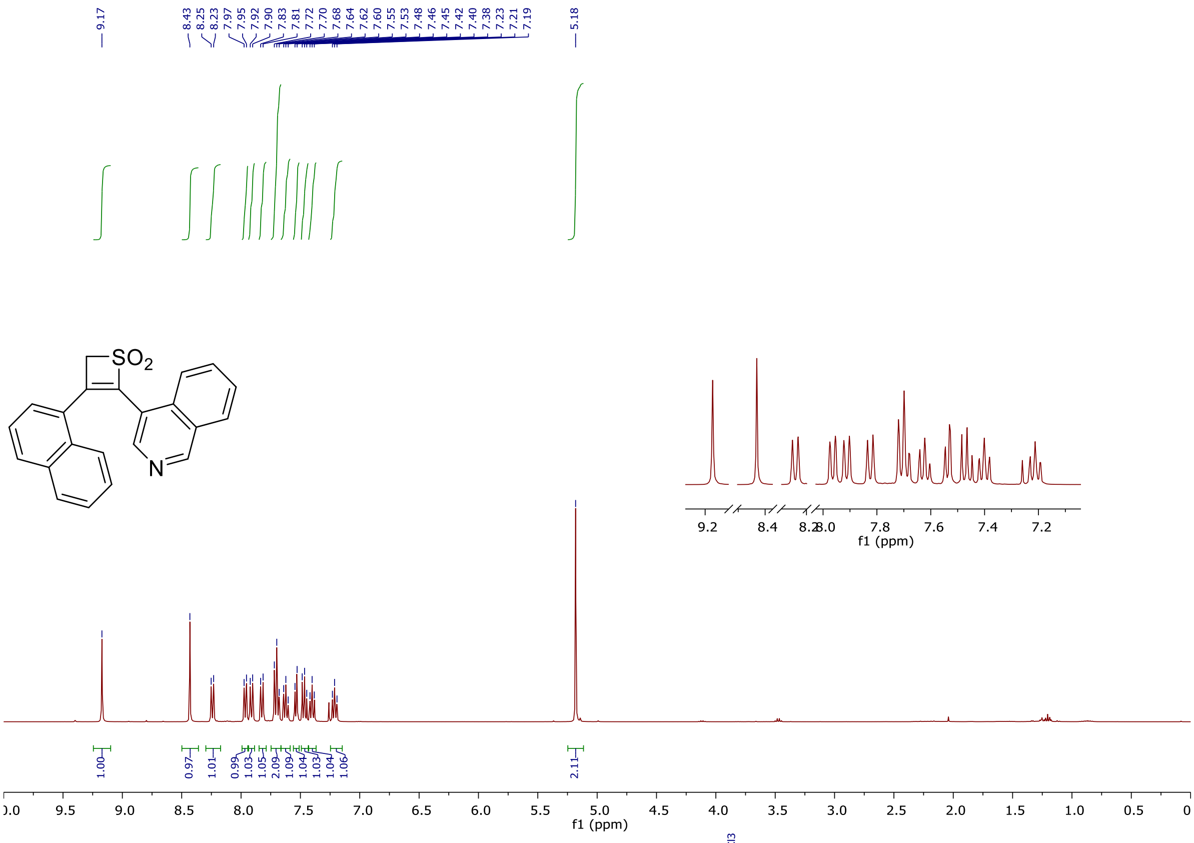

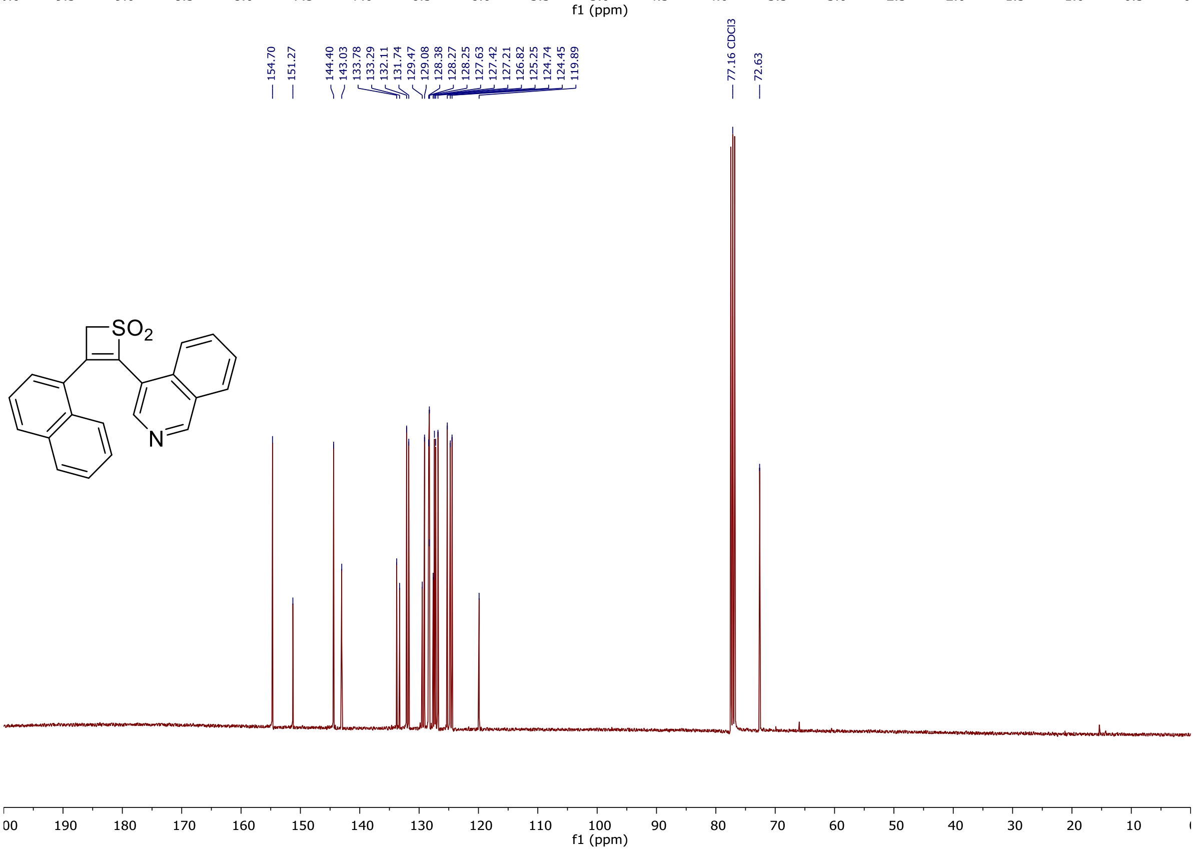

3-(Anthracen-9-yl)-4-(isoquinolin-4-yl)-2*H*-thiete 1,1-dioxide (3c)

^{1}H NMR (400 MHz, $CDCl_3$) and **^{13}C NMR** (101 MHz, $CDCl_3$)

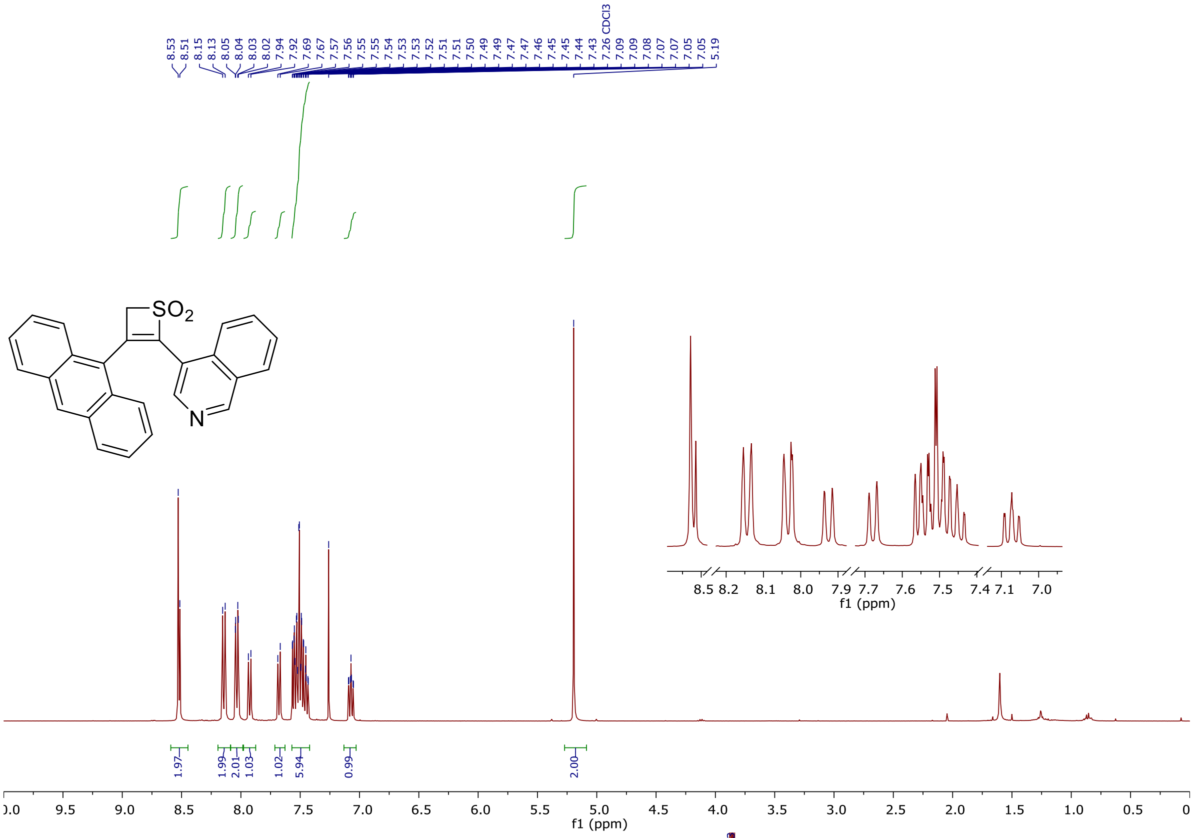

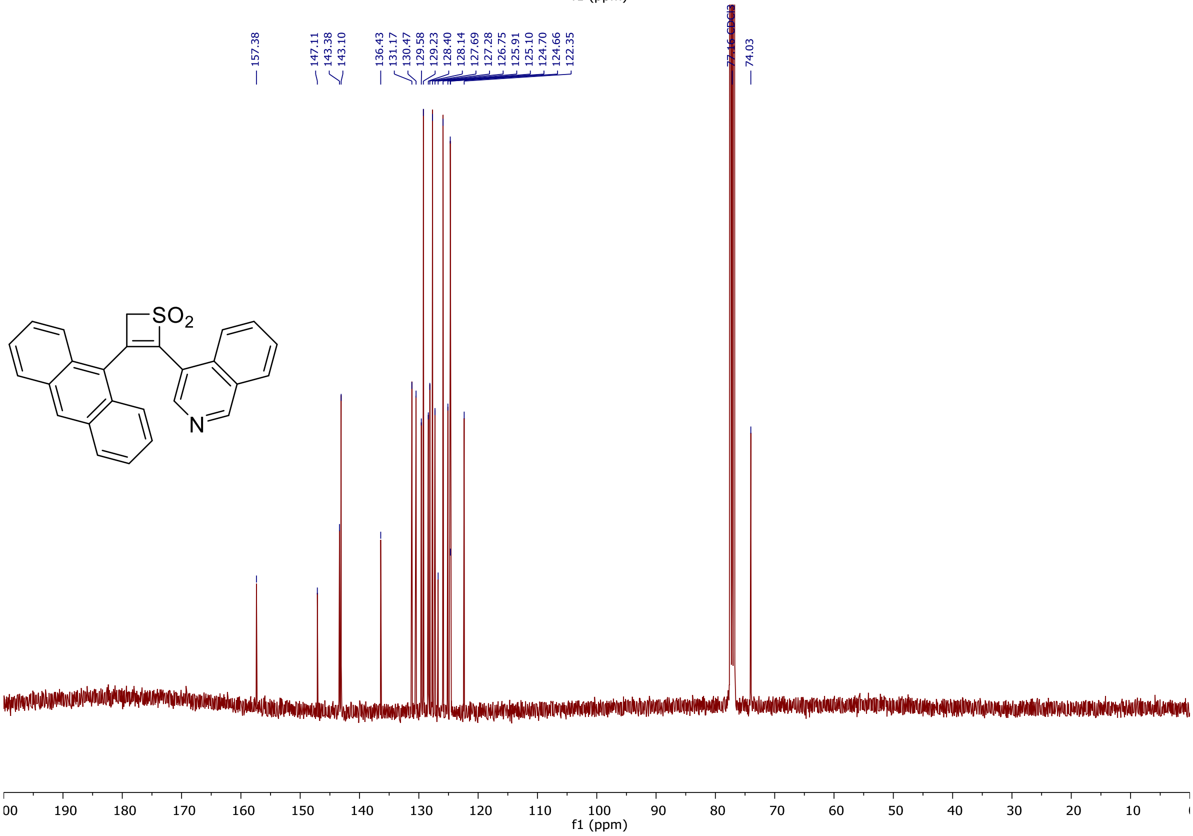

4-(Anthracen-9-yl)-3-(naphthalen-1-yl)-2*H*-thiete 1,1-dioxide (3d)

^{1}H NMR (400 MHz, $CDCl_3$) and **^{13}C NMR** (101 MHz, $CDCl_3$)

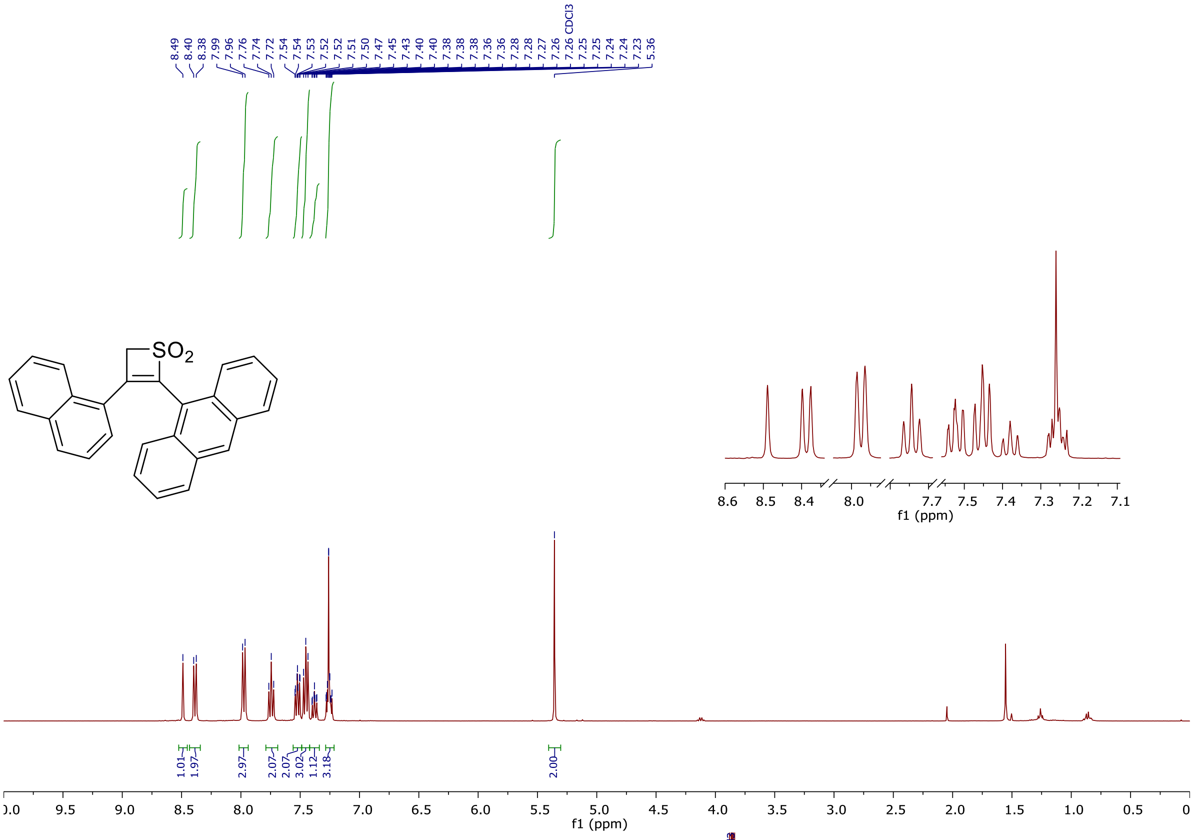

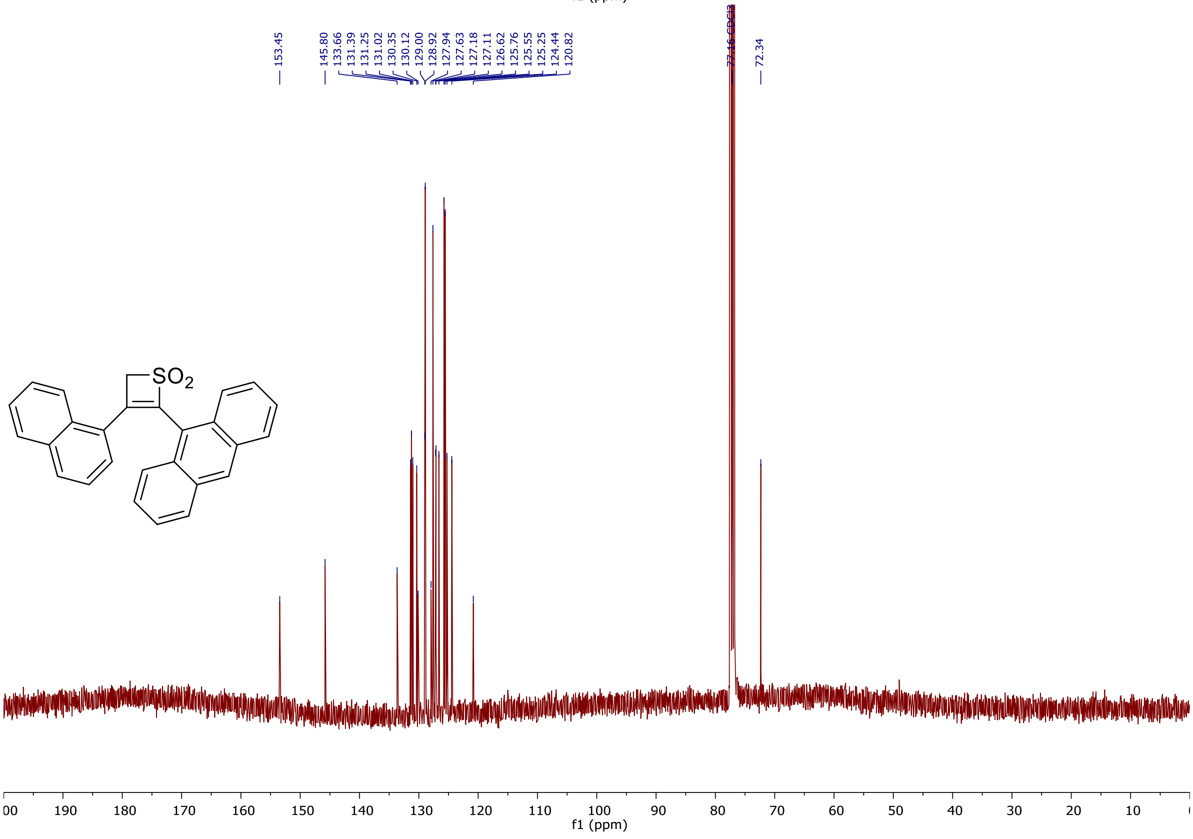

4-(Naphthalen-1-yl)-4-(4-phenylpyridin-3-yl)-2*H*-thiete 1,1-dioxide (4a)

^{1}H NMR (400 MHz, $CDCl_3$) and **^{13}C NMR** (101 MHz, $CDCl_3$)

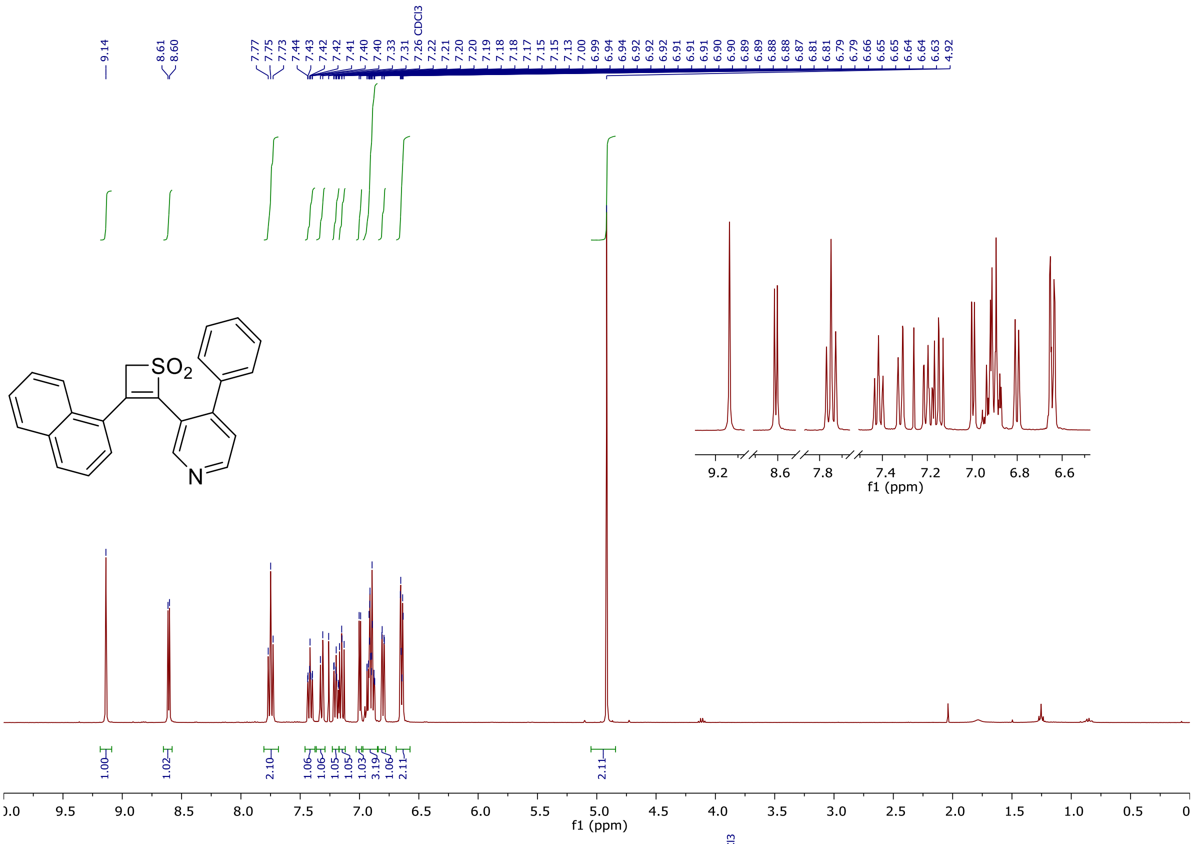

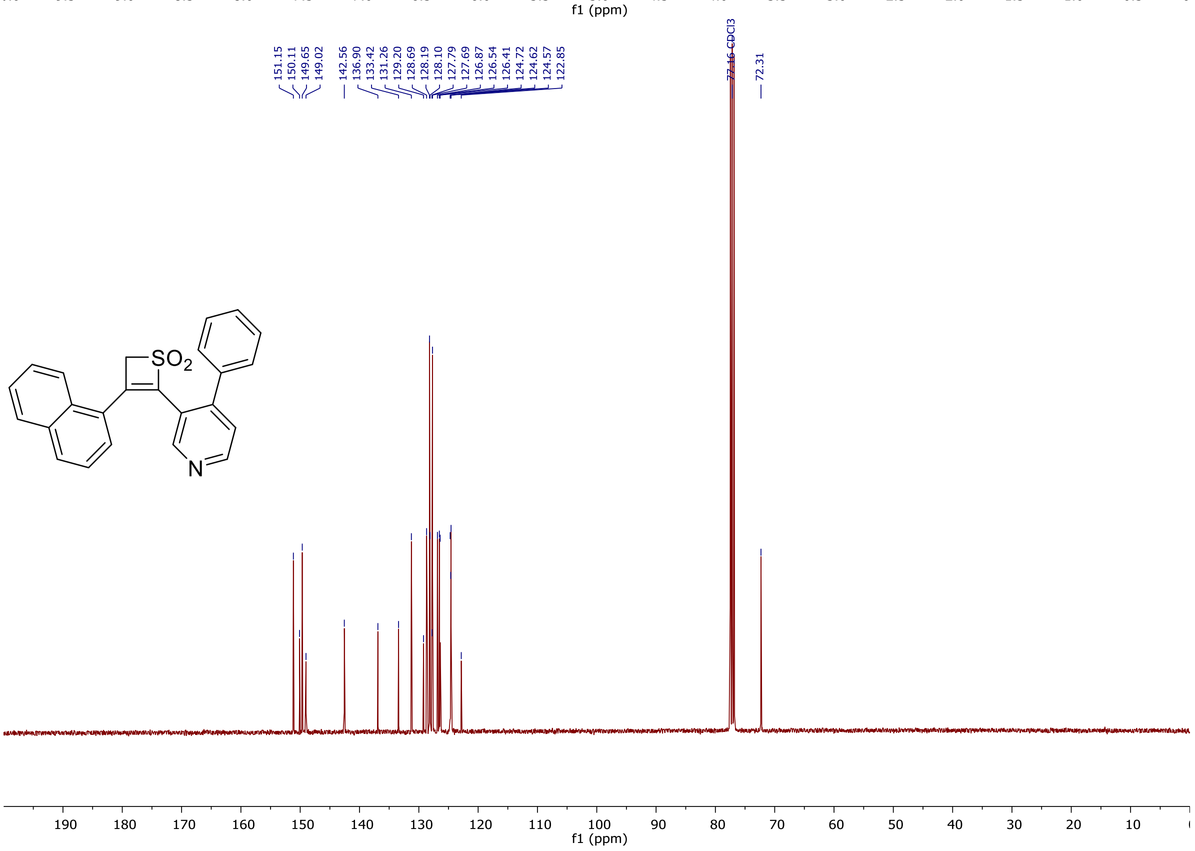

(2-(3-(Naphthalen-1-yl)-1,1-dioxido-2*H*-thiet-4-yl)phenyl)(phenyl)methanone (4b)

^{1}H NMR (400 MHz, $CDCl_3$) and **^{13}C NMR** (101 MHz, $CDCl_3$)

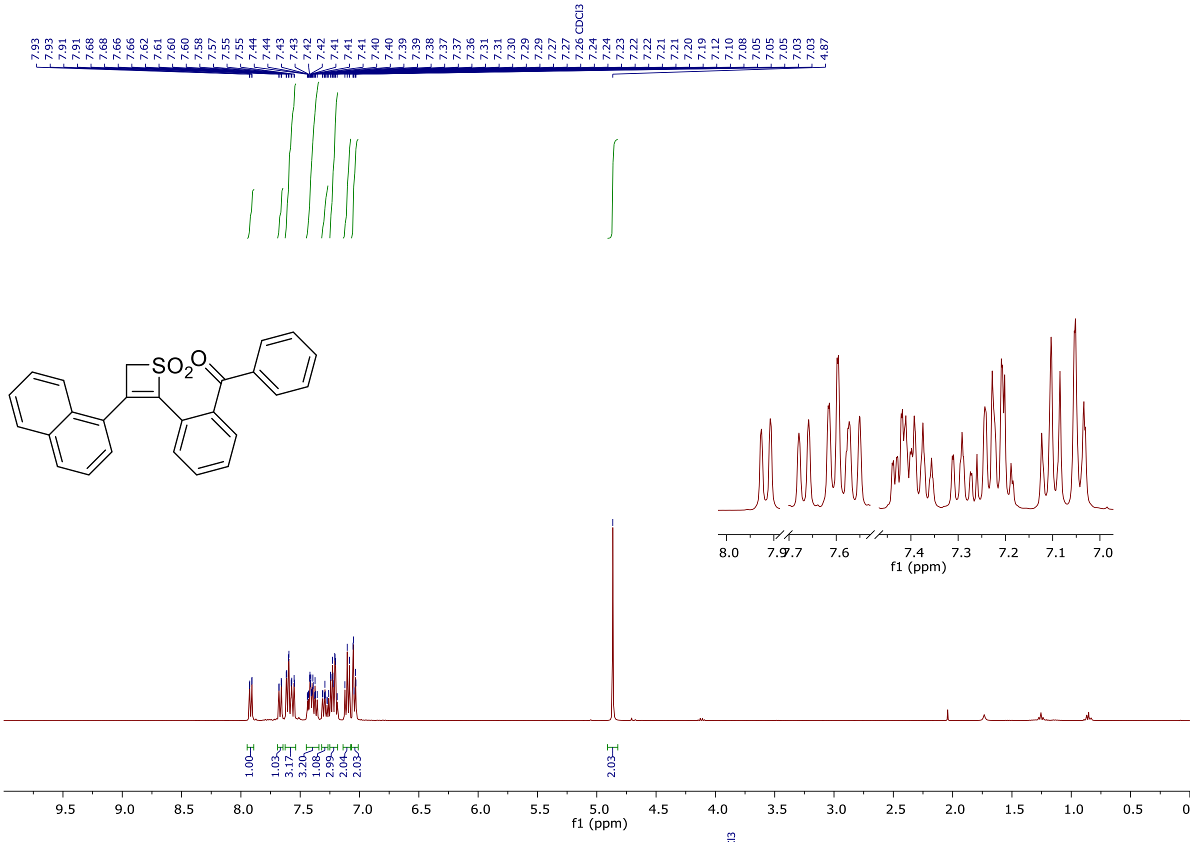

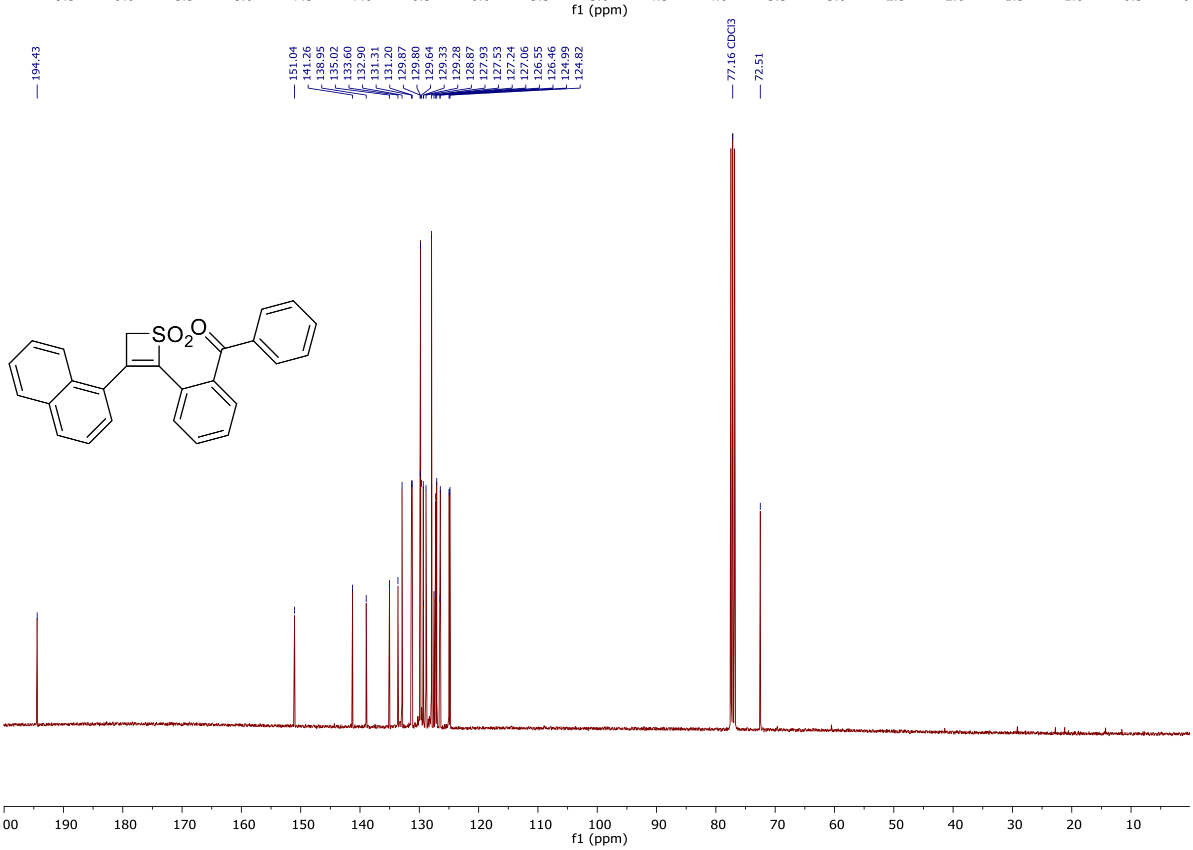

(2-(3-Mesityl-1,1-dioxido-2*H*-thiet-4-yl)phenyl)(phenyl)methanone (4c)

^{1}H NMR (400 MHz, $CDCl_3$) and **^{13}C NMR** (101 MHz, $CDCl_3$)

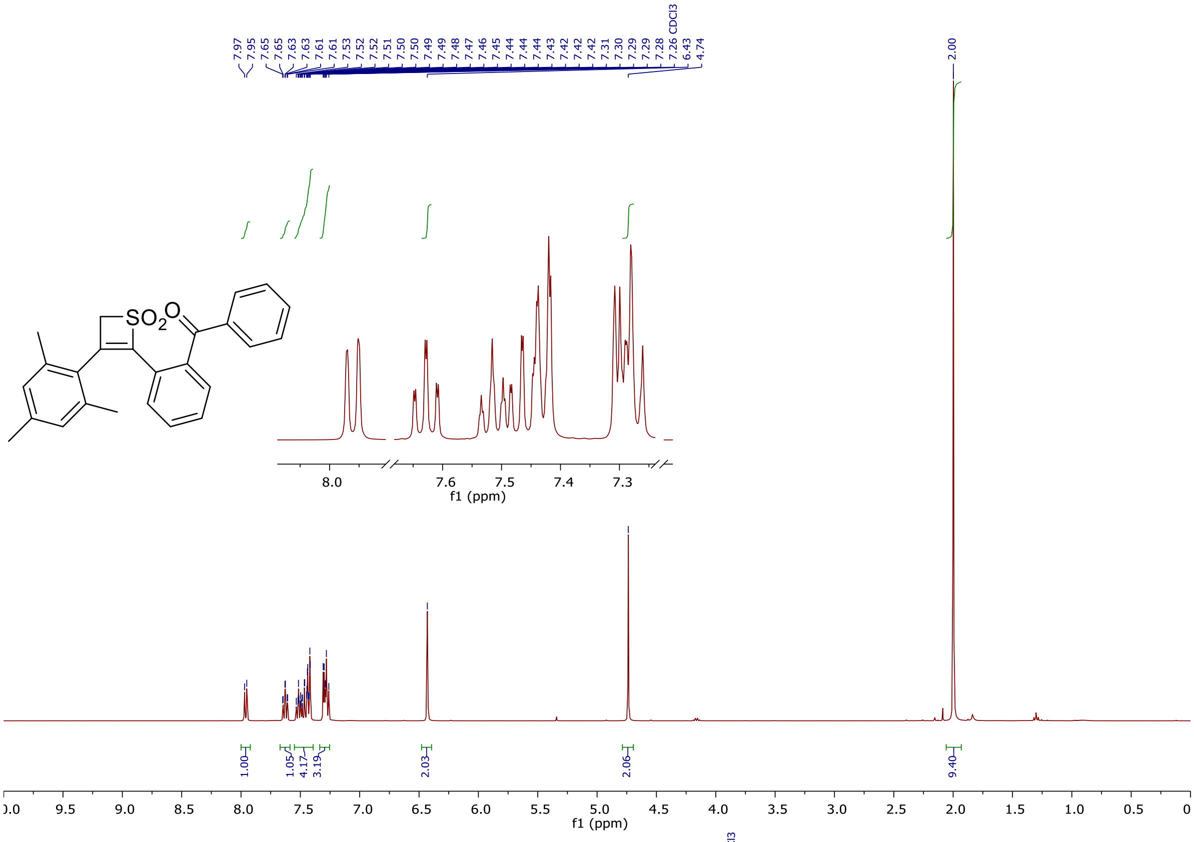

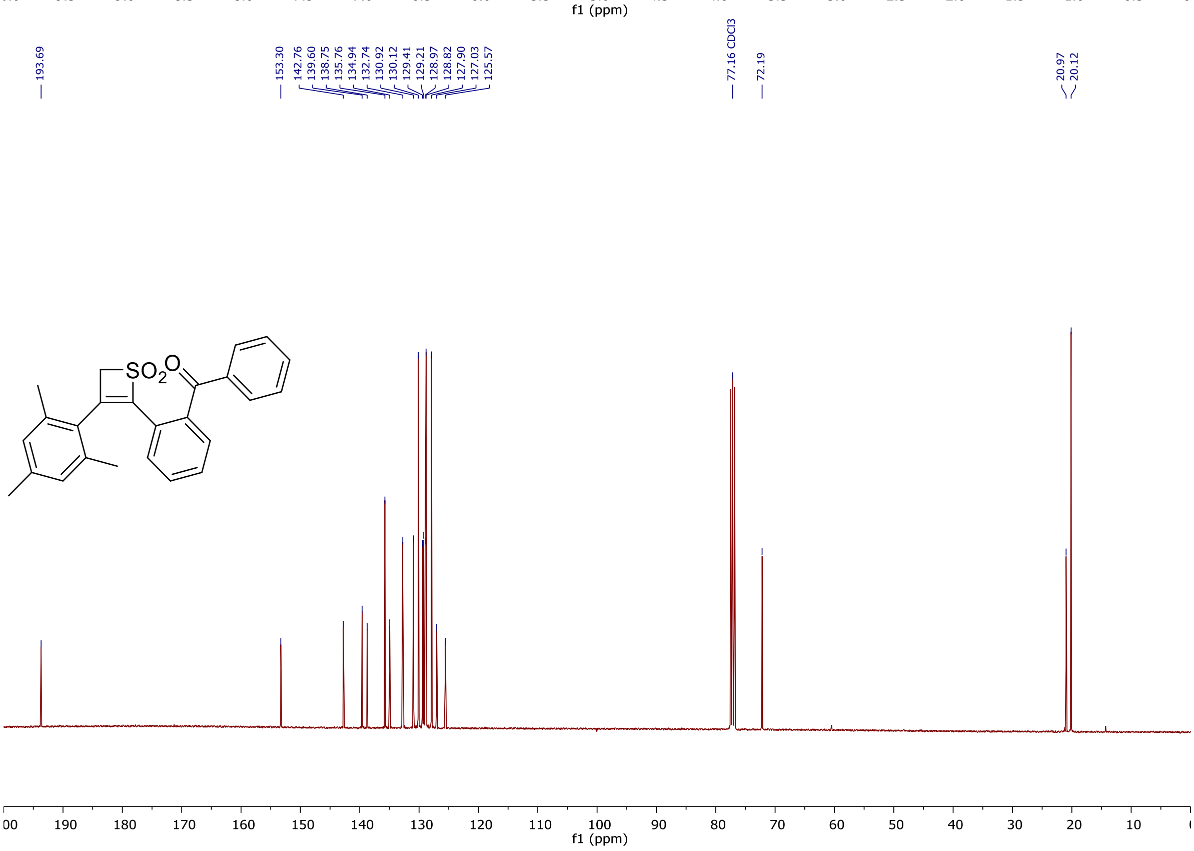

3-Mesityl-4-(4-phenylpyridin-3-yl)-2*H*-thiete 1,1-dioxide (4d)

^{1}H NMR (400 MHz, $CDCl_3$) and **^{13}C NMR** (101 MHz, $CDCl_3$)

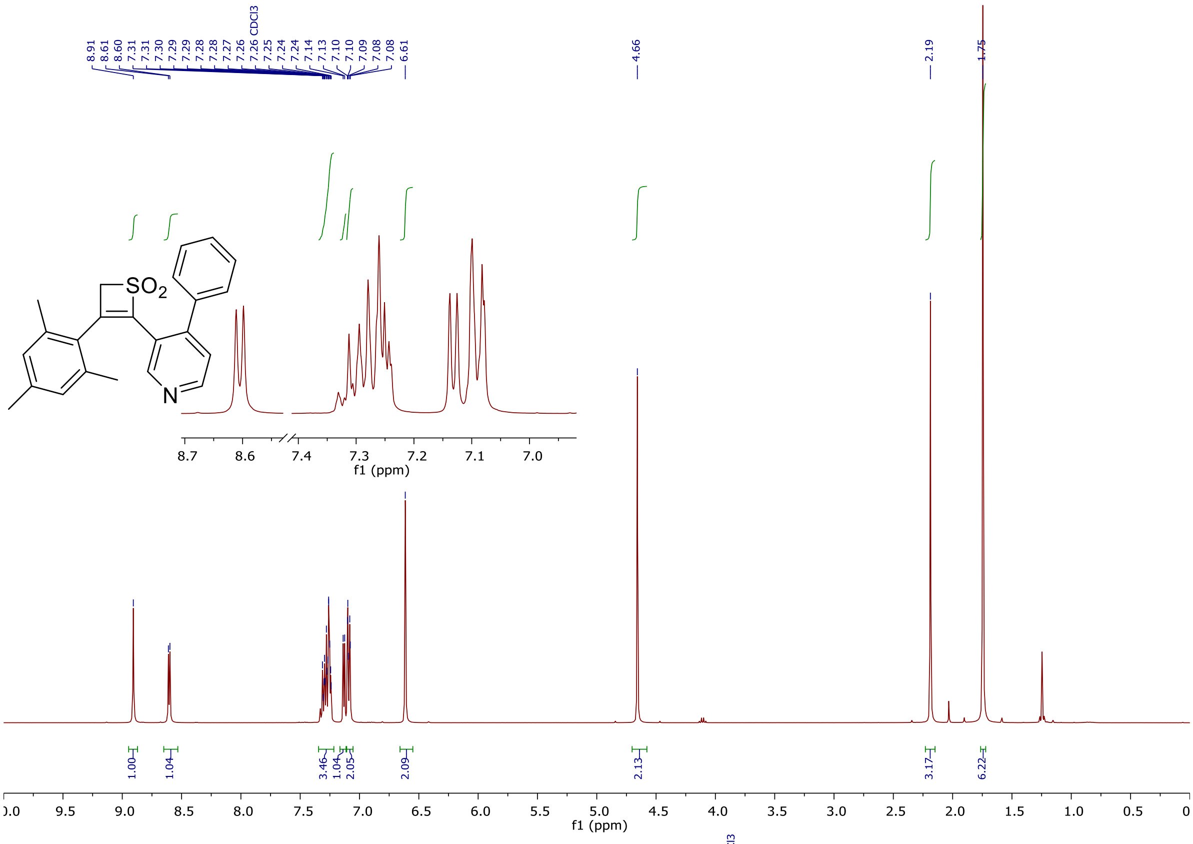

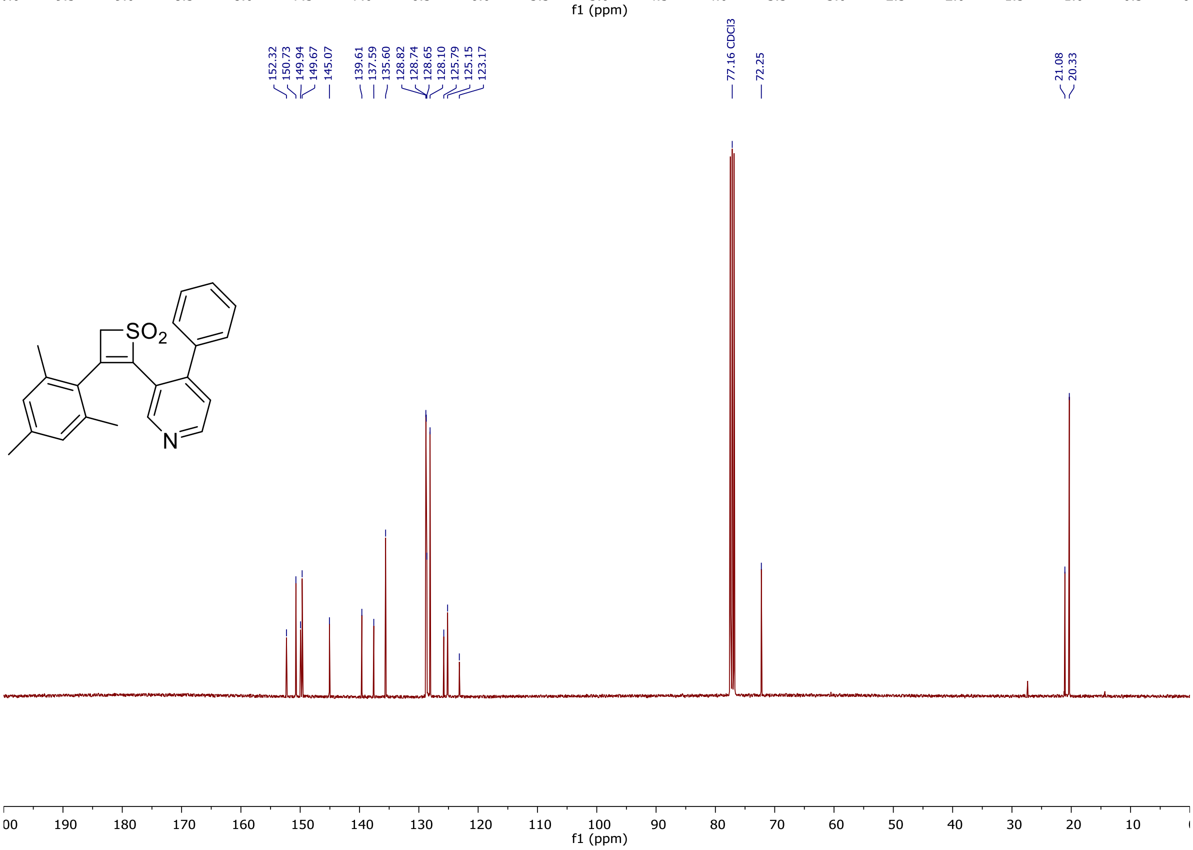

3-Phenyl-4-(4-phenylpyridin-3-yl)-2*H*-thiete 1,1-dioxide (4e)

^{1}H NMR (400 MHz, $CDCl_3$) and **^{13}C NMR** (101 MHz, $CDCl_3$)

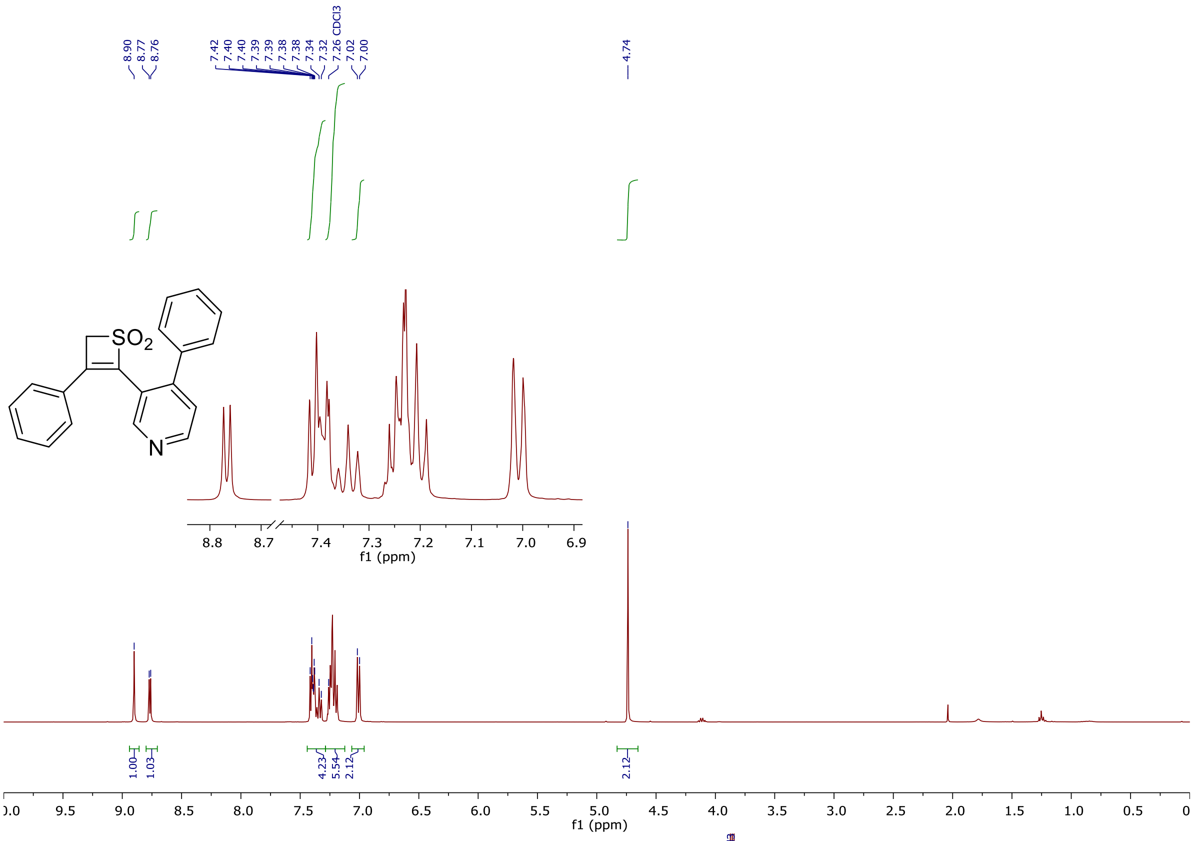

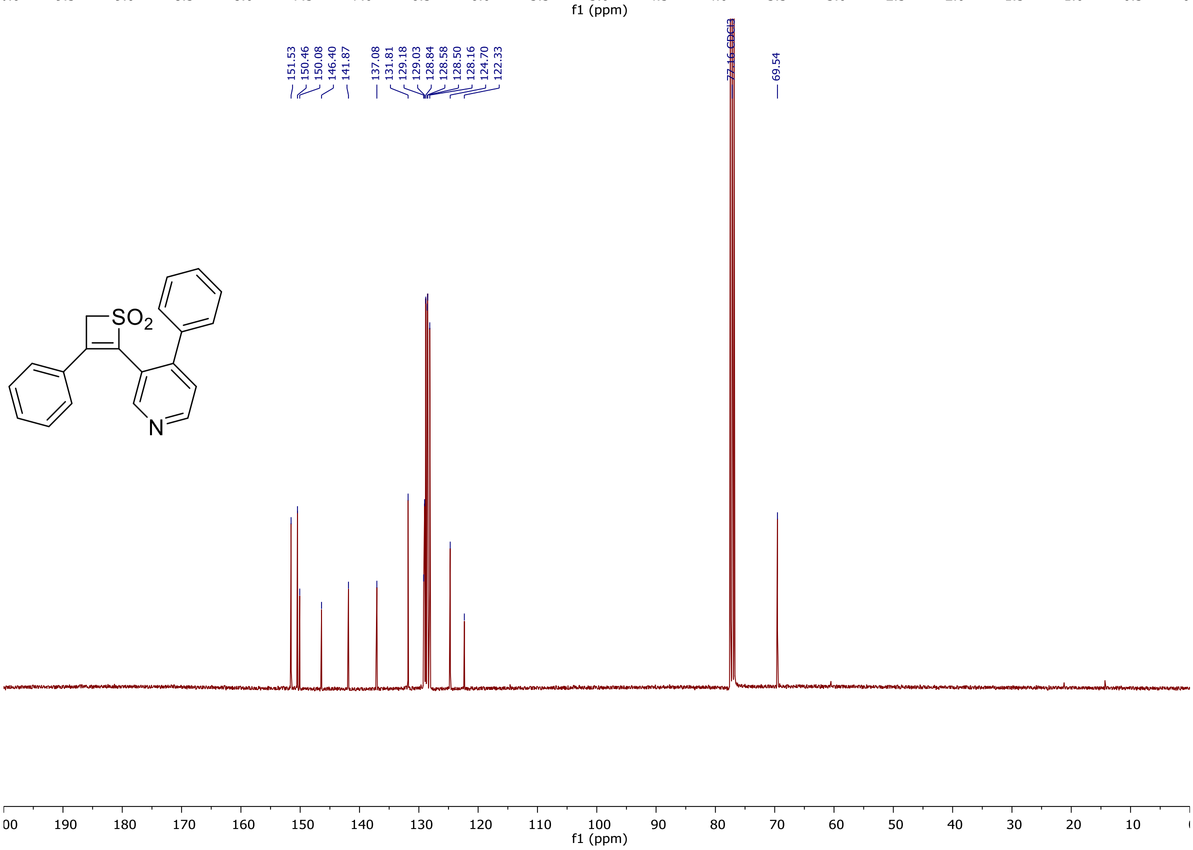

4-(4-Phenylpyridin-3-yl)-3-(3,4,5-trimethoxyphenyl)-2*H*-thiete 1,1-dioxide (4f)

^{1}H NMR (400 MHz, $CDCl_3$) and **^{13}C NMR** (101 MHz, $CDCl_3$)

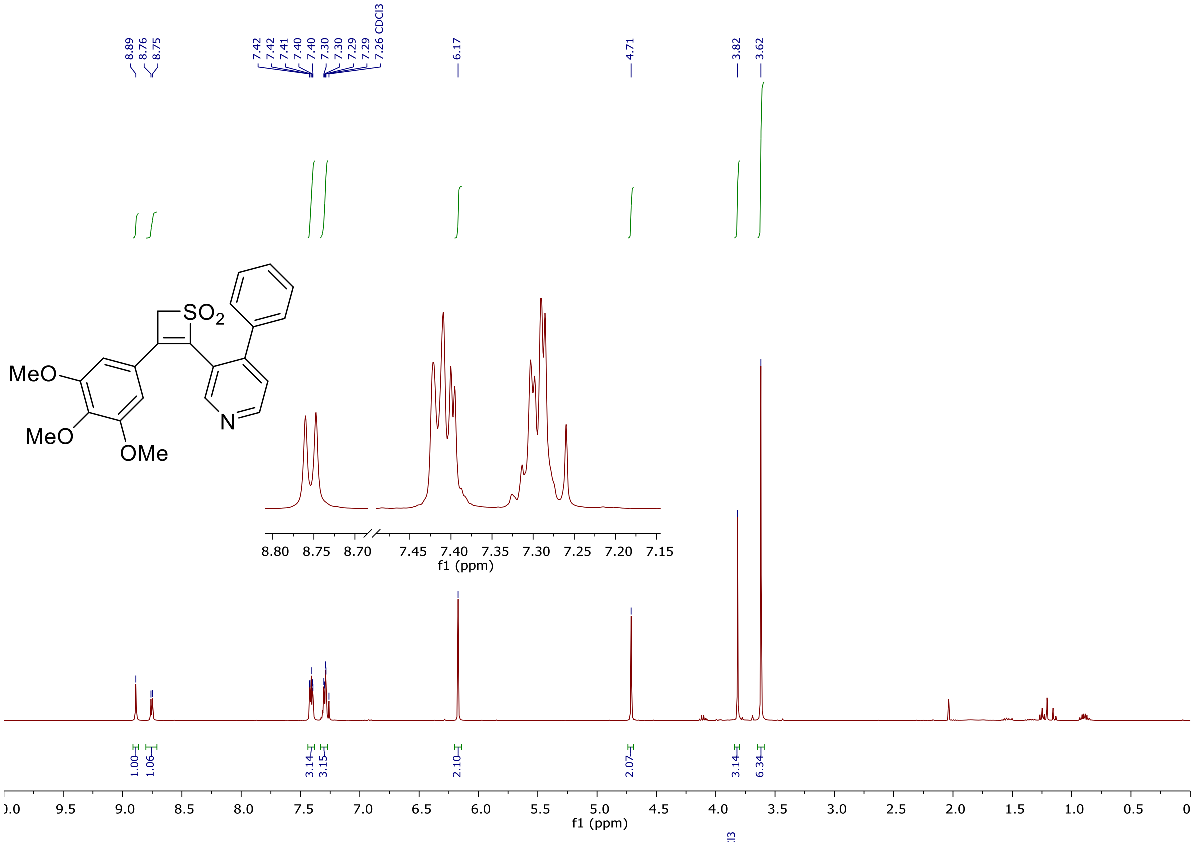

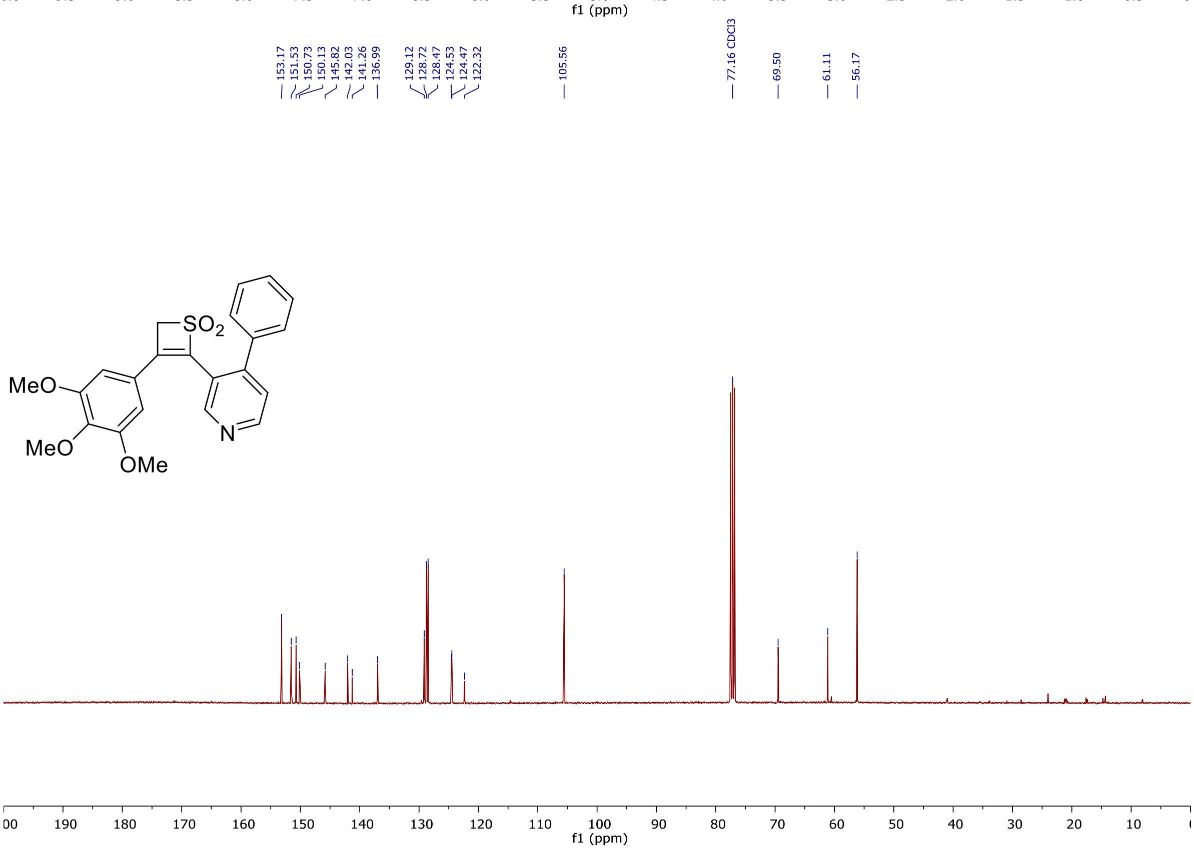

3-(4-Methoxyphenyl)-4-(4-phenylpyridin-3-yl)-2*H*-thiete 1,1-dioxide (4g)

^{1}**H NMR** (400 MHz, $CDCl_3$) and ^{13}**C NMR** (101 MHz, $CDCl_3$)

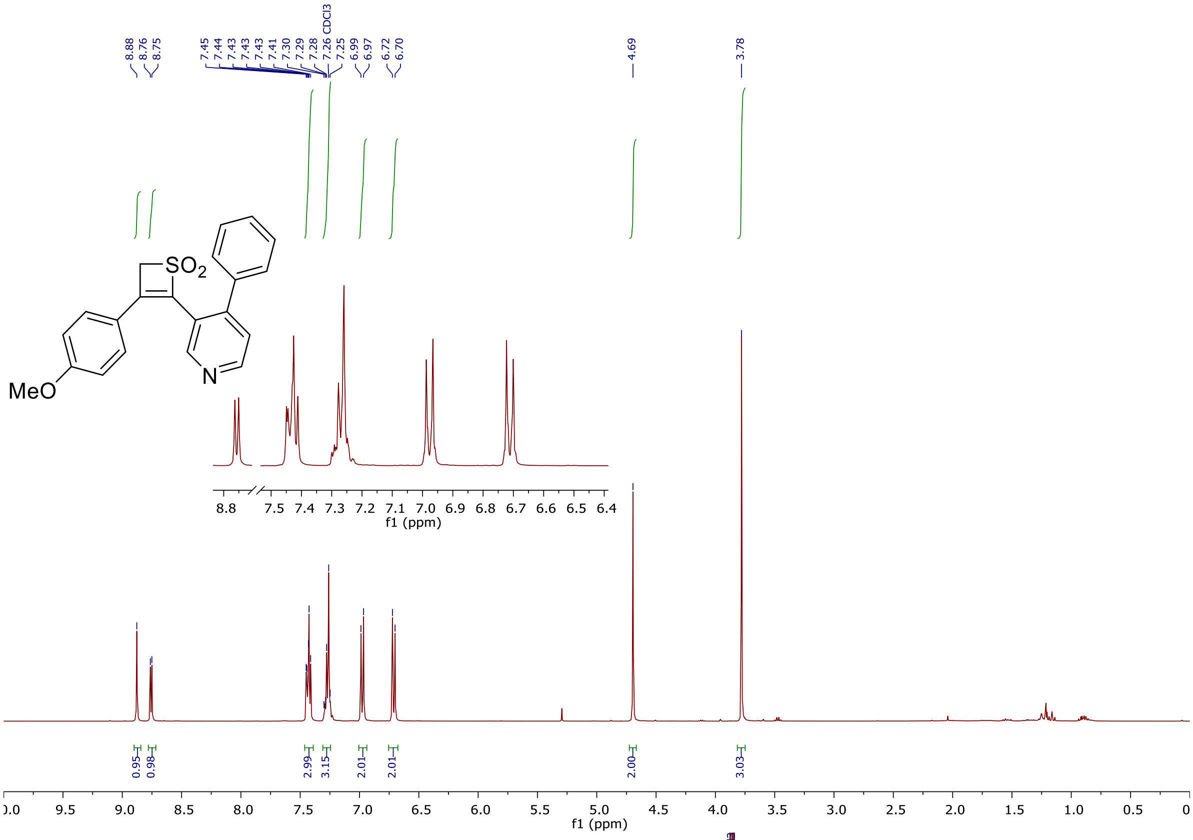

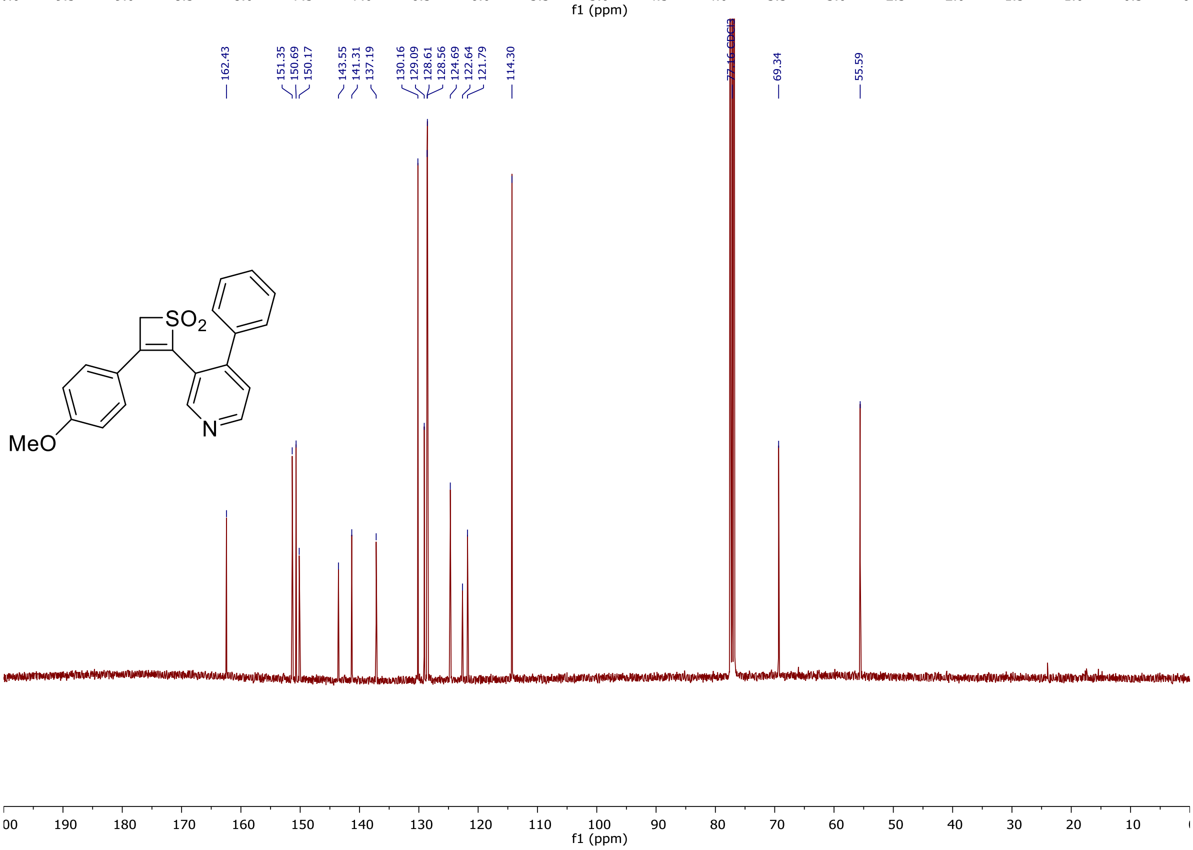

3-([1,1'-Biphenyl]-2-yl)-4-phenyl-2*H*-thiete 1,1-dioxide (5a)

^{1}H NMR (400 MHz, $CDCl_3$) and **^{13}C NMR** (101 MHz, $CDCl_3$)

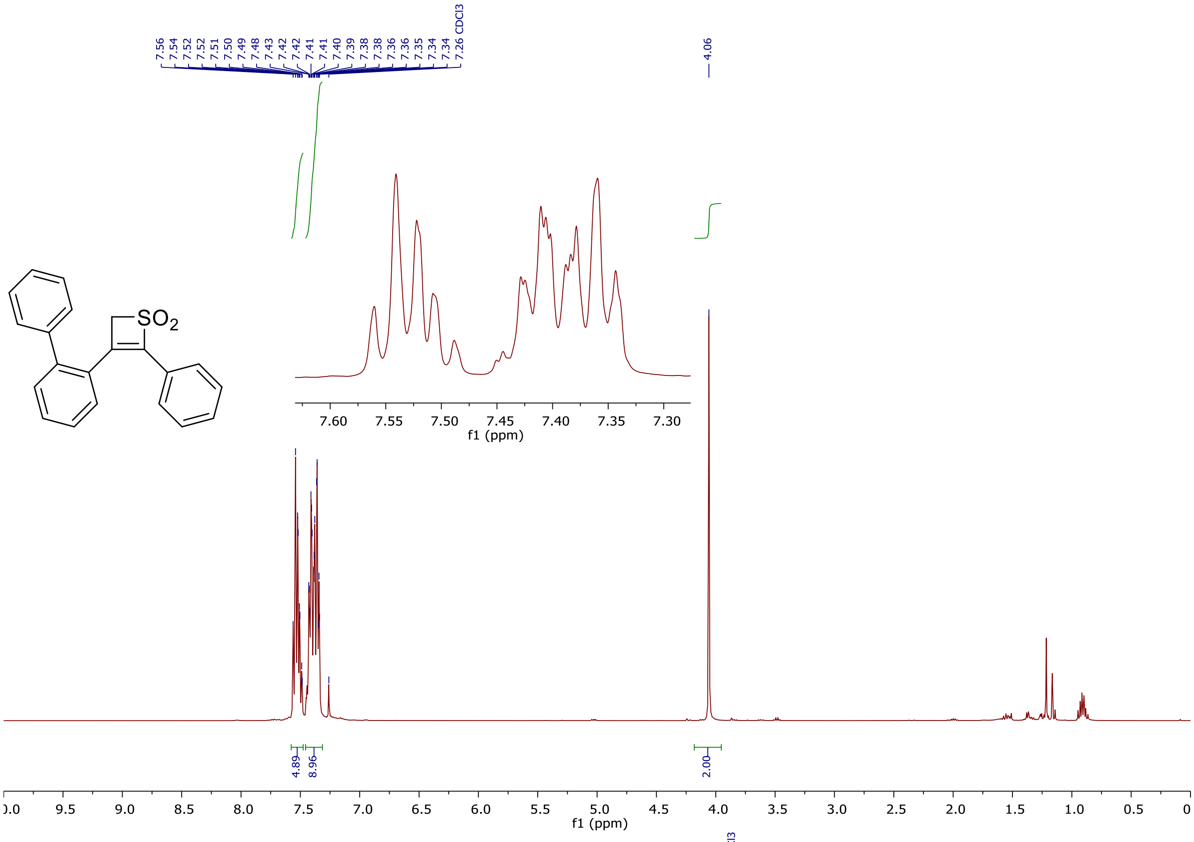

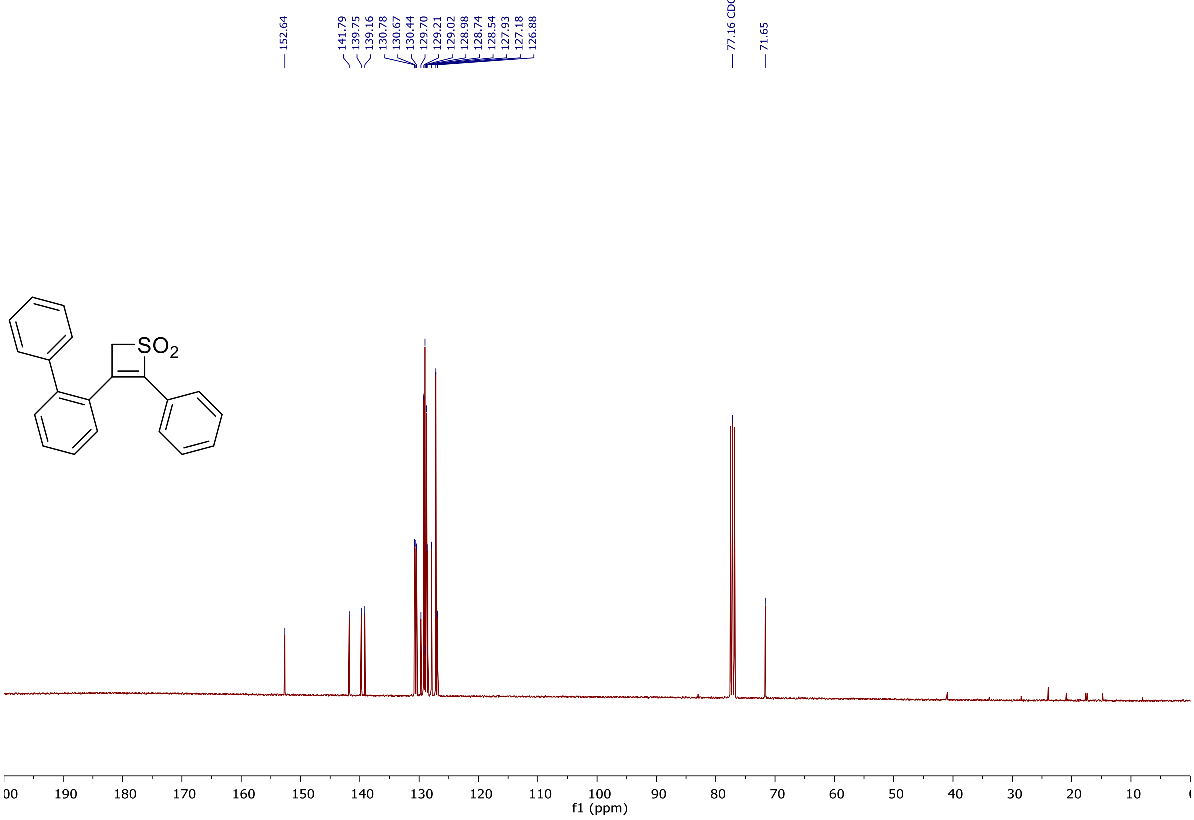

3-([1,1'-Biphenyl]-2-yl)-4-(4-methoxyphenyl)-2*H*-thiete 1,1-dioxide (5b)

^{1}H NMR (400 MHz, $CDCl_3$) and **^{13}C NMR** (101 MHz, $CDCl_3$)

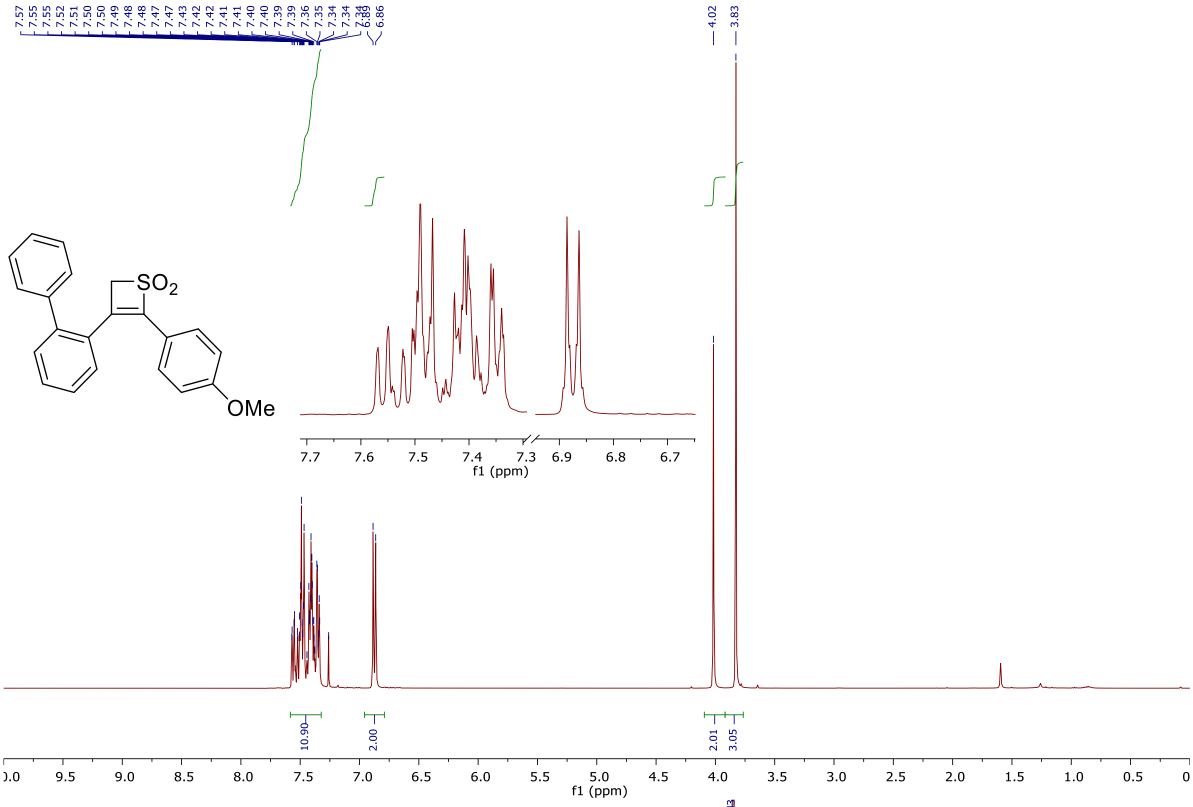

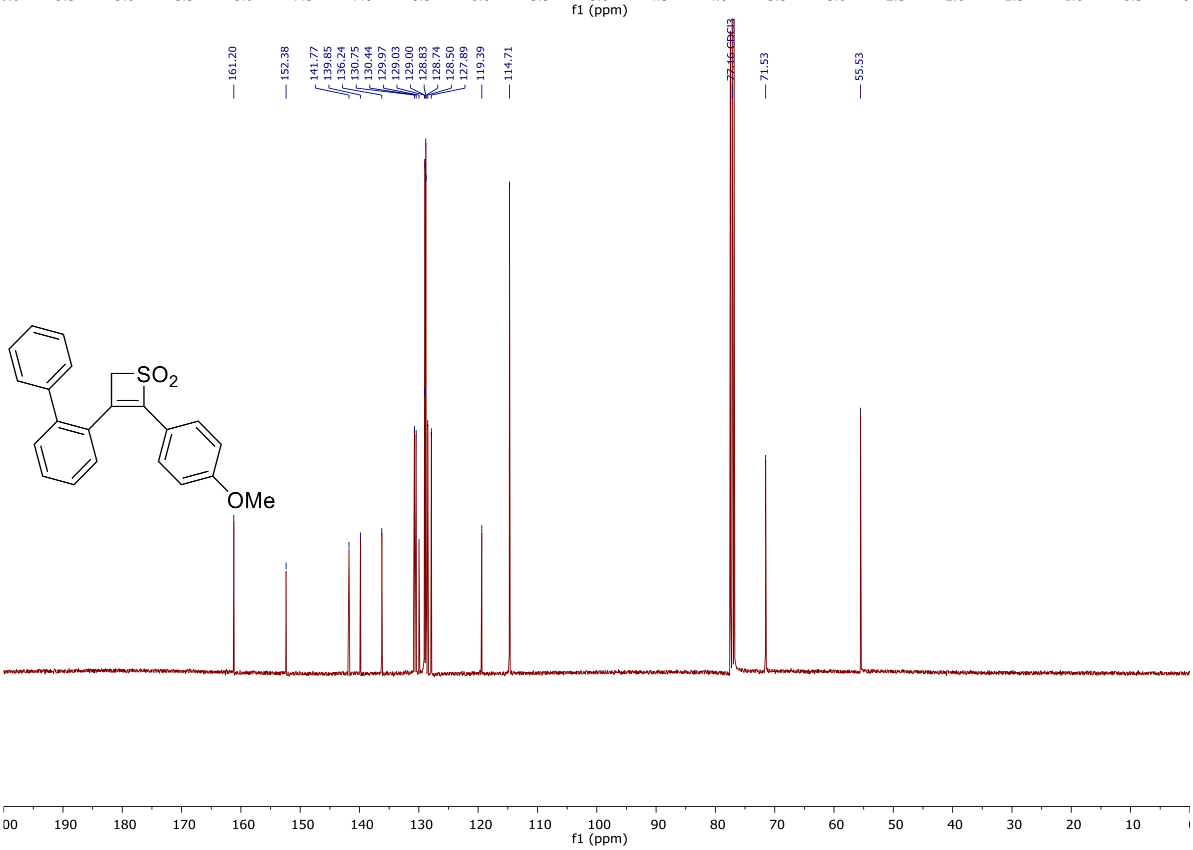

(*E*)-3-([1,1'-Biphenyl]-2-yl)-4-(4-(phenyldiazenyl)phenyl)-2*H*-thiete 1,1-dioxide (5c)

^{1}H NMR (400 MHz, $CDCl_3$) and **^{13}C NMR** (101 MHz, $CDCl_3$)

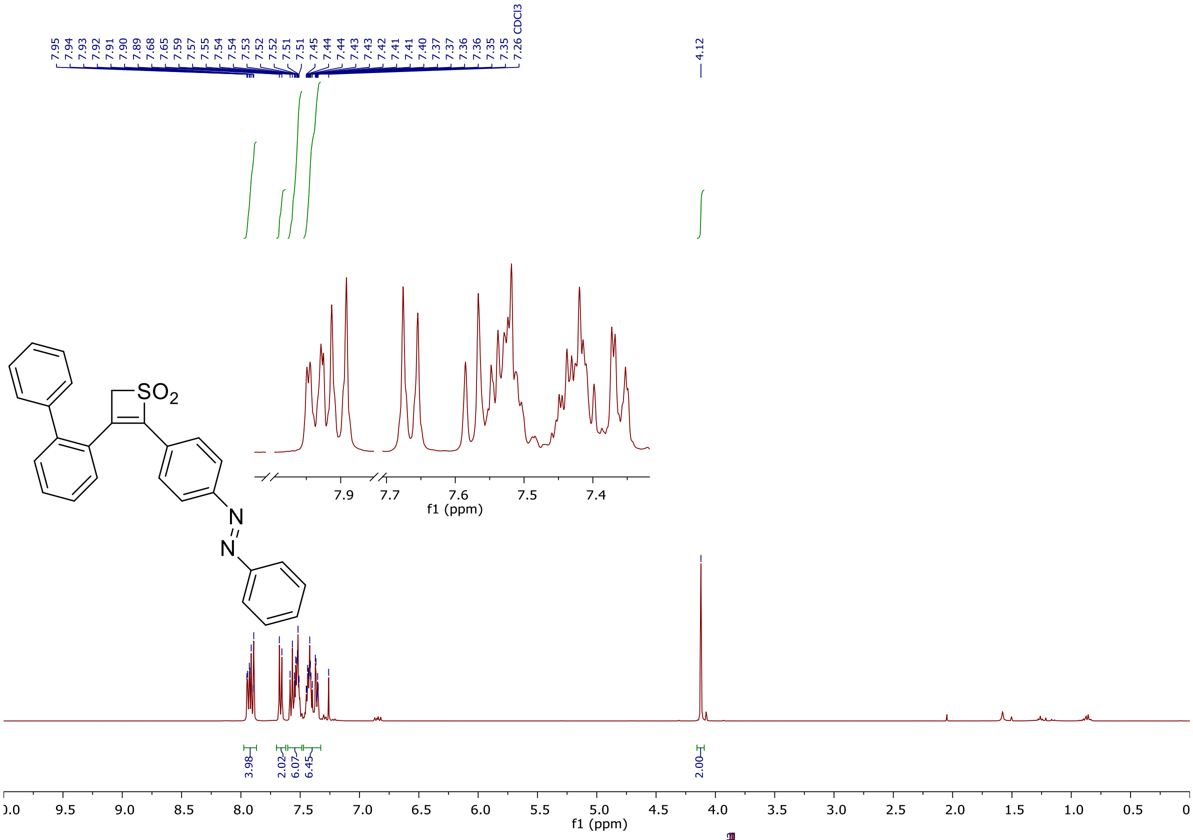

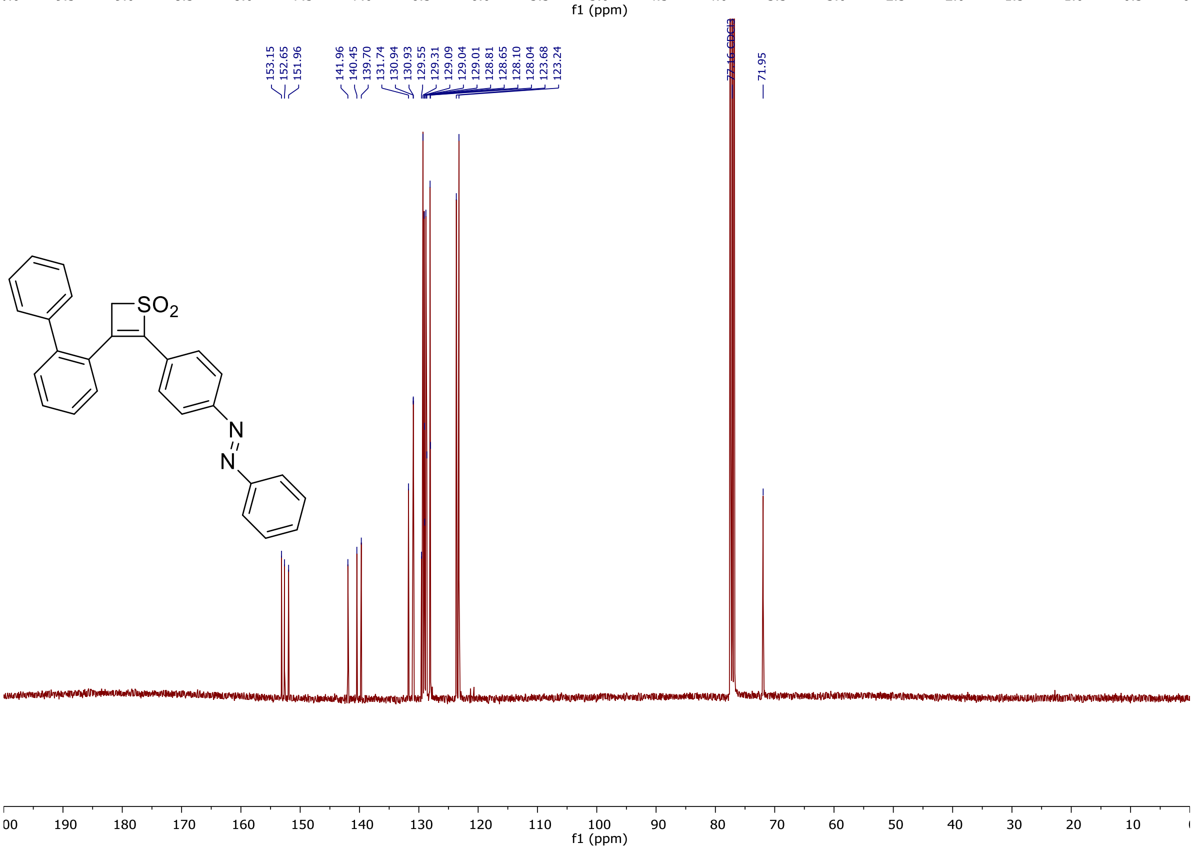

3-([1,1'-Biphenyl]-2-yl)-4-(4-nitrophenyl)-2*H*-thiete 1,1-dioxide (5d)

^{1}H NMR (400 MHz, $CDCl_3$) and **^{13}C NMR** (101 MHz, $CDCl_3$)

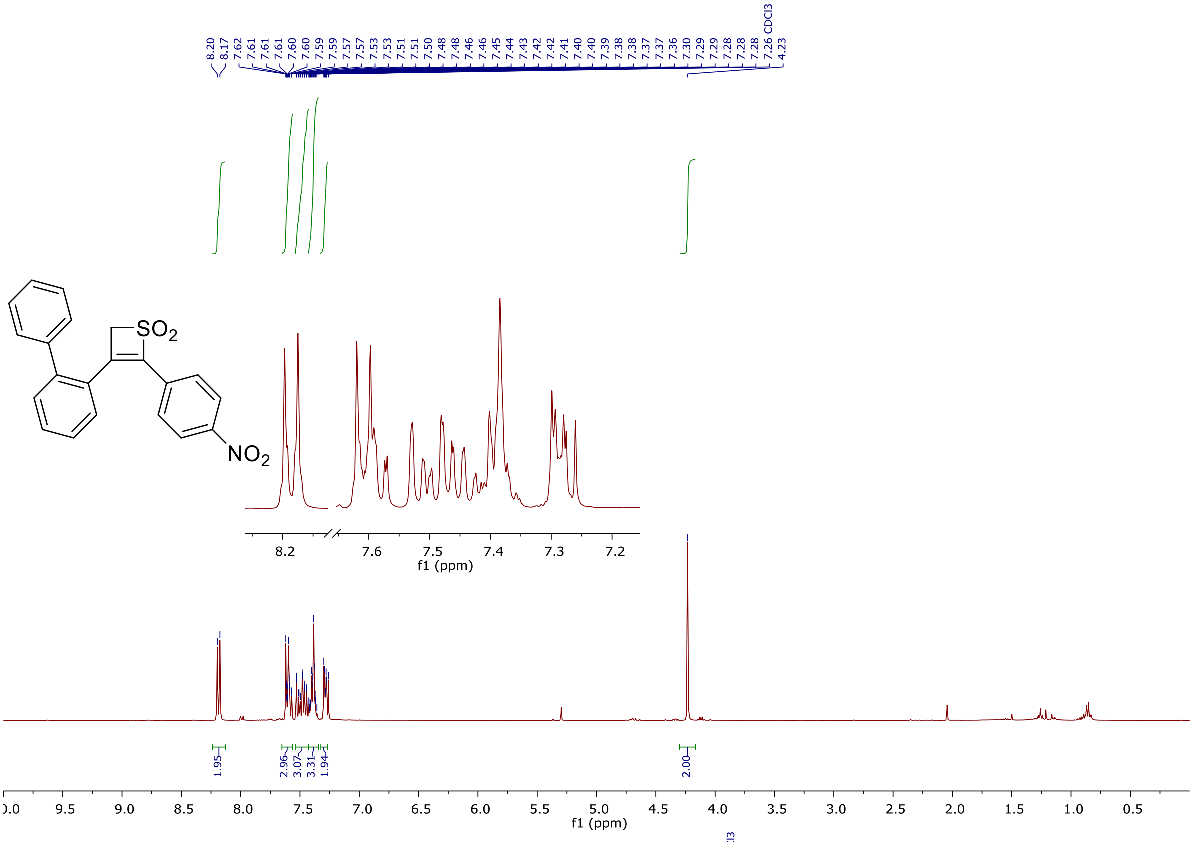

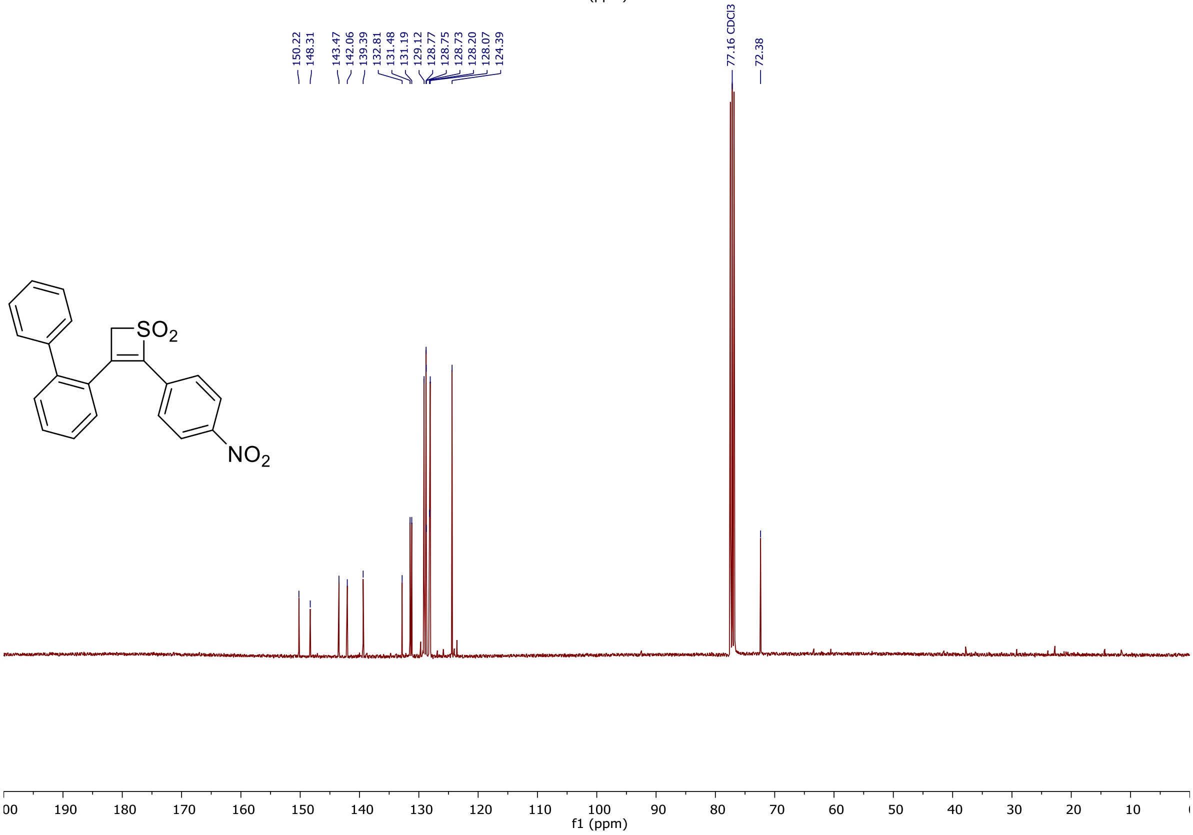

3-([1,1'-Biphenyl]-2-yl)-4-(pyridin-4-yl)-2*H*-thiete 1,1-dioxide (5e)

^{1}H NMR (400 MHz, CDCl$_3$) and **^{13}C NMR** (101 MHz, CDCl$_3$)

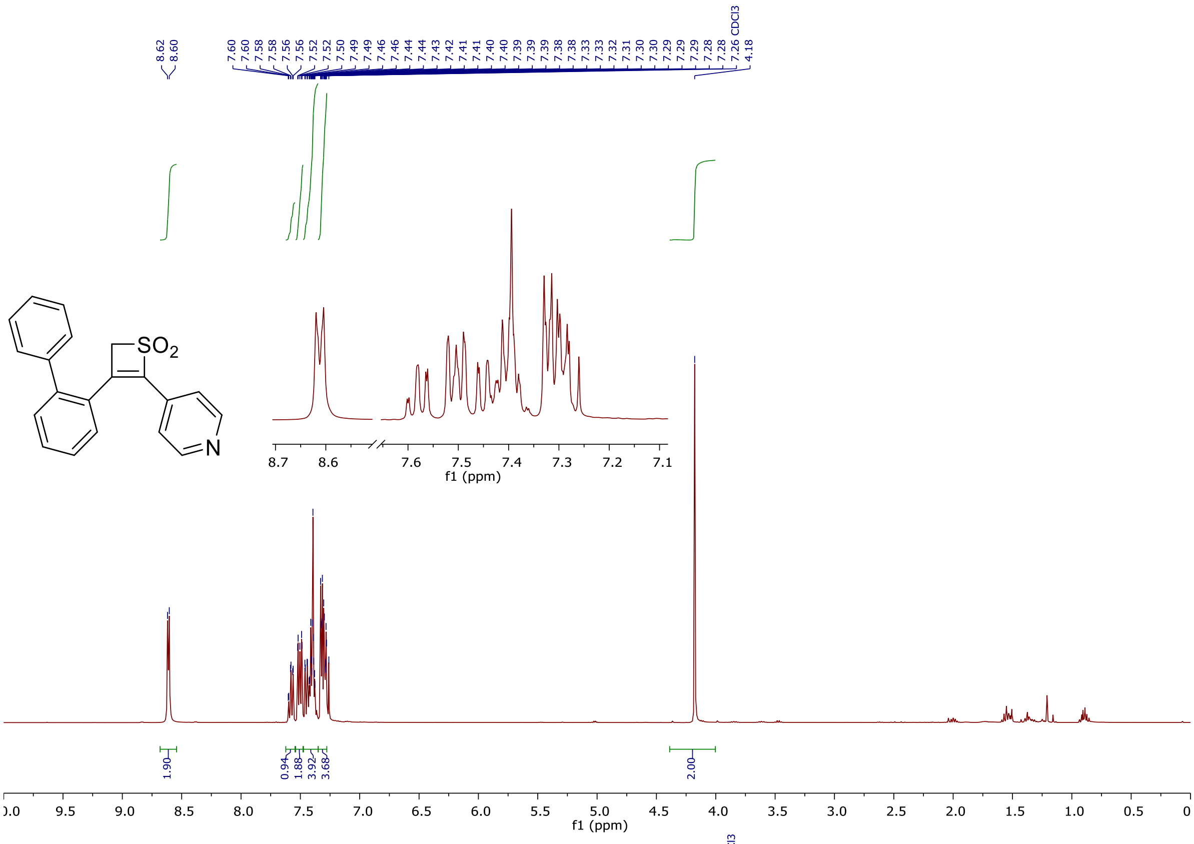

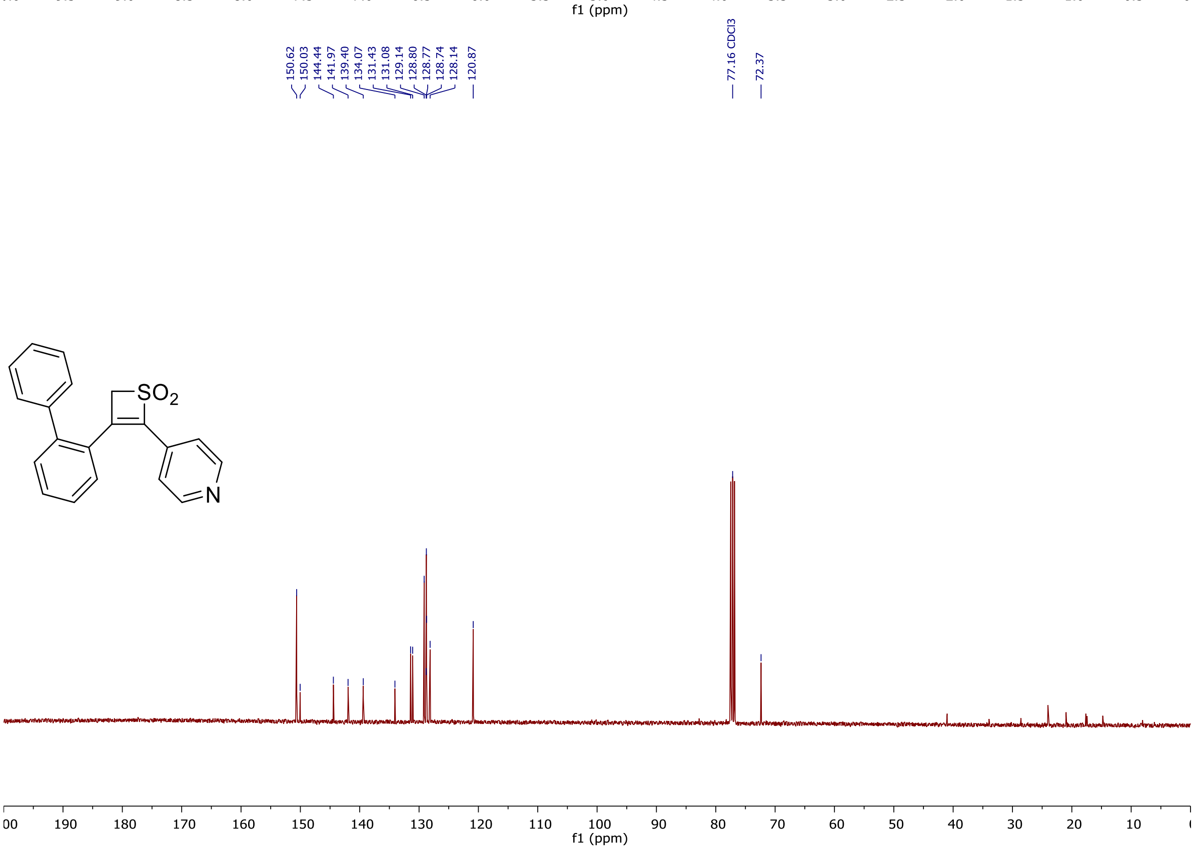

3-([1,1'-Biphenyl]-2-yl)-4-(pyrimidin-5-yl)-2*H*-thiete 1,1-dioxide (5f)

^{1}H NMR (400 MHz, CDCl$_3$) and **^{13}C NMR** (101 MHz, CDCl$_3$)

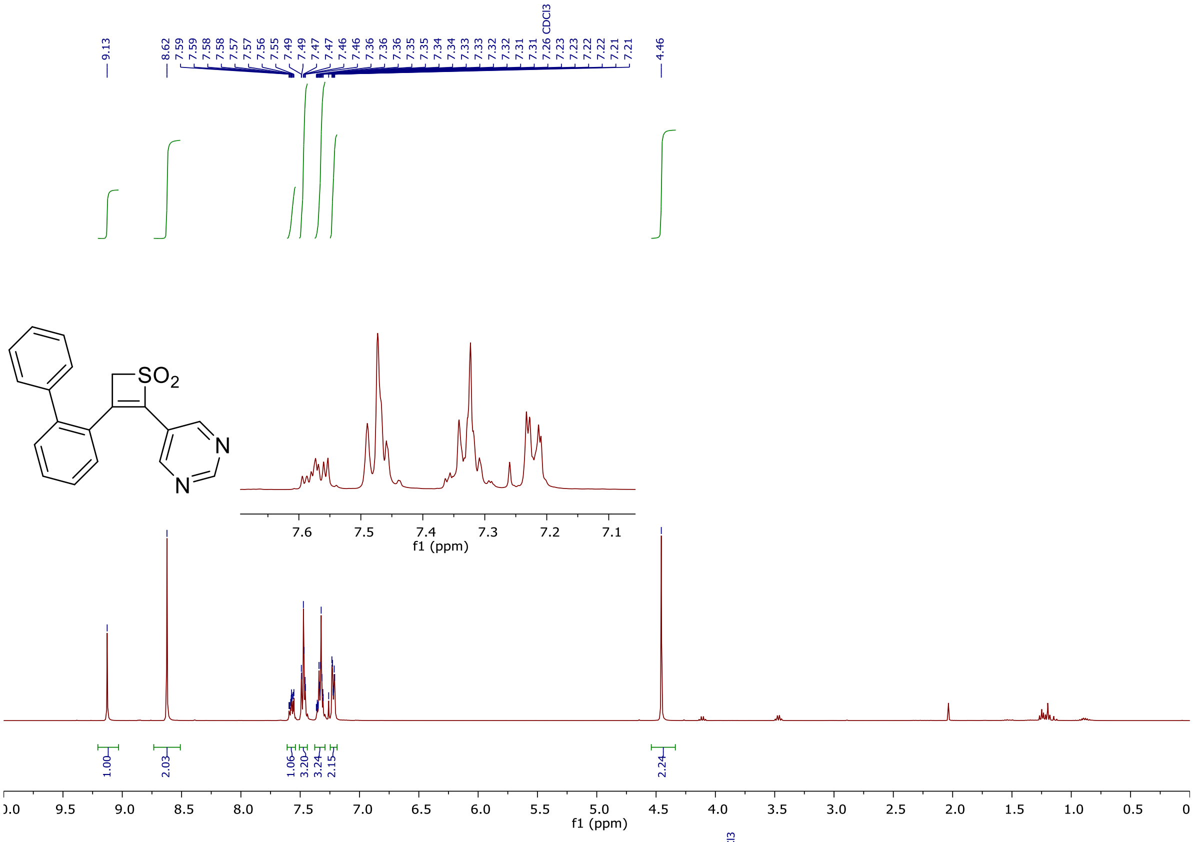

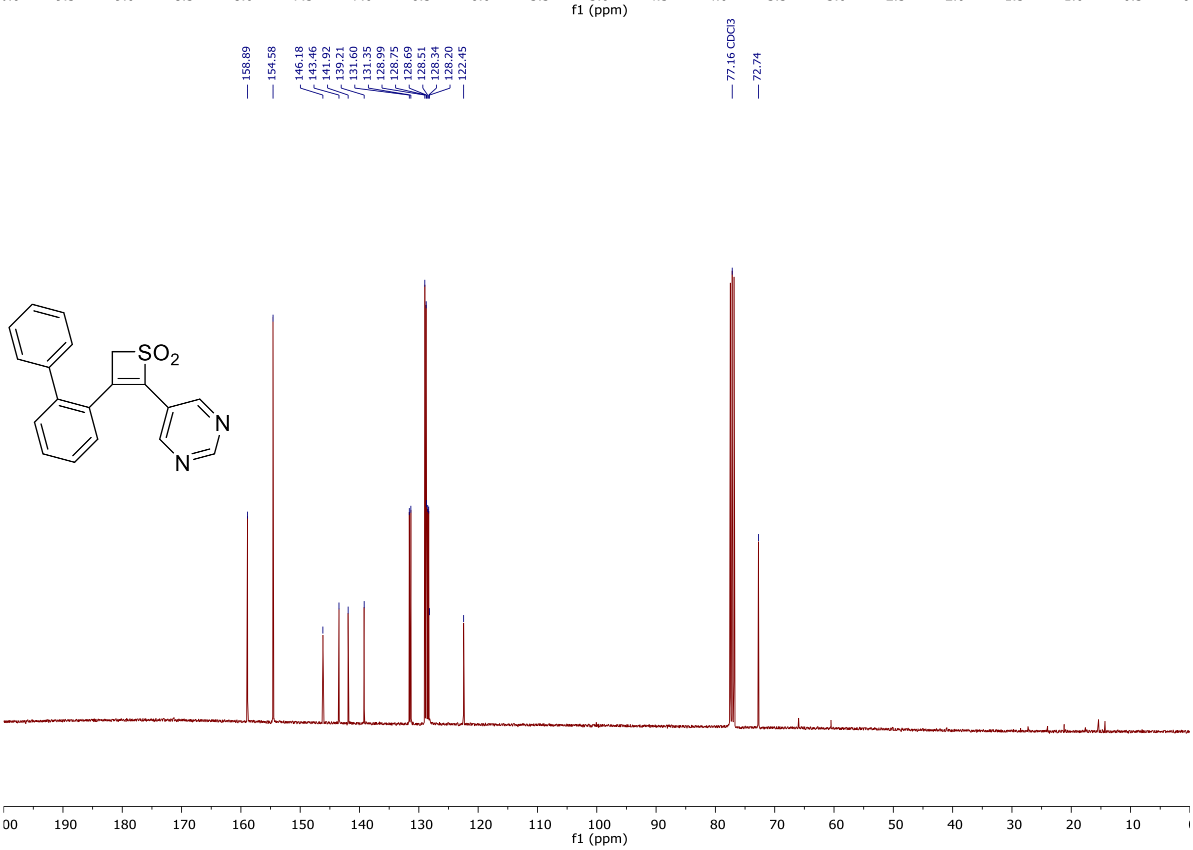

3-(4'-Methoxy-[1,1'-biphenyl]-2-yl)-4-(4-nitrophenyl)-2*H*-thiete 1,1-dioxide (5g)

^{1}H NMR (400 MHz, $CDCl_3$) and **^{13}C NMR** (101 MHz, $CDCl_3$)

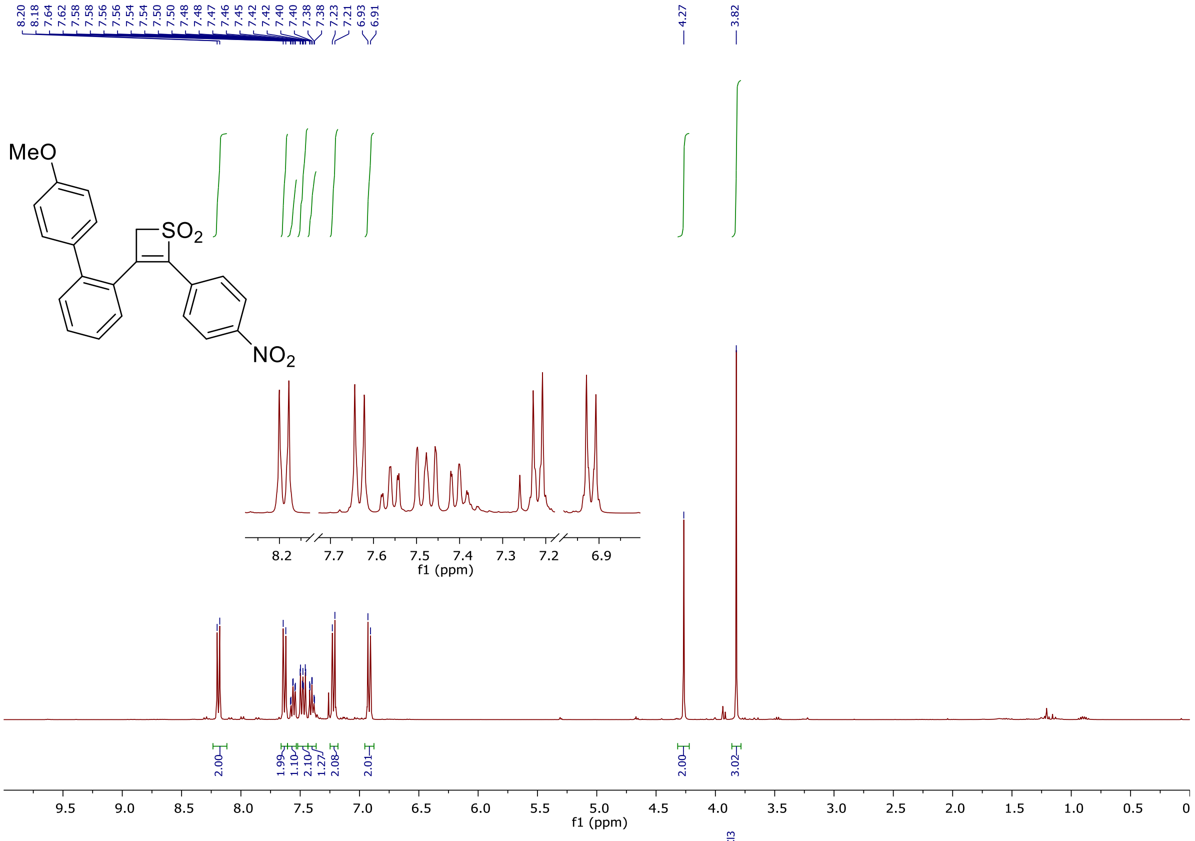

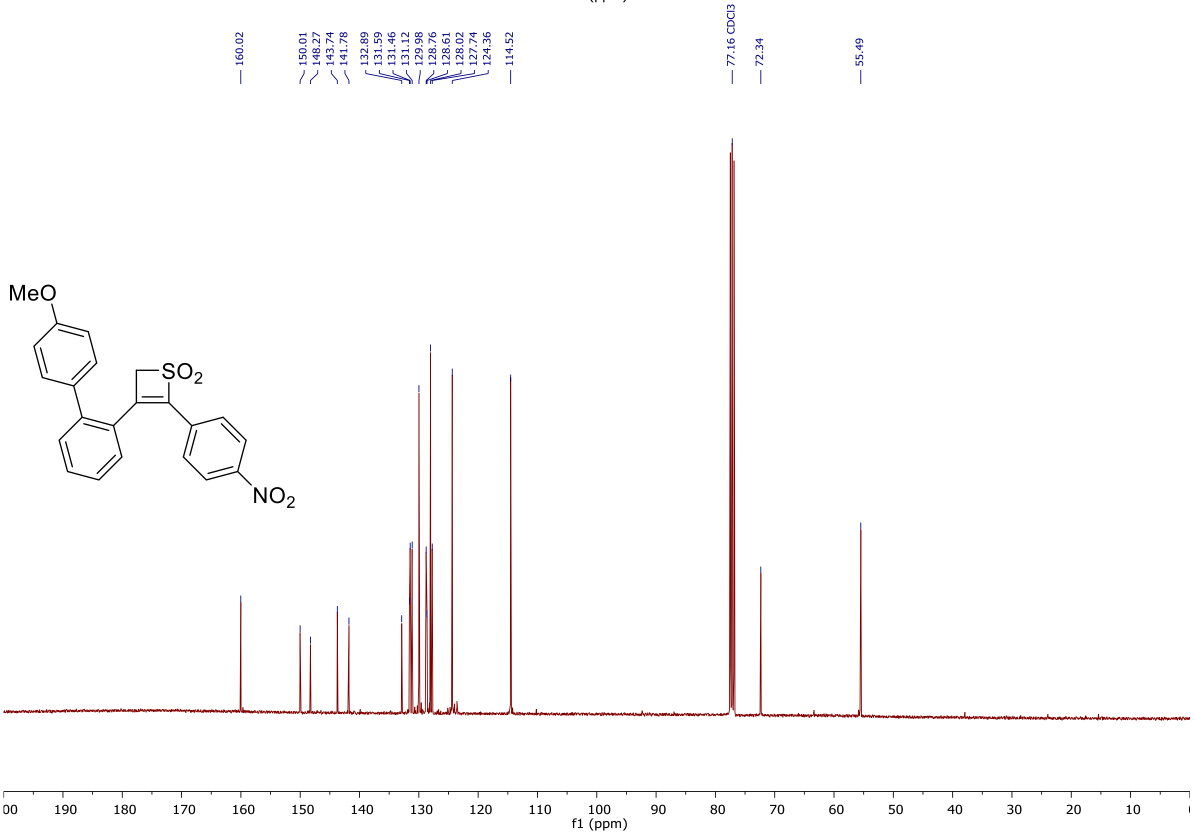

3-(4'-Methoxy-[1,1'-biphenyl]-2-yl)-4-(pyren-1-yl)-2*H*-thiete 1,1-dioxide (5h)

^{1}H NMR (400 MHz, CDCl$_3$) and **^{13}C NMR** (101 MHz, CDCl$_3$)

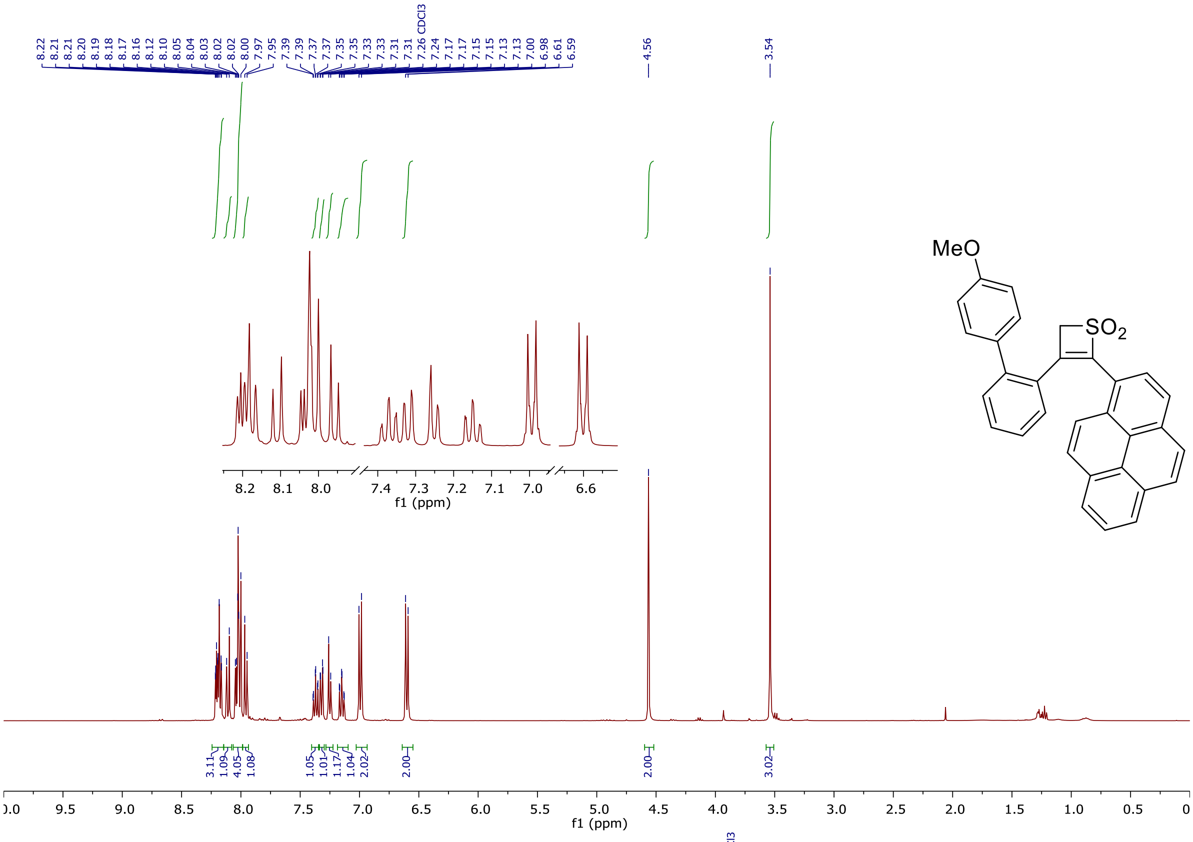

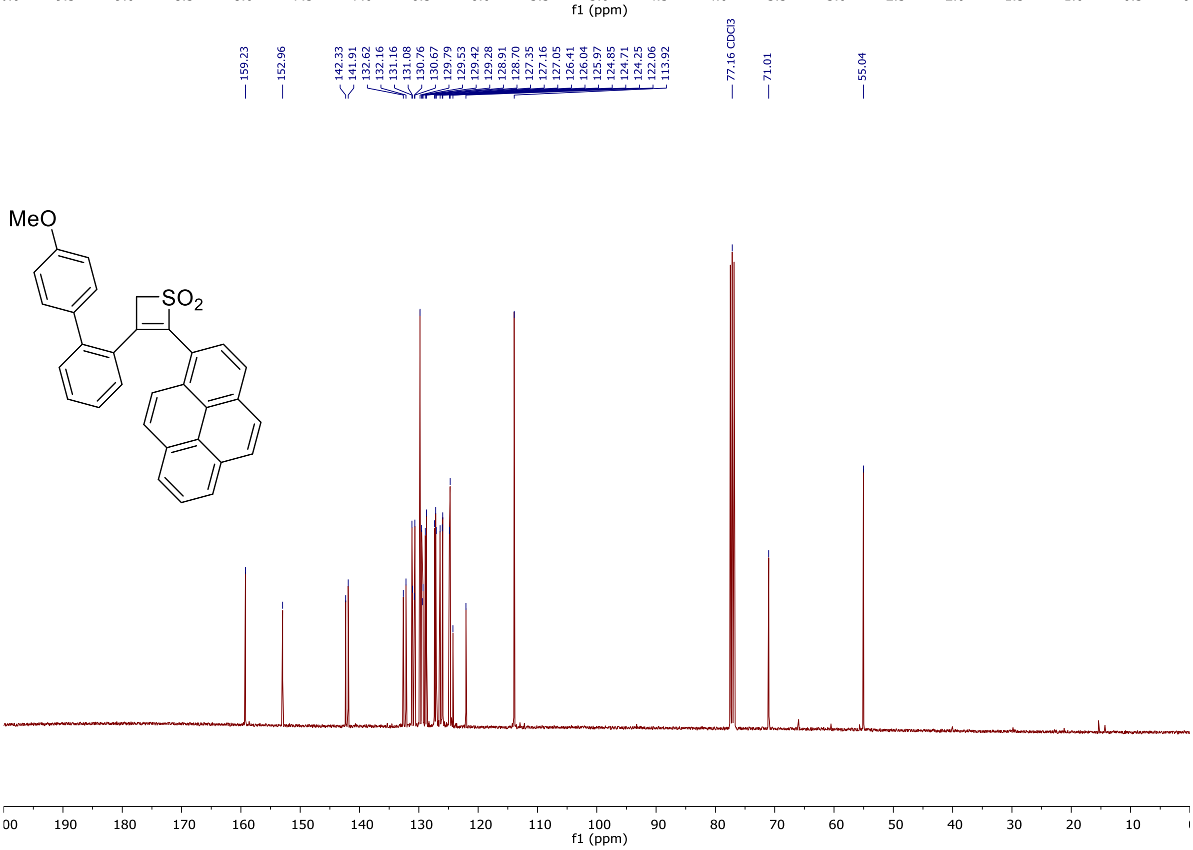

3-(2-Methoxynaphthalen-1-yl)-4-(4-phenylpyridin-3-yl)-2*H*-thiete 1,1-dioxide (6a)

^{1}H NMR (400 MHz, $CDCl_3$) and **^{13}C NMR** (101 MHz, $CDCl_3$)

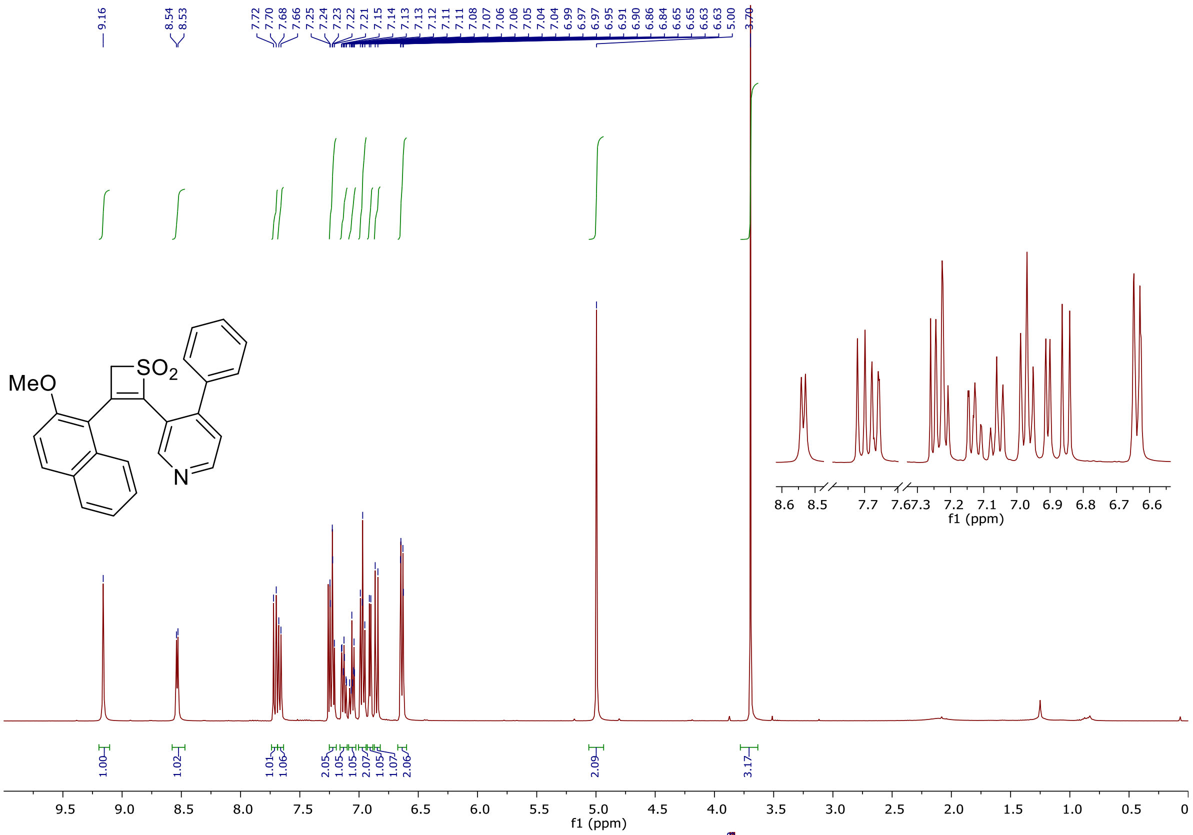

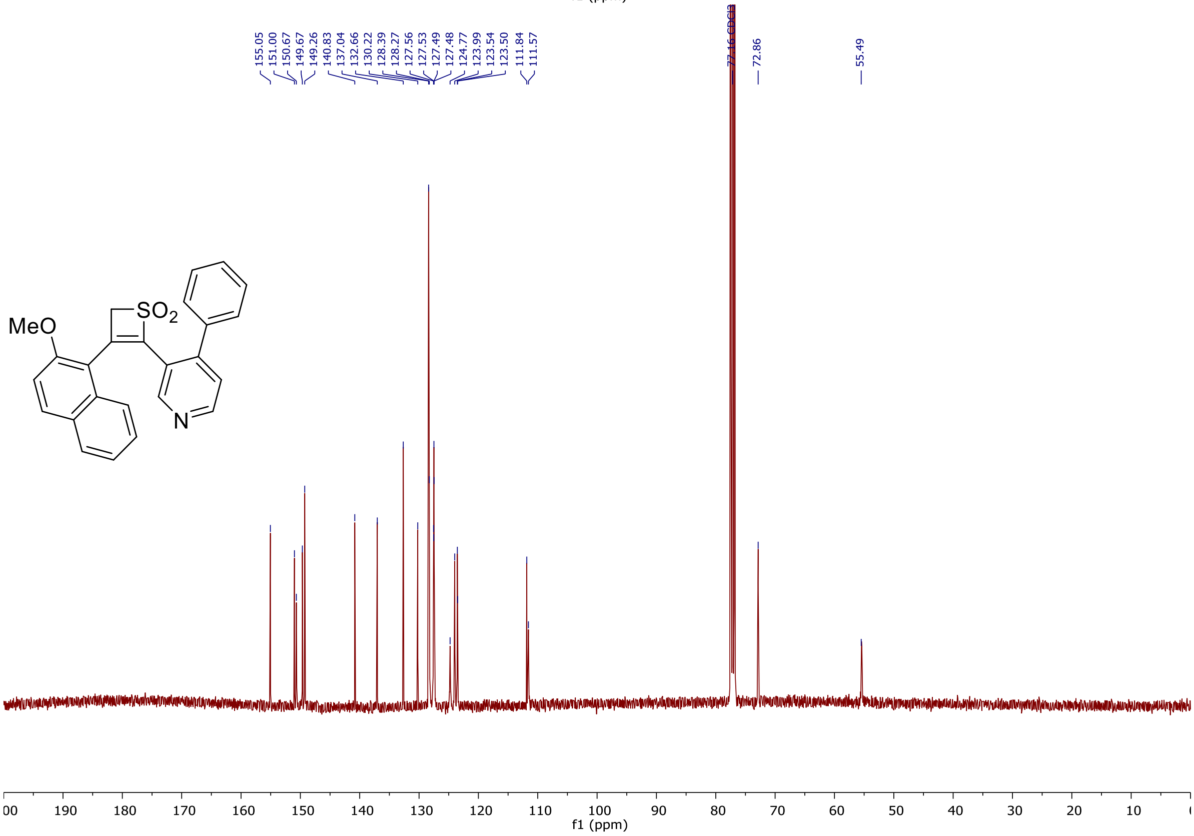

4-(3',5'-Dinitro-[1,1'-biphenyl]-2-yl)-3-(2-methoxynaphthalen-1-yl)-2*H*-thiete 1,1-dioxide (6b)

^{1}H NMR (400 MHz, $CDCl_3$) and **^{13}C NMR** (101 MHz, $CDCl_3$)

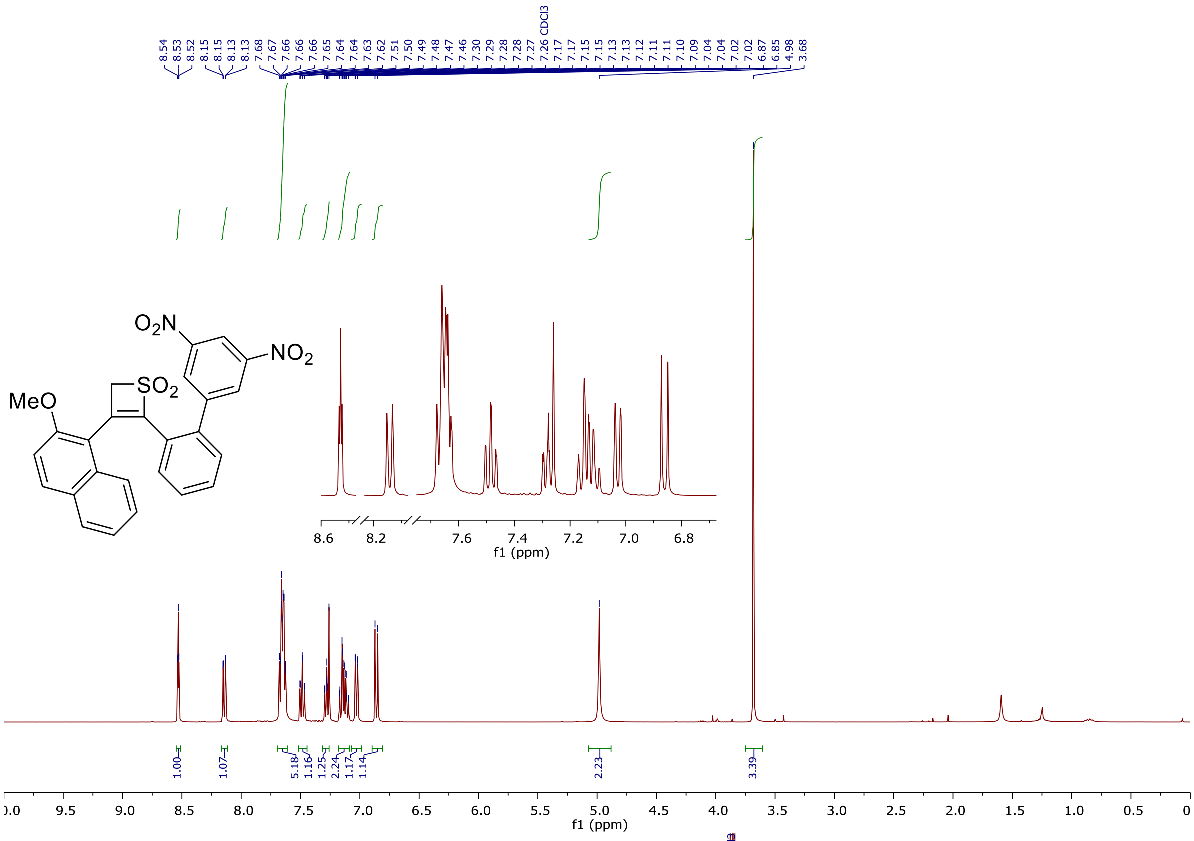

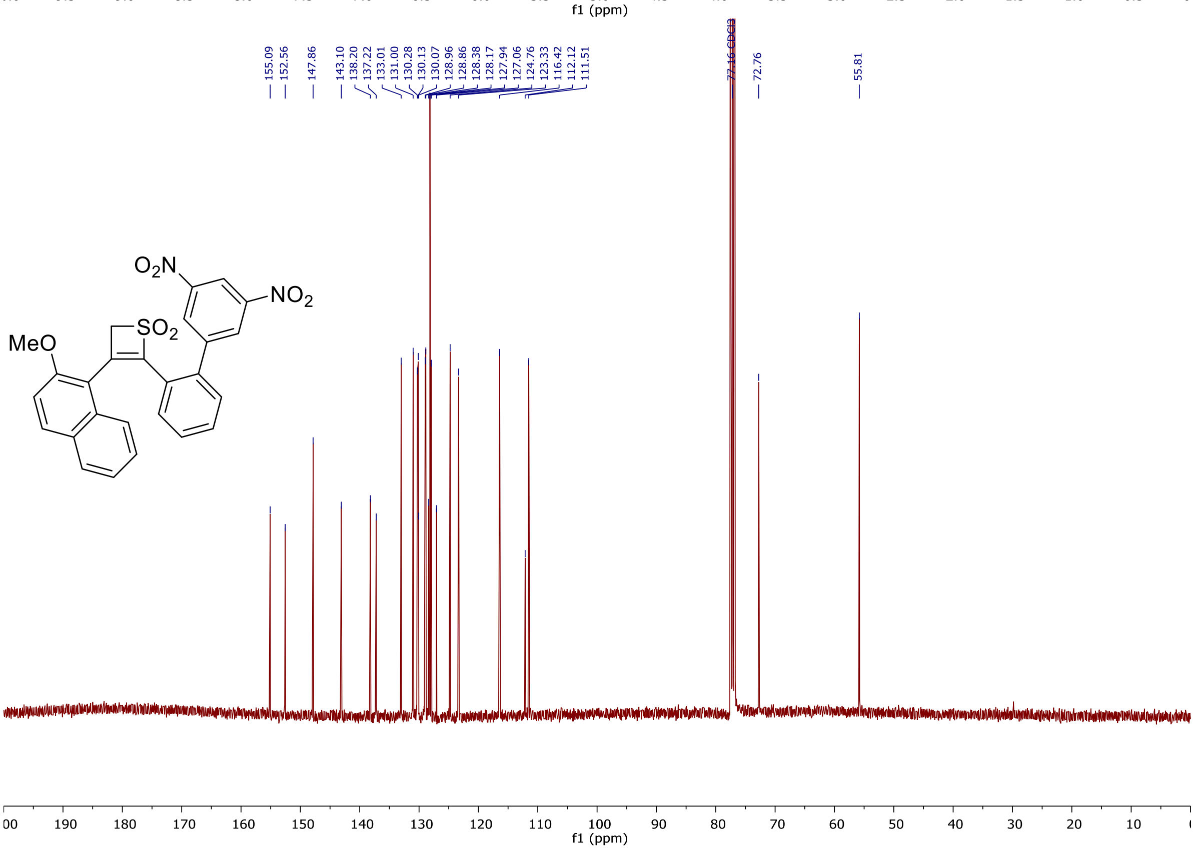

3-([1,1'-Biphenyl]-2-yl)-4-(4-phenylpyridin-3-yl)-2*H*-thiete 1,1-dioxide (6c)

^{1}H NMR (400 MHz, CDCl$_3$) and **^{13}C NMR** (101 MHz, CDCl$_3$)

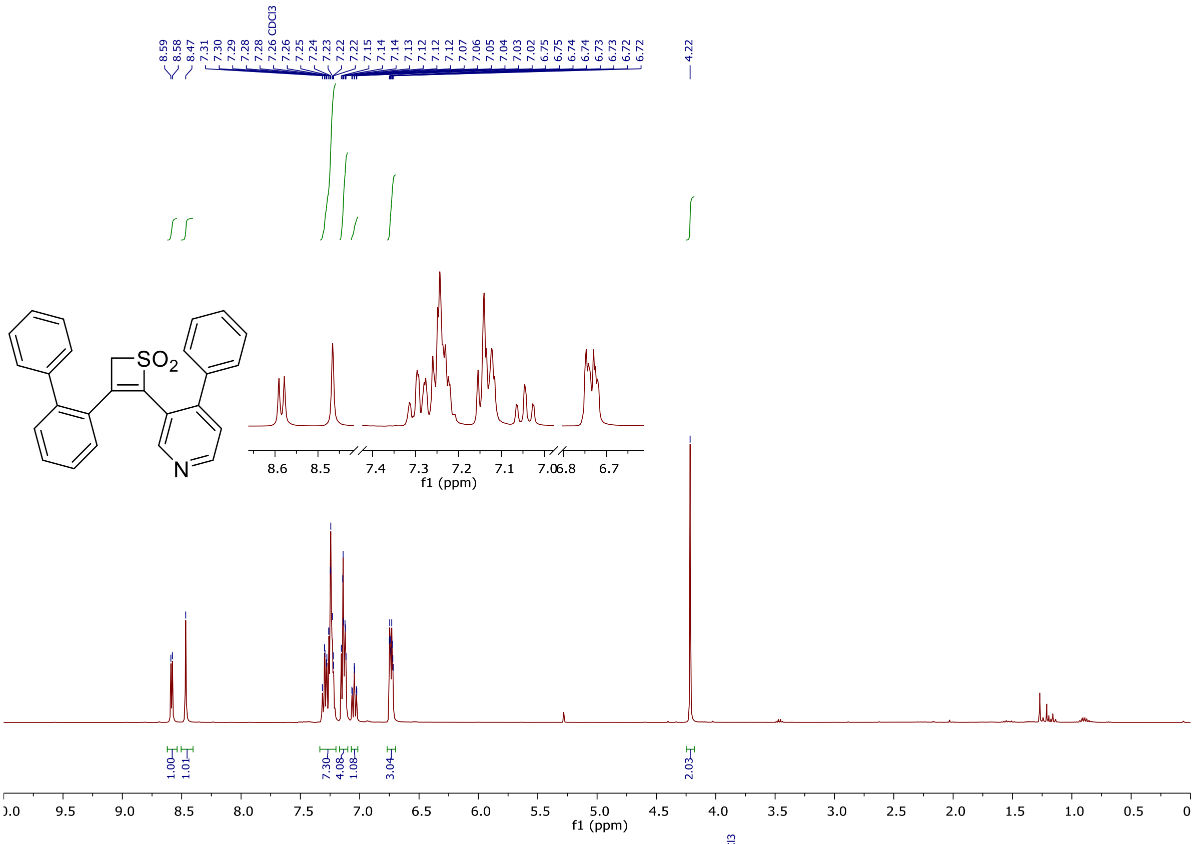

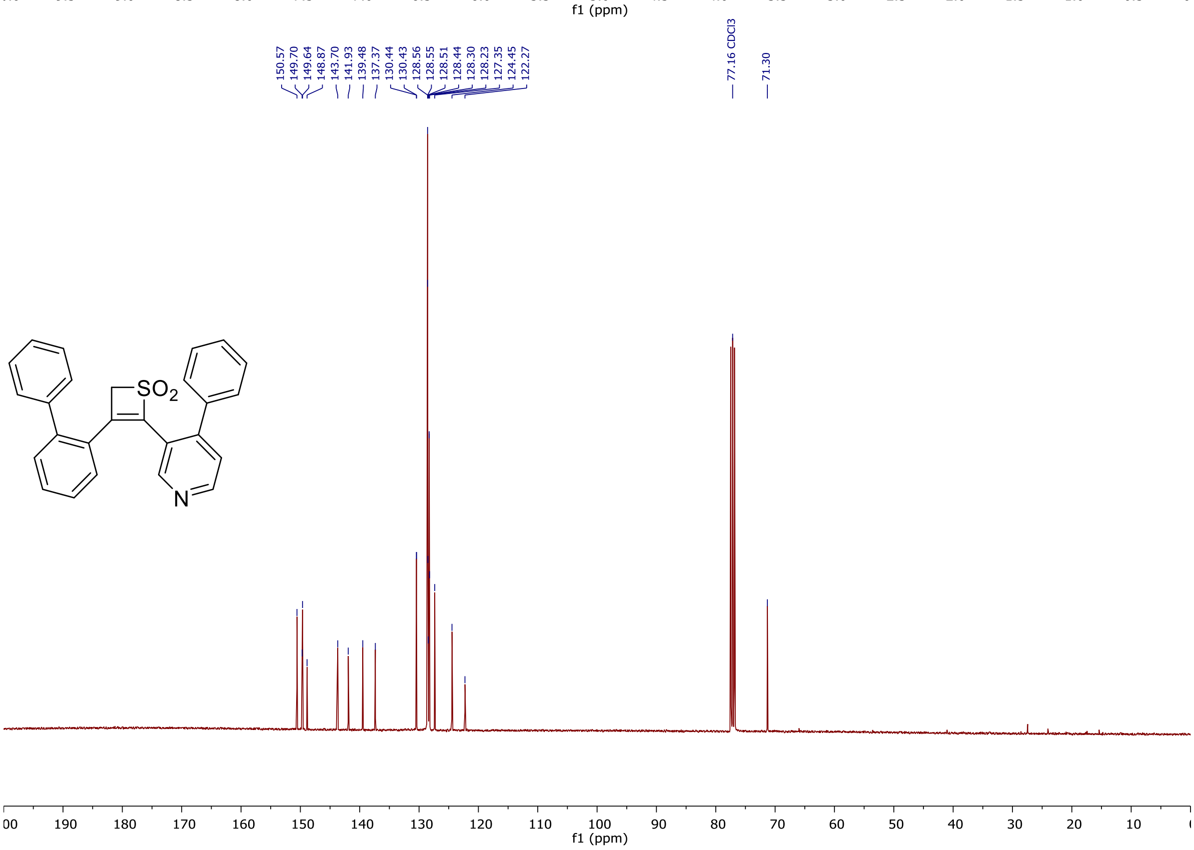

3-(4'-Methoxy-[1,1'-biphenyl]-2-yl)-4-(4-phenylpyridin-3-yl)-2*H*-thiete 1,1-dioxide (6d)

^{1}H NMR (400 MHz, $CDCl_3$) and **^{13}C NMR** (101 MHz, $CDCl_3$)

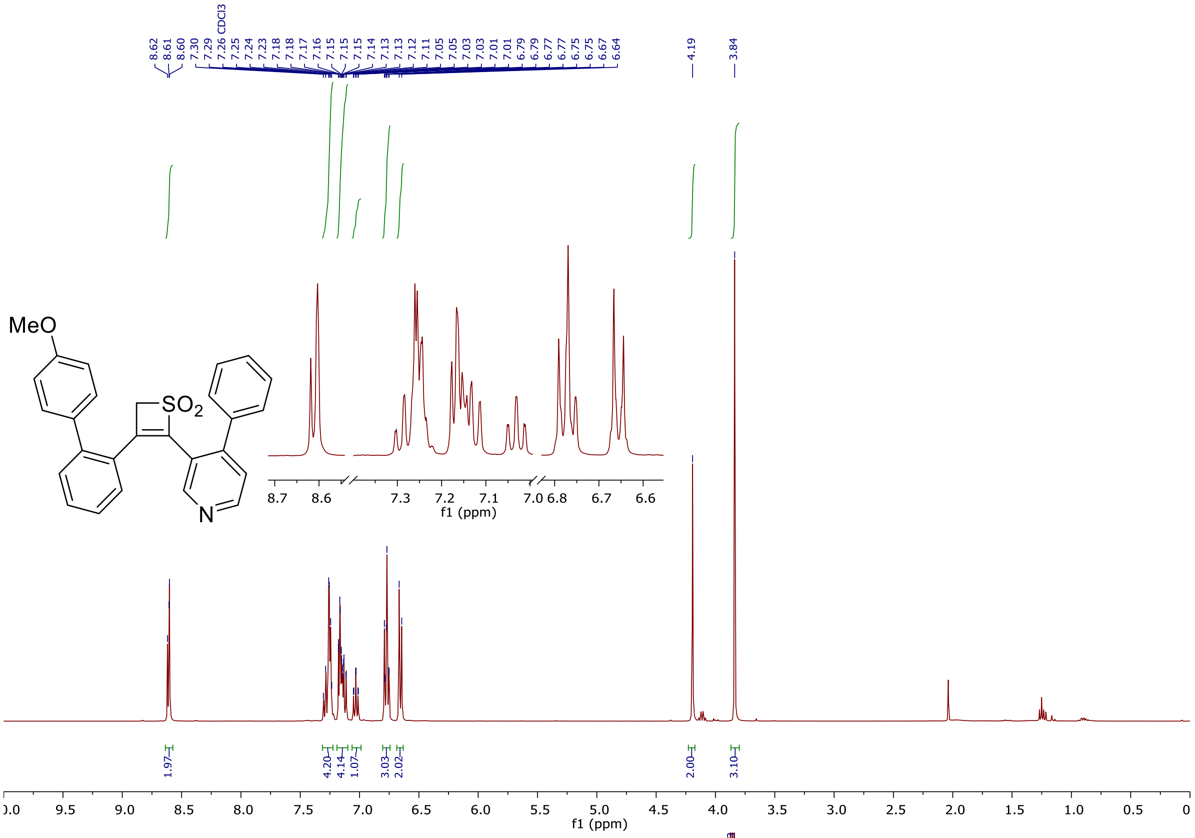

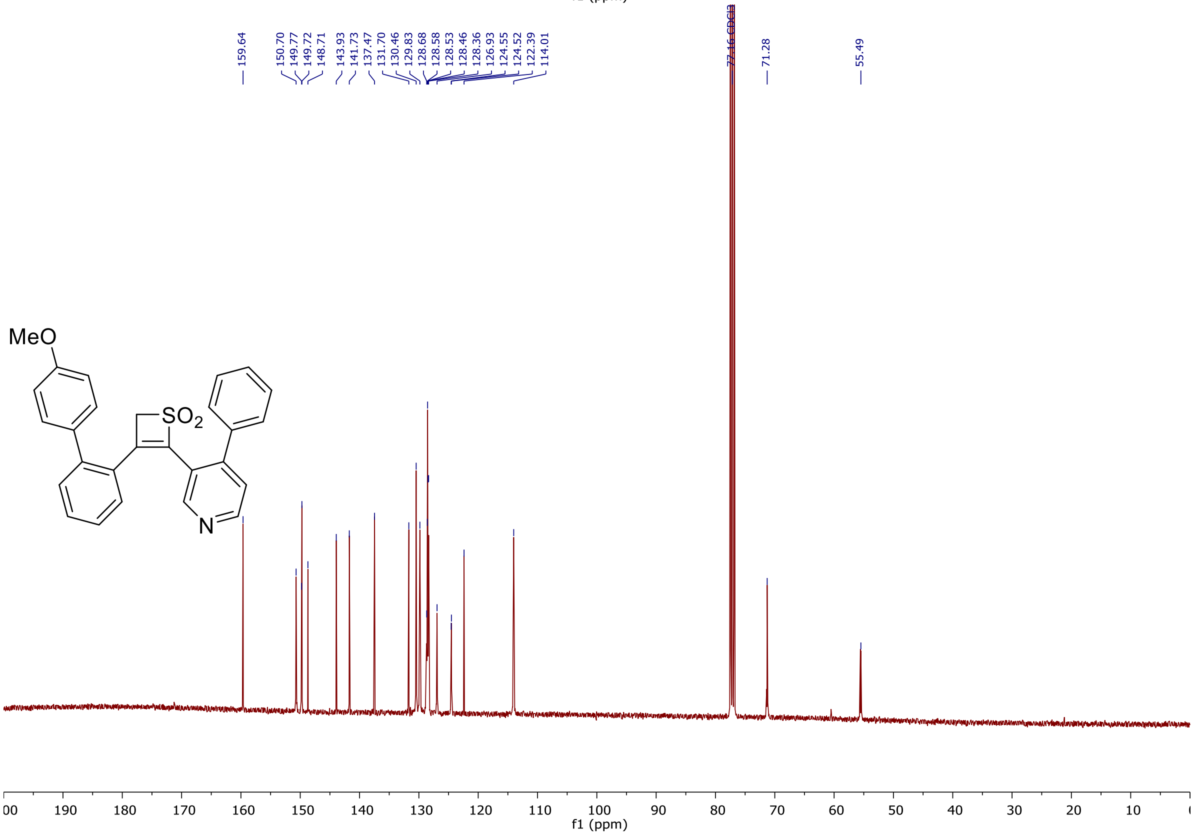

Cyclotris (3-phenylthiete 1,1-dioxide-3’, 4-diyl) (7a)

^{1}**H NMR** (400 MHz, $CDCl_3$) and ^{13}**C NMR** (101 MHz, $CDCl_3$)

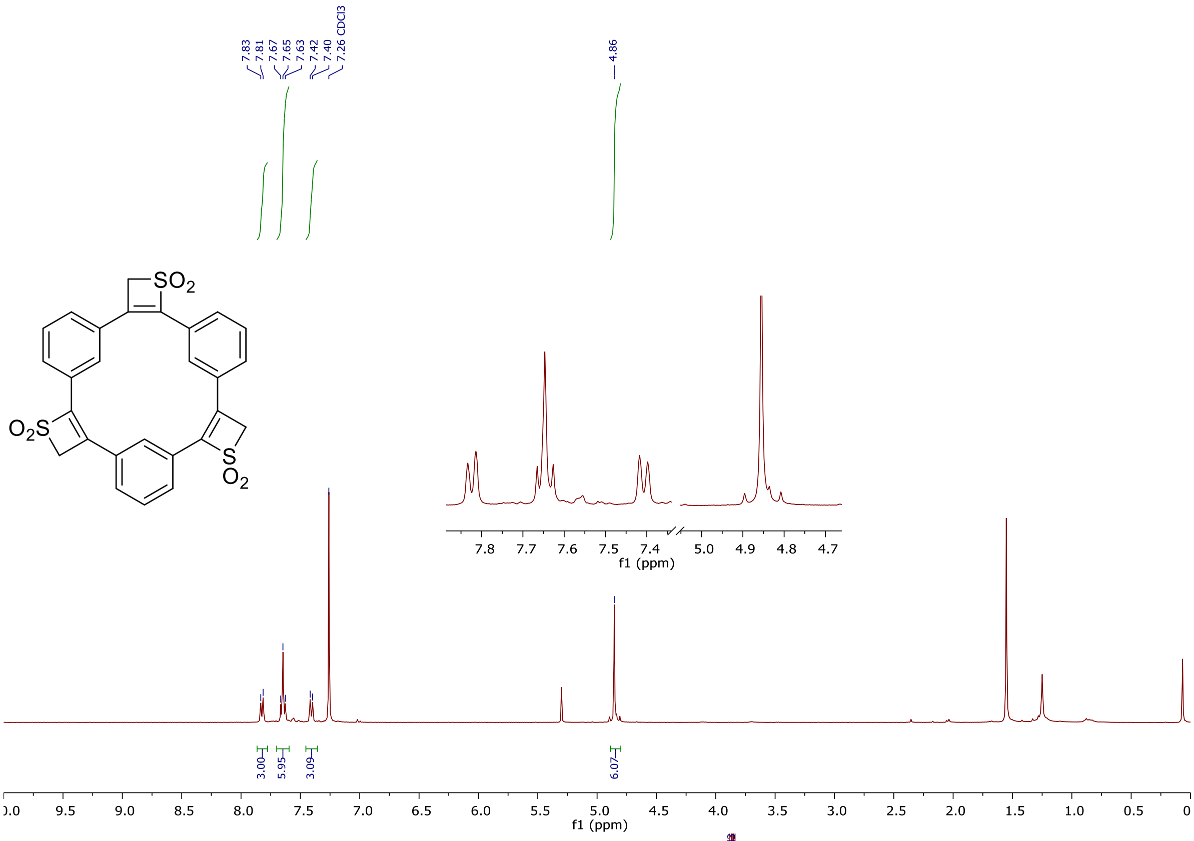

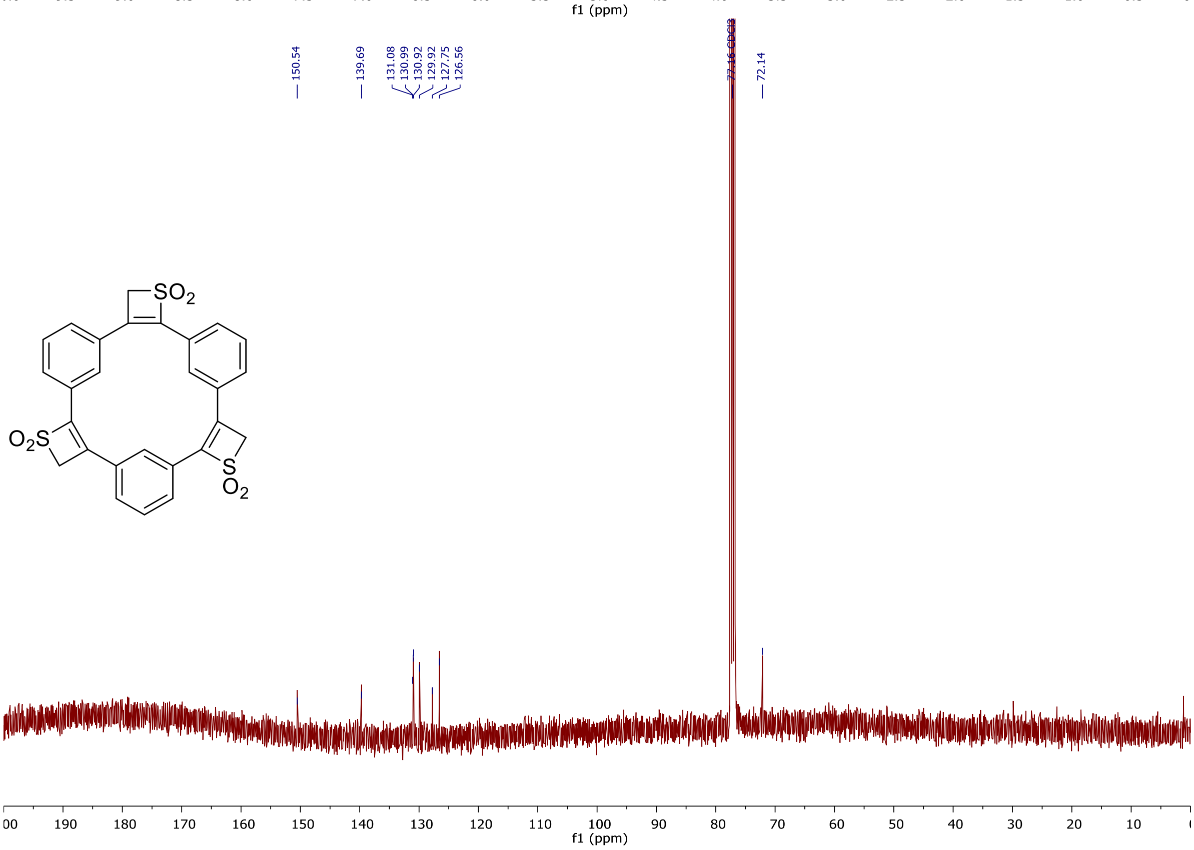

Cyclotris (3-(3’-methylphenyl)thiete 1,1-dioxide-5’, 4-diyl)

^{1}H NMR (400 MHz, CD_2Cl_2) and **^{13}C NMR** (101 MHz, CD_2Cl_2)

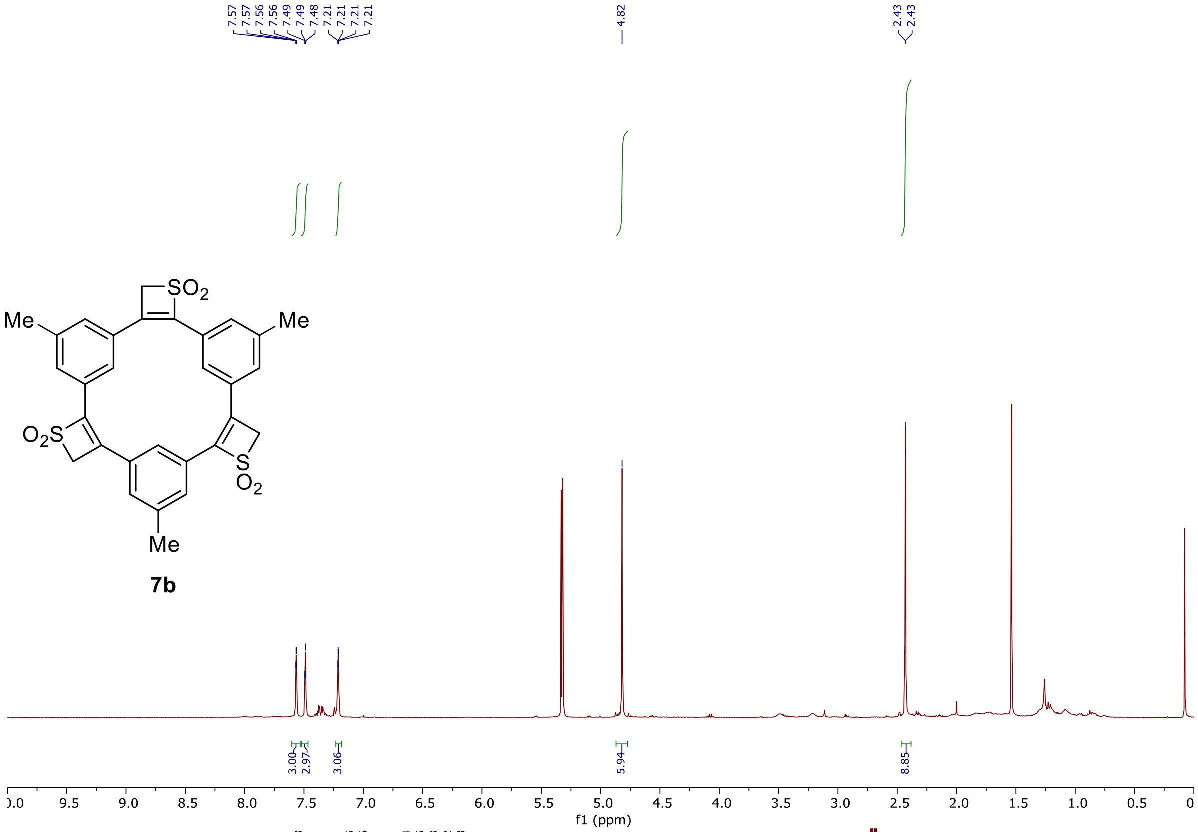

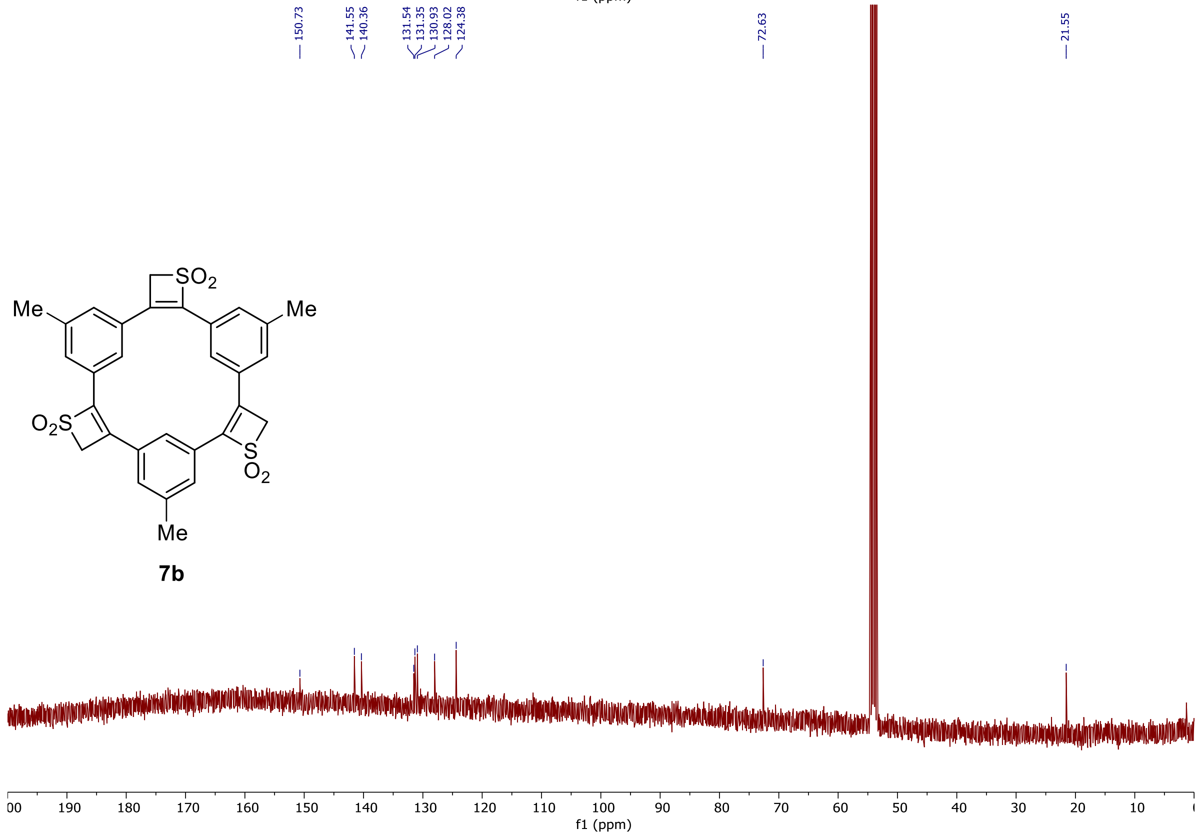

Cyclotris (3-(3’-*tert*-butylphenyl)thiete 1,1-dioxide-5’, 4-diyl) (7c)

^{1}H NMR (400 MHz, CD_2Cl_2) and **^{13}C NMR** (101 MHz, CD_2Cl_2)

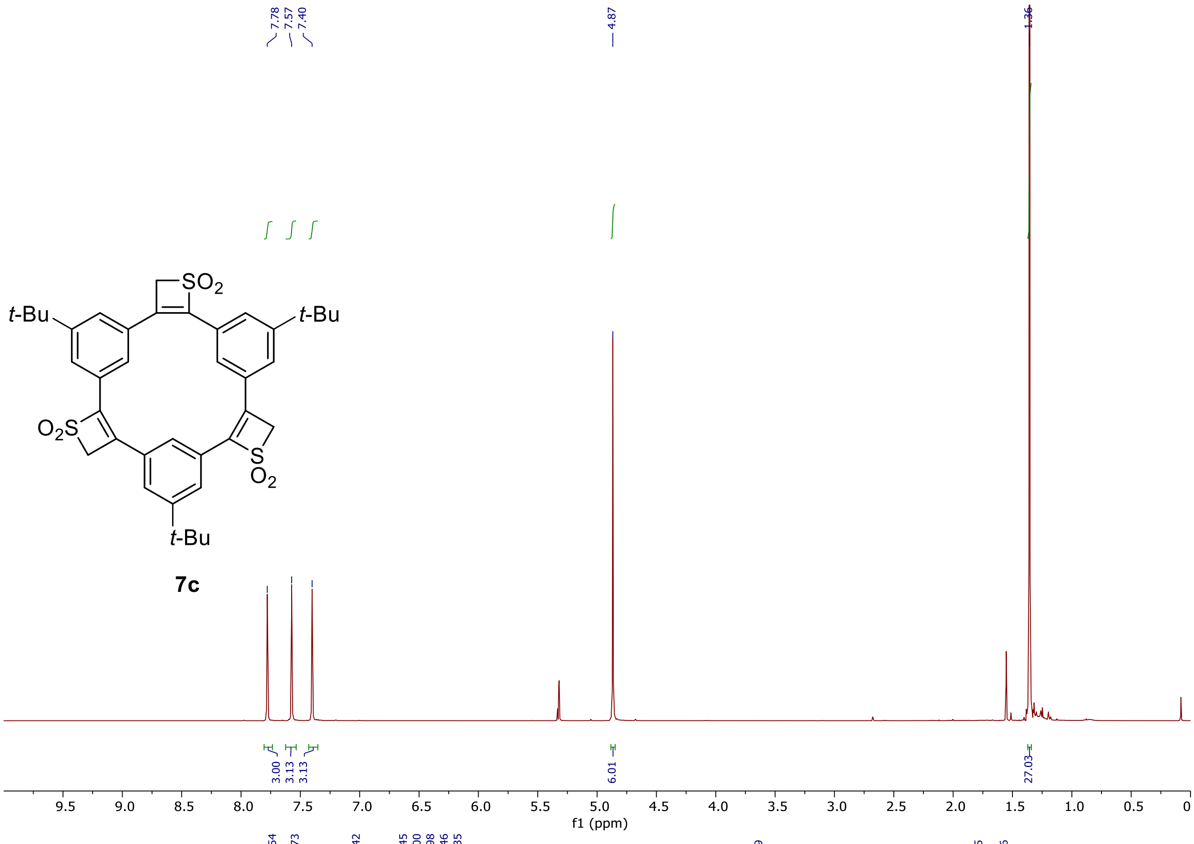

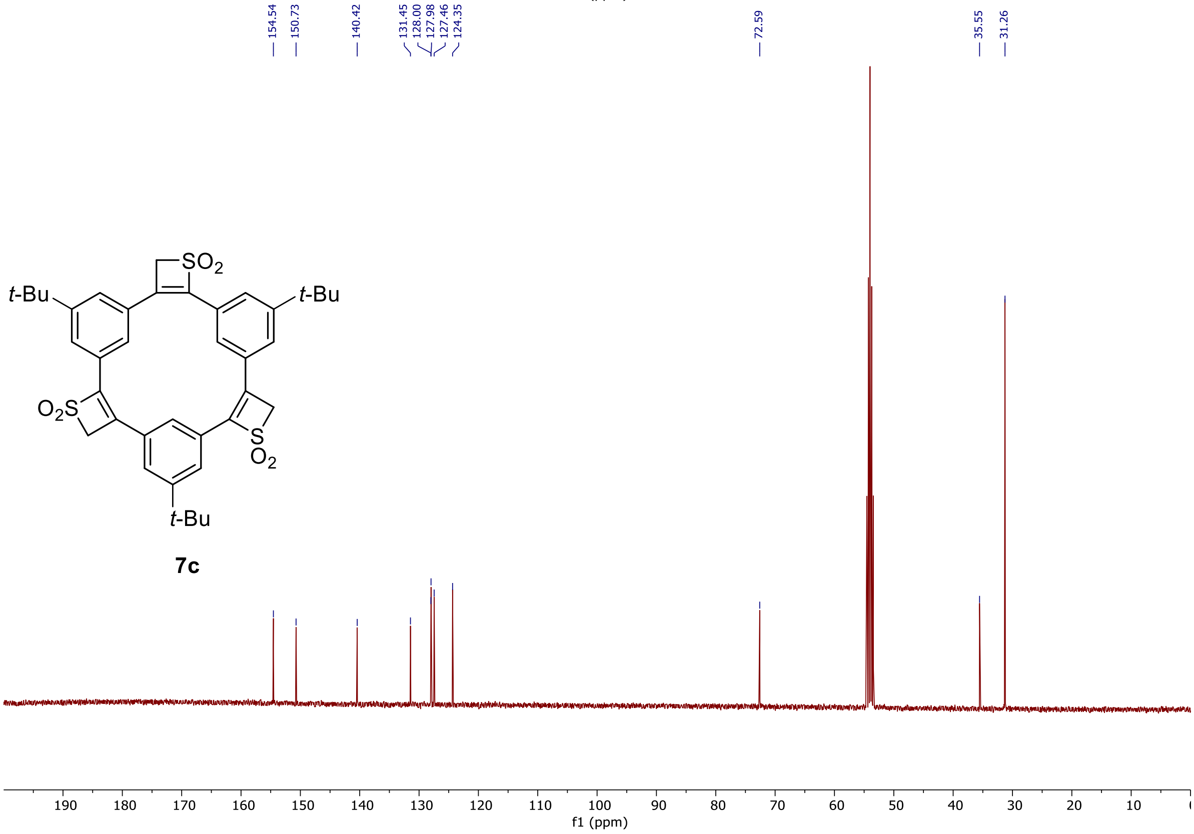

Cyclotris (3-(3'-fluorophenyl)thiete 1,1-dioxide-5', 4-diyl) (7d)

^{1}H NMR (400 MHz, CD_2Cl_2) and **^{13}C NMR** (101 MHz, CD_2Cl_2)

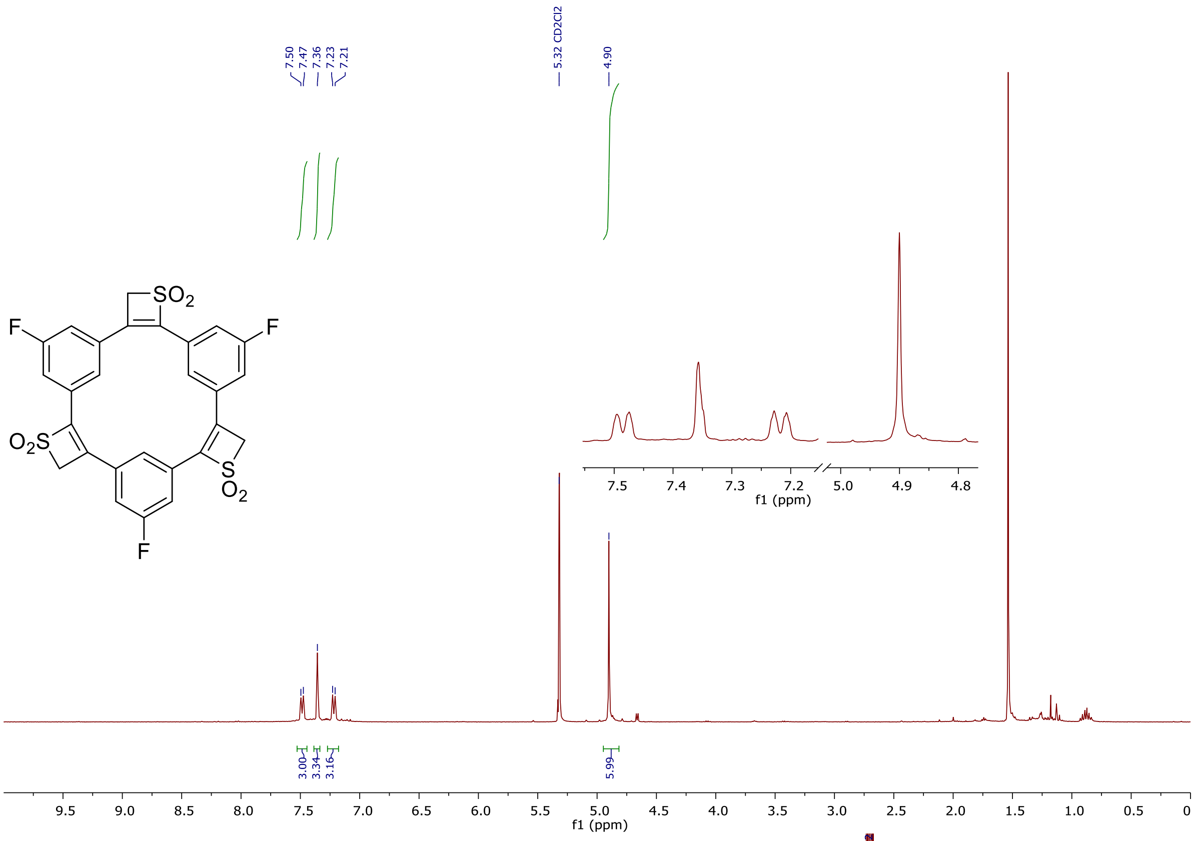

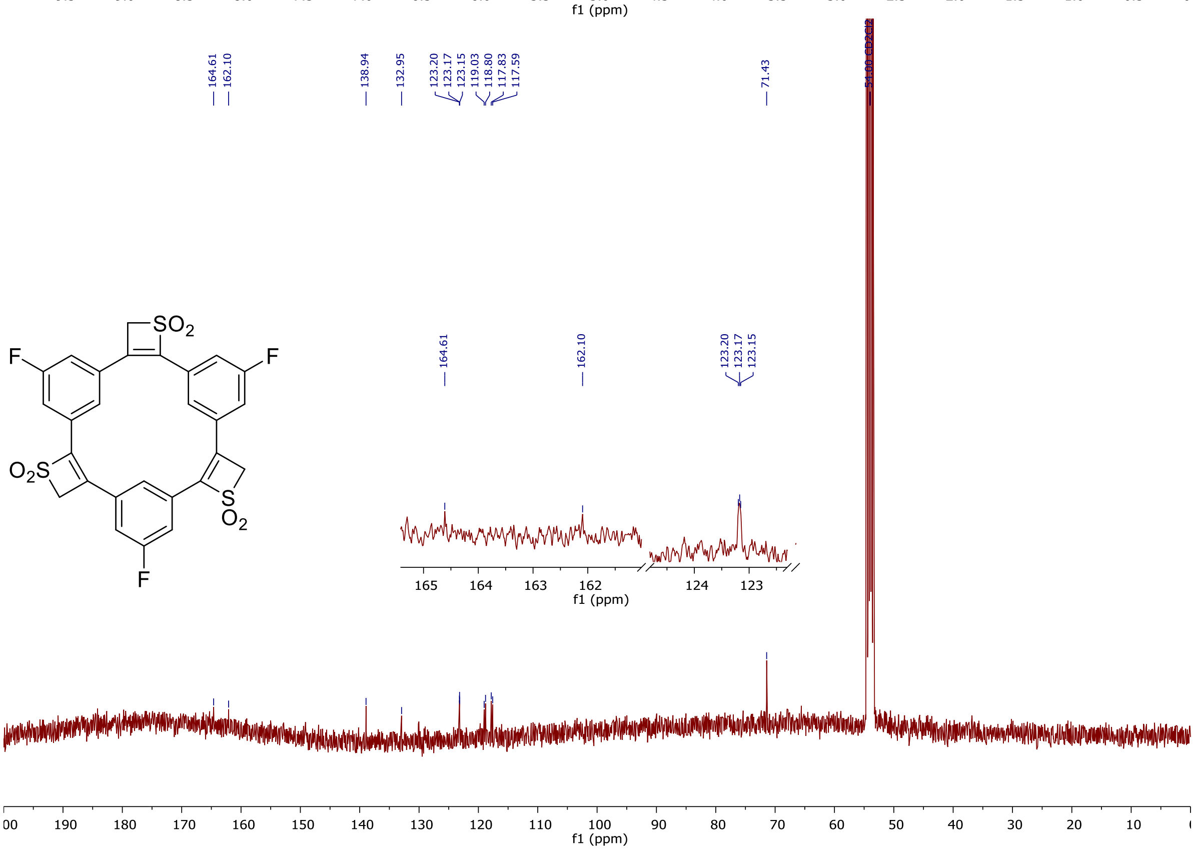

Cyclotris (3-(3'-methoxyphenyl)thiete 1,1-dioxide-5', 4-diyl) (7e)

^{1}H NMR (400 MHz, $CDCl_3$) and **^{13}C NMR** (101 MHz, $CDCl_3$)

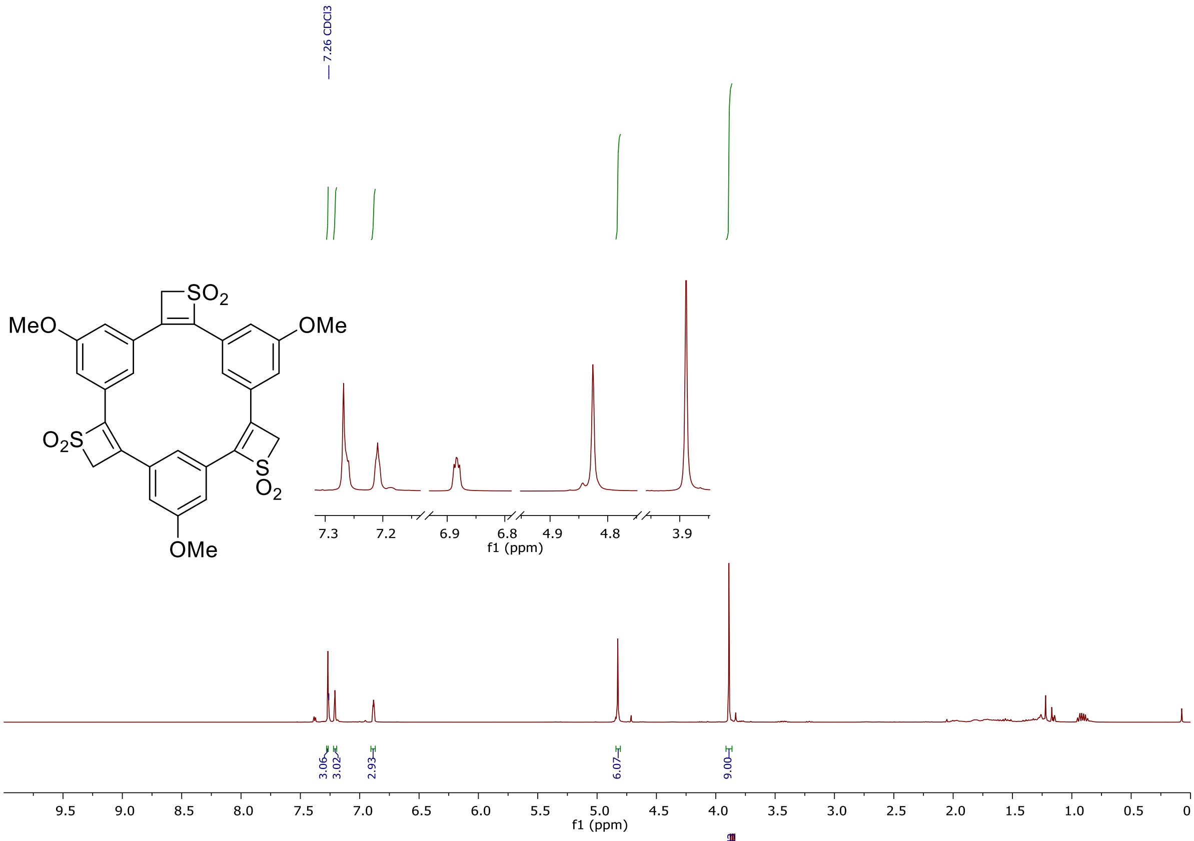

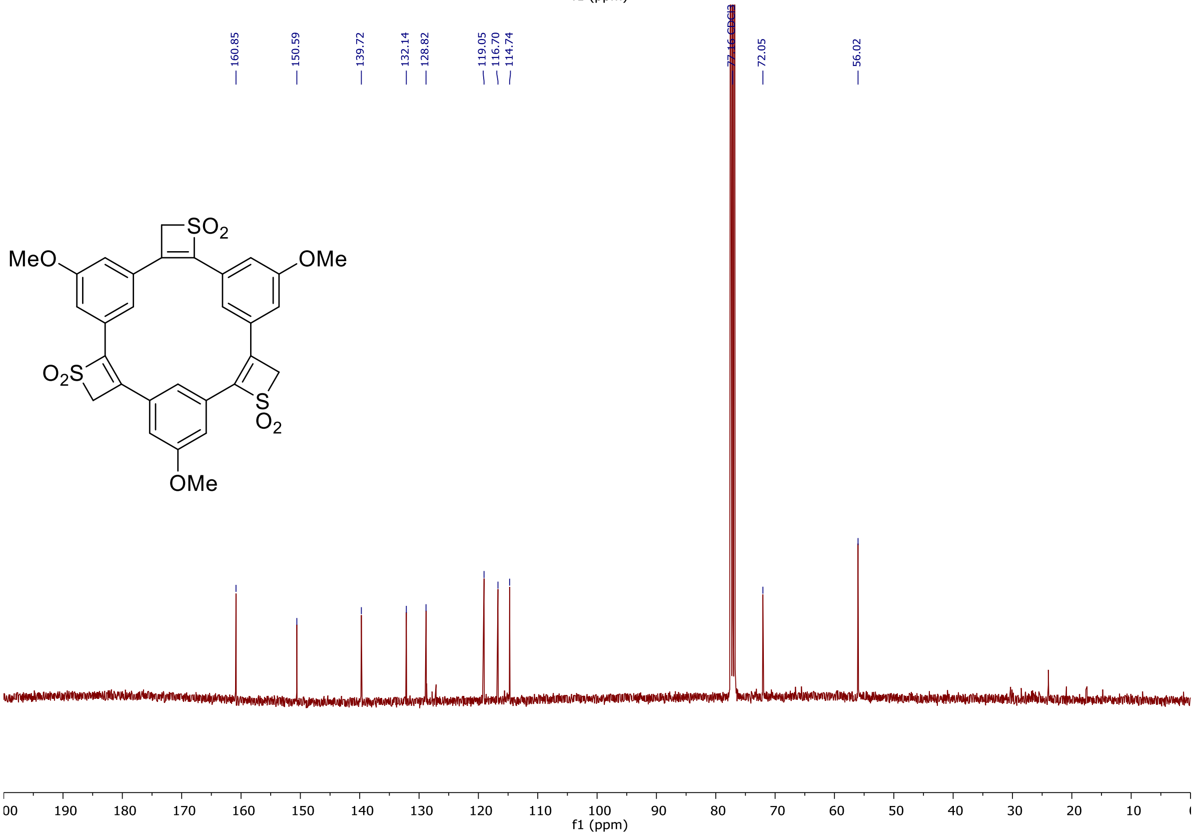

Cyclotris (3-(3'-biphenyl)thiete 1,1-dioxide-5', 4-diyl) (7g)

^{1}H NMR (400 MHz, CD_2Cl_2) and **^{13}C NMR** (101 MHz, CD_2Cl_2)

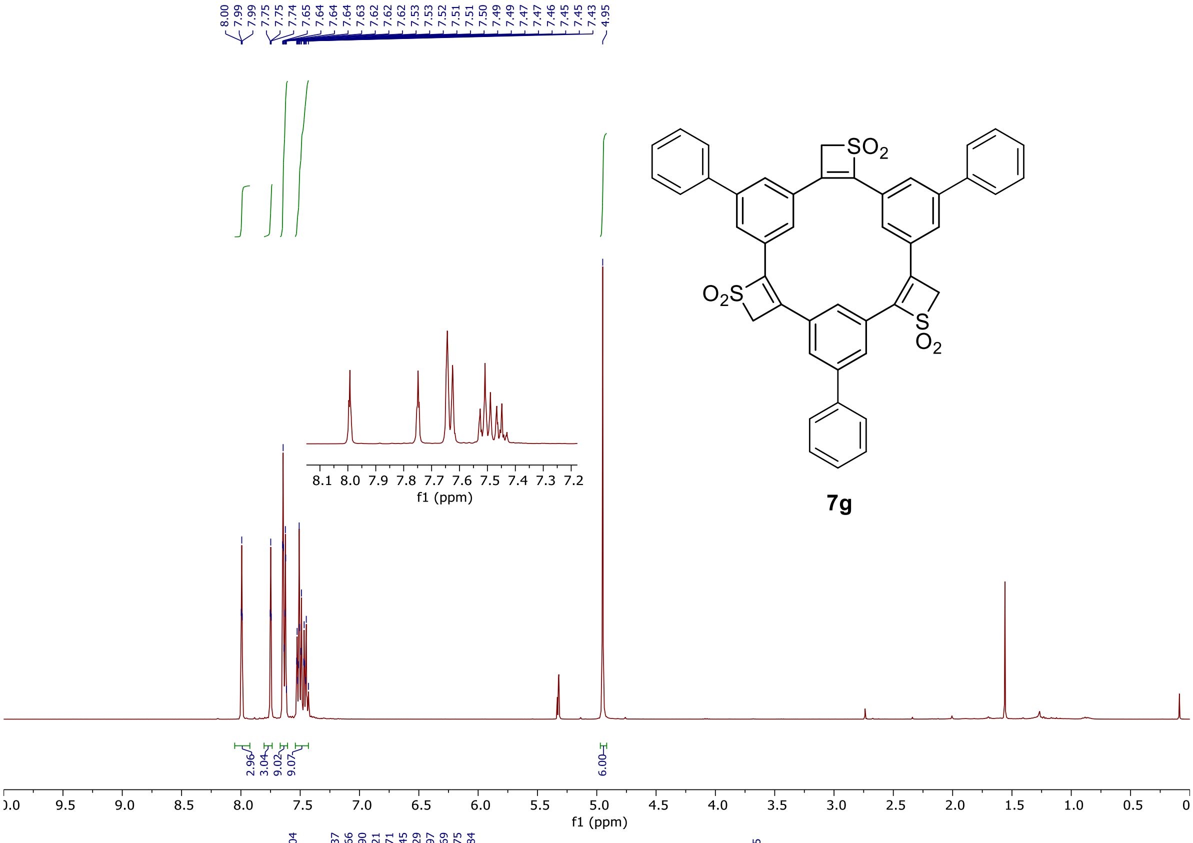

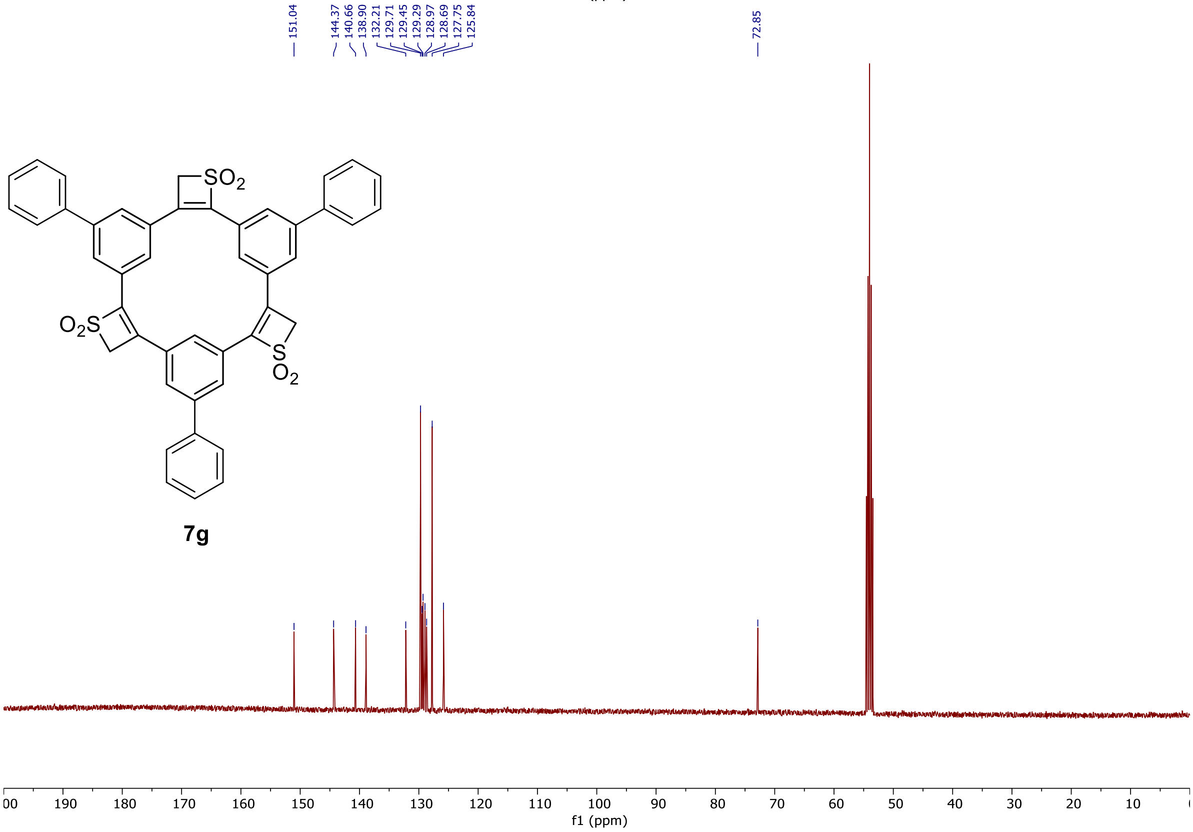

Cyclotris (3-(2’-naphtyl)thiete 1,1-dioxide-7’, 4-diyl) (7h)

^{1}H NMR (400 MHz, $CDCl_3$) and **^{13}C NMR** (101 MHz, $CDCl_3$)

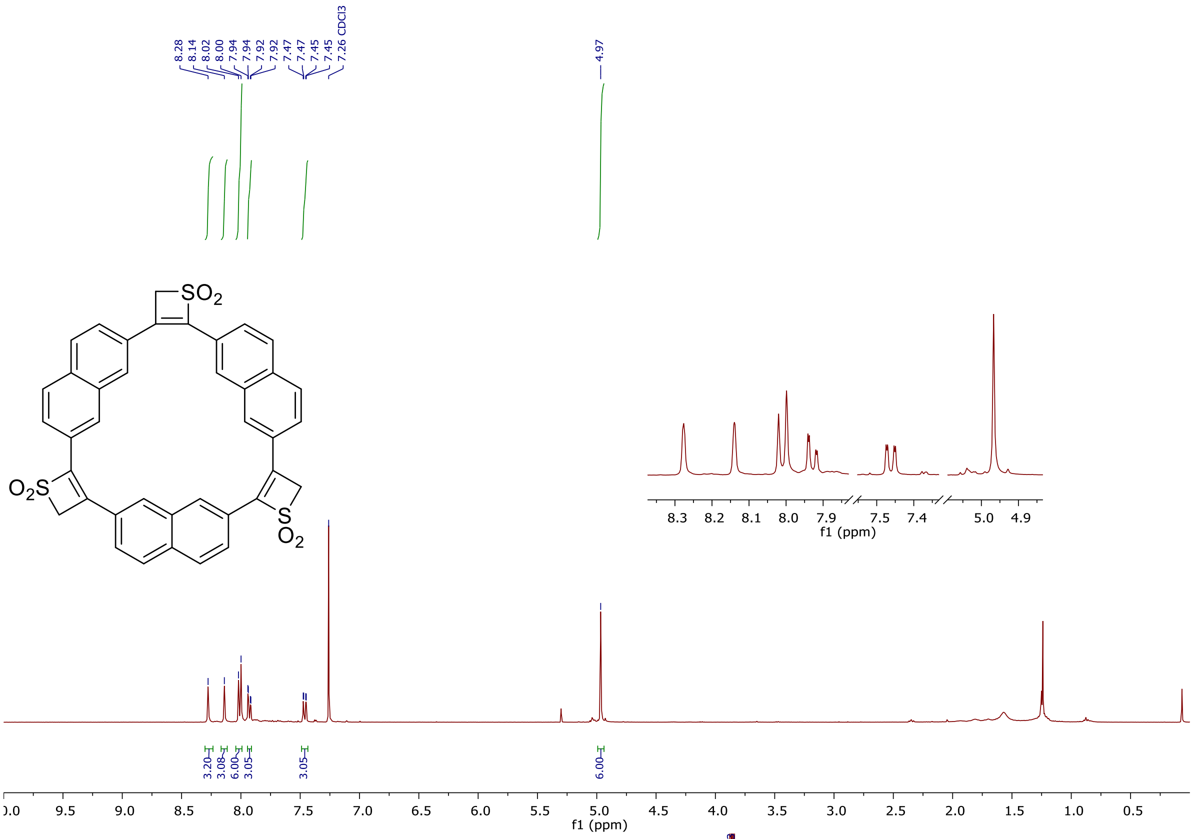

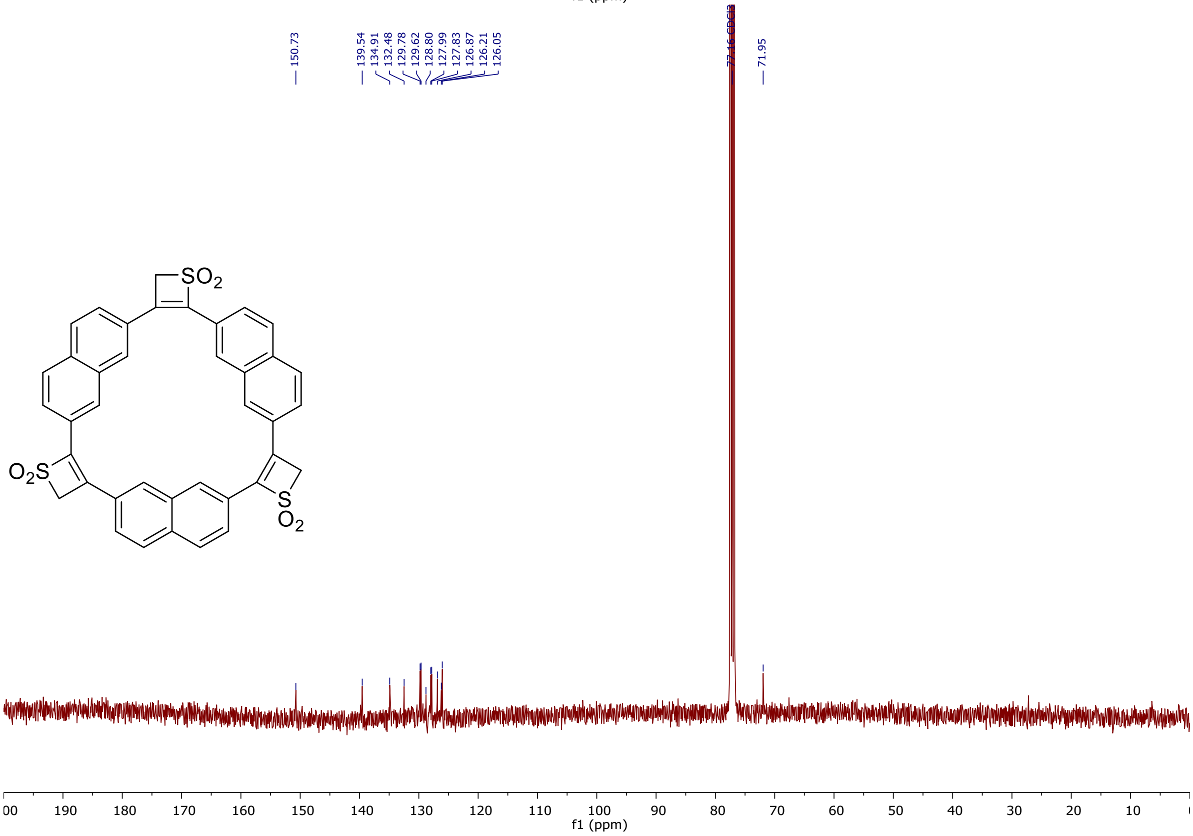

Cyclotetrakis (3-(3’-biphenyl)thiete 1,1-dioxide-5’, 4-diyl) (8a)

^{1}H NMR (400 MHz, DMSO-d_6, 80 °C)

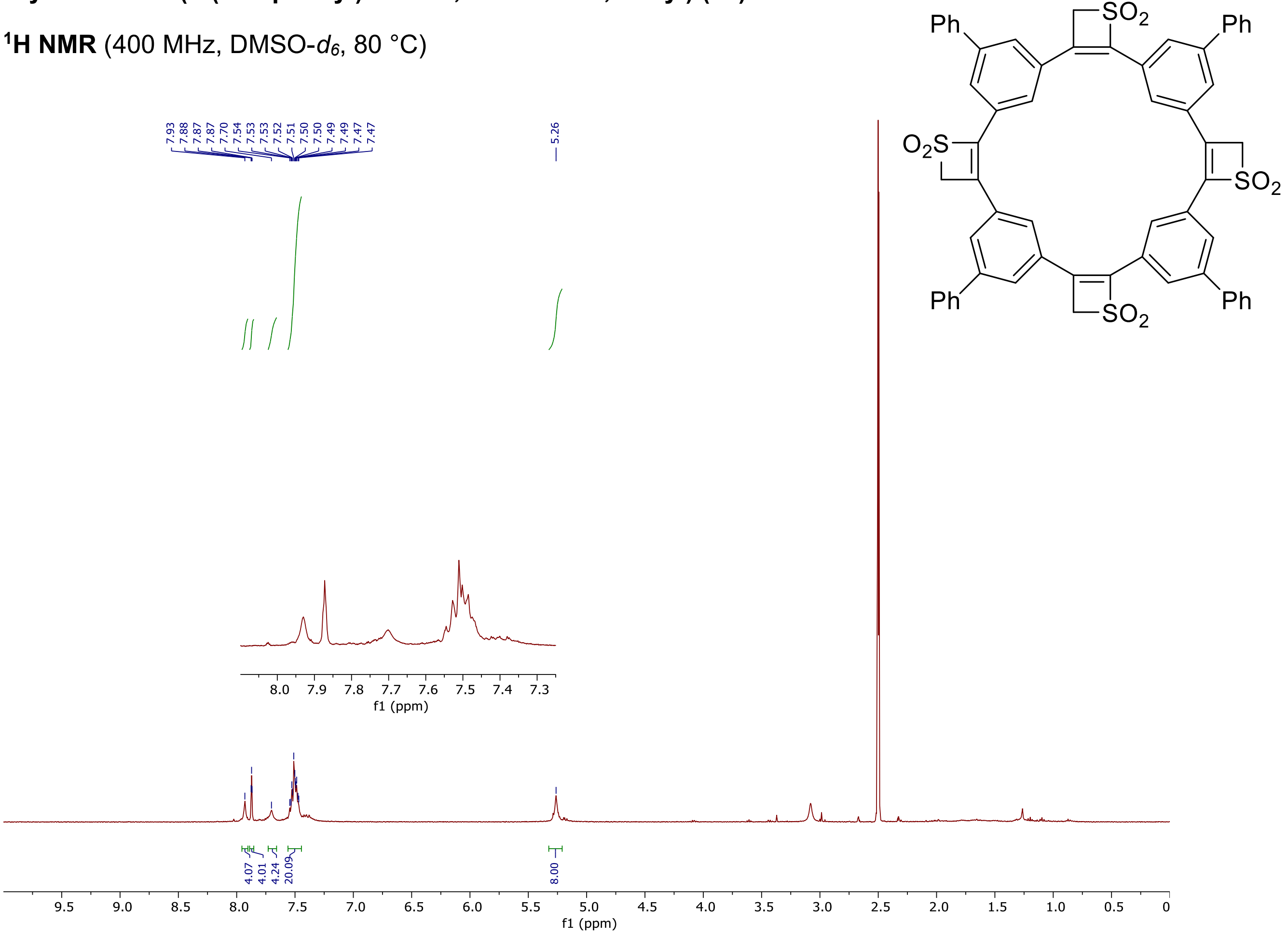

4. HPLC of Helicoidal Compounds

4.1 Column Screening

No separation of enantiomers could be observed for **3a**

Conditions: **3a** in IPA, 5 µL injection, 1 mL/min, λ = 220 nm, r.t.

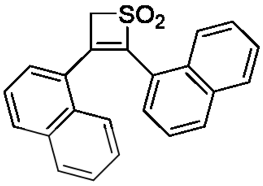

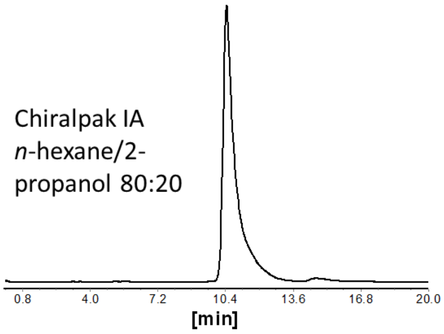

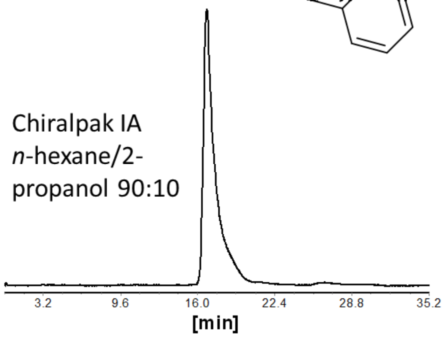

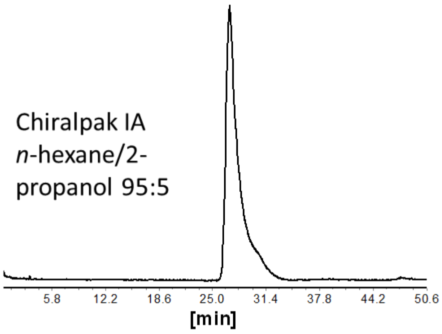

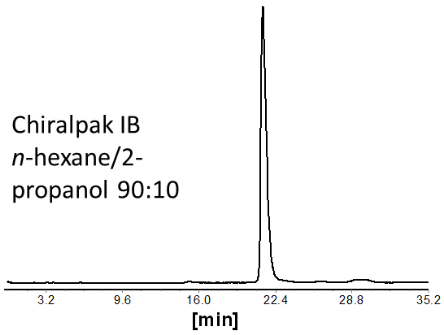

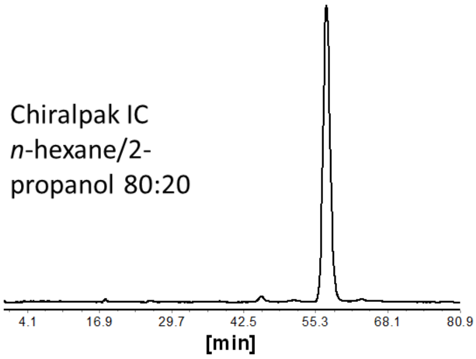

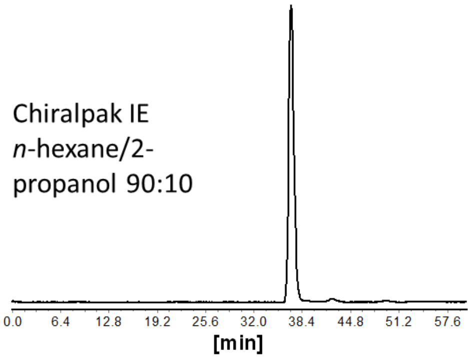

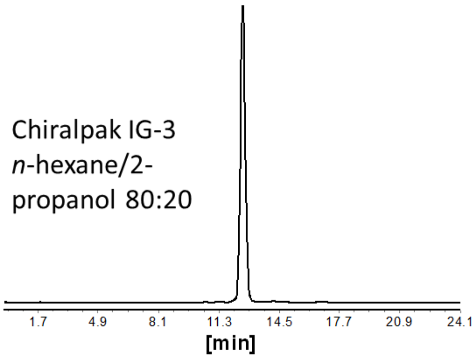

No separation of enantiomers could be observed for **3d**

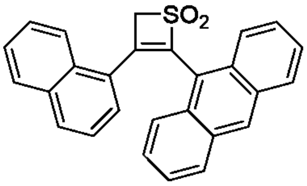

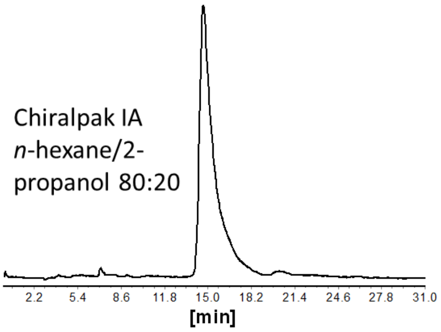

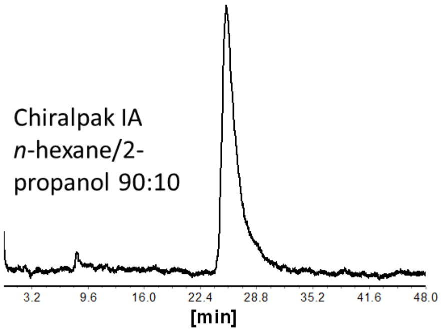

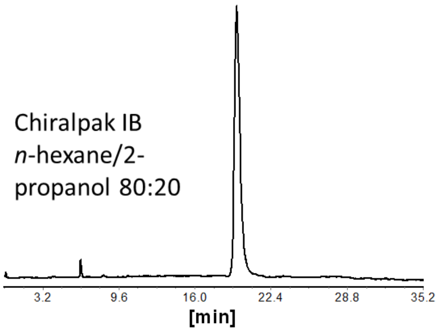

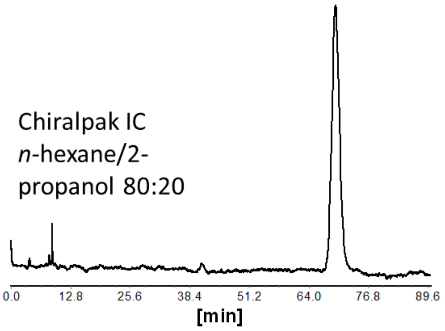

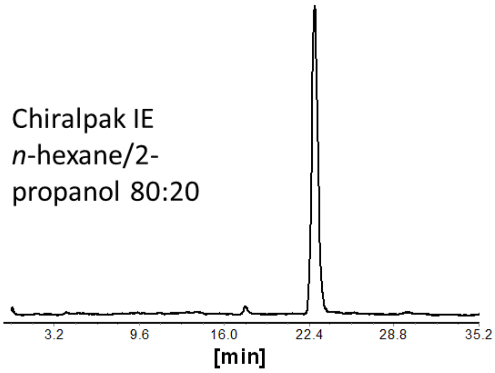

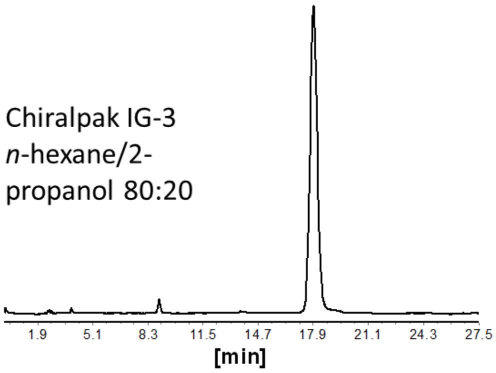

No separation of enantiomers could be observed for **5b**

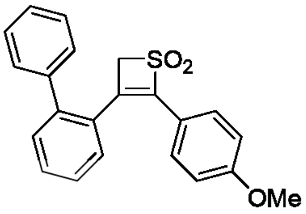

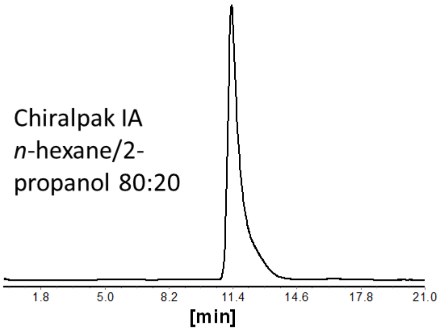

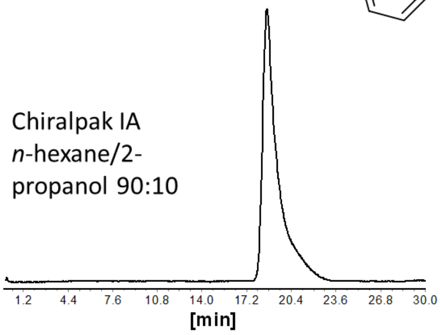

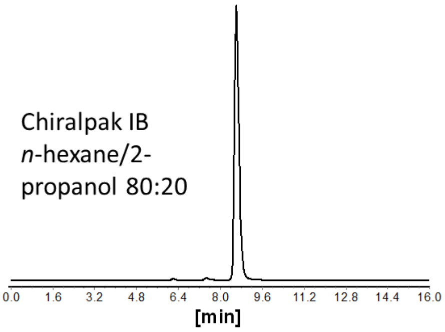

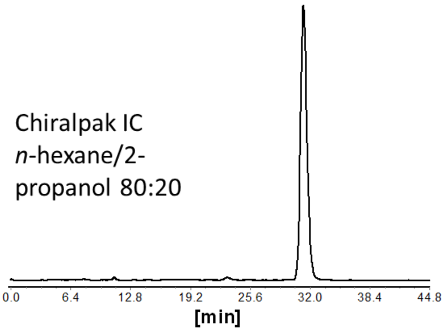

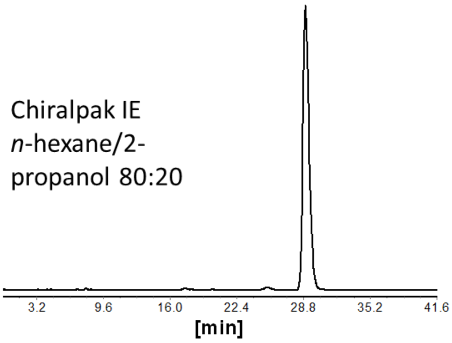

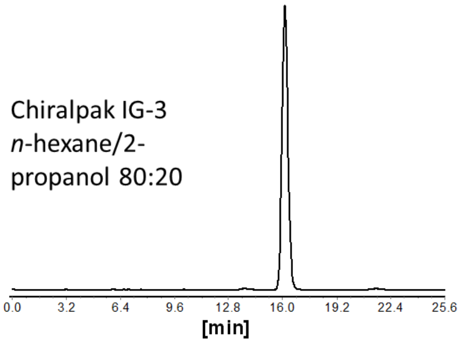

No separation of enantiomers could be observed for **5e**

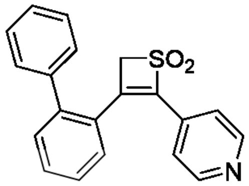

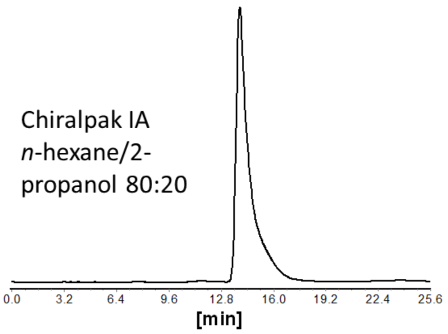

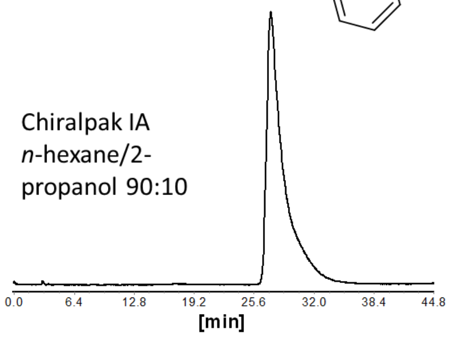

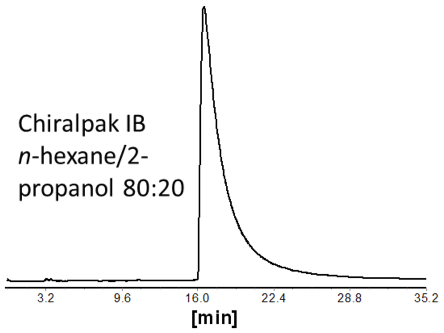

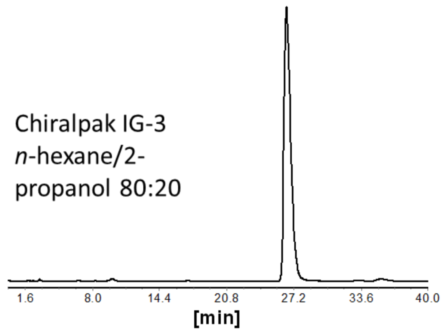

No separation of enantiomers could be observed for **5f**

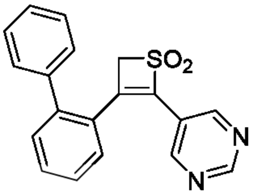

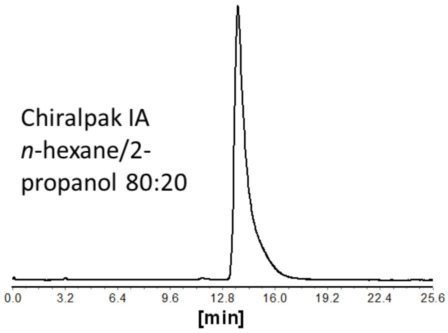

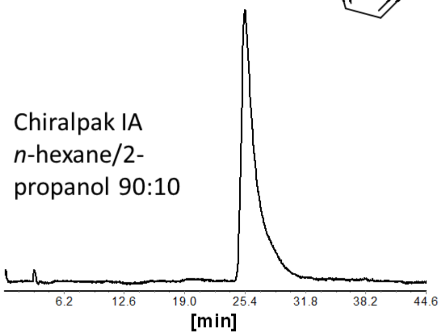

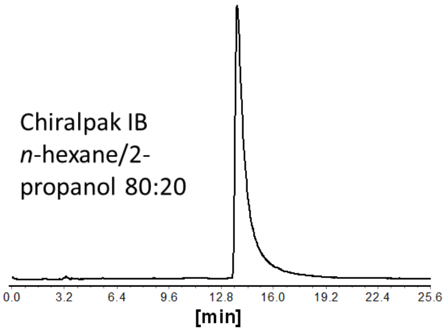

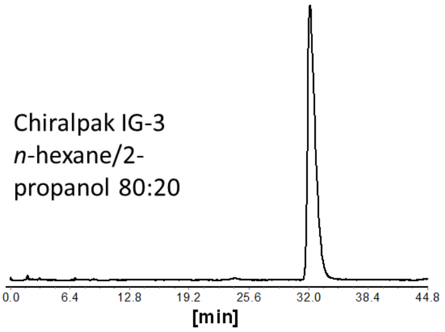

4.2 Low Temperature Measurements

No separation of enantiomers could be observed for **3d** and **4d** at T = -65°C

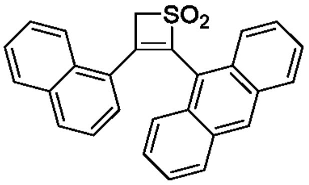

3d

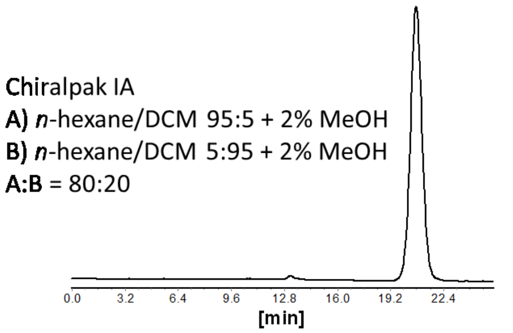

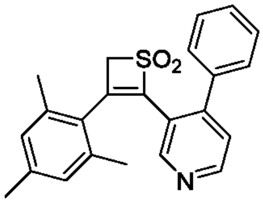

4d

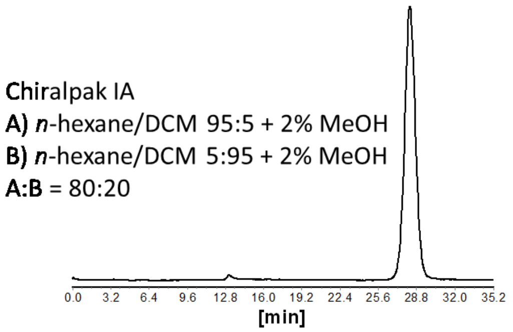

No separation of enantiomers could be observed for **4b** at T = -65°C

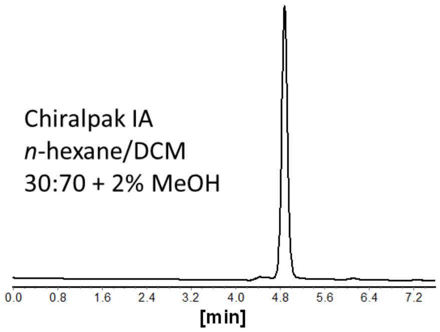

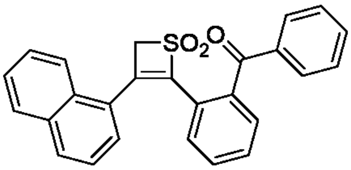

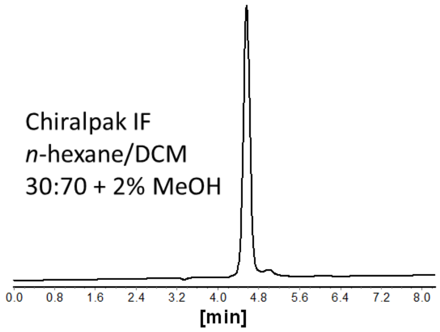

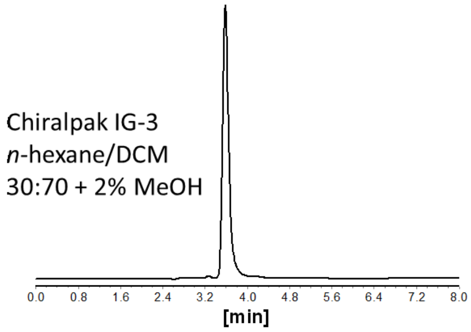

No separation of enantiomers could be observed for **6a** at T = -65°C

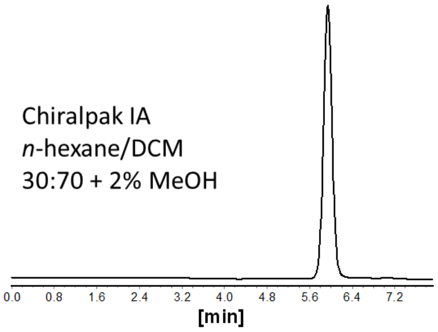

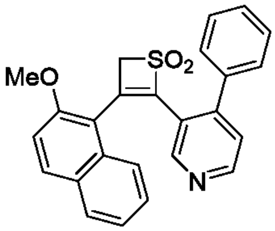

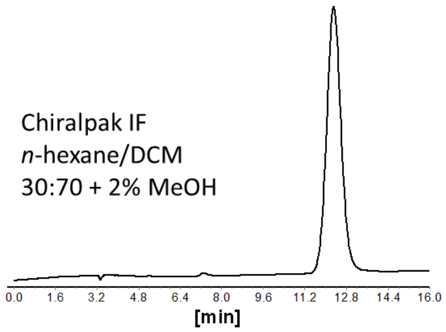

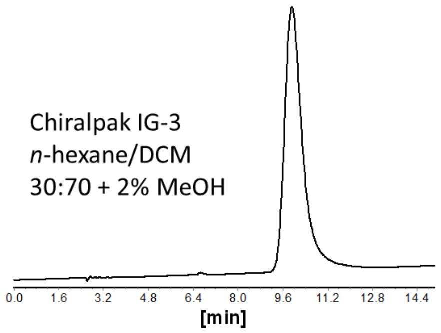

No separation of enantiomers could be observed for **6c** at T = -65°C

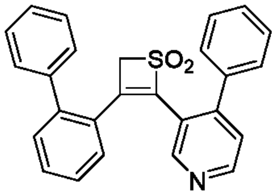

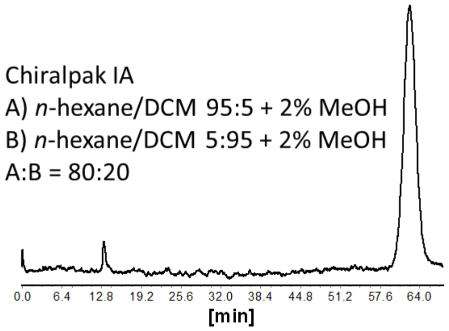

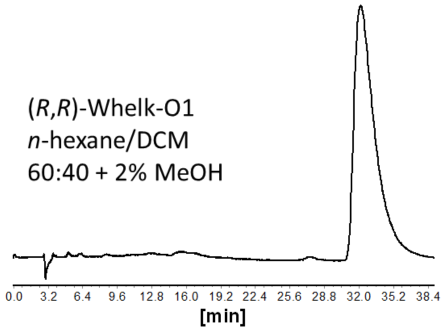

No separation of enantiomers could be observed for **6d** at T = -65°C

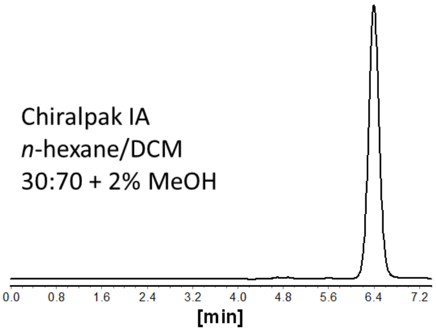

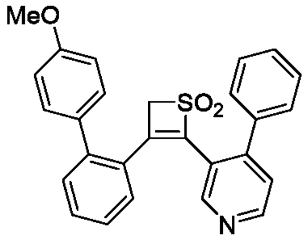

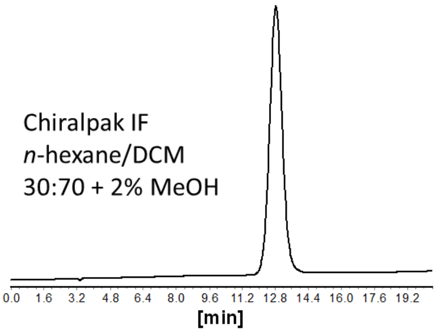

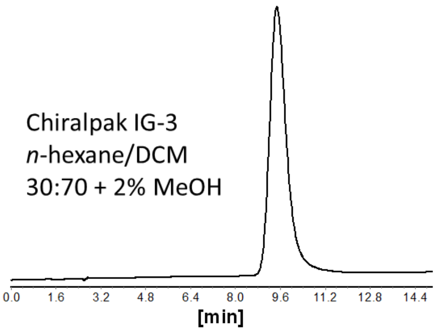

5. NMR Measurements on Axial Chirality for 6a/6d

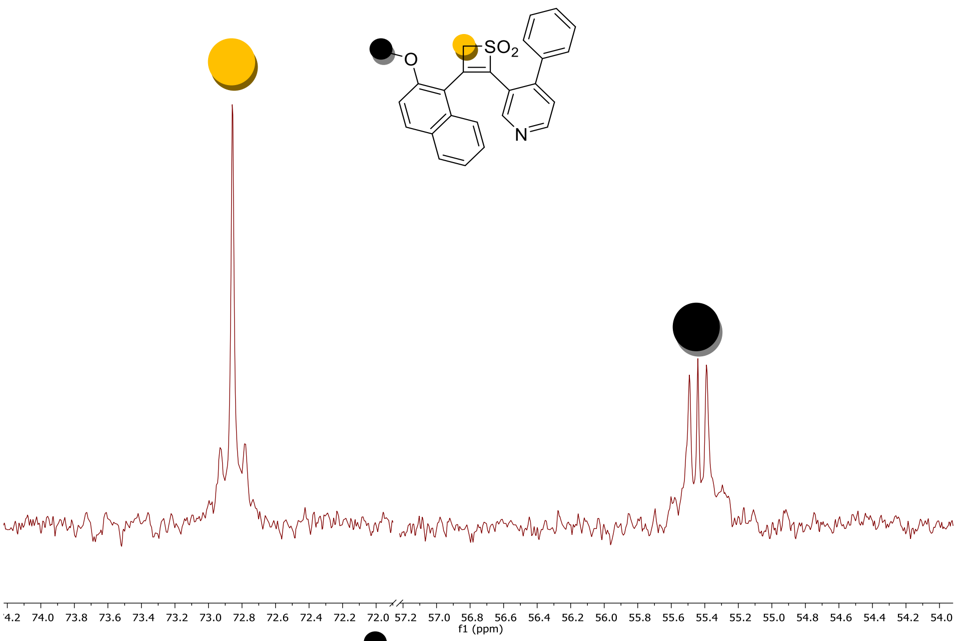

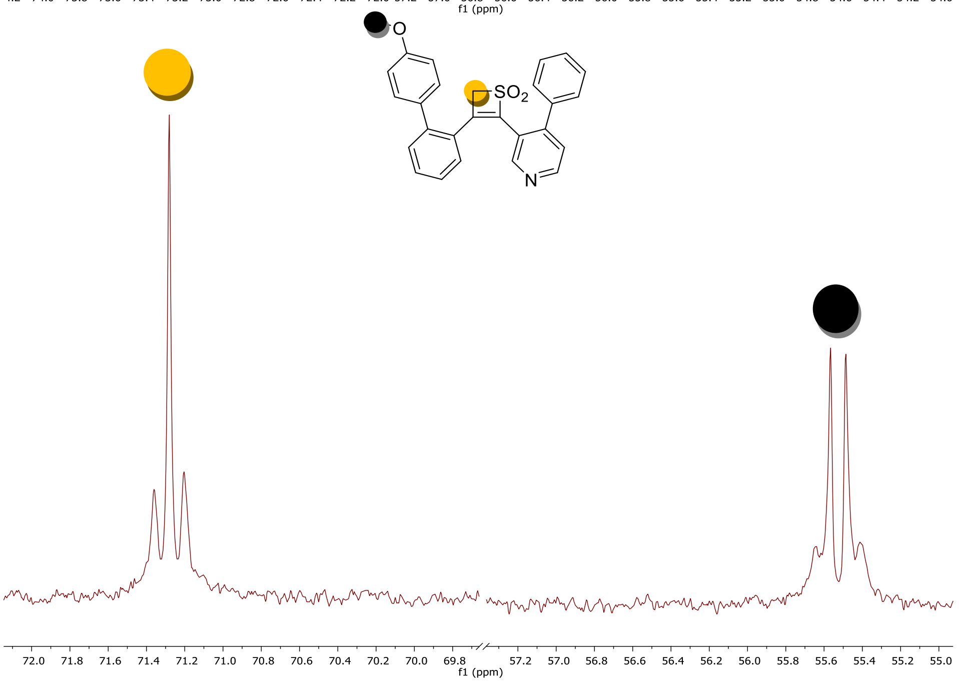

Splitting of the highlighted carbons (black and yellow) indicate the presence of conformers by axial chirality. Unfortunately, no separation of these two compounds was observed by using HPLC techniques.

6. Single Crystal X-Ray Diffraction

Structure Determinations: The intensity data of **3a**, **3d, 4a, 4b, 4f, 5a, 5b, 6c, 6d, 4d, 6b, 6a, 7a** and **7b** was collected at a temperature of 293 K (**3a**), 103 K (**3d**), 100 K (**4a**), 299 K (**4b**), 100 K (**4f**), 143 K (**5a**), 143 K (**5b**), 100 K (**6c**), 100 K (**6d**), 100 K (**4d**), 143 K (**6b**), 298 K (**6a**), 296 K (**7a**), 110 K (**7e**) on a Bruker D8 Venture TXS diffractometer using Mo-K_{α} radiation (λ = 0.71073 Å). The structures were solved by direct methods (SHELXT)[4] and refined by full-matrix least squares techniques against F_o^2 (SHELXL-2014/7)[5].

Supporting Information available: Crystallographic data have been deposited with the Cambridge Crystallographic Data Centre: CCDC-1955894for **3a;** CCDC-1955895 for **3d**; CCDC-1955896 for **4a**; CCDC- 1955897 for **4b**; CCDC- 1955898 for **5a**; CCDC-1955899 for **5b**; CCDC-1955900 for **6c**; CCDC-1955901 for **6d**; CCDC-1955902 for **4d**; CCDC-1956145 for **4f**; CCDC-1955903 for **6b**; CCDC-1955904 for **6a**; CCDC-1955905 for **7a**; CCDC-1955906 for **7e**. Copies of the data can be obtained free of charge: https://www.ccdc.cam.ac.uk/structures/.

[4] Sheldrick, G. M. (2015). *Acta Cryst.* A**71**, 3-8.
[5] Sheldrick, G. M. (2015). *Acta Cryst.* C**71**, 3-8.

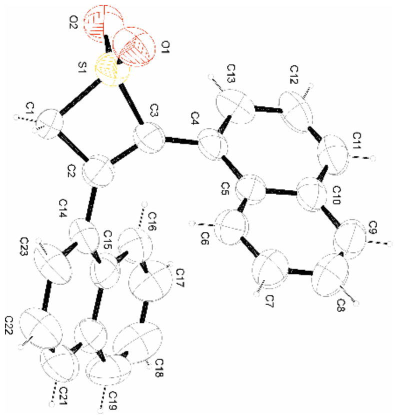

	**3a**
net formula	$C_{23}H_{16}O_2S$
M_r/g mol^{-1}	356.42
crystal size/mm	0.100 × 0.060 × 0.030
T/K	293.(2)
radiation	MoKα
diffractometer	'Bruker D8 Venture TXS'
crystal system	triclinic
space group	'P -1'
a/Å	8.0586(5)
b/Å	13.8610(8)
c/Å	16.3574(11)
α/°	89.714(2)
β/°	78.891(3)
γ/°	89.717(2)
V/Å^3	1792.85(19)
Z	4
calc. density/g cm^{-3}	1.320
μ/mm^{-1}	0.194
absorption correction	Multi-Scan
transmission factor range	0.71–0.99
refls. measured	6116
R_{int}	0.0603
mean σ(I)/I	0.0823
θ range	3.197–25.027
observed refls.	4010
x, y (weighting scheme)	0.1008, 0.2422
hydrogen refinement	constr
refls in refinement	6116
parameters	470
restraints	0
$R(F_{obs})$	0.0728
$R_w(F^2)$	0.1926
S	1.064
shift/error$_{max}$	0.001
max electron density/e Å^{-3}	0.302
min electron density/e Å^{-3}	−0.301

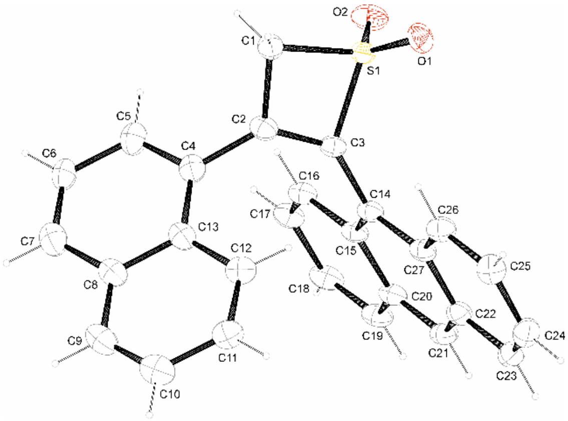

	**3d**
net formula	$C_{27}H_{18}O_2S$
M_r/g mol^{-1}	406.47
crystal size/mm	0.100 × 0.050 × 0.040
T/K	103.(2)
radiation	MoKα
diffractometer	'Bruker D8 Venture TXS'
crystal system	monoclinic
space group	'P 1 21/c 1'
a/Å	9.8504(4)
b/Å	18.7839(6)
c/Å	10.5297(4)
α/°	90
β/°	99.0900(10)
γ/°	90
V/Å^3	1923.83(12)
Z	4
calc. density/g cm^{-3}	1.403
μ/mm^{-1}	0.191
absorption correction	Multi-Scan
transmission factor range	0.95–0.99
refls. measured	19079
R_{int}	0.0414
mean σ(*I*)/*I*	0.0352
θ range	3.271–27.099
observed refls.	3467
x, y (weighting scheme)	0.0426, 2.6914
hydrogen refinement	constr
refls in refinement	4231
parameters	271
restraints	0
$R(F_{obs})$	0.0505
$R_w(F^2)$	0.1262
S	1.041
shift/error$_{max}$	0.001
max electron density/e Å^{-3}	0.848
min electron density/e Å^{-3}	−0.492

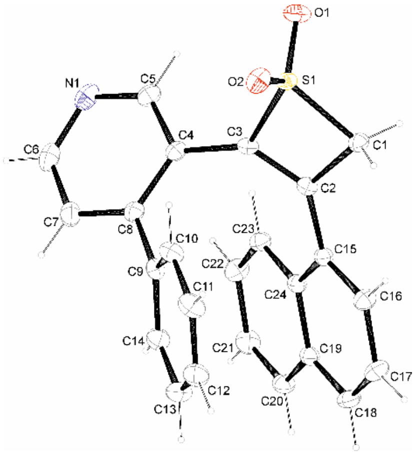

	**4a**
net formula	$C_{24}H_{17}NO_2S$
M_r/g mol^{-1}	383.44
crystal size/mm	0.100 × 0.060 × 0.050
T/K	100.(2)
radiation	MoKα
diffractometer	'Bruker D8 Venture TXS'
crystal system	triclinic
space group	'P -1'
a/Å	8.6496(4)
b/Å	10.5111(5)
c/Å	11.7560(6)
α/°	84.633(2)
β/°	70.467(2)
γ/°	68.127(2)
V/Å^3	934.20(8)
Z	2
calc. density/g cm^{-3}	1.363
μ/mm^{-1}	0.193
absorption correction	Multi-Scan
transmission factor range	0.93–0.99
refls. measured	9803
R_{int}	0.0194
mean σ(I)/I	0.0256
θ range	3.680–27.103
observed refls.	3702
x, y (weighting scheme)	0.0376, 0.5497
hydrogen refinement	constr
refls in refinement	4084
parameters	253
restraints	0
$R(F_{obs})$	0.0328
$R_w(F^2)$	0.0858
S	1.024
shift/error$_{max}$	0.001
max electron density/e Å^{-3}	0.392
min electron density/e Å^{-3}	−0.414

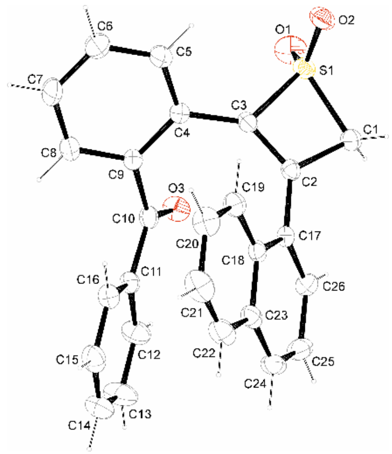

	**4b**
net formula	$C_{26}H_{18}O_3S$
M_r/g mol^{-1}	410.46
crystal size/mm	0.090 × 0.070 × 0.050
T/K	299.(2)
radiation	MoKα
diffractometer	'Bruker D8 Venture TXS'
crystal system	monoclinic
space group	'P 1 21/c 1'
a/Å	9.6180(11)
b/Å	14.8477(16)
c/Å	15.1188(11)
α/°	90
β/°	107.960(3)
γ/°	90
V/Å^3	2053.8(4)
Z	4
calc. density/g cm^{-3}	1.327
μ/mm^{-1}	0.183
absorption correction	Multi-Scan
transmission factor range	0.95–0.99
refls. measured	18827
R_{int}	0.0478
mean σ(I)/I	0.0380
θ range	3.148–25.349
observed refls.	2888
x, y (weighting scheme)	0.0342, 1.1300
hydrogen refinement	constr
refls in refinement	3751
parameters	271
restraints	0
$R(F_{obs})$	0.0430
$R_w(F^2)$	0.1017
S	1.024
shift/error$_{max}$	0.001
max electron density/e Å^{-3}	0.236
min electron density/e Å^{-3}	−0.380

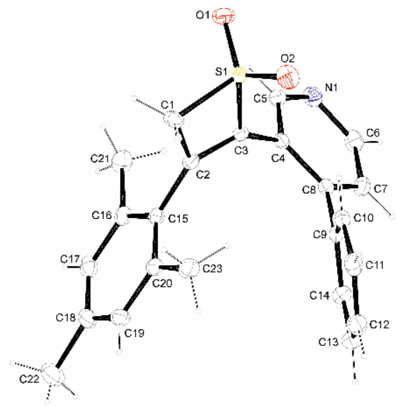

	**4d**
net formula	$C_{23}H_{21}NO_2S$
M_r/g mol^{-1}	375.47
crystal size/mm	0.080 × 0.060 × 0.050
T/K	100.(2)
radiation	MoKα
diffractometer	'Bruker D8 Venture TXS'
crystal system	monoclinic
space group	'P 1 21/c 1'
a/Å	9.2628(3)
b/Å	18.3007(5)
c/Å	11.2957(4)
α/°	90
β/°	97.6550(10)
γ/°	90
V/Å^3	1897.73(10)
Z	4
calc. density/g cm^{-3}	1.314
μ/mm^{-1}	0.188
absorption correction	Multi-Scan
transmission factor range	0.93–0.99
refls. measured	20040
R_{int}	0.0306
mean σ(*I*)/*I*	0.0254
θ range	3.143–27.484
observed refls.	3759
x, y (weighting scheme)	0.0400, 1.1560
hydrogen refinement	constr
refls in refinement	4334
parameters	247
restraints	0
$R(F_{obs})$	0.0367
$R_w(F^2)$	0.0927
S	1.034
shift/error$_{max}$	0.001
max electron density/e Å^{-3}	0.338
min electron density/e Å^{-3}	−0.433

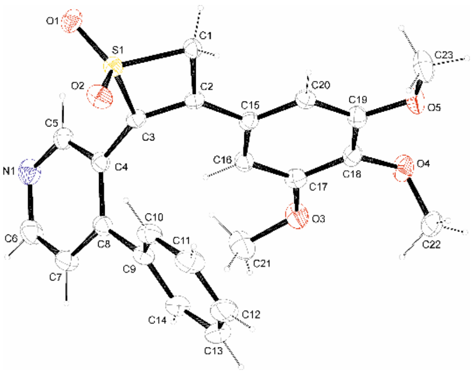

	**4f**
net formula	$C_{23}H_{21}NO_5S$
M_r/g mol^{-1}	423.47
crystal size/mm	0.100 × 0.070 × 0.050
T/K	100.(2)
radiation	MoKα
diffractometer	'Bruker D8 Venture TXS'
crystal system	monoclinic
space group	'P 1 21/n 1'
a/Å	11.9000(4)
b/Å	10.8787(3)
c/Å	17.0090(5)
α/°	90
β/°	110.1320(10)
γ/°	90
V/Å^3	2067.39(11)
Z	4
calc. density/g cm^{-3}	1.361
μ/mm^{-1}	0.192
absorption correction	Multi-Scan
transmission factor range	0.92–0.99
refls. measured	18208
R_{int}	0.0300
mean σ(*I*)/*I*	0.0276
θ range	3.165–27.102
observed refls.	3796
x, *y* (weighting scheme)	0.0397, 0.9523
hydrogen refinement	constr
refls in refinement	4540
parameters	274
restraints	0
$R(F_{obs})$	0.0339
$R_w(F^2)$	0.0890
S	1.017
shift/error$_{max}$	0.001
max electron density/e Å^{-3}	0.270
min electron density/e Å^{-3}	−0.406

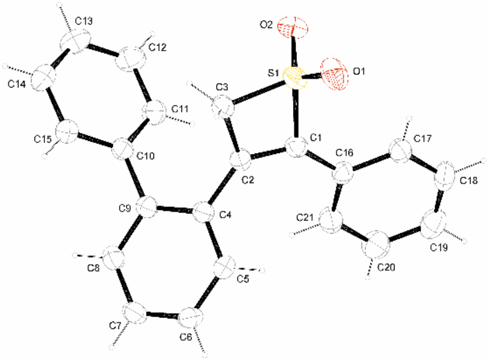

	**5a**
net formula	$C_{21}H_{16}O_2S$
M_r/g mol^{-1}	332.40
crystal size/mm	0.362 × 0.278 × 0.221
T/K	143(2)
radiation	MoKα
diffractometer	'Oxford XCalibur'
crystal system	monoclinic
space group	'P 21/n'
a/Å	9.6709(8)
b/Å	10.5272(7)
c/Å	16.3722(13)
α/°	90
β/°	94.907(7)
γ/°	90
V/Å^3	1660.7(2)
Z	4
calc. density/g cm^{-3}	1.329
μ/mm^{-1}	0.204
absorption correction	multi-scan
transmission factor range	0.88636–1.00000
refls. measured	9249
R_{int}	0.0368
mean σ(I)/I	0.0399
θ range	4.218–24.995
observed refls.	2271
x, y (weighting scheme)	0.0398, 0.6296
hydrogen refinement	constr
refls in refinement	2916
parameters	217
restraints	0
$R(F_{obs})$	0.0398
$R_w(F^2)$	0.1047
S	1.041
shift/error$_{max}$	0.001
max electron density/e Å^{-3}	0.310
min electron density/e Å^{-3}	−0.325

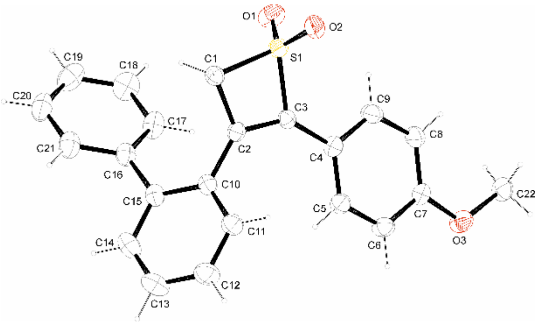

	**5b**
net formula	$C_{22}H_{18}O_3S$
M_r/g mol^{-1}	362.42
crystal size/mm	0.296 × 0.242 × 0.103
T/K	143(2)
radiation	MoKα
diffractometer	'Oxford XCalibur'
crystal system	monoclinic
space group	'C c'
a/Å	20.6412(17)
b/Å	5.4509(3)
c/Å	17.8630(12)
α/°	90
β/°	114.456(9)
γ/°	90
V/Å^3	1829.5(2)
Z	4
calc. density/g cm^{-3}	1.316
μ/mm^{-1}	0.195
absorption correction	multi-scan
transmission factor range	0.85807–1.00000
refls. measured	4836
R_{int}	0.0318
mean σ(I)/I	0.0545
θ range	4.338–27.946
observed refls.	2912
x, y (weighting scheme)	0.0440, 0.0390
hydrogen refinement	constr
Flack parameter	−0.02(7)
refls in refinement	3200
parameters	236
restraints	2
$R(F_{obs})$	0.0399
$R_w(F^2)$	0.0965
S	1.063
shift/error$_{max}$	0.001
max electron density/e Å^{-3}	0.257
min electron density/e Å^{-3}	−0.274

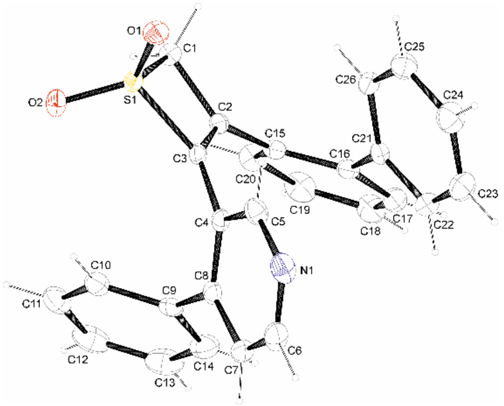

	**6c**
net formula	$C_{26}H_{19}NO_2S$
M_r/g mol^{-1}	409.48
crystal size/mm	0.090 × 0.070 × 0.040
T/K	100.(2)
radiation	MoKα
diffractometer	'Bruker D8 Venture TXS'
crystal system	monoclinic
space group	'C 1 2/c 1'
a/Å	22.6159(5)
b/Å	8.5749(2)
c/Å	21.2839(5)
α/°	90
β/°	91.8770(10)
γ/°	90
V/Å^3	4125.35(16)
Z	8
calc. density/g cm^{-3}	1.319
μ/mm^{-1}	0.180
absorption correction	Multi-Scan
transmission factor range	0.94–0.99
refls. measured	20750
R_{int}	0.0358
mean σ(*I*)/*I*	0.0283
θ range	3.164–26.372
observed refls.	3580
x, *y* (weighting scheme)	0.0310, 5.1208
hydrogen refinement	constr
refls in refinement	4202
parameters	271
restraints	0
$R(F_{obs})$	0.0364
$R_w(F^2)$	0.0850
S	1.044
shift/error$_{max}$	0.001
max electron density/e Å^{-3}	0.361
min electron density/e Å^{-3}	−0.404

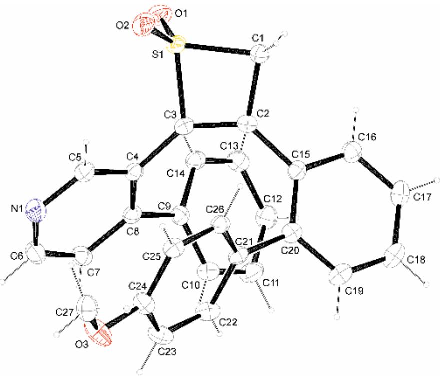

	**6d**
net formula	$C_{27}H_{21}NO_3S$
M_r/g mol^{-1}	439.51
crystal size/mm	0.080 × 0.070 × 0.040
T/K	100.(2)
radiation	MoKα
diffractometer	'Bruker D8 Venture TXS'
crystal system	monoclinic
space group	'P 1 21/n 1'
a/Å	8.9875(2)
b/Å	13.8360(4)
c/Å	17.3172(4)
α/°	90
β/°	100.4190(10)
γ/°	90
V/Å^3	2117.91(9)
Z	4
calc. density/g cm^{-3}	1.378
μ/mm^{-1}	0.184
absorption correction	Multi-Scan
transmission factor range	0.95–0.99
refls. measured	22311
R_{int}	0.0304
mean σ(I)/I	0.0246
θ range	3.147–27.102
observed refls.	4056
x, y (weighting scheme)	0.0352, 1.3894
hydrogen refinement	constr
refls in refinement	4666
parameters	290
restraints	0
$R(F_{obs})$	0.0354
$R_w(F^2)$	0.0902
S	1.034
shift/error$_{max}$	0.001
max electron density/e Å^{-3}	0.314
min electron density/e Å^{-3}	−0.461

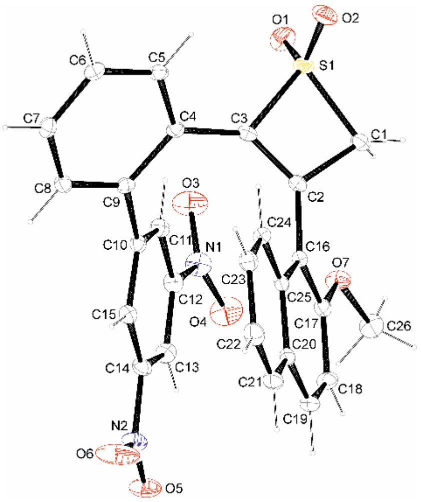

	**6b**
net formula	$C_{26}H_{18}N_2O_7S$
M_r/g mol^{-1}	502.48
crystal size/mm	0.368 × 0.255 × 0.145
T/K	143(2)
radiation	MoKα
diffractometer	'Oxford XCalibur'
crystal system	monoclinic
space group	'P 21/n'
a/Å	7.8118(4)
b/Å	35.594(2)
c/Å	8.7474(5)
α/°	90
β/°	110.609(6)
γ/°	90
V/Å^3	2276.6(2)
Z	4
calc. density/g cm^{-3}	1.466
μ/mm^{-1}	0.195
absorption correction	multi-scan
transmission factor range	0.93787–1.00000
refls. measured	12599
R_{int}	0.0358
mean σ(*I*)/*I*	0.0466
θ range	4.242–26.370
observed refls.	3698
x, *y* (weighting scheme)	0.0242, 1.6852
hydrogen refinement	constr
refls in refinement	4629
parameters	326
restraints	0
$R(F_{obs})$	0.0476
$R_w(F^2)$	0.0996
S	1.068
shift/error$_{max}$	0.001
max electron density/e Å^{-3}	0.315
min electron density/e Å^{-3}	−0.325

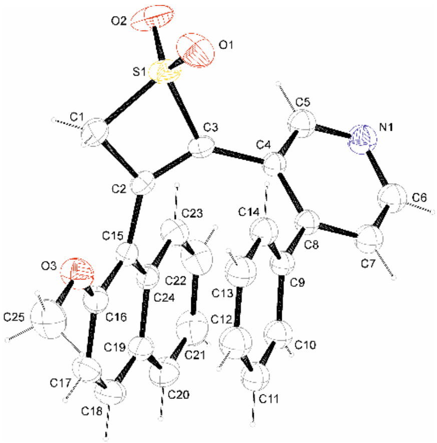

	**6a**
net formula	$C_{25}H_{19}NO_3S$
M_r/g mol^{-1}	413.47
crystal size/mm	0.080 × 0.060 × 0.050
T/K	298.(2)
radiation	MoKα
diffractometer	'Bruker D8 Venture TXS'
crystal system	monoclinic
space group	'P 1 21/n 1'
a/Å	11.0210(7)
b/Å	15.2877(9)
c/Å	12.5569(6)
α/°	90
β/°	94.216(2)
γ/°	90
V/Å^3	2109.9(2)
Z	4
calc. density/g cm^{-3}	1.302
μ/mm^{-1}	0.180
absorption correction	Multi-Scan
transmission factor range	0.94–0.99
refls. measured	21700
R_{int}	0.0405
mean σ(I)/I	0.0302
θ range	3.246–26.371
observed refls.	3096
x, y (weighting scheme)	0.0529, 1.5771
hydrogen refinement	constr
refls in refinement	4300
parameters	261
restraints	0
$R(F_{obs})$	0.0534
$R_w(F^2)$	0.1458
S	1.032
shift/error$_{max}$	0.001
max electron density/e Å^{-3}	0.277
min electron density/e Å^{-3}	−0.377

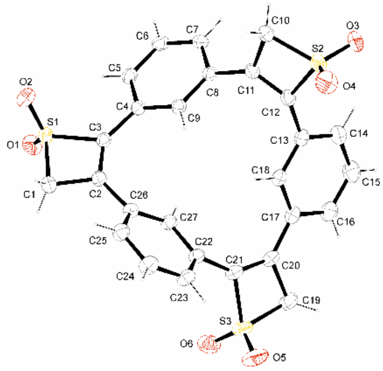

	**7a**
net formula	$C_{27}H_{18}O_6S_3$
M_r/g mol^{-1}	534.59
crystal size/mm	0.100 × 0.060 × 0.050
T/K	296.(2)
radiation	MoKα
diffractometer	'Bruker D8Quest'
crystal system	triclinic
space group	'P -1'
a/Å	10.4939(3)
b/Å	11.1477(3)
c/Å	12.1923(3)
α/°	108.6230(10)
β/°	95.7060(10)
γ/°	97.9900(10)
V/Å^3	1322.68(6)
Z	2
calc. density/g cm^{-3}	1.342
μ/mm^{-1}	0.319
absorption correction	Multi-Scan
transmission factor range	0.95–0.98
refls. measured	25275
R_{int}	0.0251
mean σ(*I*)/*I*	0.0246
θ range	3.292–26.369
observed refls.	4706
x, y (weighting scheme)	0.0963, 0.7693
hydrogen refinement	constr
Flack parameter	?
refls in refinement	5374
parameters	325
restraints	0
$R(F_{obs})$	0.0498
$R_w(F^2)$	0.1652
S	1.086
shift/error$_{max}$	0.001
max electron density/e Å^{-3}	0.410
min electron density/e Å^{-3}	−0.503

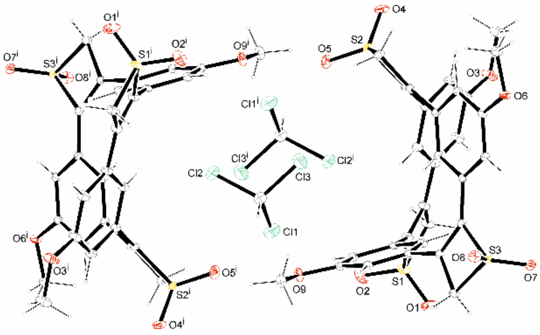

	**7e**
net formula	$C_{31}H_{25}Cl_3O_9S_3$
M_r/g mol^{-1}	744.04
crystal size/mm	0.100 × 0.090 × 0.080
T/K	110.(2)
radiation	MoKα
diffractometer	'Bruker D8 Venture TXS'
crystal system	triclinic
space group	'P -1'
a/Å	11.7054(6)
b/Å	12.3313(7)
c/Å	14.5166(8)
α/°	93.996(2)
β/°	102.009(2)
γ/°	106.053(2)
V/Å^3	1951.47(19)
Z	2
calc. density/g cm^{-3}	1.266
μ/mm^{-1}	0.440
absorption correction	Multi-Scan
transmission factor range	0.88–0.97
refls. measured	21459
R_{int}	0.0248
mean σ(I)/I	0.0392
θ range	2.460–28.281
observed refls.	7984
x, y (weighting scheme)	0.0514, 2.3137
hydrogen refinement	constr
refls in refinement	9597
parameters	418
restraints	0
$R(F_{obs})$	0.0440
$R_w(F^2)$	0.1192
S	1.029
shift/error$_{max}$	0.001
max electron density/e Å^{-3}	0.623
min electron density/e Å^{-3}	−0.651

All but one solvent CHCl3 have been SQUEEZED out.

Symmetry code for figure above: i = -x, -y, 1-z.
